# Synthesis, Spectroscopic Properties, and Metalation of 3-Alkoxybenziporphyrins

**DOI:** 10.3390/molecules29081903

**Published:** 2024-04-22

**Authors:** Rachel A. Tomlovich, Timothy D. Lash

**Affiliations:** Department of Chemistry, Illinois State University, Normal, IL 61790-4160, USA; racheltomlovich@gmail.com

**Keywords:** carbaporphyrinoids, benziporphyrins, aromaticity, organometallic compounds

## Abstract

A series of 5-alkoxy-1,3-benzenedicarbaldehydes and related dimers were prepared in three steps from dimethyl 5-hydroxyisophthalate. Acid catalyzed condensation of the dialdehydes with a tripyrrane dicarboxylic acid, followed by oxidation with 2,3-dichloro-5,6-dicyano-1,4-benzoquinone, afforded good yields of 3-alkoxybenziporphyrins, although dimeric tetraaldehydes failed to give isolatable porphyrinoid products. Proton NMR spectroscopy gave no indication of an aromatic ring current, but addition of trifluoroacetic acid resulted in the formation of dications that exhibited weakly diatropic characteristics. Spectroscopic titration with TFA demonstrated that stepwise protonation took place, generating monocationic and dicationic species. 3-Alkoxybenziporphyrins reacted with nickel(II) or palladium(II) acetate to give the related nickel(II) or palladium(II) complexes. These stable organometallic derivatives showed increased diatropic properties that were most pronounced for the palladium(II) complexes. These unique porphyrinoids provide further insights into the properties of benziporphyrins.

## 1. Introduction

**Figure 1:**
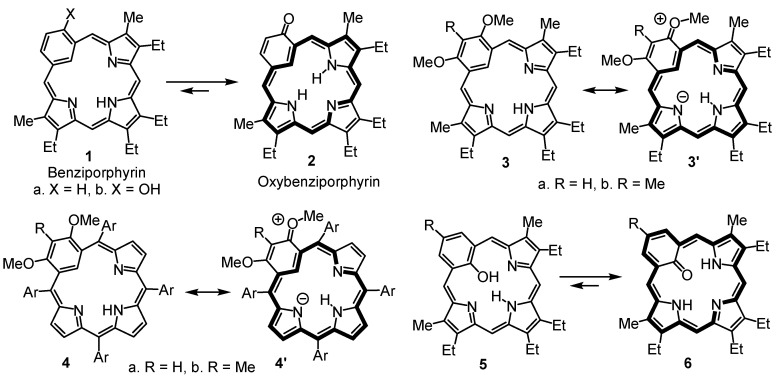
Selected examples of benziporphyrins.

Benziporphyrins [1,2], e.g., **1a**, porphyrin analogues that possess a benzene moiety in place of one of the pyrrolic units, are examples of highly modified carbaporphyrinoid systems [3]. Unlike true porphyrins, benziporphyrin **1a** (Figure 1) is nonaromatic, although a weak global diatropic ring current manifests upon protonation [4,5,6]. On the other hand, 2-hydroxybenziporphyrin **1b** favors a fully aromatic semiquinone tautomer **2** that exhibits dramatically reduced diatropicity upon protonation in the presence of excess acid due to the formation of a phenolic dication [4,7]. 2,4-Dimethoxybenziporphyrin **3a** [8] and related *meso*-tetraarylbenziporphyrins **4a** [9,10] exhibit macrocyclic ring currents due to the electron-donating methoxy groups facilitating dipolar canonical forms such as **3a′** and **4a′**, which possess 18π electron delocalization pathways. The aromatic character of dimethoxybenziporphyrins **3b** and **4b** is considerably reduced because steric congestion due to the 3-methyl group prevents the methoxy units from lying coplanar with the benzene ring, thereby undermining the electronic interactions responsible for resonance contributors **3′** and **4′** [8,9,10]. Protonation of **3a** or **4a** with excess trifluoroacetic acid gave strongly aromatic dications **3a**H_2_^2+^ and **4a**H_2_^2+^. Surprisingly, 22-hydroxybenziporphyrin **5** has been shown to favor an antiaromatic keto-tautomer **6** [11,12], further demonstrating the versatility of this family of carbaporphyrinoids.

Due to their intriguing properties, benziporphyrins have been widely investigated and have been shown to form a range of stable organometallic complexes [2,13,14,15,16,17]. In addition, benziporphyrin derivatives have shown promise as fluorescent zinc ion detectors [18] and as components of nanomolecular assemblies [19].

**Scheme 1:**
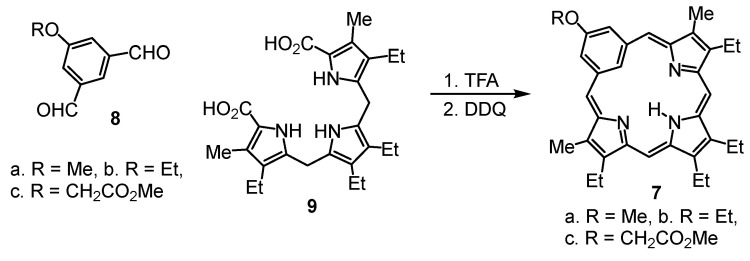
The ‘3 + 1’ synthesis of 3-alkoxybenziporphyrins.

In a continuation of our studies in this area, a series of 3-alkoxybenziporphyrins **7** (Scheme 1) were targeted for synthesis. The properties of these substituted benziporphyrins were assessed and metalation studies were performed. In addition, the potential use of this substitution pattern to generate a tether between two porphyrinoid units was considered.

## 2. Results and Discussion

**Scheme 2:**
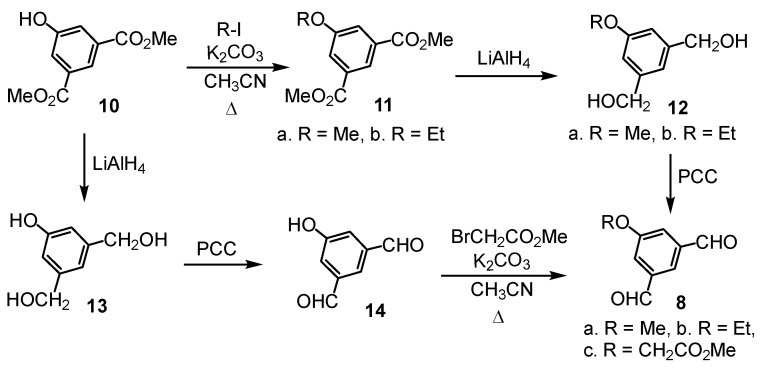
Synthesis of 5-alkoxyisophthalaldehydes.

Alkoxybenziporphyrins **7a–c** were prepared using the ‘3 + 1’ variant of the MacDonald condensation (Scheme 1) [20,21]. The key intermediates were 5-alkoxyisophthalaldehydes **8a–c** and tripyrrane dicarboxylic acid **9** [22,23]. The dialdehydes were prepared in turn from commercially available dimethyl 5-hydroxyisophthalate **10** (Scheme 2). Alkylation of **10** with methyl or ethyl iodide and potassium carbonate in refluxing acetonitrile gave methoxy and ethoxy derivatives **11a** and **11b**, respectively. Reduction with lithium aluminum hydride in THF afforded the corresponding dialcohols **12a** and **12b**, and subsequent treatment with pyridinium chlorochromate (PCC) gave dialdehydes **8a** and **8b**. Related dialdehyde **8c** was prepared by an alternative route. Reduction of **10** with lithium aluminum hydride gave phenolic dicarbinol **13**, and subsequent oxidation with PCC afforded dialdehyde **14**. Reaction with methyl bromoacetate and potassium carbonate then furnished aryloxyacetate dialdehyde **8c**.

Tripyrrane **9** was treated with trifluoroacetic acid (TFA), the reaction solution diluted with dichloromethane, and the intermediate condensed with dialdehydes **8a–c**. Following oxidation with 2,3-dichloro-5,6-dicyano-1,4-benzoquinone (DDQ), purification by column chromatography on grade 3 alumina, and recrystallization from chloroform-methanol, 3-alkoxybenziporphyrins **7a–c** were isolated as dark-purple crystals in 34–44% yield. Interestingly, no porphyrinoid products could be isolated when phenolic dialdehyde **14** was reacted with tripyrrane **9**, possibly due to the instability of hydroxybenziporphyrins. As had been expected, the proton NMR spectra for **7a–c** showed no indication of an aromatic ring current. Porphyrins, which exhibit exceptionally powerful diamagnetic ring currents, give strongly deshielded resonances for the external protons, while the internal protons are shifted atypically upfield [24,25]. For instance, the bridging methine protons (*meso*-protons) in porphyrins commonly appear downfield near +10 ppm, while the inner N-H protons are generally observed upfield near −4 ppm.

**Scheme 3:**
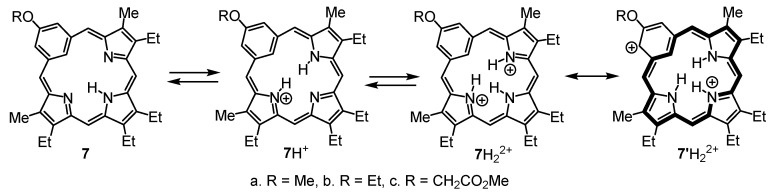
Protonation of 3-alkoxybenziporphyrins.

**Figure 2:**
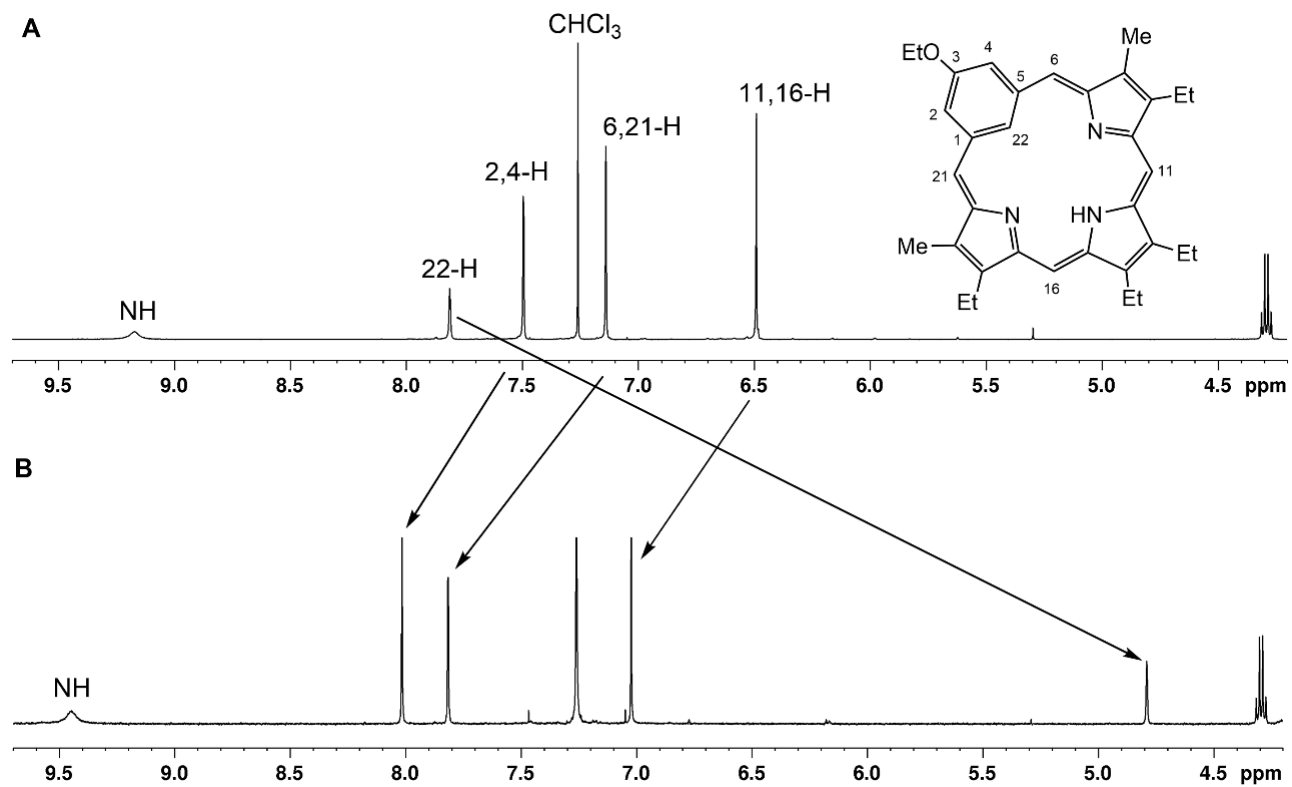
Partial 500 MHz proton NMR spectra of ethoxybenziporphyrin **7b** in CDCl_3_ (**A**) and the related diprotonated dication in TFA-CDCl_3_ (**B**). Protonation leads to the internal 22-H shifting upfield by ca. 3 ppm while the external protons are significantly deshielded, results that demonstrate the emergence of a global aromatic ring current.

The proton NMR spectrum of 3-ethoxybenziporphyrin **7b** (Figure 2) confirmed that the macrocycle possesses a plane of symmetry and demonstrated the absence of overall aromatic character. The *meso*-protons gave rise to two 2H singlets at 6.5 and 7.1 ppm, values that are consistent with a nonaromatic porphyrinoid. Furthermore, the internal C-H resonance appeared downfield at 7.8 ppm, while the N-H appeared at 8.2 ppm. Benziporphyrins **7a** and **7c** gave similar results. However, the addition of TFA to the NMR tube resulted in the emergence of weak, but nonetheless significant, aromatic character. In the case of **7b** (Figure 2), the *meso*-protons of the resulting dication **7b**H_2_^2+^ (Scheme 3) shifted downfield to give 2H singlets at 7.0 and 7.8 ppm, while the external benzene rings (2,4-H) moved from 7.5 to 8.0 ppm. However, the inner C-H (22-H) shifted upfield by 3 ppm to give a 1H resonance at 4.8 ppm (Figure 2). In the absence of any other changes, protonation would be expected to result in deshielding, so the shift associated with the 22-H resonance is significant. This result can be attributed to contributions from canonical forms such as **7b′**H_2_^2+^, which possess 18π electron pathways (Scheme 3). Nevertheless, the diatropic character for the dication is relatively weak compared to porphyrins or fully aromatic porphyrinoids such as oxybenziporphyrin **2**. Protonation of **7a** and **7c** gave similar results (Figures S49 and S84).

**Figure 3:**
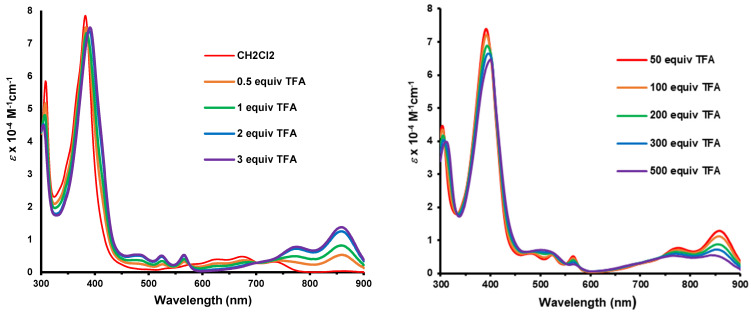
UV-vis spectra of ethoxybenziporphyrin **7b** in dichloromethane with 0–3 equivalents of TFA (**left**) and 50–500 equivalents of TFA (**right**) showing sequential mono- and diprotonation.

The UV-vis spectra of **7a–c** were also consistent with nonaromatic structures. For instance, benziporphyrin **7b** gave two moderate absorptions at 308 and 382 nm and weaker broad absorptions between 500 and 800 nm (Figure 3). Spectroscopic titration with TFA demonstrated that two sequential protonations occurred. The addition of 0.5–3 equivalents of TFA resulted in a bathochromic shift of the band at 382 nm to 391 nm, while long wavelength absorptions emerged at 776 and 858 nm (Figure 3). This was attributed to the formation of monoprotonated cation **7b**H^+^ (Scheme 3). At higher concentrations of TFA, further changes were noted, and the long wavelength bands hypsochromically shifted to 760 and 842 nm, while the Soret-like band was reduced in intensity and moved to 400 nm (Figure 3). This was attributed to the formation of dication **7b**H_2_^2+^ (Scheme 3). Similar results were obtained for **7a** and **7c** (Figures S9–S14 and S17–S18).

**Scheme 4:**
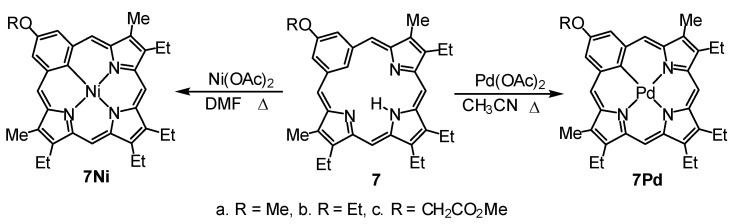
Metalation of 3-alkoxybenziporphyrins.

Metalation of 3-alkoxybenziporphyrins **7a–c** was also investigated (Scheme 4). The palladium complexes were synthesized by refluxing 3-alkoxybenziporphyrins **7a–c** in acetonitrile for 30 min in the presence of palladium(II) acetate. Following purification by column chromatography on a grade 3 basic alumina column and recrystallization from chloroform-methanol, organometallic complexes **7Pd** were isolated in a 60–73% yield. When benziporphyrins **7a–c** were refluxed in *N*,*N*-dimethylformamide (DMF) for 30 min in the presence of nickel(II) acetate and purified similarly, the corresponding nickel(II) complexes **7Ni** were obtained as dark-green solids in a 55–86% yield.

**Figure 4:**
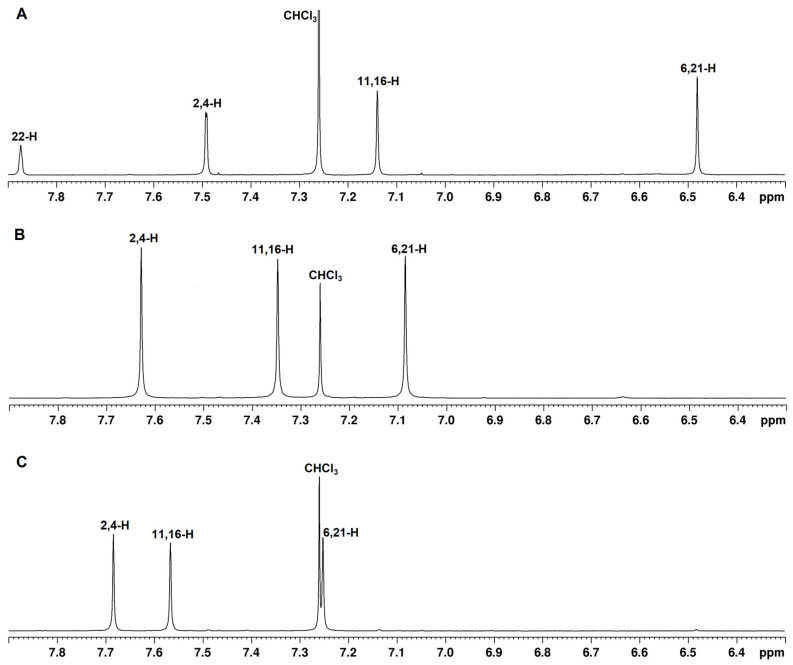
Partial proton NMR spectra of methoxybenziporphyrin **7a** (**A**) and the related nickel(II) complex **7aNi** (**B**) and palladium(II) complex **7aPd** (**C**) showing a marked increase in diatropicity upon metalation, which was most pronounced for **7aPd**.

The proton NMR spectra for the palladium and nickel complexes demonstrated that metalation induced the emergence of aromatic character in these structures (Figure 4). This was particularly evident for palladium(II) complexes **7Pd**. Figure 4 illustrates how the *meso*-protons are shifted downfield for the 3-methoxybenziporphyrin series **7a**, **7aNi**, and **7aPd**. The *meso*-protons for free base **7a** appeared at 6.48 and 7.14 ppm, but these resonances shifted to 7.08 and 7.35 ppm in **7aNi**, and 7.25 and 7.57 ppm in **7aPd**. In addition, the 2,4-protons on the arene unit were also shifted downfield from 7.49 to 7.63 to 7.68 ppm, going from **7a** to **7aNi** to **7aPd**. Although the observed global ring currents are weak, the results show significant changes, and these were replicated for the metal complexes of **7b** and **7c**. The carbon-13 NMR spectra showed the *meso*-carbon resonances at 95.3 (11,16-CH) and 123.2 ppm (6,21-CH) for **7aNi**, while these peaks appeared at 95.5 and 126.6 ppm, respectively, for **7aPd**. As was the case for the non-metalated benziporphyrins, the *meso*-carbons flanking the phenylene unit are ca. 30 ppm further downfield than the *meso*-carbons connecting two pyrrolic moieties. Similar results were obtained for **7bNi**, **7cNi**, **7bPd**, and **7cPd** (Figures S69, S74, S88 and S93).

**Figure 5:**
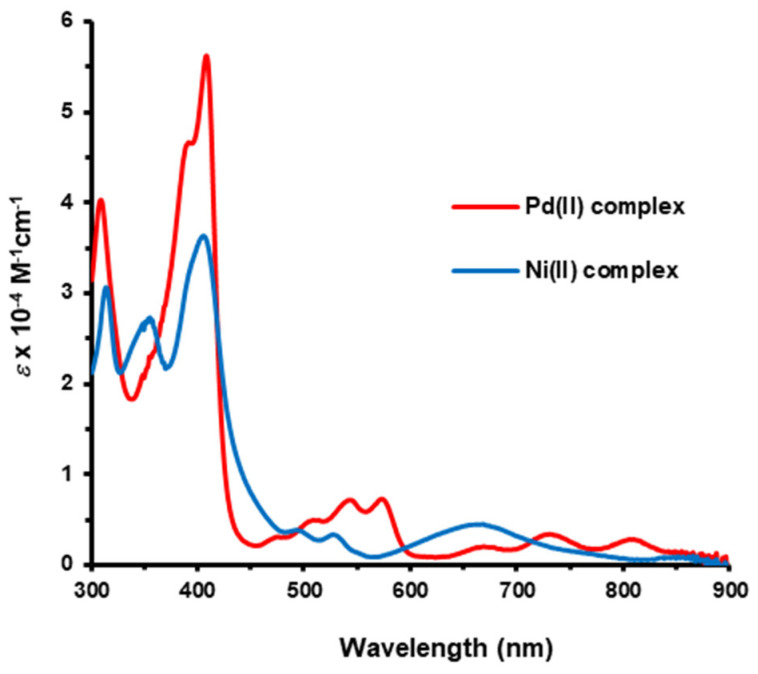
UV-vis spectra of **7aNi** (blue) and **7aPd** (red) in CH_2_Cl_2_.

The UV-Vis spectra of the metalated species also showed characteristic features (Figure 5). Palladium complex **7aPd** showed a strong Soret-like absorbance at 407 nm, a shoulder at 391 nm, and a weaker absorbance at band 308 nm. Multiple Q-type absorbances appeared between 407 and 850 nm. However, nickel(II) complex **7aNi** gave very different results, showing three moderately strong absorptions at 313, 353, and 405 nm and a broad band centered on 660 nm (Figure 5). Similar spectra were obtained for the nickel(II) and palladium(II) complexes of **7b** and **7c** (Figures S7, S8, S19 and S20).

**Scheme 5:**
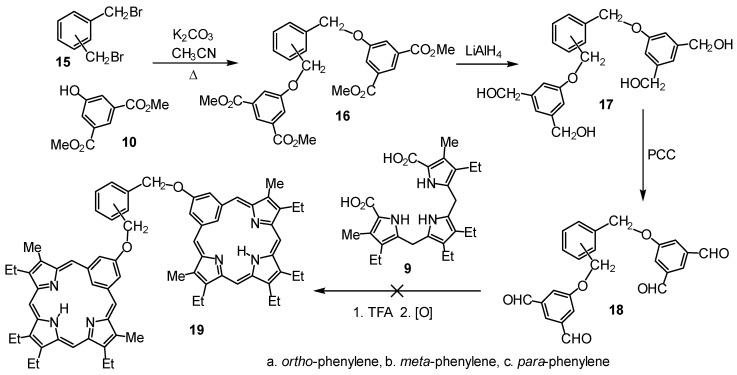
Attempted synthesis of benziporphyrin dimers.

The possibility of using this strategy to prepare linked benziporphyrins was also investigated. Alkylation of dimethyl 5-hydroxybenzene-1,3-dicarboxylate **10** with *o*-, *m*-, or *p*-dibromoxylenes **15a–c** and potassium carbonate in refluxing acetonitrile gave a series of linked tetraesters **16a–c** in a 67–99% yield. Reduction with lithium aluminum hydride afforded the related tetraalcohols **17a–c**, and subsequent oxidation with PCC yielded tetraaldehydes **18a–c** (Scheme 5). Unfortunately, attempts to prepare benziporphyrin dimers **19a–c** by reacting tetraaldehydes **18a–c** with two equivalents of tripyrrane **9** only afforded trace amounts of porphyrinoid products. The reactions were attempted using DDQ or FeCl_3_ as the oxidant, and at different concentrations of reactants, but none of the conditions investigated produced useful quantities of product, and substantial decomposition was observed. Although this route proved not to be viable, these new tetraaldehydes have potentially valuable architectures that may allow the synthesis of other tethered systems.

## 3. Experimental

Melting points are uncorrected. NMR spectra are recorded using a 400 or 500 MHz NMR spectrometer and are run at 302 K unless otherwise indicated. ^1^H NMR values are reported as chemical shifts δ, relative integral, multiplicity (s, singlet; d, doublet; t, triplet; q, quartet; m, multiplet; br, broad peak) and coupling constant (*J*). Chemical shifts are reported in parts per million (ppm) relative to CDCl_3_ (^1^H residual CHCl_3_ singlet δ 7.26 ppm, ^13^C CDCl_3_ triplet δ 77.23 ppm) or DMSO-*d_6_* (^1^H residual DMSO-*d_5_* pentet δ 2.49 ppm, ^13^C DMSO-*d_6_* septet δ 39.7 ppm), and coupling constants are taken directly from the spectra. NMR assignments are made with the aid of ^1^H-^1^H COSY, HSQC, DEPT-135, and nOe difference proton NMR spectroscopy. Two-dimensional (2D)-NMR experiments are performed using standard software. Mass spectral data are acquired using positive-mode electrospray ionization (ESI^+^) and a high-resolution time-of-flight mass spectrometer.

**Dimethyl 5-methoxy-1,3-benzenedicarboxylate** (**11a**). Dimethyl 5-hydroxyisophthalate (1.860 g, 8.9 mmol), methyl iodide (2.301 g, 16.2 mmol), and potassium carbonate (1.342 g, 10.4 mmol) were dissolved in acetonitrile (30 mL) and refluxed overnight. The solution was cooled, and the solvent was removed on a rotary evaporator. The residue was dissolved in ethyl acetate (50 mL) and then washed with water (2 × 30 mL) and saturated sodium bicarbonate (2 × 30 mL). The organic layer was dried over sodium sulfate and filtered, and the solvent was evaporated under reduced pressure to yield 5-methoxyisophthalic acid dimethyl ester (1.989 g, 8,88 mmol, quantitative) as a white solid, mp 111–112 °C (lit. mp [26] 110–112 °C). ^1^H NMR (500 MHz, CDCl_3_): δ 8.29 (t, 1H, ^4^*J*_HH_ = 1.4 Hz, 2-H), 7.75 (d, 2H, ^4^*J*_HH_ = 1.4 Hz, 4,6-H), 3.94 (s, 6H, 2 × CO_2_CH_3_), 3.89 (s, 3H, OCH_3_). ^13^C NMR (125 MHz, CDCl_3_): δ 166.3 (C=O), 159.9 (5-C), 128.3 (1,3-C), 123.1 (2-CH), 119.5 (4,6-CH), 55.9 (OMe), 52.5 (2 × ester OMe).

**5-Methoxy-1,3-bis(hydroxymethyl)benzene** (**12a**). Lithium aluminum hydride (1.111 g, 29.3 mmol) was added to a solution of **11a** (1.918 g, 8.56 mmol) in dry THF (100 mL), and the resulting mixture was stirred at room temperature overnight. Dilute hydrochloric acid (80 mL) was added dropwise to the solution, and the contents of the reaction flask were stirred for an additional 30 min. Ethyl acetate (200 mL) was added, and the aqueous layer was drawn off. The organic layer was washed with water (2 × 100 mL) and saturated sodium bicarbonate solution (2 × 100 mL). The ethyl acetate layer was dried over sodium sulfate and filtered, and the solvent was removed under reduced pressure to yield **12a** (1.439 g, 8.56 mmol, quantitative) as a white solid, mp 66–68 °C (lit. mp [27] 66–67 °C). ^1^H NMR (500 MHz, CDCl_3_): δ 6.94 (s, 1H, 2-H), 6.85 (br s, 2H, 4,6-H), 4.67 (s, 4H, 2 × CH_2_), 3.82 (s, 3H, OCH_3_), 1.76 (br s, 2H, 2 × OH). ^13^C NMR (125 MHz, CDCl_3_): δ 160.4 (5-C), 143.3 (1,3-C). 117.7 (2-CH), 111.8 (4,6-CH), 65.4 (2 × CH_2_OH), 55.5 (OCH_3_).

**5-Methoxybenzene-1,3-dicarbaldehyde** (**8a**). Dialcohol **12a** (1.515 g, 9.02 mmol) was dissolved in dichloromethane (108 mL) and THF (72 mL). Pyridinium chlorochromate (5.979 g, 27.7 mmol) and silica gel (3.720 g, 61.9 mmol) were added to the solution, and the mixture was stirred at room temperature for 1 h. The contents of the reaction flask were immediately chromatographed twice on silica gel, eluting with dichloromethane. The desired column fractions were evaporated under reduced pressure to afford the dialdehyde (1.185 g, 7.22 mmol, 80%) as a white solid, mp 109–110 °C (lit. mp [28] 108–109 °C). ^1^H NMR (500 MHz, CDCl_3_): δ 10.05 (s, 2H, 2 × CHO), 7.96 (t, 1H, ^4^*J*_HH_ = 1.4 Hz, 2-H), 7.65 (d, 2H, ^4^*J*_HH_ = 1.4 Hz, 4,6-H), 3.93 (s, 3H, OCH_3_). ^13^C NMR (125 MHz, CDCl_3_): δ 191.0 (2 × CHO), 161.1 (5-C), 138.6 (1,3-C), 124.4 (2-CH), 119.6 (4,6-CH), 56.2 (OCH_3_).

**Dimethyl 5-ethoxy-1,3-benzenedicarboxylate** (**11b**). Dimethyl 5-hydroxy-isophthalate (1.680 g, 8.0 mmol), ethyl iodide (1.905 g, 12.2 mmol), and potassium carbonate (1.344 g, 9.7 mmol) were dissolved in acetonitrile (30 mL) and refluxed overnight. The solution was cooled, and the solvent was removed using a rotary evaporator. The resulting solid was dissolved in ethyl acetate (50 mL) and washed with water (2 × 30 mL) and saturated sodium bicarbonate (2 × 30 mL). The organic layer was dried over sodium sulfate and filtered, and the solvent was removed under reduced pressure to yield 5-ethoxyisophthalic acid dimethyl ester (1.754 g, 7,57 mmol, 92%) as a white solid, mp 101–102 °C (lit. mp [29] 102 °C). ^1^H NMR (500 MHz, CDCl_3_): δ 8.25 (t, 1H, ^4^*J*_HH_ = 1.4 Hz, 2-H), 7.73 (d, 2H, ^4^*J*_HH_ = 1.4 Hz, 4,6-H), 4.11 (q, 2H, ^3^*J*_HH_ = 7.0 Hz, OC*H*_2_CH_3_), 3.93 (s, 6H, 2 × OCH_3_), 1.44 (t, 3H, ^3^*J*_HH_ = 7.0 Hz, OCH_2_C*H*_3_). ^13^C NMR (125 MHz, CDCl_3_): δ 166.4 (C=O), 159.2 (5-C), 131.9 (1,3-C), 123.0 (2-CH), 120.0 (4,6-CH), 64.3 (OCH_2_), 52.5 (2 × OMe), 14.8 (OCH_2_*C*H_3_).

**5-Ethoxy-1,3-bis(hydroxymethyl)benzene** (**12b**). Lithium aluminum hydride (1.018 g, 26.8 mmol) was added to a solution of **11b** (2.018 g, 8.48 mmol) in dry THF (100 mL). The mixture was stirred at room temperature overnight, dilute hydrochloric acid (80 mL) was added dropwise to the solution, and the contents of the reaction flask were stirred for an additional 30 min. Ethyl acetate (200 mL) was added, and the aqueous layer was drawn off. The organic layer was washed with water (2 × 100 mL) and a saturated sodium bicarbonate solution (2 × 100 mL). The organic solution was dried over sodium sulfate and filtered, and the solvent was removed under reduced pressure to yield the dialcohol (1.534 g, 8.43 mmol, 99%) as a white solid, mp 123–124 °C (lit. mp [29] 124–125 °C). ^1^H NMR (500 MHz, CDCl_3_): δ 6.88 (br s, 1H, 2-H), 6.79 (br s, 2H, 4,6-H), 4.59 (s, 4H, 2 × C*H*_2_OH), 4.02 (q, 2H, ^3^*J*_HH_ = 7.0 Hz, OC*H*_2_CH_3_), 2.53 (br s, 2H, 2 × OH), 1.39 (t, 3H, ^3^*J*_HH_ = 7.0 Hz, OCH_2_C*H*_3_). ^13^C NMR (125 MHz, CDCl_3_): δ 159.6 (5-C), 143.0 (1,3-C). 117.6 (2-CH), 112.3 (4,6-CH), 65.2 (2 × CH_2_OH), 63.7 (O*C*H_2_CH_3_), 15.0 (CH_2_*C*H_3_).

**5-Ethoxybenzene-1,3-dicarbaldehyde** (**8b**). Dialcohol **12b** (0.191 g, 1.05 mmol) was dissolved in dichloromethane (12 mL) and THF (8 mL). Pyridinium chlorochromate (0.656 g, 3.0 mmol) and silica gel (0.411 g, 6.8 mmol) were added to the solution, and the mixture was stirred at room temperature for 1 h. The contents of the reaction flask were immediately chromatographed twice on silica gel, eluting with dichloromethane. The desired column fractions were evaporated under reduced pressure to give the dialdehyde (0.175 g, 0.98 mmol, 94%) as a white solid, mp 207–208 °C (lit. mp [29] 208–209 °C). ^1^H NMR (500 MHz, CDCl_3_): δ 10.05 (s, 2H, 2 × CHO), 7.94 (s, 1H, ^4^*J*_HH_ = 1.4 Hz, 2-H), 7.64 (s, 2H, ^4^*J*_HH_ = 1.4 Hz, 4,6-H), 4.16 (q, 4H, ^3^*J*_HH_ = 7.0 Hz, OCH_2_), 1.47 (t, 3H, ^3^*J*_HH_ = 7.0 Hz, CH_2_C*H*_3_). ^13^C NMR (125 MHz, CDCl_3_): δ 191.1 (2 × CHO), 160.4 (5-C), 138.6 (1,3-C), 124.2 (2-CH), 120.1 (4,6-CH), 64.6 (OCH_2_), 14.8 (CH_2_*C*H_3_).

**5-Hydroxybenzene-1,3-dicarbaldehyde** (**14**). Lithium aluminum hydride (6.076 g, 160 mmol) was dissolved in dry THF (150 mL) in a 3-neck flask fitted with an addition funnel, stopper, and condenser. A solution of dimethyl 5-hydroxyisophthalate (10.501 g, 50.0 mmol) in dry THF (175 mL) was added dropwise to the solution, and the mixture was refluxed for 2 h and subsequently stirred at room temperature overnight. A mixture of ethanol (5 mL), ethyl acetate (10 mL), and brine (50 mL) was added dropwise. After the addition was complete, the contents of the flask were filtered, and the solid was washed with ethanol. The filtrate was evaporated under reduced pressure to yield 3,5-bis(hydroxymethyl)phenol (**13**, 7.005 g, 45.5 mmol, 91%) as a white solid, mp 174–175 °C (lit. mp [30] 175–176 °C). The crude solid (3.90 g, 25.3 mmol) and potassium dichromate (14.94 g, 50.8 mmol) were dissolved in DMSO (166 mL) and stirred at 100 °C for 4 h. The mixture was cooled, poured into a beaker of water (750 mL), and extracted with diethyl ether (5 × 200 mL). The aqueous layer was discarded, and the organic phase was washed with water (150 mL), dried over sodium sulfate, and filtered, and the solvent was removed on a rotary evaporator to give the dialdehyde (2.512 g, 16.7 mmol, 66%) as a light tan solid, mp 146–147 °C (lit. mp [30] 146–147.5 °C). ^1^H NMR (500 MHz, CDCl_3_): δ 10.04 (s, 2H, 2 × CHO), 7.95 (t, 1H, ^4^*J*_HH_ = 1.4 Hz, 2-H), 7.63 (d, 2H, ^4^*J*_HH_ = 1.4 Hz, 4,6-H), 6.23 (1H, br s, OH). ^13^C NMR (125 MHz, CDCl_3_): δ 191.3 (2 × CHO), 157.5 (5-C), 138.8 (1,3-C), 124.5 (2-CH), 121.0 (4,6-CH).

**5-Methoxycarbonylmethoxybenzene-1,3-dicarbaldehyde** (**8c**). Dialdehyde **12** (301 mg, 2.00 mmol), potassium carbonate (357 mg, 2.6 mmol), and methyl bromoacetate (0.3 mL, 3.2 mmol) in acetone (25 mL) were stirred at room temperature overnight. The solvent was removed under reduced pressure, and the residue was dissolved in ethyl acetate (20 mL). The solution was washed with water and saturated sodium bicarbonate solution. The organic layer was dried over sodium sulfate and filtered, and the solvent was removed under reduced pressure to yield **8c** (275 mg, 1.24 mmol, 62%) as a white solid, mp 97–99 °C. ^1^H NMR (500 MHz, CDCl_3_): δ 10.05 (s, 2H, 2 × CHO), 8.01 (t, 1H, ^4^*J*_HH_ = 1.3 Hz, 2-H), 7.66 (d, 2H, ^4^*J*_HH_ = 1.3 Hz, 4,6-H), 4.77 (s, 2H, CH_2_O), 3.83 (s, 3H, OCH_3_). ^13^C NMR (100 MHz, CDCl_3_): δ 190.6 (CHO), 168.4 (OCH_2_*C*O_2_CH_3_), 159.0 (5-C), 138.4 (1,3-C), 124.9 (2-CH), 119.9 (4,6-CH), 65.3 (O*C*H_2_CO_2_CH_3_), 52.4 (OCH_2_CO_2_*C*H_3_). HRMS (ESI-TOF) *m*/*z*: [M + H]^+^ Calcd for C_11_H_11_O_5_^+^ 223.0601; Found 223.0601.

**9,13,14,18-Tetraethyl-3-methoxy-8,19-dimethylbenziporphyrin** (**7a**). In a pear-shaped flask, tripyrrane dicarboxylic acid **9** (99 mg, 0.22 mmol) was dissolved in TFA (1 mL) and stirred under nitrogen for 2 min. Dichloromethane (100 mL) was added, followed by 5-methoxybenzene-1,3-dicarbaldehyde (42 mg, 0.25 mmol). The flask was subsequently covered in aluminum foil to protect the reaction from ambient light, and the solution was stirred overnight under nitrogen. DDQ (81 mg, 0.357 mmol) was added, and the mixture was stirred for 1 h. The resulting solution was washed with water and saturated sodium bicarbonate solution (the aqueous layers were back-extracted with chloroform to ensure all of the product was collected). The combined organic layers were evaporated under reduced pressure, and the residue was purified on a grade 3 basic alumina column, eluting with dichloromethane. The product was collected as a dark-blue band. Recrystallization from chloroform-methanol afforded the benziporphyrin (37 mg, 0.075 mmol, 34%) as dark-purple crystals, mp > 300 °C. UV-vis (1% Et_3_N-CH_2_Cl_2_): λ_max_ (log ε): 308 (4.73), 382 (4.84), 580 (sh, 3.46), 630 (3.62), 672 (3.65), 733 nm (3.44). UV-vis (1% TFA-CH_2_Cl_2_): λ_max_ (log ε): 315 (4.63), 399 (4.78), 504 (3.91), 573 (3.36), 692 (sh, 3.53), 745 (3.07), 838 nm (sh, 3.61). ^1^H NMR (500 MHz, CDCl_3_): δ 9.21 (br s, 1H, NH), 7.86 (s, 1H, 22-H), 7.49 (d, 2H, ^4^*J*_HH_ = 1.2 Hz, 2,4-H), 7.14 (s, 2H, 6,21-H), 6.48 (s, 2H, 11,16-H), 4.04 (s, 3H, OCH_3_), 2.82 (q, 4H, ^3^*J*_HH_ = 7.6 Hz, 9,18-C*H*_2_CH_3_), 2.73 (q, 4H, ^3^*J*_HH_ = 7.6 Hz, 13,14-C*H*_2_CH_3_), 2.39 (s, 6H, 8,19-CH_3_), 1.33 (t, 6H, ^3^*J*_HH_ = 7.6 Hz, 13,14-CH_2_C*H*_3_), 1.25 (t, 6H, ^3^*J*_HH_ = 7.6 Hz, 9,18-CH_2_C*H*_3_). ^1^H NMR (500 MHz, TFA-CDCl_3_): 9.86 (br s, 1H, NH), 7.92 (s, 2H, 6,21-H), 7.75 (s, 2H, 2,4-H), 6.92 (s, 2H, 11,16-H), 5.06 (s, 1H, 22-H), 4.03 (s, 3H, OCH_3_), 2.92–2.87 (m, 8H, 9,13,14,18-CH_2_), 2.61 (s, 6H, 8,19-CH_3_), 1.33 (t, 6H, ^3^*J*_HH_ = 7.6 Hz), 1.30 (t, 6H, ^3^*J*_HH_ = 7.6 Hz) (4 × CH_2_C*H*_3_). ^13^C NMR (125 MHz, CDCl_3_): δ 169.3, 159.6, 157.4, 148.1, 141.3, 141.2, 140.5, 135.2, 122.6 (6,21-CH), 122.1 (2,4-CH), 118.7 (22-CH), 93.1 (11,16-CH), 55.7 (OCH_3_), 18.5 (13,14-*C*H_2_CH_3_), 18.1 (9,18-*C*H_2_CH_3_), 16.1 (13,14-CH_2_*C*H_3_), 15.3 (9,18-CH_2_*C*H_3_), 10.4 (8,19-CH_3_). ^13^C NMR (125 MHz, TFA-CDCl_3_): δ 163.1, 162.8, 154.7, 148.0, 146.6, 143.8, 141.6, 133.2, 128.4 (6,21-CH), 124.7 (2,4-CH), 101.7 (22-CH), 94.2 (11,16-CH), 56.3 (OCH_3_), 18.4 (13,14-*C*H_2_CH_3_), 18.1 (9,18-*C*H_2_CH_3_), 15.2 (13,14-CH_2_*C*H_3_), 14.3 (9,18-CH_2_*C*H_3_), 10.7 (8,19-CH_3_). HRMS (ESI-TOF) *m*/*z*: [M + H]^+^ Calcd for C_33_H_38_N_3_O^+^ 492.3009; Found 492.3011.

**[9,13,14,18-Tetraethyl-3-methoxy-8,19-dimethylbenziporphyrinato]palladium(II)** (**7aPd**). Methoxybenziporphyrin **7a** (17 mg, 0.0346 mmol) and palladium(II) acetate (20 mg, 0.089 mmol) in acetonitrile (15 mL) were refluxed for 30 min. The solution was cooled to room temperature and then diluted with dichloromethane (35 mL). The solution was washed with water, and the solvent was removed using a rotary evaporator. The residue was chromatographed on a grade 3 basic alumina column, eluting with dichloromethane. A reddish-purple fraction was collected, and the solvent was removed under reduced pressure. The residue was recrystallized from chloroform-methanol to give the palladium complex (15 mg, 0.025 mmol, 72%) as a dark-purple solid, mp > 300 °C. UV-vis (CH_2_Cl_2_): λ_max_ (log ε): 308 (4.61), 391 (sh, 4.69), 407 (4.75), 474 (sh, 3.48), 507 (sh, 3.67), 543 (3.82), 572 (3.84), 666 (3.31), 728 (3.55), 807 (3.50). ^1^H NMR (400 MHz, CDCl_3_): δ 7.68 (2H, s, 2,4-H), 7.57 (2H, s, 6,21-H), 7.25 (2H, s, 11,16-H), 4.10 (3H, s, OCH_3_), 3.01–2.92 (8H, overlapping quartets, 4 × C*H*_2_CH_3_), 2.59 (6H, s, 8,19-CH_3_), 1.43 (6H, t, ^3^*J*_HH_ = 7.6 Hz, 13,14-CH_2_C*H*_3_), 1.37 (6H, t, ^3^*J*_HH_ = 7.6 Hz, 9,18-CH_2_C*H*_3_). ^13^C NMR (100 MHz, CDCl_3_): δ 157.4, 157.2, 153.7, 145.3, 144.0, 141.1, 138.5, 133.4, 131.6, 126.8 (2,4-CH), 126.6 (6,16-CH), 95.5 (11,21-CH), 55.8 (OCH_3_), 18.9 (13,14-*C*H_2_CH_3_), 18.6 (9,18-*C*H_2_CH_3_), 16.6 (13,14-CH_2_*C*H_3_), 15.3 (9,18-CH_2_*C*H_3_). HRMS (ESI-TOF) *m*/*z*: M^+^ Calcd for C_33_H_36_N_3_OPd^+^ 506.1888; Found 596.1869.

**[9,13,14,18-Tetraethyl-3-methoxy-8,19-dimethylbenziporphyrinato]nickel(II)** (**7aNi**). Methoxybenziporphyrin **7a** (19 mg, 0.0387 mmol) and nickel(II) acetate (40 mg, 0.16 mmol) in DMF (20 mL) were refluxed for 30 min. The solution was cooled to room temperature and diluted with chloroform (25 mL). The solution was washed with water, and the solvent was evaporated under reduced pressure. The residue was chromatographed on a grade 3 basic alumina column, eluting with chloroform. A dark-green band was collected, and the solvent was removed under reduced pressure. The residue was recrystallized from chloroform-methanol to give the nickel complex (16 mg, 0.029 mmol, 75%) as a dark-green solid, mp > 300 °C. UV-vis (CH_2_Cl_2_): λ_max_ (log ε): 313 (4.32), 353 (4.27), 405 (4.39), 493 (sh, 3.43), 527 (3.38), 660 (3.51). ^1^H NMR (500 MHz, CDCl_3_) δ 7.63 (s, 2H, 2,4-H), 7.35 (s, 2H, 6,21-H), 7.08 (s, 2H, 11,16-H), 4.05 (s, 3H, OCH_3_), 2.91 (q, 4H, ^3^*J*_HH_ = 7.6 Hz, 13,14-C*H*_2_CH_3_), 2.84 (q, 4H, ^3^*J*_HH_ = 7.6 Hz, 9,18-C*H*_2_CH_3_), 2.44 (s, 6H, 8,19-CH_3_), 1.37 (t, 6H, ^3^*J*_HH_ = 7.6 Hz, 13,14-CH_2_C*H*_3_), 1.30 (t, 6H, ^3^*J*_HH_ = 7.6 Hz, 9,18-CH_2_C*H*_3_). ^13^C NMR (125 MHz, CDCl_3_): δ 159.5, 157,8, 153.5, 146.7, 144.7, 141.4, 139.9, 136.7, 130.0, 125.8 (2,4-CH), 123.2 (6,21-CH), 95.3 (11,16-CH), 55.7 (OCH_3_), 18.50, 18.48 (13,14-*C*H_2_CH_3_), 16.5 (13,14-CH_2_*C*H_3_), 15.3 (9,18-CH_2_*C*H_3_), 10.4 (8,19-CH_3_). HRMS (ESI-TOF) *m*/*z*: M^+^ Calcd for C_33_H_36_N_3_NiO^+^ 548.2206; Found 548.2205.

**3-Ethoxy-9,13,14,18-tetraethyl-8,19-dimethylbenziporphyrin** (**7b**). In a pear-shaped flask, tripyrrane dicarboxylic acid **9** (106 mg, 0.234 mmol) was dissolved in TFA (1 mL) and stirred under nitrogen for 2 min. Dichloromethane (100 mL) was added, followed by dialdehyde **8b** (42 g, 0.236 mol), and the mixture was stirred in the dark overnight under nitrogen. DDQ (73 mg, 0.321 mmol) was added, and the mixture was stirred for 1 h. The resulting solution was washed with water and saturated sodium bicarbonate, back extracting the aqueous layers with chloroform at each stage to ensure that all of the product was collected. The combined organic layers were evaporated under reduced pressure, and the residue was purified on a grade 3 basic alumina column, eluting with dichloromethane. The product was collected as a dark-blue band. Recrystallization from chloroform-methanol gave benziporphyrin **7b** (52 mg, 0.103 mmol, 44%) as dark-purple crystals, mp > 300 °C. UV-vis (1% Et_3_N-CH_2_Cl_2_): λ_max_ (log ε): 308 (4.77), 382 (4.89), 581 (sh, 3.37), 626 (3.59), 673 (3.68), 733 nm (3.48). UV-vis (5 equiv. TFA-CH_2_Cl_2_): λ_max_ (log ε): 304 (4.65), 391 (4.87), 479 (3,74), 524 (3.71), 566 (3.73), 776 (3.90), 858 (4.15). UV-vis (1% TFA-CH_2_Cl_2_): λ_max_ (log ε): 315 (4.65), 400 (4.82), 510 (3.92), 573 (3.36), 711 (sh, 3.57), 760 (3.70), 842 nm (sh, 3.60). ^1^H NMR (500 MHz, CDCl_3_): δ 9.16 (br s, 1H, NH), 7.80 (s, 1H, 22-H), 7.49 (br d, 2H, ^4^*J*_HH_ = ca. 1 Hz, 2,4-H), 7.14 (s, 1H, 6,21-H), 6.48 (s, 2H, 11,16-H), 4.29 (q, 2H, ^3^*J*_HH_ = 6.9 Hz, OCH_2_), 2.82 (q, 4H, ^3^*J*_HH_ = 7.6 Hz, 13,14-CH_2_), 2.74 (q, 4H, ^3^*J*_HH_ = 7.6 Hz, 9,18-CH_2_), 2.39 (s, 6H, 8,19-CH_3_), 1.53 (t, 3H, ^3^*J*_HH_ = 6.9 Hz, OCH_2_C*H*_3_), 1.34 (t, 6H, ^3^*J*_HH_ = 7.6 Hz, 13,14-CH_2_C*H*_3_), 1.26 (t, 6H, ^3^*J*_HH_ = 7.6 Hz, 9,18-CH_2_C*H*_3_). ^1^H NMR (500 MHz, TFA-CDCl_3_): δ 9.42 (br s, 2H, 2 × NH), 8.03 (s, 2H, 6,21-H), 7.83 (br d, 2H, 2,4-H), 7.04 (s, 2H, 10,15-H), 4.77 (s, 1H, 22-H), 4.30 (q, 2H, ^3^*J*_HH_ = 7.0 Hz, OCH_2_), 2.98–2.92 (m, 8H, 9,13,14,18-CH_2_), 2.66 (s, 6H, 8,19-CH_3_), 1.54 (t, 3H, ^3^*J*_HH_ = 7.0 Hz, OCH_2_C*H*_3_), 1.37 (t, 6H, ^3^*J*_HH_ = 7.7 Hz), 1.32 (t, 6H, ^3^*J*_HH_ = 7.7 Hz) (4 × CH_2_C*H*_3_). ^13^C NMR (125 MHz, CDCl_3_): δ 169.2, 158.9, 157.3, 148.1, 141.3, 141.1, 140.5, 135.2, 122.9 (2,4-CH), 122.7 (6,21-CH), 118.5 (22-CH), 93.1 (11,16-CH), 64.0 (OCH_2_), 18.5 (13,14-CH_2_), 18.1 (9,18-CH_2_), 16.1 (13,14-CH_2_*C*H_3_), 15.3 (9,18-CH_2_*C*H_3_), 15.2 (OCH_2_*C*H_3_), 10.4 (8,19-CH_3_). ^13^C NMR (125 MHz, TFA-CDCl_3_): δ 162.7, 162.3, 155.0, 147.3, 146.3, 143.8, 141.3, 133.2, 128.0 (6,21-CH), 124.8 (2,4-CH), 101.9 (22-CH), 94.0 (11,16-CH), 64.8 (OCH_2_), 18.4 (13,14-*C*H_2_), 18.1 (9,18-CH_2_), 15.2 (13,14-CH_2_*C*H_3_), 14.8 (9,18-CH_2_*C*H_3_), 14.3 (OCH_2_*C*H_3_), 10.6 (8,19-CH_3_). HRMS (ESI-TOF) *m*/*z*: [M + H]^+^ Calcd for C_34_H_40_N_3_O^+^ 506.3166; Found 506.3167.

**[3-Ethoxy-9,13,14,18-tetraethyl-8,19-dimethylbenziporphyrinato]palladium(II)** (**7bPd**)**.** Ethoxybenziporphyrin **7b** (17 mg, 0.0336 mmol) and palladium(II) acetate (17 mg, 0.076 mmol) in acetonitrile (15 mL) were refluxed for 30 min. The solution was cooled to room temperature and then diluted with dichloromethane (35 mL). The solution was washed with water, and the solvent was removed on a rotary evaporator. The residue was chromatographed on a grade 3 basic alumina column, eluting with dichloromethane. A reddish-purple fraction was collected, and the solvent was removed under reduced pressure. The residue was recrystallized from chloroform-methanol to give the palladium complex (15 mg, 0.025 mmol, 73%) as a dark-purple solid, mp > 300 °C. UV-vis (CH_2_Cl_2_): λ_max_ (log ε): 308 (4.61), 391 (sh, 4.67), 408 (4.75), 474 (sh, 3.48), 507 (sh, 3.69), 542 (3.85), 573 (3.86), 666 (3.27), 731 (3.53), 806 (3.46). ^1^H NMR (500 MHz, CDCl_3_): δ 7.70 (s, 2H, 2,4-H), 7.57 (s, 1H, 6,21-H), 7.27 (s, 2H, 11,16-H), 4.37 (q, 2H, ^3^*J*_HH_ = 7.0 Hz, OCH_2_), 3.01–2.93 (m, 8H, 9,13,14,18-CH_2_), 2.59 (s, 6H, 8,19-CH_3_), 1.57 (t, 3H, ^3^*J*_HH_ = 7.0 Hz, OCH_2_C*H*_3_), 1.43 (t, 6H, ^3^*J*_HH_ = 7.6 Hz, 13,14-CH_2_C*H*_3_), 1.38 (t, 6H, ^3^*J*_HH_ = 7.6 Hz, 9,18-CH_2_C*H*_3_). ^13^C NMR (125 MHz, CDCl_3_): δ 157.1, 156.1, 153.7, 145.2, 144.0, 141.1, 138.5, 133.3, 131.5, 127.7 (2,4-CH), 126.6 (6,21-CH), 95.5 (11,16-CH), 64.0 (OCH_2_), 18.9, 18.6 (9,13,14,18-CH_2_), 16.6 (13,14-CH_2_*C*H_3_), 15.3 (OCH_2_*C*H_3_ and 9,18-CH_2_*C*H_3_), 10.5 (8,19-CH_3_). HRMS (ESI-TOF) *m*/*z*: M^+^ Calcd for C_34_H_38_N_3_OPd^+^ 610.2044; Found 610.2037.

**[3-Ethoxy-9,13,14,18-tetraethyl-8,19-dimethylbenziporphyrinato]nickel(II)** (**7bNi**). Ethoxybenziporphyrin **7b** (20 mg, 0.0396 mmol) and nickel(II) acetate (32 mg, 0.129 mmol) in DMF (20 mL) were refluxed for 30 min. The mixture was cooled to room temperature and diluted with chloroform (20 mL). The solution was washed with water, and the solvent was evaporated under reduced pressure. The residue was chromatographed on a grade 3 basic alumina column, eluting with chloroform. A dark-green band was collected, and the solvent was removed under reduced pressure. The residue was recrystallized from chloroform-methanol to give the nickel complex (12 mg, 0.0213 mmol, 54%) as a dark-green solid, mp > 300 °C. UV-vis (CH_2_Cl_2_): λ_max_ (log ε): 313 (4.48), 355 (4.43), 406 (4.56), 493 (sh, 3.58), 528 (3.52), 669 (3.65). ^1^H NMR (500 MHz, CDCl_3_): δ 7.63 (s, 2H, 2,4-H), 7.34 (s, 1H, 6,21-H), 7.09 (s, 2H, 11,16-H), 4.31 (q, 2H, ^3^*J*_HH_ = 7.0 Hz, OCH_2_), 2.91 (q, 4H, ^3^*J*_HH_ = 7.6 Hz, 12,13-CH_2_), 2.84 (q, 4H, ^3^*J*_HH_ = 7.6 Hz, 9,18-CH_2_), 2.44 (s, 6H, 8,19-CH_3_), 1.53 (t, 3H, ^3^*J*_HH_ = 7.0 Hz, OCH_2_C*H*_3_), 1.37 (t, 6H, ^3^*J*_HH_ = 7.6 Hz, 13,14-CH_2_C*H*_3_), 1.30 (t, 6H, ^3^*J*_HH_ = 7.6 Hz, 9,18-CH_2_C*H*_3_). ^13^C NMR (125 MHz, CDCl_3_): δ 159.5, 157.1, 153.5, 146.7, 144.7, 141.4, 139.9, 136.7, 130.0, 126.7 (2,4-CH), 123.2 (6,21-CH), 95.3 (11,16-CH), 64.0 (OCH_2_), 18.51, 18.49 (9,13,14,18-CH_2_), 16.5 (13,14-CH_2_*C*H_3_), 15.29 (9,18-CH_2_*C*H_3_), 15.27 (OCH_2_*C*H_3_), 10.4 (8,19-CH_3_). HRMS (ESI-TOF) *m*/*z*: M^+^ Calcd for C_34_H_38_N_3_NiO^+^ 562.2363; Found 562.2352.

**9,13,14,18-Tetraethyl-3-(methoxycarbonylmethoxy)-8,19-dimethylbenziporphyrin** (**7c**). In a pear-shaped flask, tripyrrane dicarboxylic acid **9** (202 mg, 0.446 mmol) was dissolved in TFA (2 mL) and stirred under nitrogen for 2 min. Dichloromethane (200 mL) was added, followed by dialdehyde **8c** (104 mg, 0.468 mmol). The flask was covered in aluminum foil to protect the reaction from ambient light, and the solution was stirred overnight under nitrogen. DDQ (154 mg, 0.678 mmol) was added, and the mixture was stirred for 1 h. The resulting solution was washed with water and saturated sodium bicarbonate, back-extracting the aqueous layers with chloroform at each stage to ensure that all of the product was collected. The organic layers were evaporated under reduced pressure, and the residue was purified on a grade 3 basic alumina column, eluting with dichloromethane. The product was collected as a dark-blue band. Recrystallization from chloroform-methanol afforded benziporphyrin **7c** (100 mg, 0.182 mmol, 41%) as dark-purple crystals, mp > 300 °C. UV-vis (1% Et_3_N-CH_2_Cl_2_): λ_max_ (log ε): 308 (5.60), 380 (5.72), 541 (sh, 4.39), 583 (sh, 4.59), 629 (sh, 4.62), 677 (sh, 4.46), 742 (sh, 4.12). UV-vis (1% TFA-CH_2_Cl_2_): λ_max_ (log ε): 313 (5.56), 398 (5.71), 494 (sh, 4.89), 535 (sh, 4.66), 575 (sh, 4.55), 723 (sh, 4.69), 836 (sh, 4.46). ^1^H NMR (500 MHz, CDCl_3_): δ 9.35 (br s, 1H, NH), 8.04 (br t, 1H, 22-H), 7.48 (d, 2H, ^4^*J*_HH_ = 1.2 Hz, 2,4-H), 7.07 (s, 2H, 6,21-H), 6.45 (s, 2H, 11,16-H), 4.86 (s, 2H, CH_2_CO_2_Me), 3.85 (s, 3H, OCH_3_), 2.81 (q, 4H, ^3^*J*_HH_ = 7.6 Hz, 12,13-C*H*_2_CH_3_), 2.72 (q, 4H, ^3^*J*_HH_ = 7.6 Hz, 9,18-C*H*_2_CH_3_), 2.37 (s, 6H, 8,19-CH_3_), 1.33 (t, 6H, ^3^*J*_HH_ = 7.6 Hz, 13,14-CH_2_C*H*_3_), 1.25 (t, 6H, ^3^*J*_HH_ = 7.6 Hz, 9,18-CH_2_C*H*_3_). ^1^H NMR (400 MHz, TFA-CDCl_3_): δ 7.71 (s, 2H), 7.64 (s, 2H, 6,21-H), 6.70 (s, 2H, 11,16-H), 4.83 (s, 2H, CH_2_CO_2_Me), 3.85 (s, 3H, OCH_3_), 2.82 (q, 8H, ^3^*J*_HH_ = 7.6 Hz, 4 × C*H*_2_CH_3_), 2.55 (s, 6H, 8,19-CH_3_), 1.32–1.26 (overlapping triplets, 12H, 4 × CH_2_C*H*_3_). ^13^C NMR (125 MHz, CDCl_3_): δ 169.7, 169.5, 157.9, 157.6, 148.3, 141.34, 141.26, 140.6, 135.4, 122.6 (2,4-CH), 122.3 (6,21-CH), 119.6 (22-CH), 93.1 (11,16-CH), 66.0 (OCH_2_), 52.6 (OCH_3_), 18.4 (13,14-*C*H_2_CH_3_), 18.1 (9,18-*C*H_2_CH_3_), 16.1 (13,14-CH_2_*C*H_3_), 15.3 (9,18-CH_2_*C*H_3_), 10.4 (8,19-CH_3_). ^13^C NMR (100 MHz, TFA-CDCl_3_): δ 169.3, 163.2, 161.1, 155.3, 147.4, 146.4, 141.1, 133.5, 127.6 (2,4-CH), 124.3 (6,21-CH), 102.6 (22-CH), 94.1 (11,16-CH), 65.9 (OCH_2_), 52.9 (OCH_3_), 18.4 (13,14-*C*H_2_CH_3_), 18.1 (9,18-*C*H_2_CH_3_), 15.2 (13,14-CH_2_*C*H_3_), 14.3 (9,18-CH_2_*C*H_3_), 10.6 (8,19-CH_3_). HRMS (ESI-TOF) *m*/*z*: [M + H]^+^ Calcd for C_35_H_40_N_3_O_3_^+^ 550.3064; Found 550.3067.

**[9,13,14,18-Tetraethyl-3-(methoxycarbonylmethoxy)-8,19-dimethylbenziporphyrinato]palladium(II)** (**7cPd**). Benziporphyrin **7c** (20 mg, 0.0364 mmol) and palladium(II) acetate (22 mg, 0.098 mmol) in acetonitrile (15 mL) were refluxed for 30 min. The solution was cooled to room temperature and diluted with dichloromethane (35 mL). The solution was washed with water, and the solvent was removed on a rotary evaporator. The residue was chromatographed on a grade 3 basic alumina column, eluting with dichloromethane. A reddish-purple fraction was collected, and the solvent was removed under reduced pressure. The residue was recrystallized from chloroform-methanol to give the palladium complex (12 mg, 0.0183 mmol, 50%) as a dark-purple solid, mp > 300 °C. UV-vis (CH_2_Cl_2_): λ_max_ (log ε): 308 (4.47), 350 (sh, 4.21), 389 (sh, 4.49), 406 (4.63), 473 (sh, 3.33), 507 (sh, 3.52), 539 (3.65), 570 (3.63), 665 (3.21), 730 (3.41), 805 (3.31). ^1^H NMR (500 MHz, CDCl_3_): δ 7.67 (s, 2,4-H), 7.50 (s, 2H, 6,21-H), 7.22 (s, 2H, 11,16-H), 4.93 (s, 2H, CH_2_CO_2_Me), 3.87 (s, 3H, OCH_3_), 2.99–2.91 (m, 8H, 4 × C*H*_2_CH_3_), 2.57 (s, 6H, 8,19-CH_3_), 1.42 (t, 6H, ^3^*J*_HH_ = 7.6 Hz, 13,14-CH_2_C*H*_3_), 1.37 (t, 6H, ^3^*J*_HH_ = 7.6 Hz, 9,18-CH_2_C*H*_3_). ^13^C NMR (125 MHz, CDCl_3_): δ 169.9, 157.4, 155.8, 153.9, 145.4, 144.0, 141.2, 138.6, 133.6, 132.9, 127.1 (2,4-CH), 126.3 (6,21-CH), 95.6 (11,16-CH), 66.1 (OCH_2_), 52.6 (OCH_3_), 18.9 (13,14-*C*H_2_CH_3_), 18.6 (9,18-*C*H_2_CH_3_), 16.6 (13,14-CH_2_*C*H_3_), 15.3 (9,18-CH_2_*C*H_3_), 10.4 (8,19-CH_3_). HRMS (ESI-TOF) *m*/*z*: M^+^ Calcd for C_35_H_38_N_3_O_3_Pd^+^ 654.1943; Found 654.1934.

**[9,13,14,18-Tetraethyl-3-(methoxycarbonylmethoxy)-8,19-dimethylbenziporphyrinato]nickel(II)** (**7cNi**). Benziporphyrin **40c** (19 mg, 0.0346 mmol) and nickel(II) acetate (40 mg, 0.161 mmol) in DMF (20 mL) were refluxed for 30 min. The solution was cooled to room temperature, diluted with chloroform (20 mL), and washed with water. The solvent was evaporated under reduced pressure, and the residue chromatographed on a grade 3 basic alumina column, eluting with chloroform. A dark-green band was collected, and the solvent was removed under reduced pressure. The residue was recrystallized from chloroform-methanol to give the nickel complex (16 mg, 0.026 mmol, 76%) as a dark-green solid, mp > 300 °C. UV-vis (CH_2_Cl_2_): λ_max_ (log ε): 312 (4.49), 348 (4.45), 405 (4.56), 492 (sh, 3.66), 526 (3.59), 667 (3.69). ^1^H NMR (500 MHz, CDCl_3_): δ 7.61 (s, 2,4-H), 7.29 (s, 2H, 6,21-H), 7.05 (s, 2H, 11,16-H), 4.87 (s, 2H, CH_2_CO_2_Me), 3.85 (s, 3H, OCH_3_), 2.89 (q, 4H, ^3^*J*_HH_ = 7.6 Hz, 13,14-CH_2_), 2.82 (q, 4H, ^3^*J*_HH_ = 7.6 Hz, 9,18-CH_2_), 2.42 (s, 6H, 8,19-CH_3_), 1.36 (t, 6H, ^3^*J*_HH_ = 7.6 Hz, 13,14-CH_2_C*H*_3_), 1.29 (t, 6H, ^3^*J*_HH_ = 7.6 Hz, 9,18-CH_2_C*H*_3_). ^13^C NMR (125 MHz, CDCl_3_): δ 169.9, 159.8, 156.1, 153.7, 146.9, 144.8, 141.5, 140.0, 136.9, 131.6, 126.1 (2,4-CH), 123.0 (6,21-CH), 95.4 (11,16-CH), 66.1 (OCH_2_), 52.5 (OCH_3_), 18.48, 18.46 (4 × *C*H_2_CH_3_), 16.4 (13,14-CH_2_*C*H_3_), 15.2 (9,18-CH_2_*C*H_3_), 10.4 (8,19-CH_3_). HRMS (ESI-TOF) *m*/*z*: M^+^ Calcd for C_35_H_38_N_3_NiO_3_^+^ 606.2261; Found 606.2262.

**1,2-Bis[3,5-dimethoxycarbonylbenzyloxy]benzene** (**16a**). Dimethyl 5-hydroxyisophthalate (2.415 g, 11.5 mmol), α,α’-dibromo-*o*-xylene (1.510 g, 5.72 mmol), and potassium carbonate (1.66 g, 12 mmol) in acetonitrile (100 mL) were refluxed overnight. The solvent was removed on a rotary evaporator, and the solid residue was dissolved in ethyl acetate (75 mL) and washed with water (2 × 30 mL) and saturated sodium bicarbonate (2 × 30 mL). The organic layer was dried over sodium sulfate and filtered, and the solvent was removed under reduced pressure to yield 1,2-bis[3,5-dimethoxycarbonylbenzyloxy]benzene (2.90 g, 5.55 mmol, 97%) as a white solid, mp 150–151 °C. ^1^H NMR (400 MHz, CDCl_3_): δ 8.26 (t, 2H, ^4^*J*_HH_ = 1.4 Hz, 4′,4″-H), 7.78 (d, 4H, ^4^*J*_HH_ = 1.4 Hz, 2′,2″,6′,6″-H), 7.53–7.50 (m, 2H, 3,6-H), 7.42–7.39 (m, 2H, m, 4,5-H), 5.26 (s, 4H, 2 × CH_2_O), 3.92 (s, 12H, s, 4 × OCH_3_). ^13^C NMR (100 MHz, CDCl_3_): δ 166.2 (*C*O_2_CH_3_), 158.8, 134.8, 132.1, 129.7 (4,5-CH), 129.1 (3,6-CH), 123.6 (4′,4″-H), 120.3 (2′,2″,6′,6″-H), 69.0 (\1–\2CH_2_O), 52.6 (4 × OCH_3_). HRMS (ESI-TOF) *m*/*z*: [M + Na]^+^ Calcd for C_28_H_26_NaO_10_^+^ 545.1418; Found 545.1415.

**1,2-Bis[3,5-diformylbenzyloxy]benzene** (**18a**). Lithium aluminum hydride (0.400 g, 10.5 mmol) was added to a solution of 1,2-bis[3,5-dimethoxycarbonylbenzyloxy]benzene (1.006 g, 1.93 mmol) in dry THF (100 mL). The mixture was stirred at room temperature overnight. Dilute hydrochloric acid (40 mL) was added dropwise to the solution, and the contents of the reaction flask were stirred for an additional 30 min. Ethyl acetate (200 mL) was added, and the aqueous layer was drawn off. The organic layer was washed with water (2 × 100 mL) and saturated sodium bicarbonate solution (2 × 100 mL). The organic layer was dried over sodium sulfate and filtered, and the solvent was removed under reduced pressure to yield 1,2-bis[3,5-dihydroxymethylbenzyloxy]benzene (**17a**, 0.735 g, 1.79 mmol, 93%) as a white solid, mp 65–66 °C. ^1^H NMR (500 MHz, DMSO-*d_6_*): δ 7.53–7.50 (m, 2H, 4,5-H), 7.37–7.34 (m, 2H, 3,6-H), 6.86 (br s, 2H, 4′,4″-H), 6.83 (br s, 4H, 2′,2″,6′,6″-H), 5.19 (s, 4H, 2 × CH_2_O), 5.13 (t, 4H, ^3^*J*_HH_ = 5.7 Hz, 4 × OH), 4.43 (8H, d, ^3^*J*_HH_ = 5.7 Hz, 4 × C*H*_2_OH). ^13^C NMR (125 MHz, DMSO-*d_6_*): δ 158.4, 144.1, 135.5, 128.6 (3,6-CH), 128.1 (4,5-CH), 117.2 (4′,4″-CH), 111.1 (2′,2″,6′,6″-CH), 67.1 (2 × CH_2_O), 63.0 (4 × CH_2_OH). Tetraalcohol **17a** (100 mg, 0.246 mmol) was dissolved in dichloromethane (12 mL) and THF (8 mL). Pyridinium chlorochromate (0.330 g, 1.5 mmol) and silica gel (0.342 g, 5.7 mmol) were added to the solution, and the mixture was stirred at room temperature for 1 h. The contents of the reaction flask were immediately chromatographed twice on silica gel, eluting with dichloromethane. The desired column fractions were evaporated under reduced pressure to yield tetraaldehyde **18a** (72 mg, 0.179 mmol, 73%) as a white solid, mp 175–177 °C. ^1^H NMR (400 MHz, DMSO-*d_6_*): δ 10.02 (s, 4H, 4 × CHO), 8.00 (t, 2H, ^4^*J*_HH_ = 1.3 Hz, 4′,4″-H), 7.79 (d, 4H, ^4^*J*_HH_ = 1.3 Hz, 2′,2″,6′,6″-H), 7.61–7.57 (m, 2H, 3,6-H), 7.44–7.40 (m, 2H, 4,5-H), 5.43 (s, 4H, 2 × CH_2_O). ^13^C NMR (100 MHz, DMSO-*d_6_*): δ 192.4 (CHO), 159.5, 138.3, 134.9, 129.3 (4,5-CH), 128.7 (3,6-CH), 123.3 (4′,4″-H), 120.4 (2′,2″,6′,6″-CH), 68.3 (CH_2_O). HRMS (ESI-TOF) *m*/*z*: [M + Na]^+^ Calcd for C_24_H_18_NaO_6_^+^ 425.0996; Found 425.0999.

**1,3-Bis[3,5-dimethoxycarbonylbenzyloxy]benzene** (**16b**). Dimethyl 5-hydroxyisophthalate (735 mg, 3.50 mmol), α,α’-dibromo-*m*-xylene (4.60 mg, 1.74 mmol) and potassium carbonate (500 mg, 3.6 mmol) were dissolved in acetonitrile (30 mL) and refluxed overnight. The solution was cooled, and the solvent was removed on a rotary evaporator. The solid residue was dissolved in ethyl acetate (75 mL) and then washed with water (2 × 30 mL) and saturated sodium bicarbonate solution (2 × 30 mL). The organic layer was dried over sodium sulfate and filtered, and the solvent was evaporated under reduced pressure to afford tetraester **16b** (902 mg, 1.73 mmol, 99%) as a white solid, mp 92–93 °C. ^1^H NMR (500 MHz, CDCl_3_): δ 8.28 (t, 2H, ^4^*J*_HH_ = 1.4 Hz, 4′,4″-H), 7.82 (d, 4H, ^4^*J*_HH_ = 1.4 Hz, 2′,2″,6′,6″-H), 7.53 (br s, 1H, 2-H), 7.43–7.41 (m, 3H, 4,5,6-H), 5.15 (s, 4H, CH_2_O), 3.92 (s, 12H, 4 × OCH_3_). ^13^C NMR (125 MHz, CDCl_3_): δ 166.2 (4 × C=O), 158.9, 136.9, 132.1, 129.2 (5-CH), 127.5 (4,6-CH), 126.7 (2-CH), 123.5 (4′,4″-CH), 120.3 (2′,2″,6′,6″-CH), 70.2 (2 × CH_2_O), 52.6 (4 × OCH_3_). HRMS (ESI-TOF) *m*/*z*: [M + Na]^+^ Calcd for C_28_H_26_NaO_10_^+^ 545.1418; Found 545.1418.

**1,3-Bis[3,5-diformylbenzyloxy]benzene** (**18b**). Lithium aluminum hydride (100 mg, 2.63 mmol) was added to a solution of **16b** (209 mg, 0.400 mmol) in dry THF (50 mL), and the resulting mixture was stirred at room temperature overnight. Dilute hydrochloric acid (30 mL) was added dropwise to the solution, and the contents of the reaction flask were stirred for an additional 30 min. Ethyl acetate (100 mL) was added, and the aqueous layer was drawn off. The organic layer was washed with water (2 × 50 mL) and a saturated sodium bicarbonate solution (2 × 50 mL). The organic solution was dried over sodium sulfate and filtered, and the solvent was removed under reduced pressure to give 1,3-bis[3,5-dihydroxymethylbenzyloxy]benzene (**17b**, 147 mg, 0.358 mmol, 89%) as a white solid, mp 82–84 °C. ^1^H NMR (400 MHz, DMSO-*d_6_*): δ 7.53 (s, 1H, 2-H), 7.42–7.38 (3H, s, 4,5,6-H), 6.86 (br s, 2H, 4′,4″-H), 6.84 (br s, 4H, 2′,2″,6′,6″-H), 5.14 (4H, t, ^3^*J*_HH_ = 5.7 Hz, 2 × OH), 5.09 (s, 4H, 2 × CH_2_O), 4.45 (d, 8H, ^3^*J*_HH_ = 5.7 Hz, 4 × C*H*_2_OH). ^13^C NMR (100 MHz, DMSO-*d_6_*): δ 158.5, 144.1, 137.7, 128.7 (5-CH), 127.1 (4,6-CH), 126.8 (2-CH), 117.1 (2′,2″,6′,6″-CH), 111.2 (4′,4″-CH), 69.2 (CH_2_O), 63.0 (CH_2_OH). Tetraalcohol **17b** (100 mg, 0.244 mmol) was dissolved in dichloromethane (12 mL) and THF (8 mL), pyridinium chlorochromate (0.426 g, 2.0 mmol) and silica gel (0.208 g, 3.5 mmol) were added, and the mixture was stirred at room temperature for 1 h. The contents of the reaction flask were immediately run through a short silica gel column and eluted with dichloromethane. The solvent was evaporated, and the residue was purified on silica, eluting fist with dichloromethane and then with chloroform. The product fractions were evaporated under reduced pressure, and the residue was recrystallized from chloroform-hexanes to yield the tetraaldehyde (55 mg, 0.137 mmol, 56%) as a white solid, mp 158–159 °C. ^1^H NMR (500 MHz, CDCl_3_): δ 10.05 (s, 4H, 4 × CHO), 7.98 (t, 2H, ^4^*J*_HH_ = 1.3 Hz, 4′,4″-H), 7.73 (d, 4H, ^4^*J*_HH_ = 1.3 Hz, 2′,2″,6′,6″-H), 7.55 (br s, 1H, 2-H), 7.48–7.43 (m, 3H, 4,5,6-H), 5.22 (s, 4H, CH_2_O). ^13^C NMR (125 MHz, CDCl_3_): δ 190.9 (4 × C=O), 160.0, 138.7, 136.6, 129.5 (5-CH), 127.8 (4,6-CH), 126.7 (2-CH), 124.8 (4′,4″-CH), 120.4 (2′,2″,6′,6″-CH), 70.6 (2 × CH_2_O). HRMS (ESI-TOF) *m*/*z*: [M + Na]^+^ Calcd for C_24_H_18_NaO_6_^+^ 425.0996; Found 425.1000.

**1,4-Bis[3,5-dimethoxycarbonylbenzyloxy]benzene** (**16c**). Dimethyl 5-hydroxyisophthalate (2.508 g, 11.9 mmol), α,α’-dibromo-*p*-xylene (1.066 g, 4.04 mmol), and potassium carbonate (1.70 g, 12.3 mmol) were refluxed in acetonitrile (100 mL) overnight. After the solution was cooled, the solvent was evaporated on a rotary evaporator. The residue was dissolved in ethyl acetate (50 mL) and washed with water (2 × 30 mL) and saturated sodium bicarbonate solution (2 × 30 mL). The organic layer was dried over sodium sulfate and filtered, and the solvent was removed under reduced pressure to give **16c** (1.42 g, 2.72 mmol, 67%) as a white solid, mp 197–200 °C. ^1^H NMR (400 MHz, CDCl_3_): δ 8.30 (t, 2H, ^4^*J*_HH_ = 1.4 Hz, 4′,4″-H), 7.84 (d, 4H, ^4^*J*_HH_ = 1.4 Hz, 2′,2″,6′,6″-H), 7.48 (s, 4H, 2,3,5,6-H), 5.16 (s, 4H, 2 × CH_2_O), 3.94 (12H, s, 4 × OCH_3_). ^13^C NMR (100 MHz, CDCl_3_): δ 166.3, 158.9, 136.4, 132.1, 128.1 (2,3,5,6-CH), 123.5 (4′,4″-CH), 120.4 (2′,2″,6′,6″-CH), 70.4 (CH_2_O), 52.6 (CO_2_*C*H_3_). HRMS (ESI-TOF) *m*/*z*: [M + Na]^+^ Calcd for C_28_H_26_NaO_10_^+^ 545.1418; Found 545.1418.

**1,4-Bis[3,5-diformylbenzyloxy]benzene** (**18c**). Lithium aluminum hydride (0.200 g, 5.3 mmol) was added to a solution of **16c** (0.472 g, 0.904 mmol) in dry THF (50 mL). The mixture was stirred at room temperature overnight. Dilute hydrochloric acid (30 mL) was added dropwise to the solution, and the contents of the reaction flask were stirred for an additional 30 min. Ethyl acetate (100 mL) was added, and the aqueous layer was drawn off. The organic layer was washed with water (2 × 50 mL) and saturated sodium bicarbonate solution (2 × 50 mL). The ethyl acetate layer was dried over sodium sulfate and filtered, and the solvent was removed under reduced pressure to yield 1,4-bis[3,5-dihydroxymethylbenzyloxy]benzene (**17c**, 0.369 g, 0.90 mmol, 99%) as a white solid, mp 165–167 °C. ^1^H NMR (400 MHz, DMSO-*d_6_*): δ 7.45 (s, 4H, 2,3,5,6-H), 6.85 (br s, 2H, 4′,4″-H), 6.82 (br s, 4H, 2′,2″,6′,6″-H), 5.13 (t, 4H, ^3^*J*_HH_ = 5.7 Hz, 2 × OH), 5.08 (s, 4H, 2 × CH_2_O), 4.44 (d, 8H, ^3^*J*_HH_ = 5.7 Hz, 4 × C*H*_2_OH). ^13^C NMR (100 MHz, DMSO-*d_6_*): δ 158.5, 144.1, 137.0, 127.8 (2,3,5,6-CH), 117.0 (4′,4″-CH), 111.1 (2′,2″,6′,6″-CH), 69.0 (CH_2_O), 63.0 (CH_2_OH). Tetraalcohol **17c** (94 mg, 0.23 mmol), was dissolved in dichloromethane (12 mL) and THF (8 mL), pyridinium chlorochromate (0.426 g, 2.0 mmol) and silica gel (0.208 g, 3.5 mmol) were added, and the mixture was stirred at room temperature for 1 h. The contents of the reaction flask were immediately chromatographed twice on silica gel, eluting with dichloromethane. The desired column fractions were evaporated under reduced pressure to yield the tetraaldehyde (90 mg, 0.224 mmol, 97%) as a white solid, mp 195–198 °C. ^1^H NMR (500 MHz, DMSO-*d_6_*): δ 10.06 (s, 4H, CHO), 8.04 (br t, 2H, ^4^*J*_HH_ = 1.2 Hz, 4′,4″-H), 7.83 (br d, 4H, ^4^*J*_HH_ = 1.2 Hz, 2′,2″,6′,6″-H), 7.53 (s, 4H, 2,3,5,6-H), 5.30 (s, 4H, 2 × CH_2_O). ^13^C NMR (125 MHz, DMSO-*d_6_*): δ 192.5 (CHO), 159.5, 138.4, 136.3, 128.1 (2,3,5,6-CH), 123.1 (4′,4″-CH), 120.5 (2′,2″,6′,6″-CH), 69.8 (CH_2_O). HRMS (ESI-TOF) *m*/*z*: [M + Na]^+^ Calcd for C_24_H_18_NaO_6_^+^ 425.0996; Found 425.1005.

## 4. Conclusions

A series of nonaromatic 3-alkoxybenziporphyrins were synthesized in 34–44% yield by reacting 5-alkoxyisophthalaldehydes with a tripyrrane dicarboxylic acid. Stepwise protonation of the macrocycles was observed to give mono- and diprotonated species. The proton NMR spectra for the diprotonated dications showed significant upfield shifts to the internal C-H resonances, and this demonstrated that a degree of overall global diatropic character was present. This can be attributed to dipolar resonance contributors that possess 18π-electron delocalization pathways. Metalation of the benziporphyrins with nickel(II) or palladium(II) acetate afforded the corresponding nickel(II) or palladium(II) organometallic complexes in 50–76% isolated yields. These derivatives exhibited enhanced diatropicity compared to the parent free base benziporphyrins, although the nickel(II) complexes showed less diatropic character than their palladium(II) counterparts. This may be due in part to the palladium complexes taking on more planar conformations, as the porphyrinoid macrocycle may be distorted to facilitate coordination to smaller nickel(II) cations. Although the strategy used to prepare 3-alkoxybenziporphyrins was quite successful, attempts to apply the methodology to the synthesis of benziporphyrin dimers did not prove to be fruitful. Nevertheless, this study provides access to structurally novel carbaporphyrinoids and gives insights into the effects that result from the introduction of electron-donating substituents.

## Data Availability

The data presented in this article are available in the Supporting Information section.

## Supplementary Materials

The following supporting information can be downloaded at: https://www.mdpi.com/article/10.3390/molecules29081903/s1, Figures S1–S20: selected UV-vis spectra; Figures S21–S133: selected proton, ^1^H-^1^H COSY, HSQC, DEPT-135, and carbon-13 NMR spectra; Figures S134–S149: selected HR-TOF-ESI mass spectra.
