# Supplementary material for: Synthesis, Spectroscopic Properties, and Metalation of 3-Alkoxybenziporphyrins
Source: Molecules. 2024 Apr 22;29(8):1903. doi: 10.3390/molecules29081903 (PMC11054816; doi:10.3390/molecules29081903)
Supplement: Supplementary file 1 [file molecules-29-01903-s001.zip › molecules-2965168-supplementary.pdf]

Supporting information for

Synthesis, Metalation and Spectroscopic Characterization of 3-Alkoxybenziporphyrins

Rachel A. Tomlovich and Timothy D. Lash, Department of Chemistry, llinois State University, Normal, llinois 61790-4160

Table of Contents

Page

S2-S11 Selected UV-Vis spectra (Figures S1-S20)

S12-S95 Selected proton, DEPT-135, ^1H-^1H COSY, HSQC and carbon-13 NMR spectra (Figures S21-S133)

S96-S107 Selected mass spectra (Figures S134-S149)

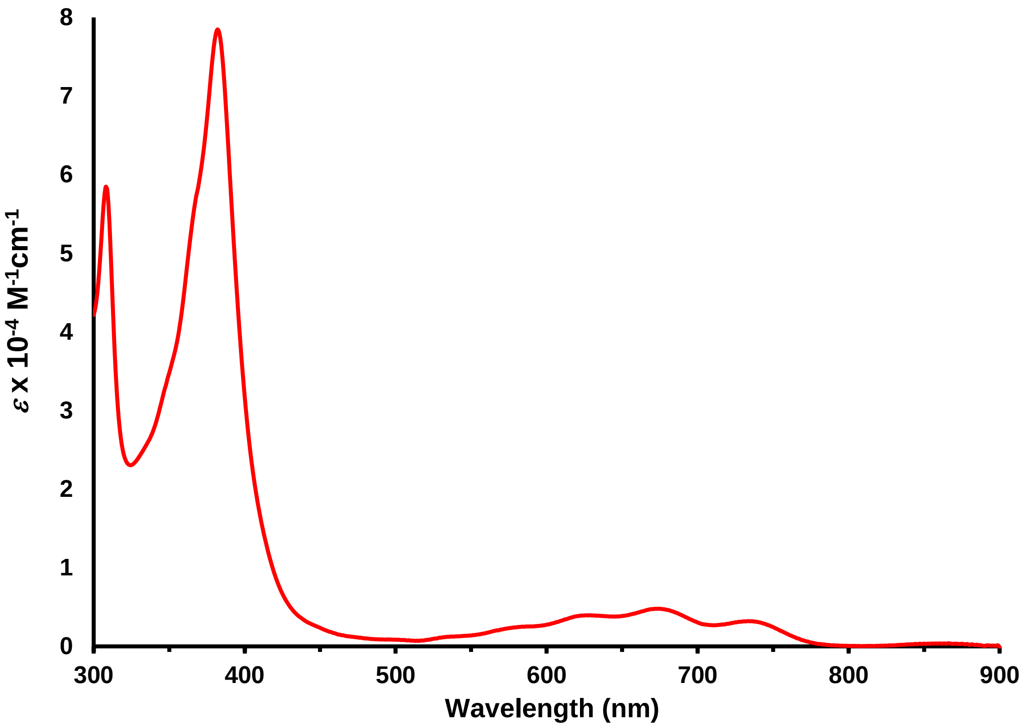

Figure S1. UV-vis spectrum of 3-ethoxybenziporphyrin **7b** in CH_2Cl_2.

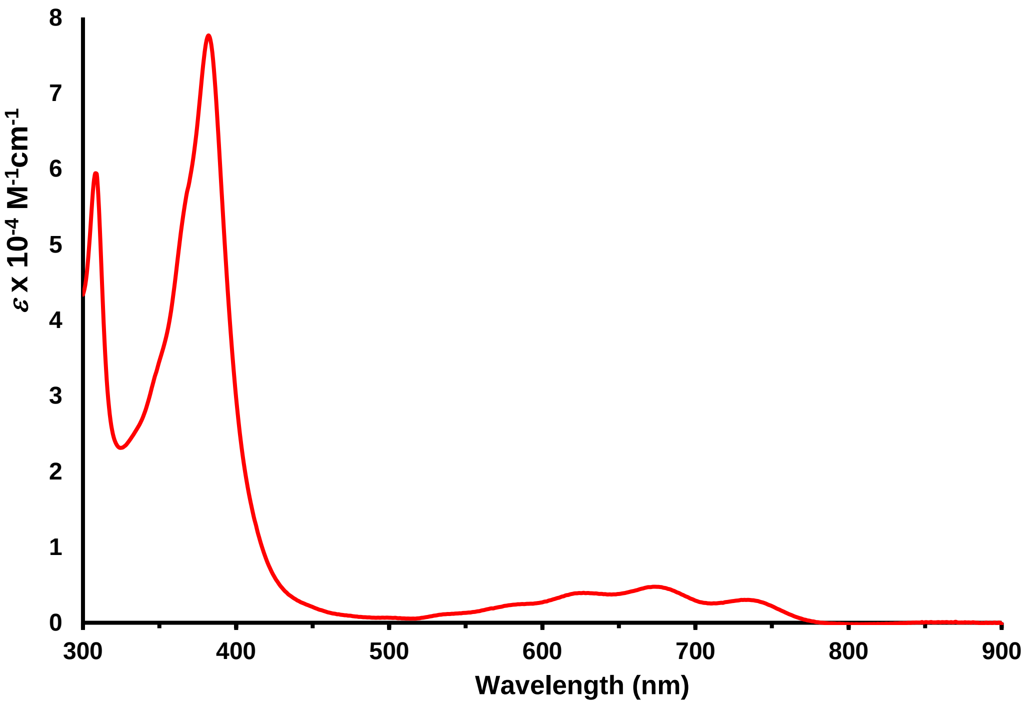

Figure S2. UV-vis spectrum of 3-ethoxybenziporphyrin **7b** in 1% Et_3N-CH_2Cl_2.

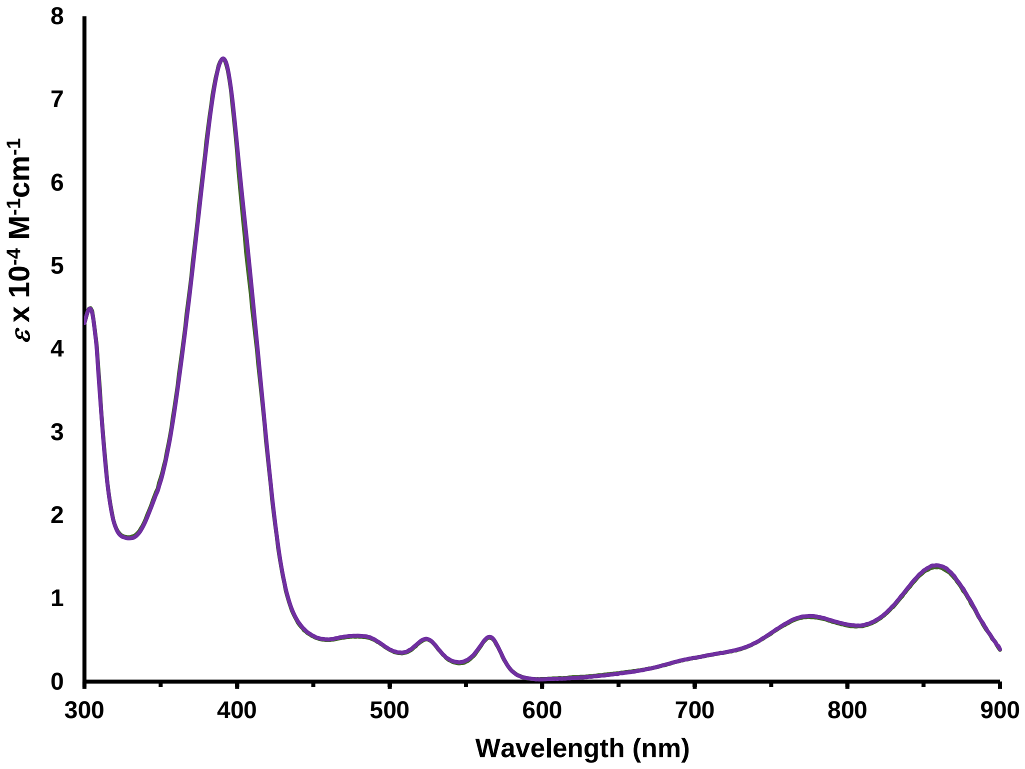

Figure S3. UV-vis spectrum of 3-ethoxybenziporphyrin **7b** in CH_2Cl_2 with 5 equivalents of TFA.

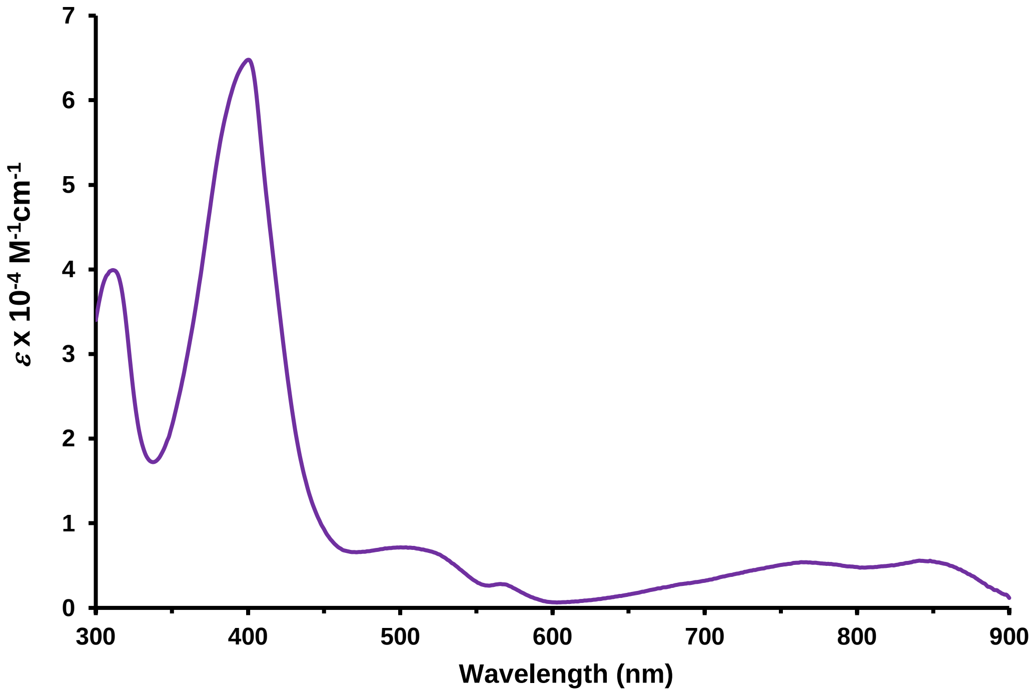

Figure S4. UV-vis spectrum of 3-ethoxybenziporphyrin **7b** in CH_2Cl_2 with 500 equivalents of TFA.

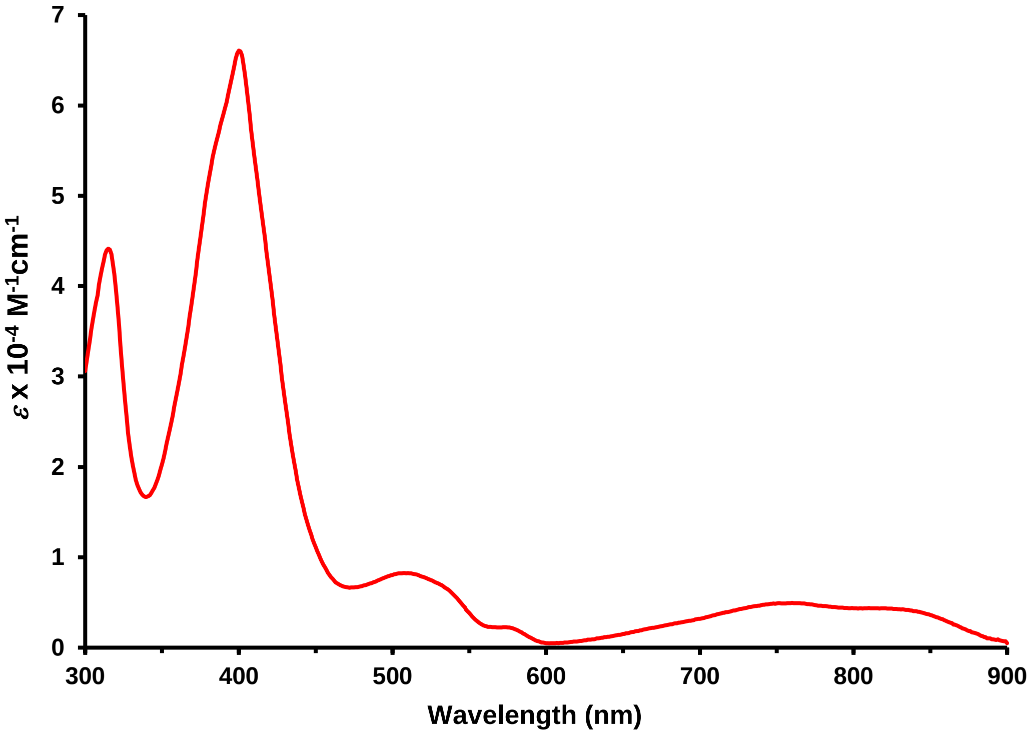

Figure S5. UV-vis spectrum of 3-ethoxybenziporphyrin **7b** in 1% TFA-CH_2Cl_2.

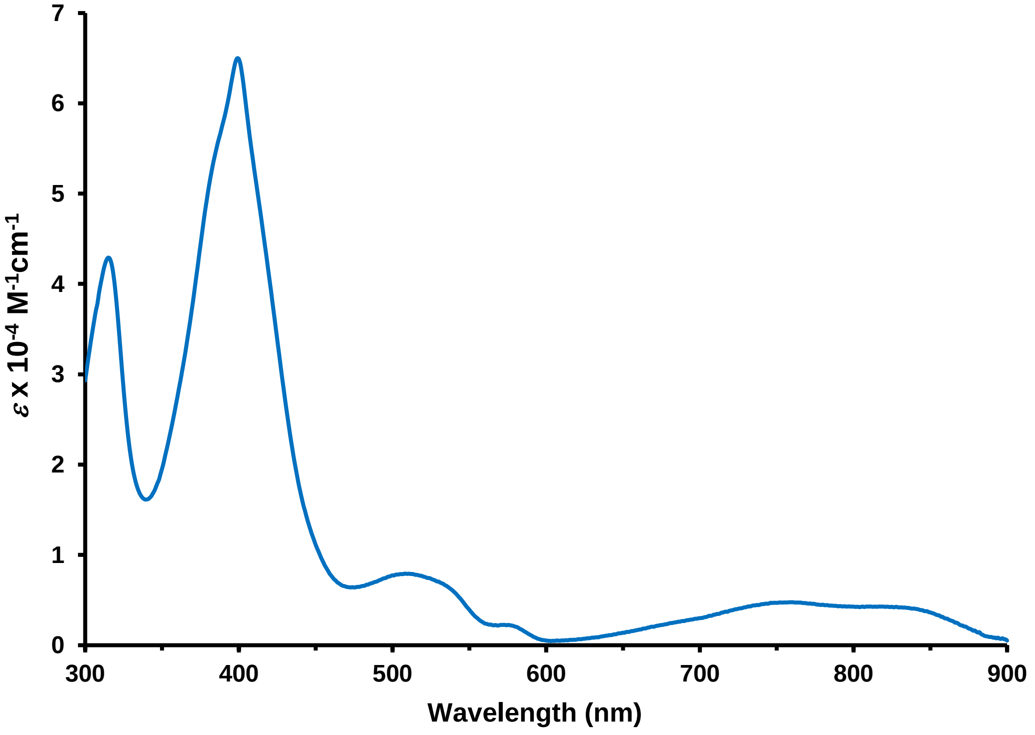

Figure S6. UV-vis spectrum of 3-ethoxybenziporphyrin **7b** in 5% TFA- CH_2Cl_2.

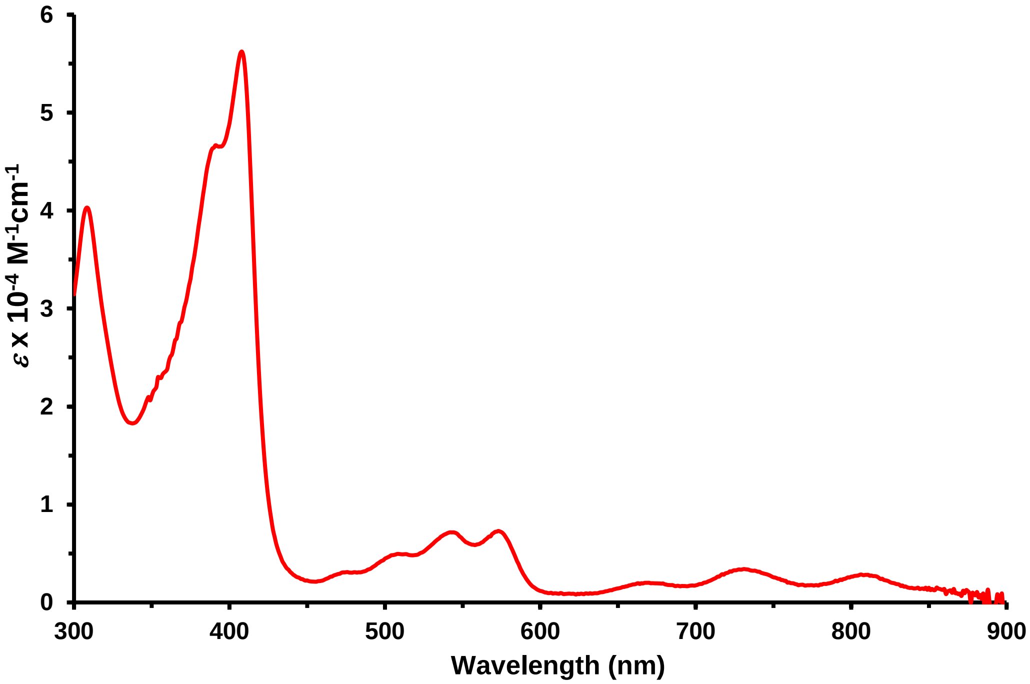

Figure S7. UV-vis spectrum of Pd(II) complex **7bPd** in CH_2Cl_2.

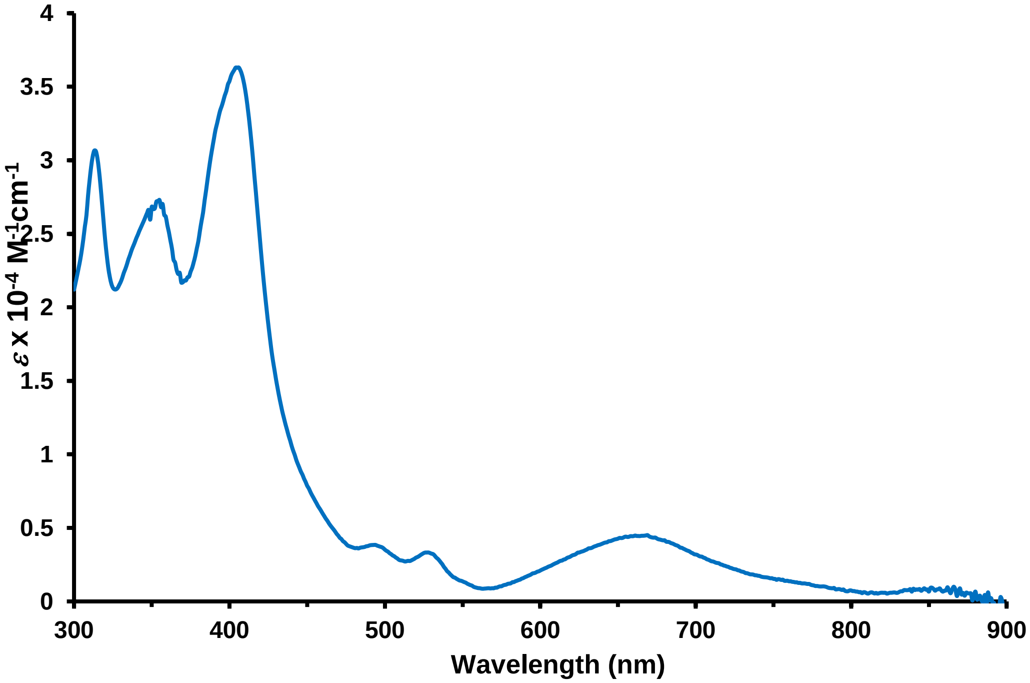

Figure S8. UV-vis spectrum of Ni(II) complex **7bNi** in CH_2Cl_2.

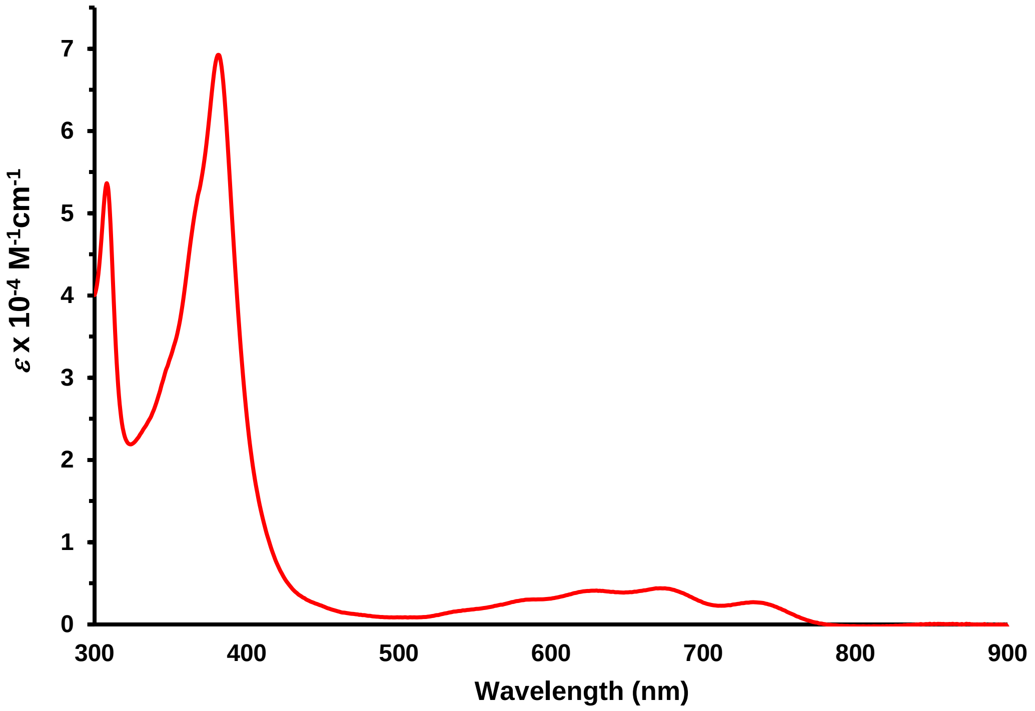

Figure S9. UV-vis spectrum of methoxybenziporphyrin **7a** in 1% Et_3N-CH_2Cl_2.

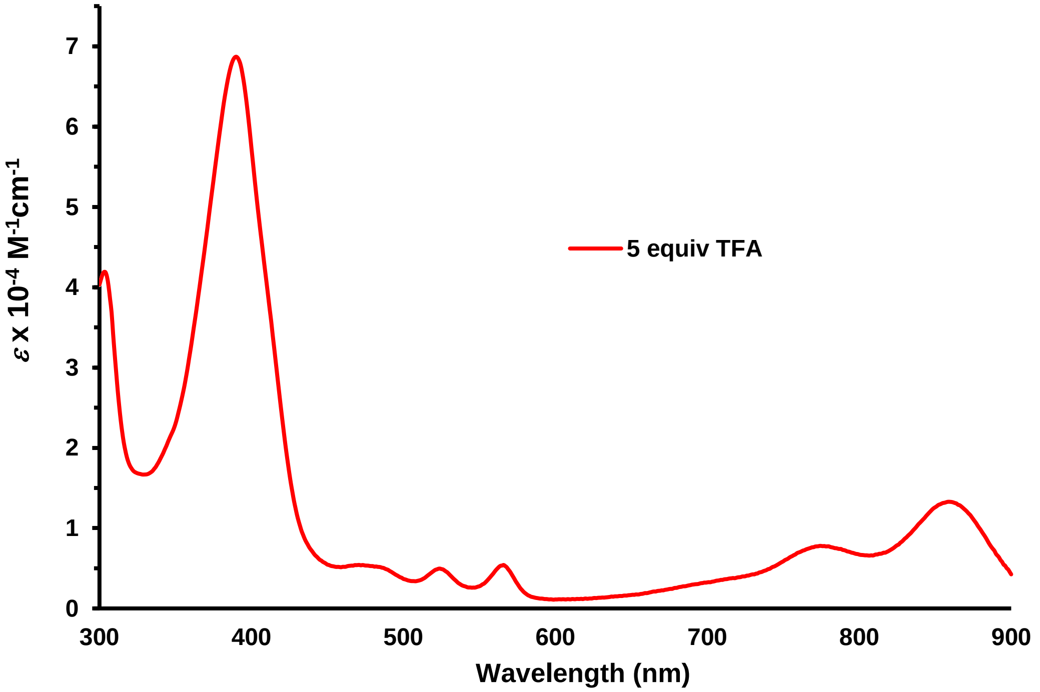

Figure S10. UV-vis spectrum of methoxybenziporphyrin **7a** in CH_2Cl_2 with 5 equivalents of TFA.

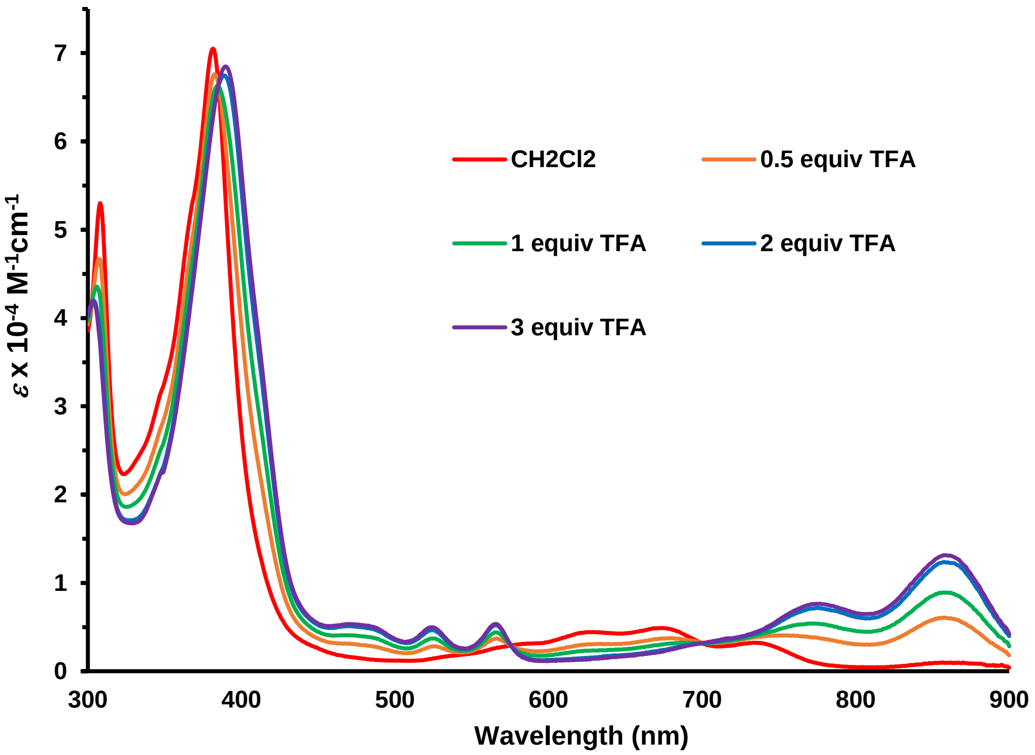

Figure S11. UV-vis spectra of methoxybenziporphyrin **7a** in CH_2Cl_2 with 0-3 equivalents of TFA.

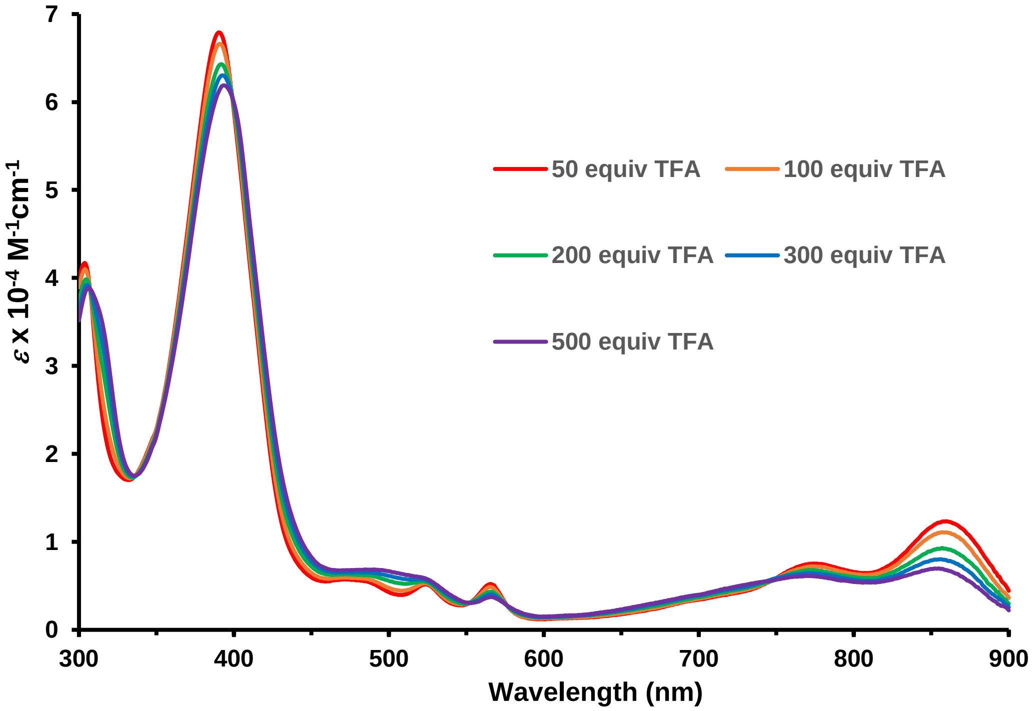

Figure S12. UV-vis spectra of methoxybenziporphyrin **7a** in CH_2Cl_2 with 50-500 equivalents of TFA.

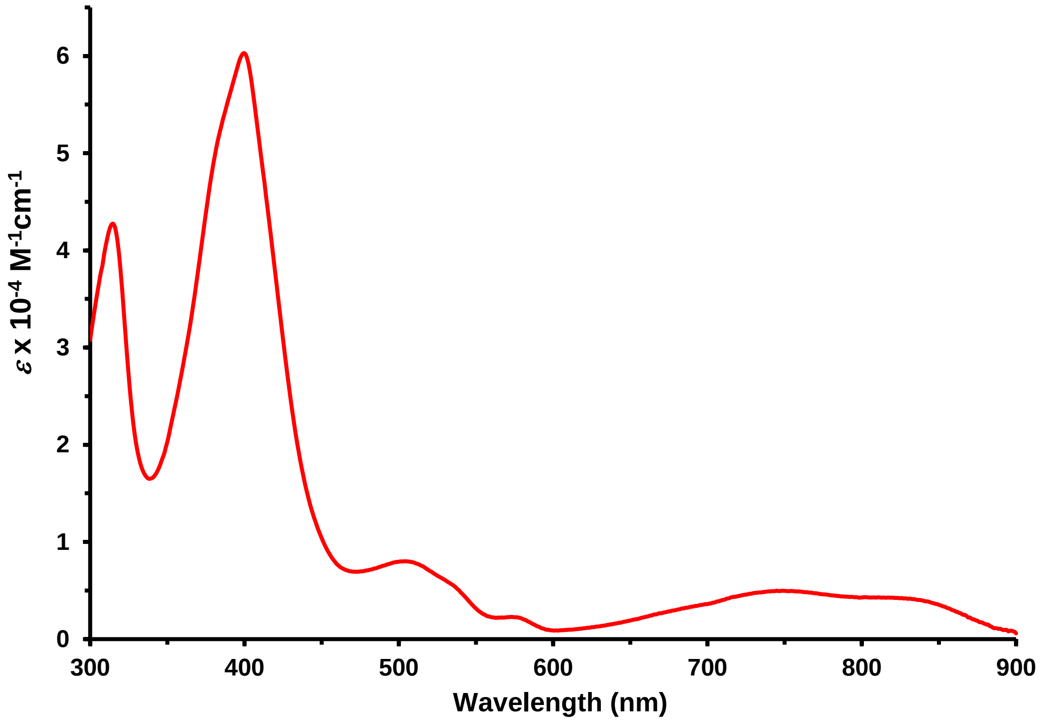

Figure S13. UV-vis spectrum of methoxybenziporphyrin **7a** in 1% TFA-CH_2Cl_2.

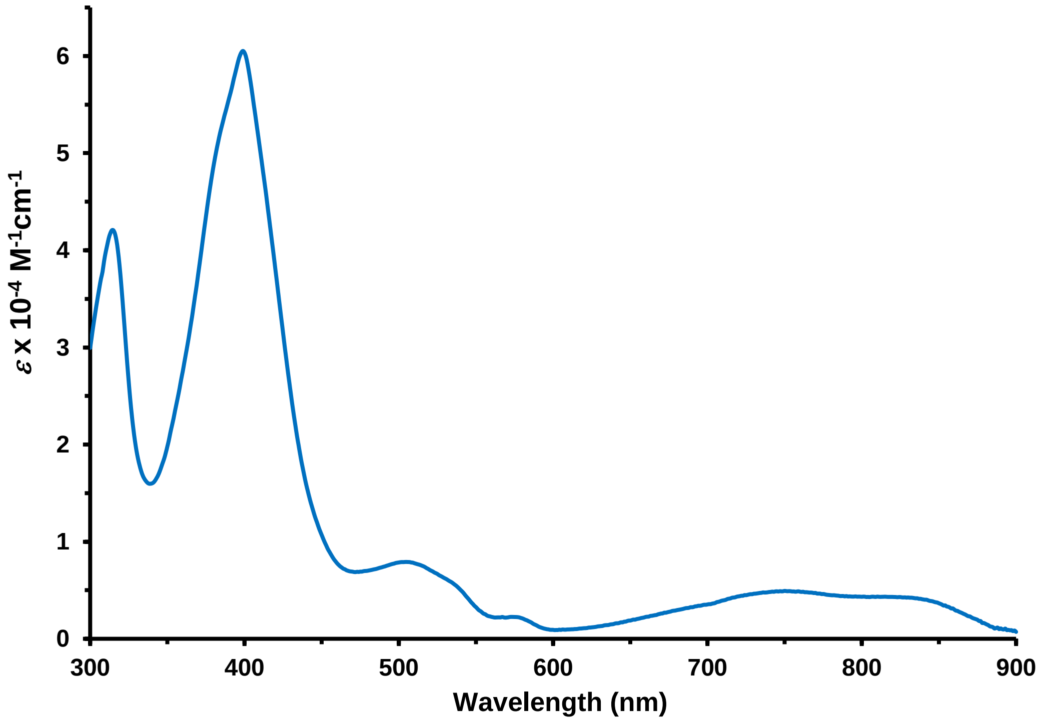

Figure S14. UV-vis spectrum of methoxybenziporphyrin **7a** in 5% TFA-CH_2Cl_2.

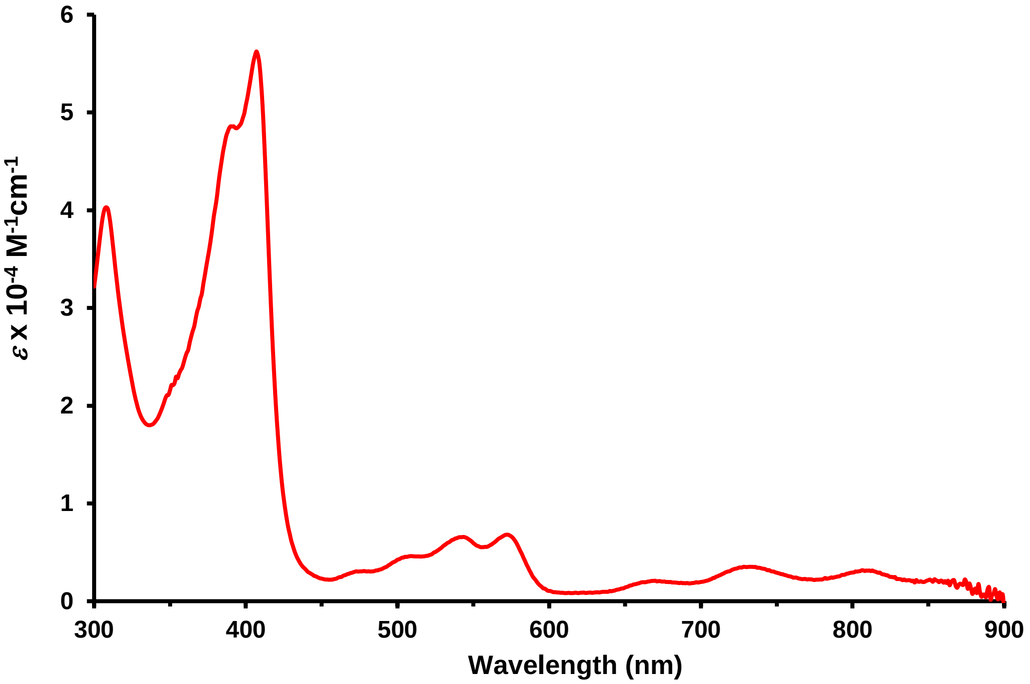

Figure S15. UV-vis spectrum of palladium(II) complex **7aPd** in CH_2Cl_2.

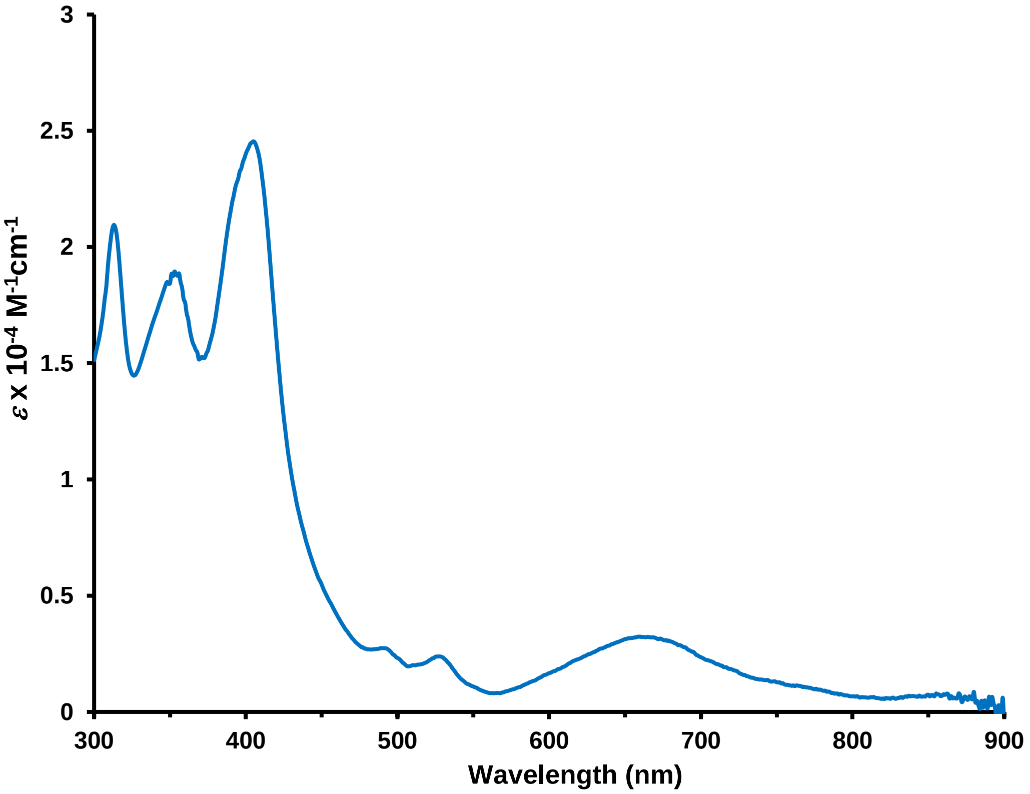

Figure S16. UV-vis spectrum of nickel(II) complex **7aNi** in CH_2Cl_2.

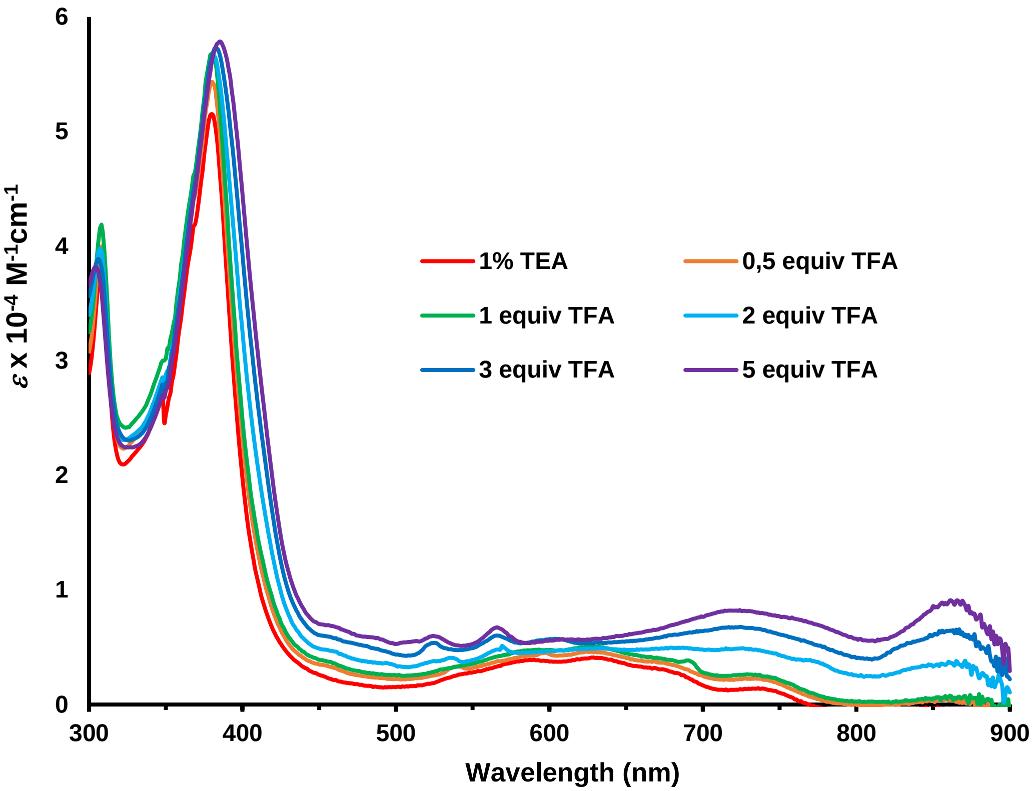

Figure S17. UV-vis spectra of methoxycarbonylmethoxybenziporphyrin **7c** in 1% Et_3N-CH_2Cl_2 and with 0.5-5 equivalents of TFA in CH_2Cl_2.

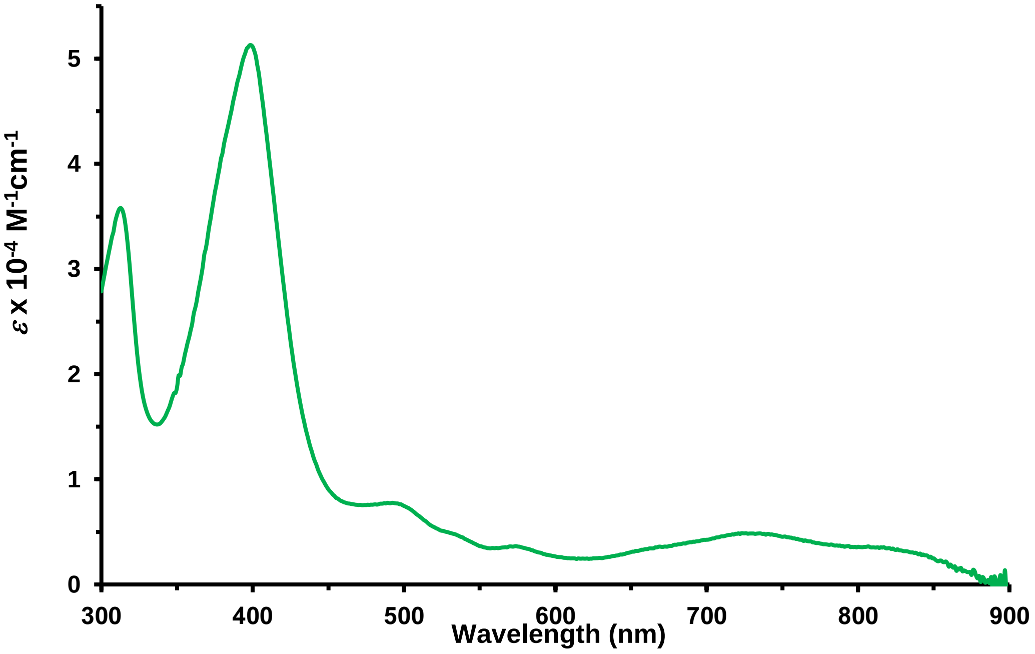

Figure S18. UV-vis spectrum of **7c** in 1% TFA-CH_2Cl_2.

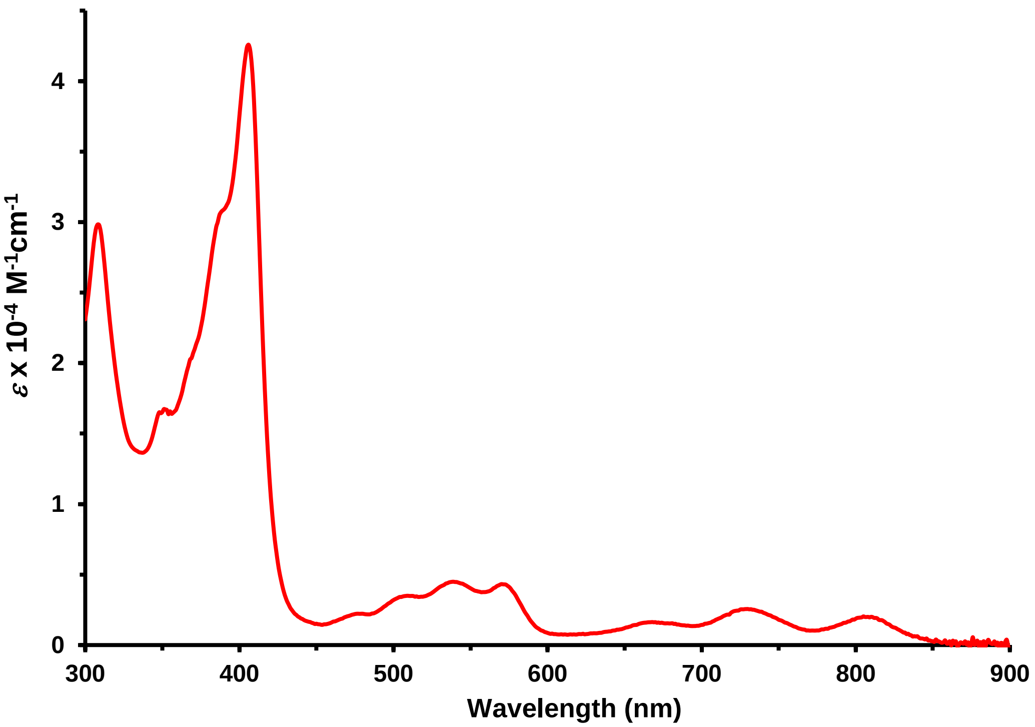

Figure S19. UV-vis spectrum of palladium(II) complex **7cPd** in CH_2Cl_2.

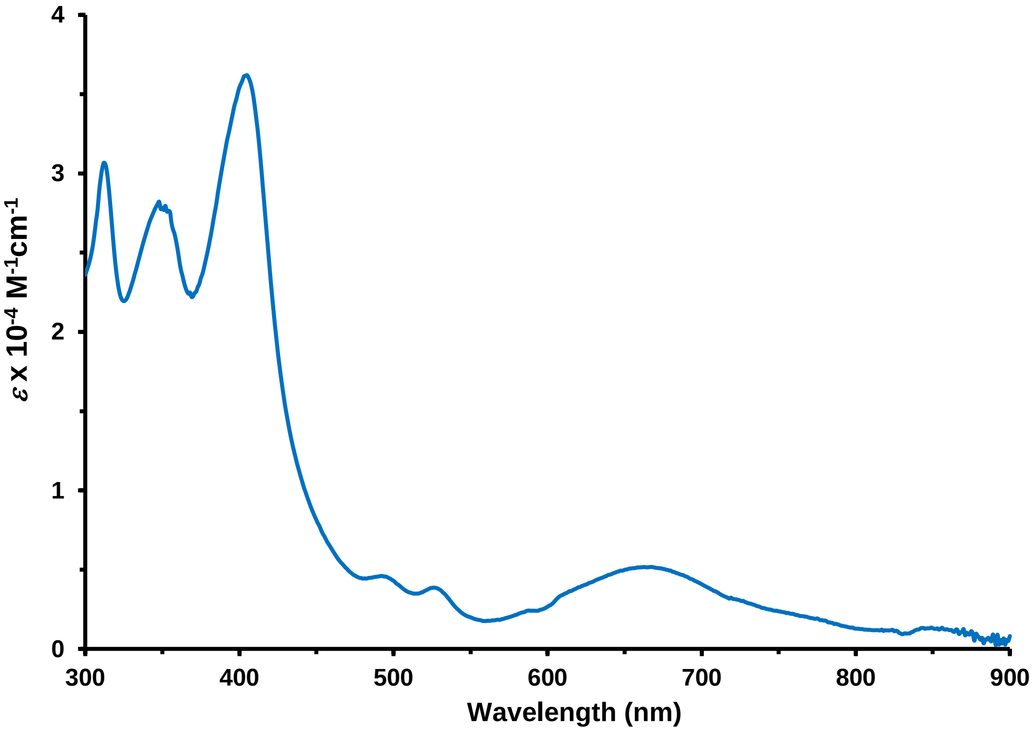

Figure S20. UV-vis spectrum of nickel(II) complex **7cNi** in CH_2Cl_2.

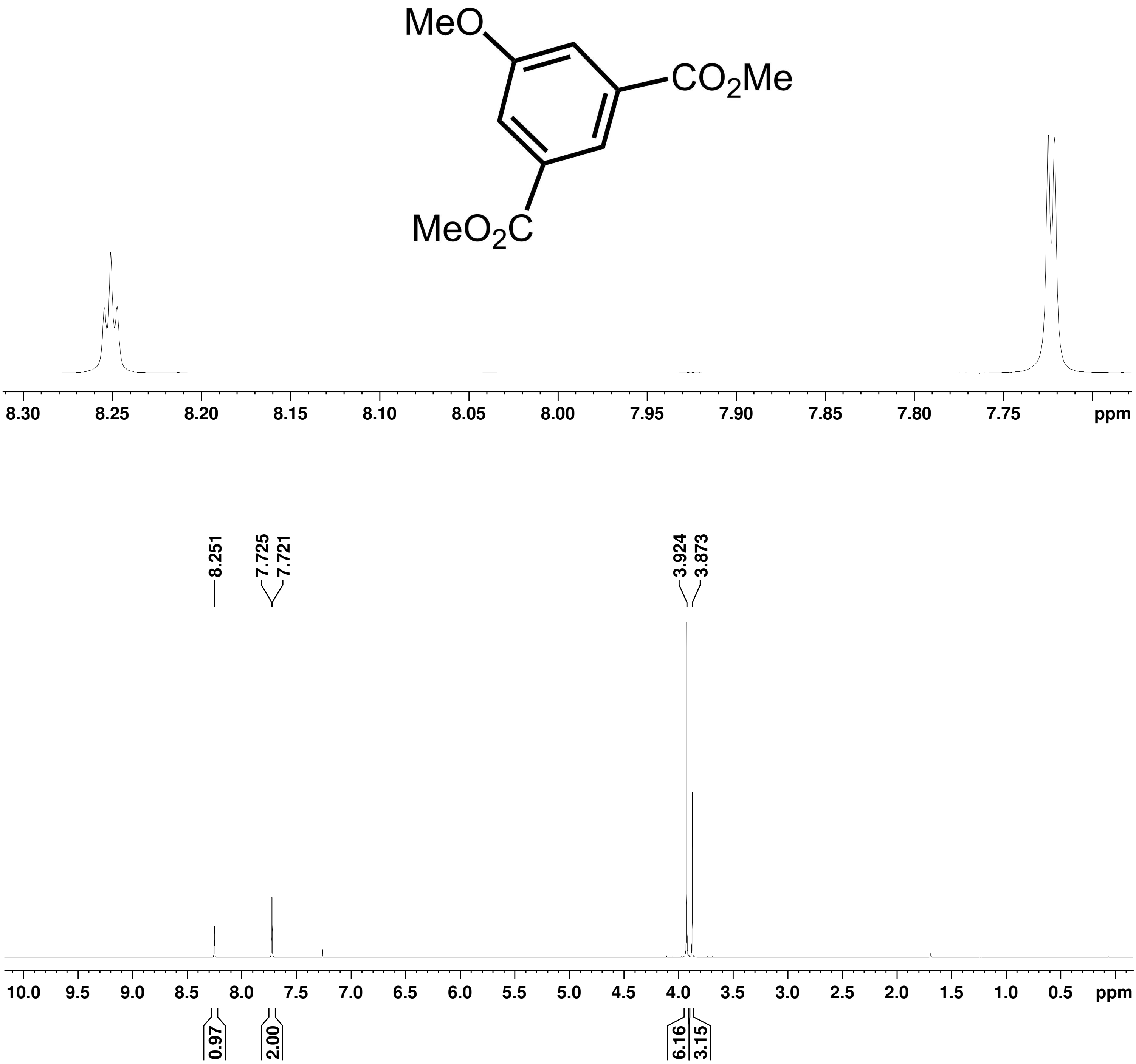

Figure S21. 400 MHz proton NMR spectrum of dimethyl 5-methoxy-1,3-benzenedicarboxylate **11a** in $CDCl_3$.

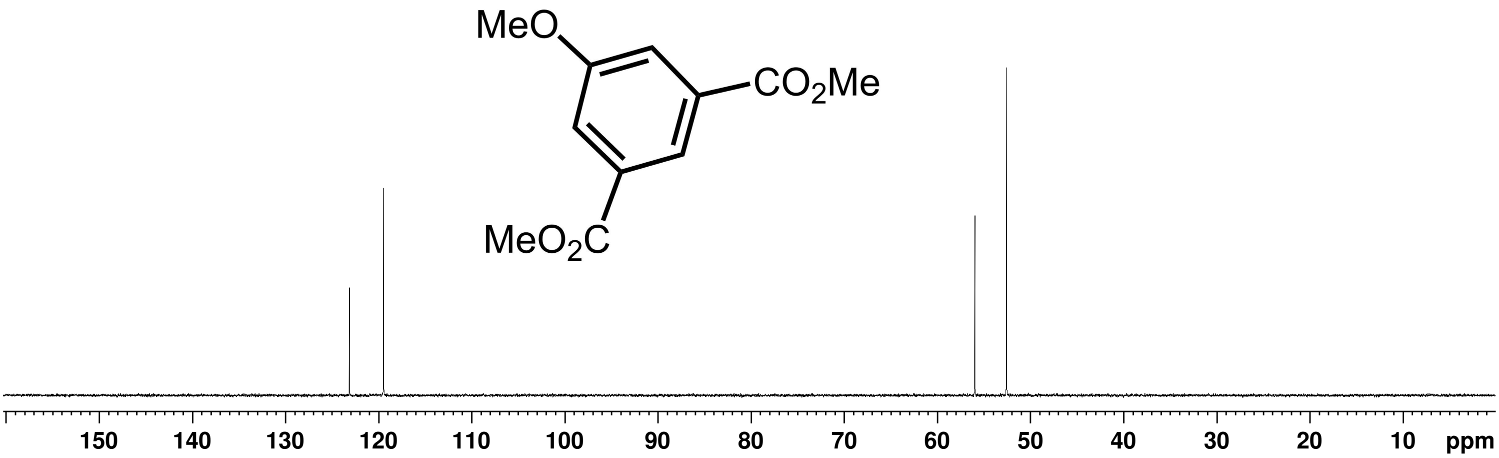

Figure S22. DEPT-135 NMR spectrum of dimethyl 5-methoxy-1,3-benzenedicarboxylate in $CDCl_3$.

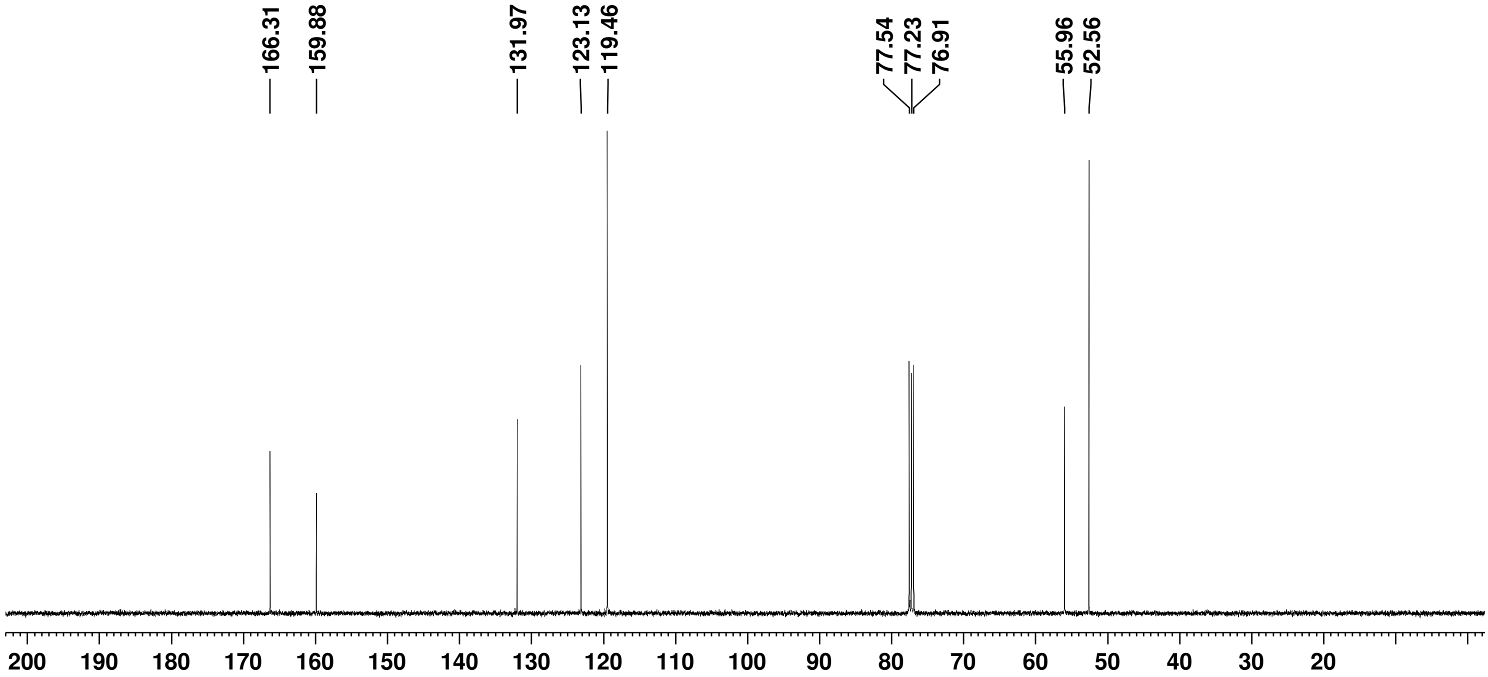

Figure S23. 100 MHz carbon-13 NMR spectrum of dimethyl 5-methoxy-1,3-benzenedicarboxylate in $CDCl_3$.

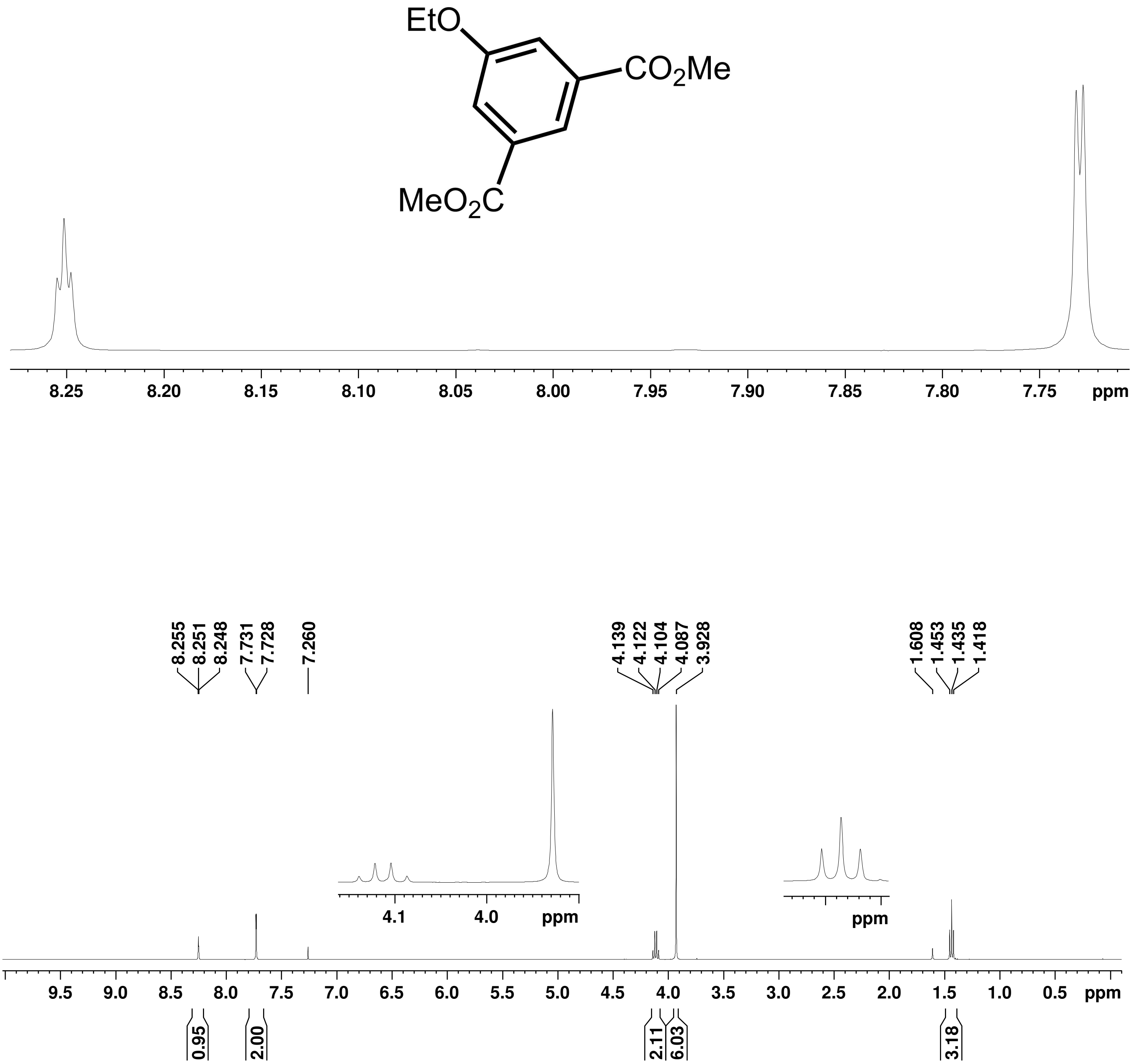

Figure S24. 400 MHz proton NMR spectrum of dimethyl 5-ethoxy-1,3-benzenedicarboxylate **11b** in $CDCl_3$.

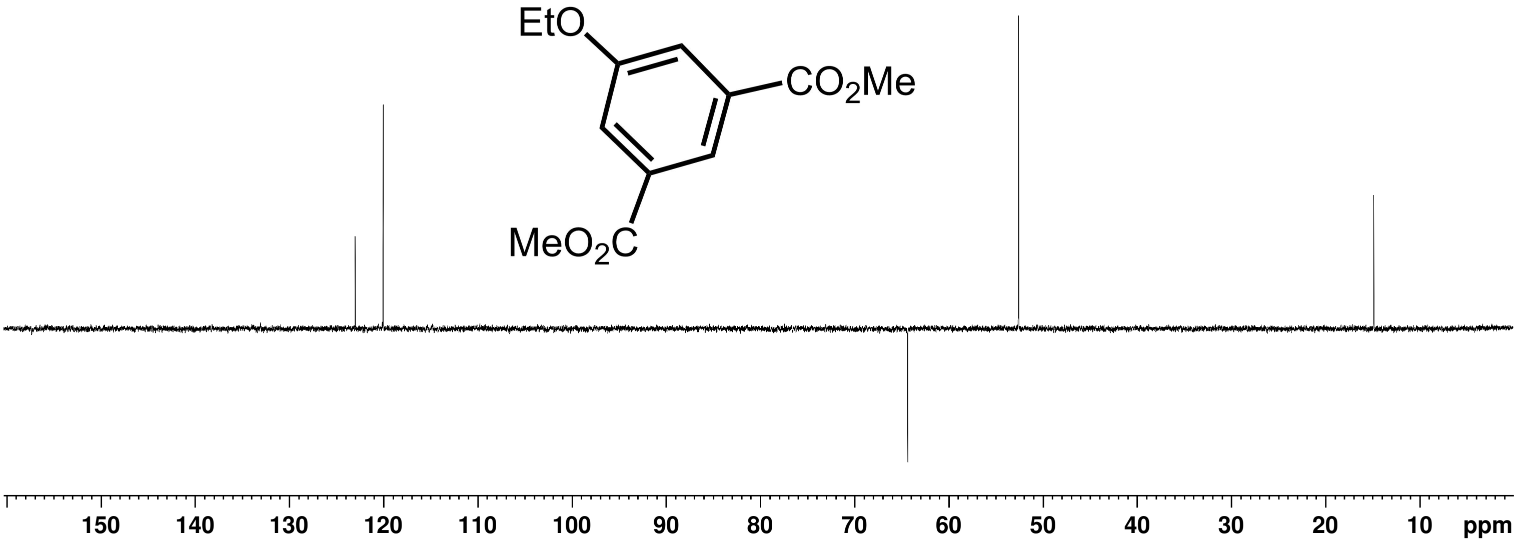

Figure S25. DEPT-135 NMR spectrum of dimethyl 5-ethoxy-1,3-benzenedicarboxylate in $CDCl_3$.

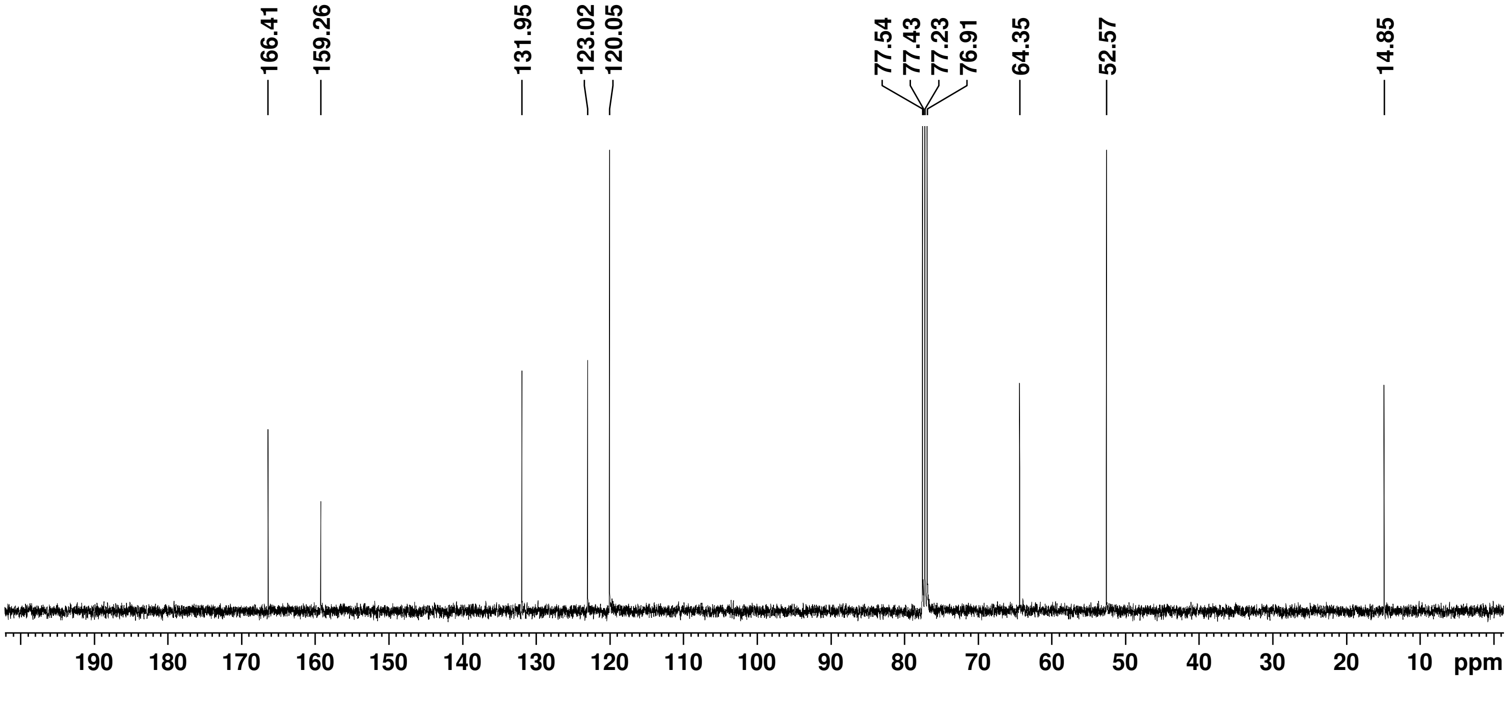

Figure S26. 100 MHz carbon-13 NMR spectrum of dimethyl 5-ethoxy-1,3-benzenedicarboxylate in $CDCl_3$.

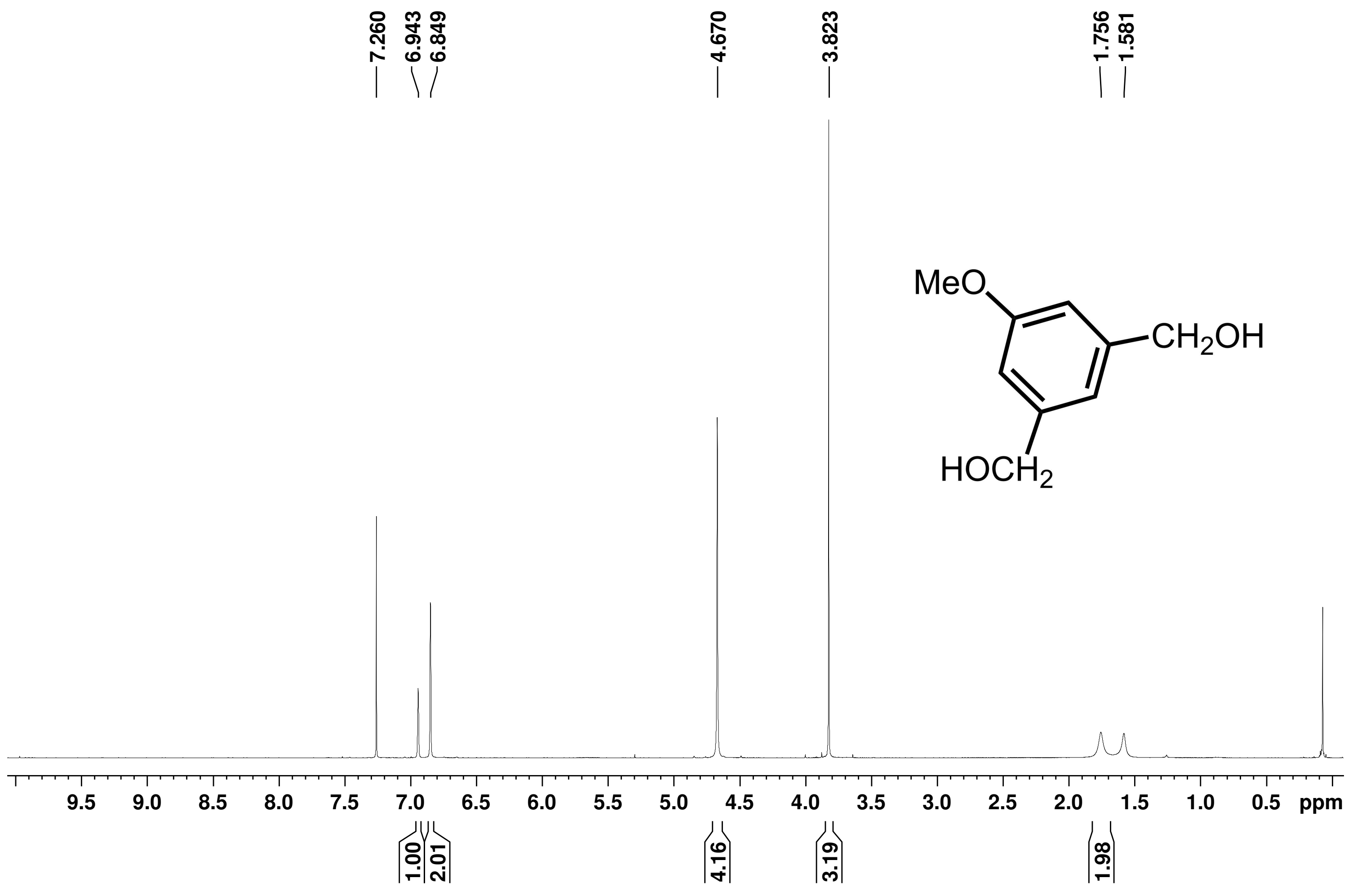

Scheme S27. 400 MHz proton NMR spectrum of methoxybenzenedicarbinol **12a** in $CDCl_3$.

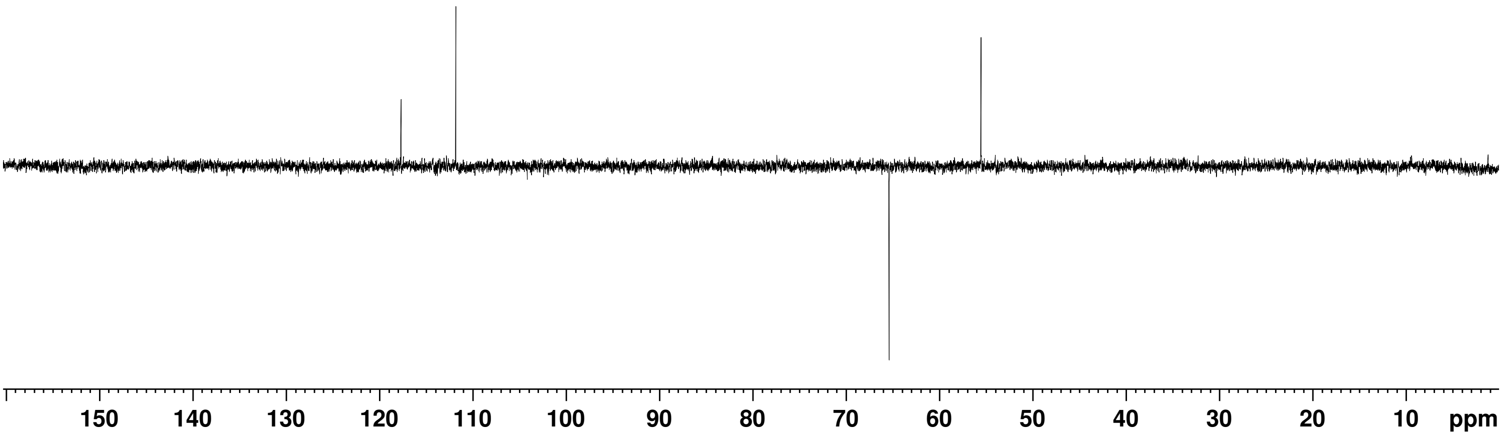

Scheme S28. DEPT-135 NMR spectrum of methoxybenzenedicarbinol **12a** in $CDCl_3$.

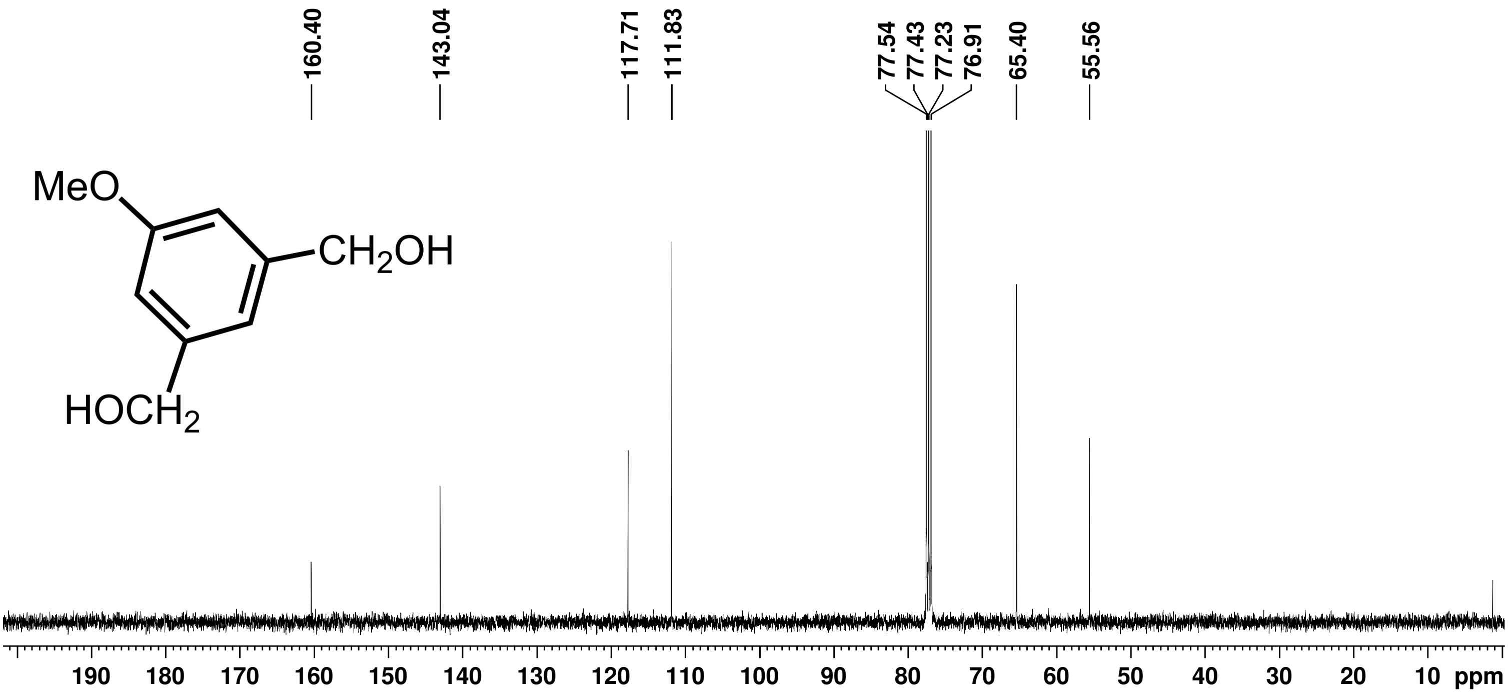

Scheme S29. 100 MHz carbon-13 NMR spectrum of methoxybenzenedicarbinol **12a** in $CDCl_3$.

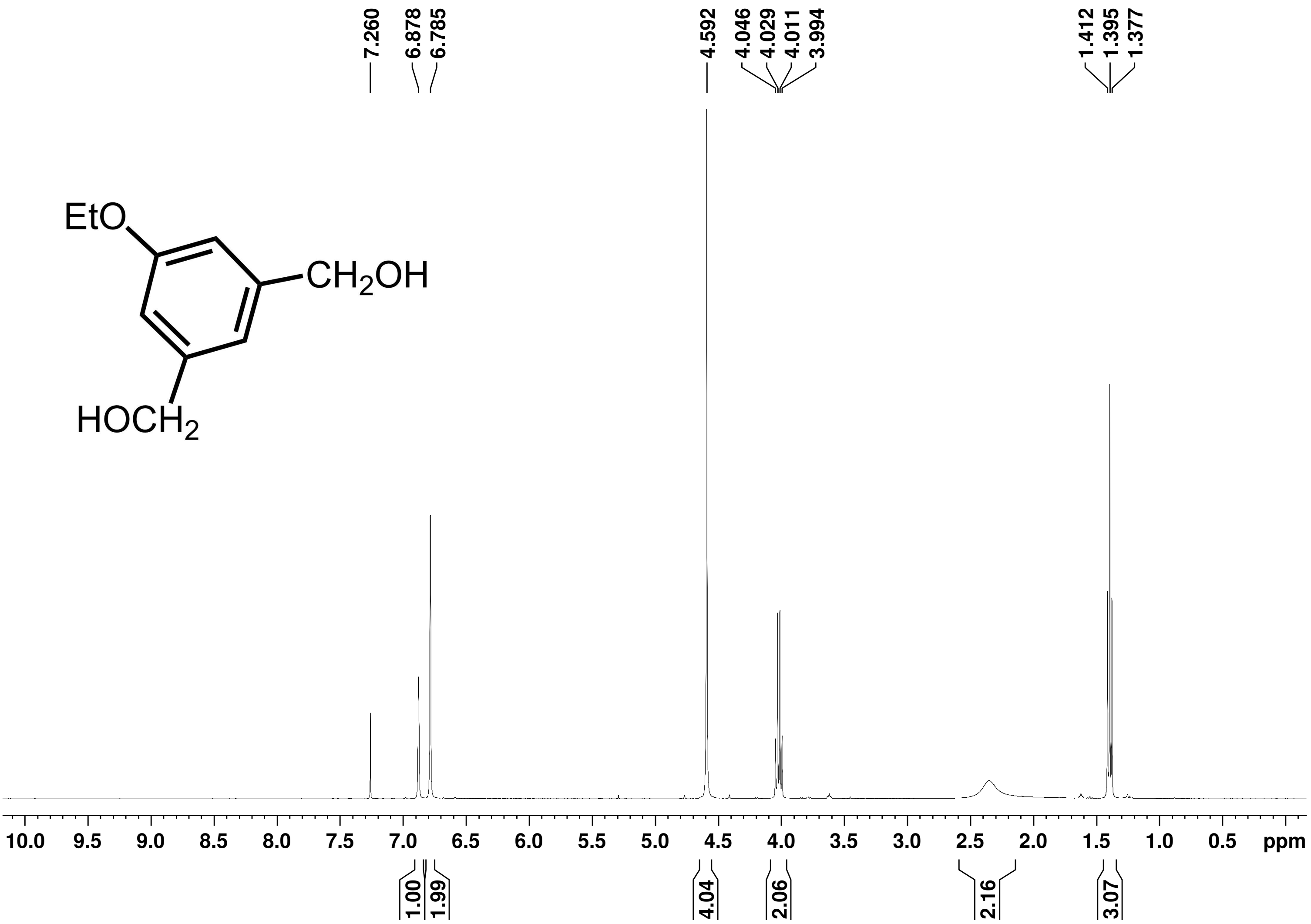

Scheme S30. 400 MHz proton NMR spectrum of ethoxybenzenedicarbinol **12b** in $CDCl_3$.

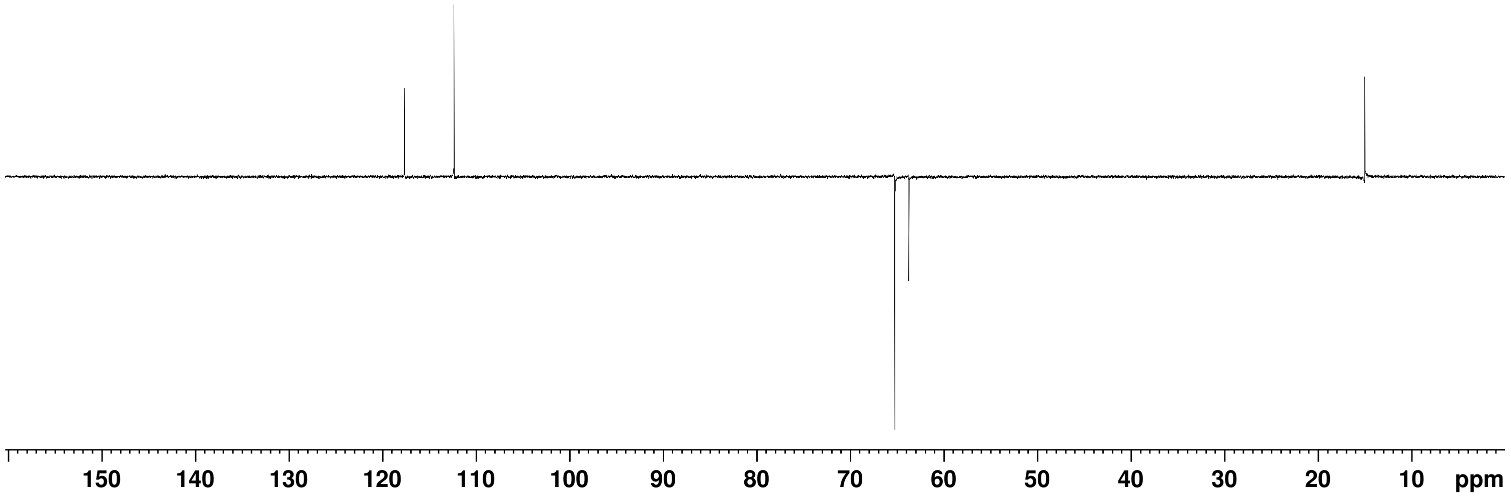

Scheme S31. DEPT-135 NMR spectrum of ethoxybenzenedicarbinol **12b** in $CDCl_3$.

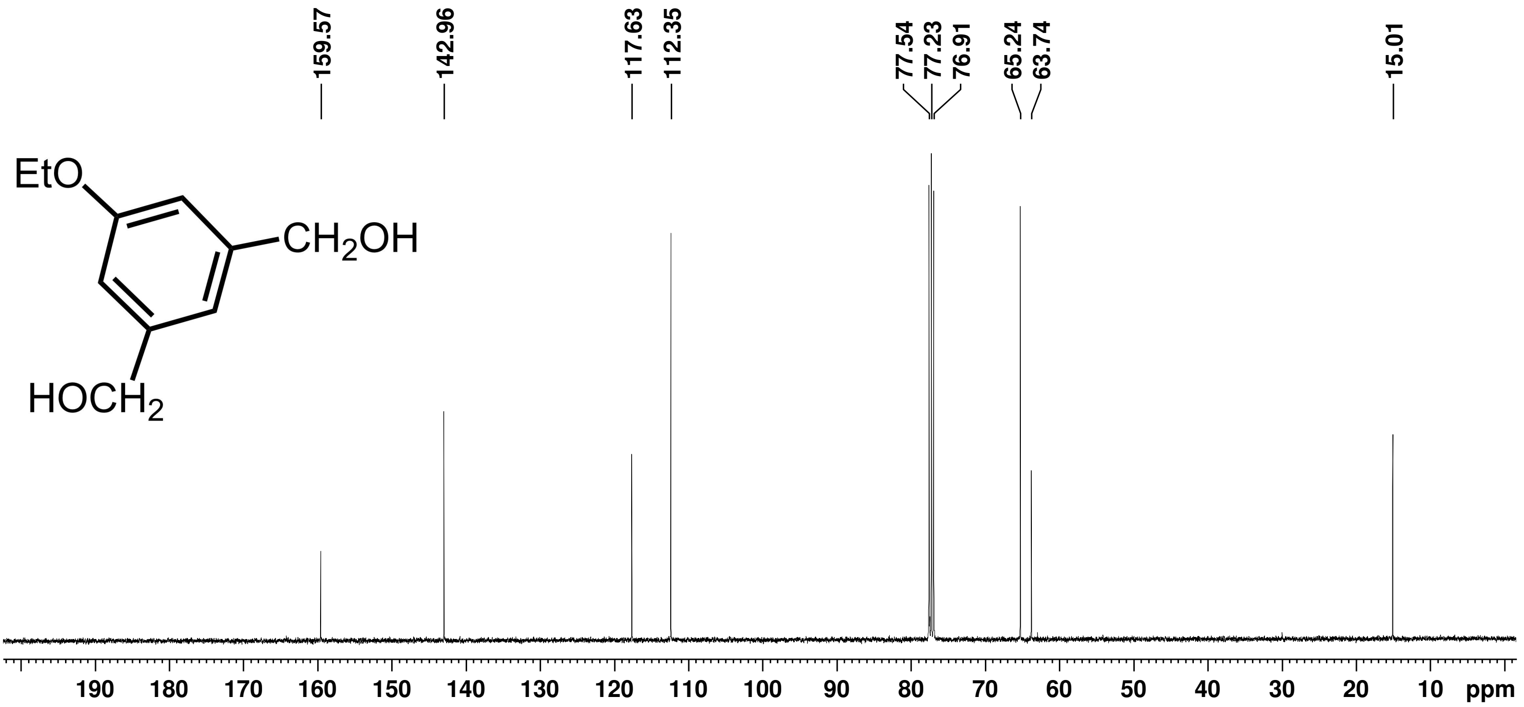

Scheme S32. 100 MHz carbon-13 NMR spectrum of ethoxybenzenedicarbinol **12b** in $CDCl_3$.

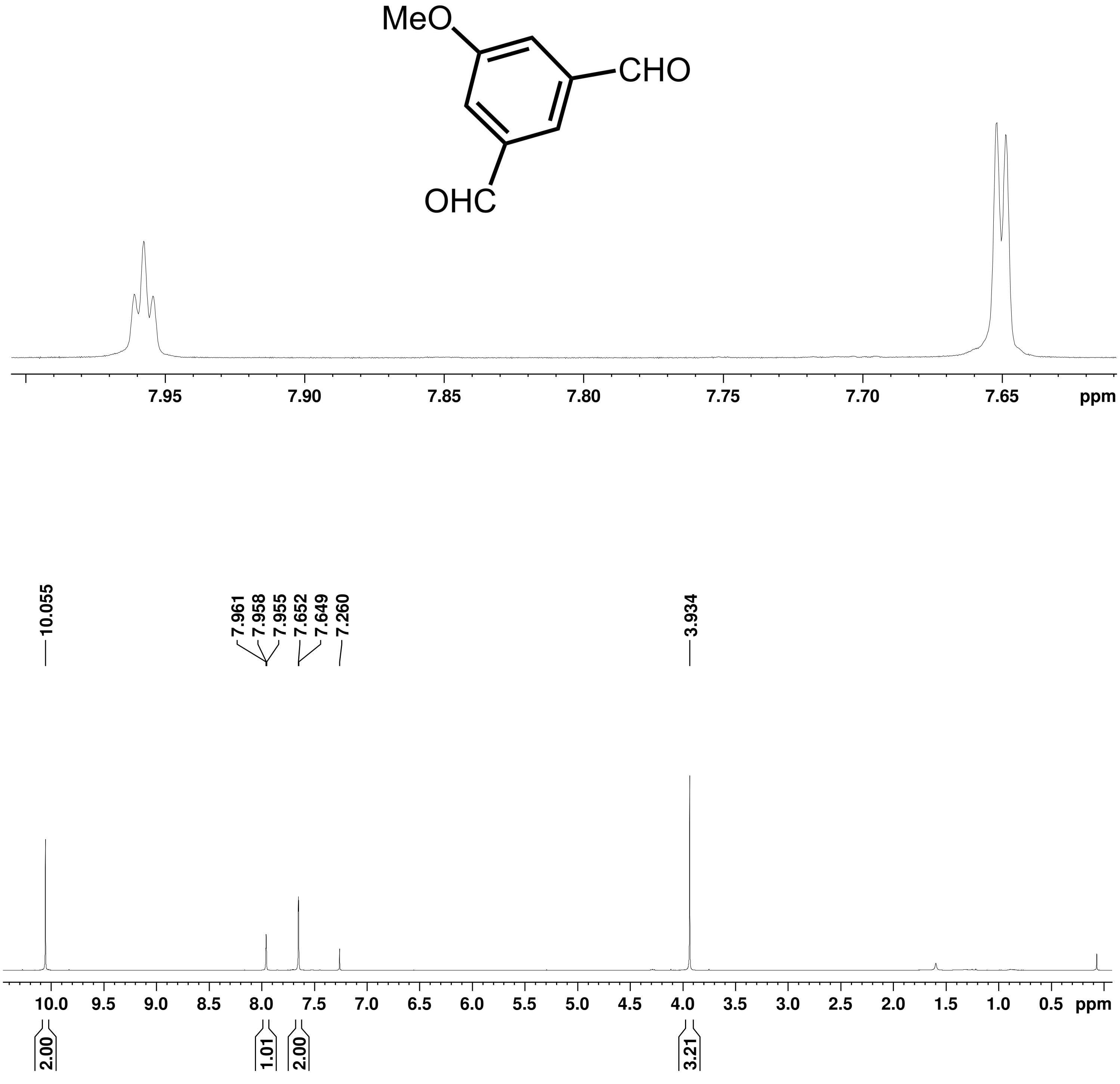

Figure S33. 400 MHz proton NMR spectrum of 5-methoxy-1,3-benzene dicarbaldehyde (**8a**) in $CDCl_3$.

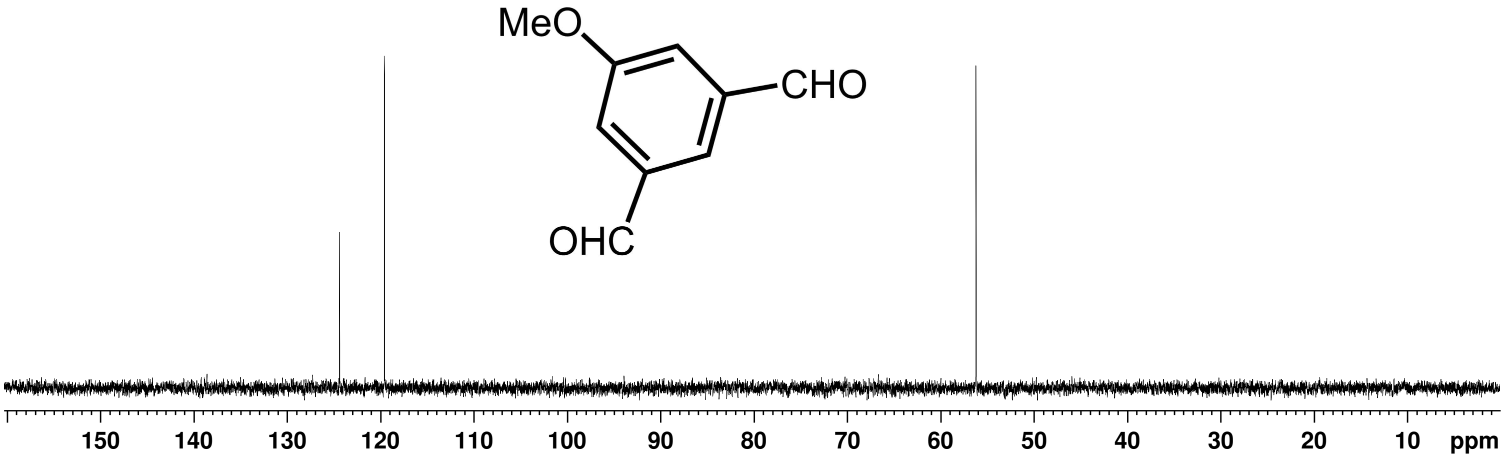

Figure S34. DEPT-135 NMR spectrum of 5-methoxy-1,3-benzene dicarbaldehyde (**8a**) in $CDCl_3$.

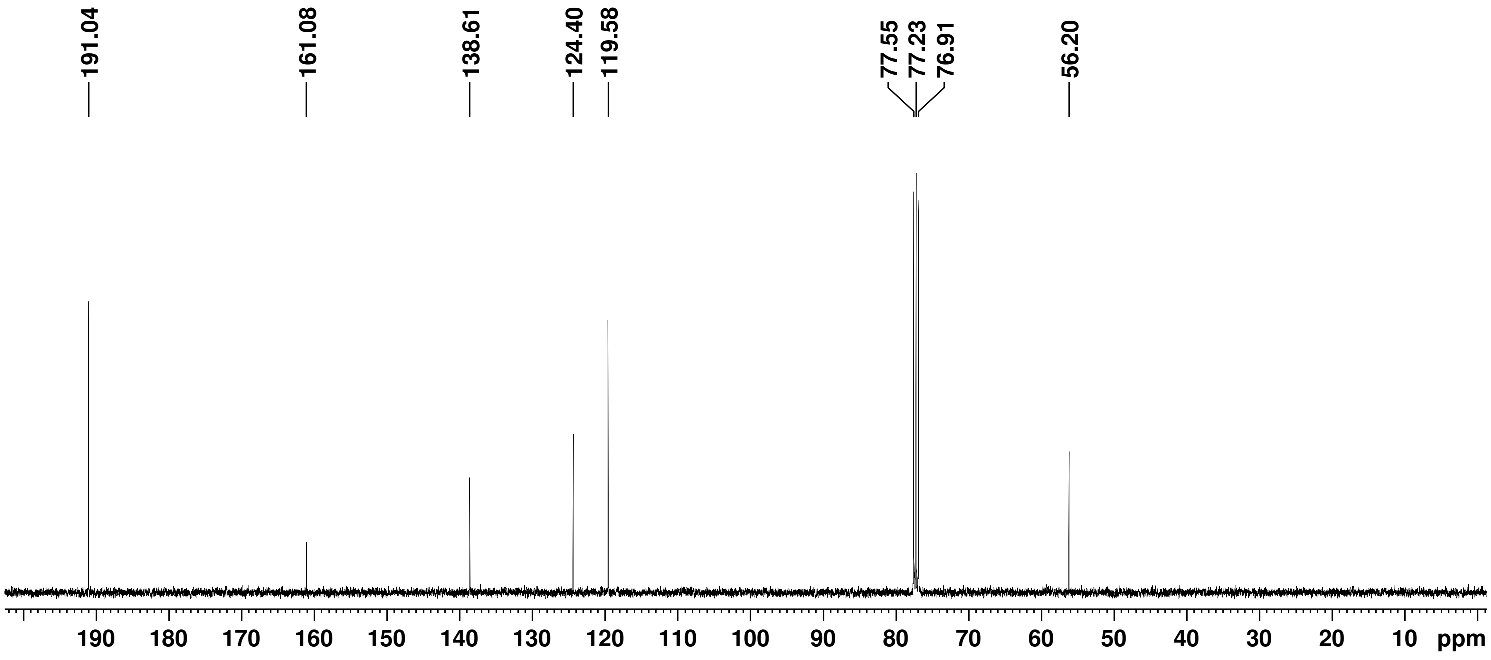

Figure S35. 100 MHz carbon-13 NMR spectrum of 5-methoxy-1,3-benzene dicarbaldehyde (**8a**) in $CDCl_3$.

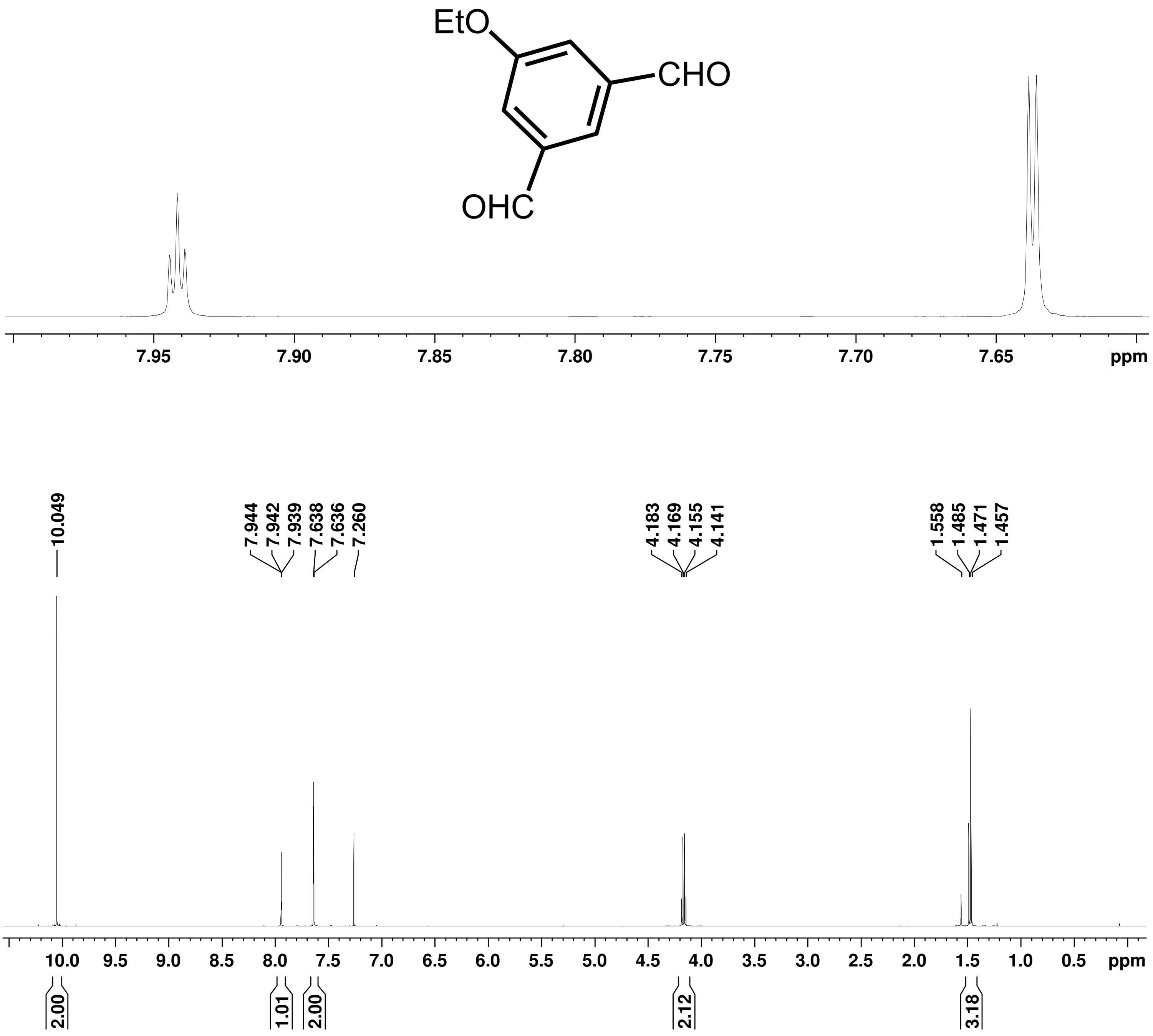

Figure S36. 500 MHz proton NMR spectrum of 5-ethoxy-1,3-benzene dicarbaldehyde (**8b)** in $CDCl_3$.

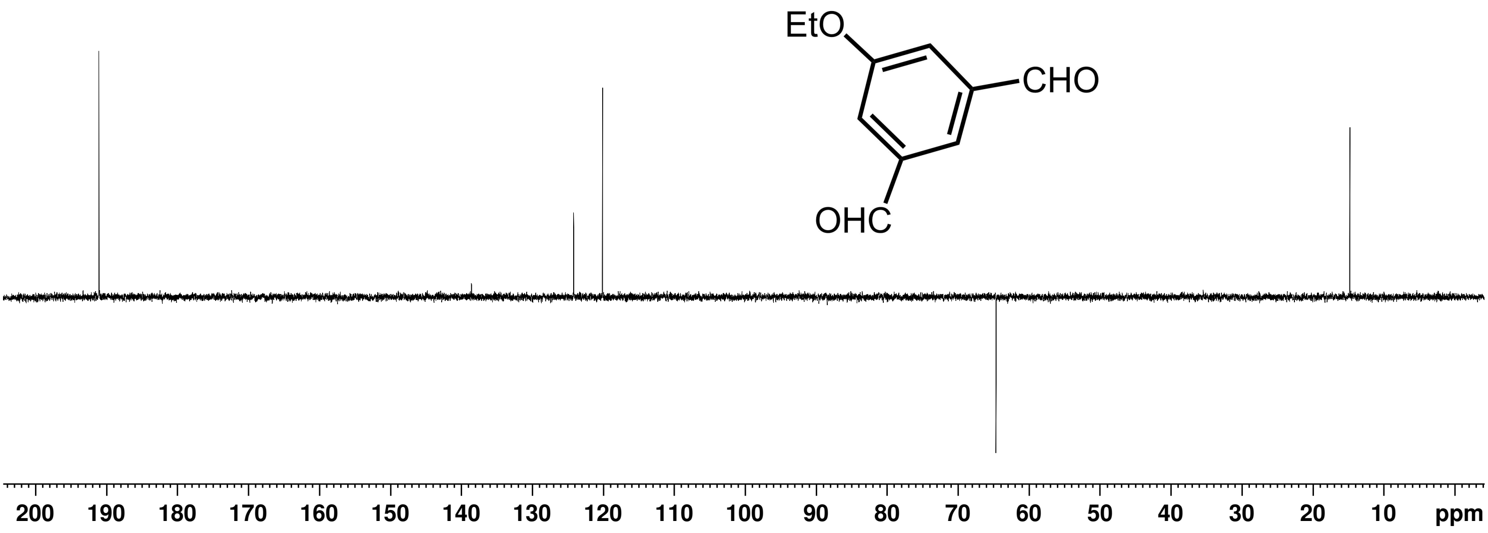

Figure S37. DEPT-135 NMR spectrum of 5-ethoxy-1,3-benzene dicarbaldehyde (**8b**) in $CDCl_3$.

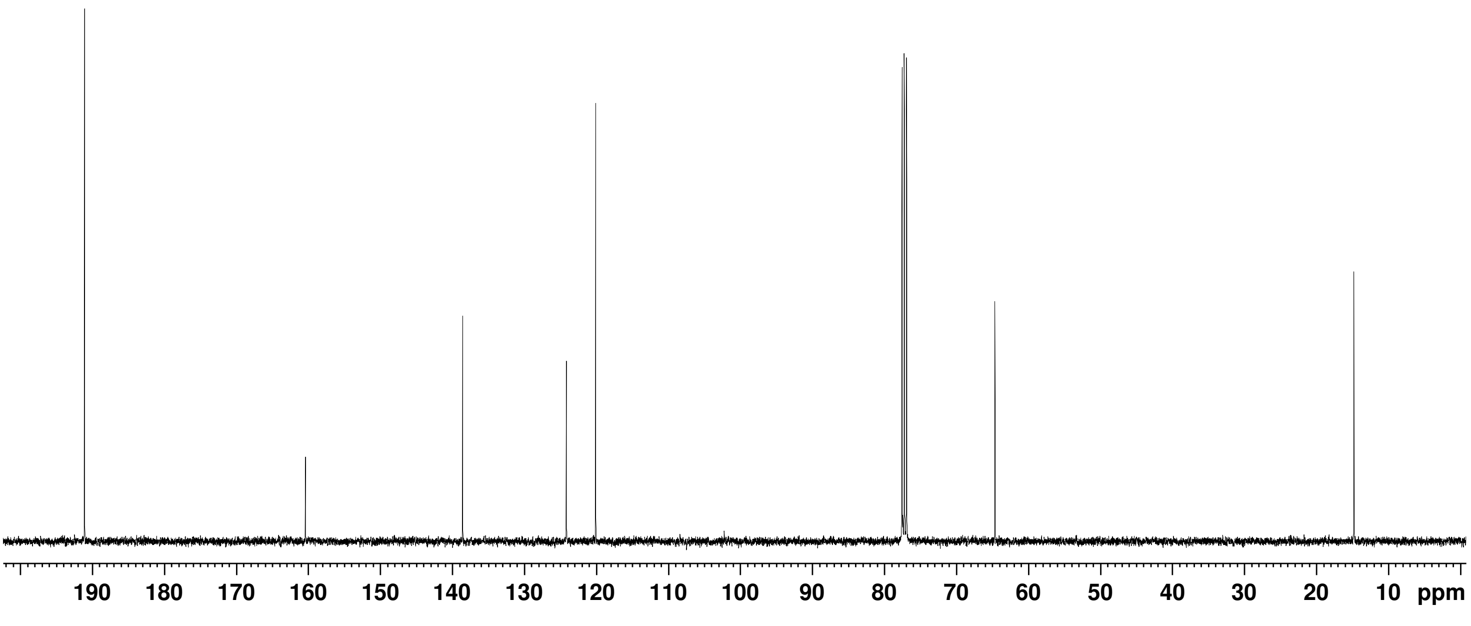

Figure S38. 125 MHz carbon-13 NMR spectrum of 5-ethoxy-1,3-benzene dicarbaldehyde (**8b)** in $CDCl_3$.

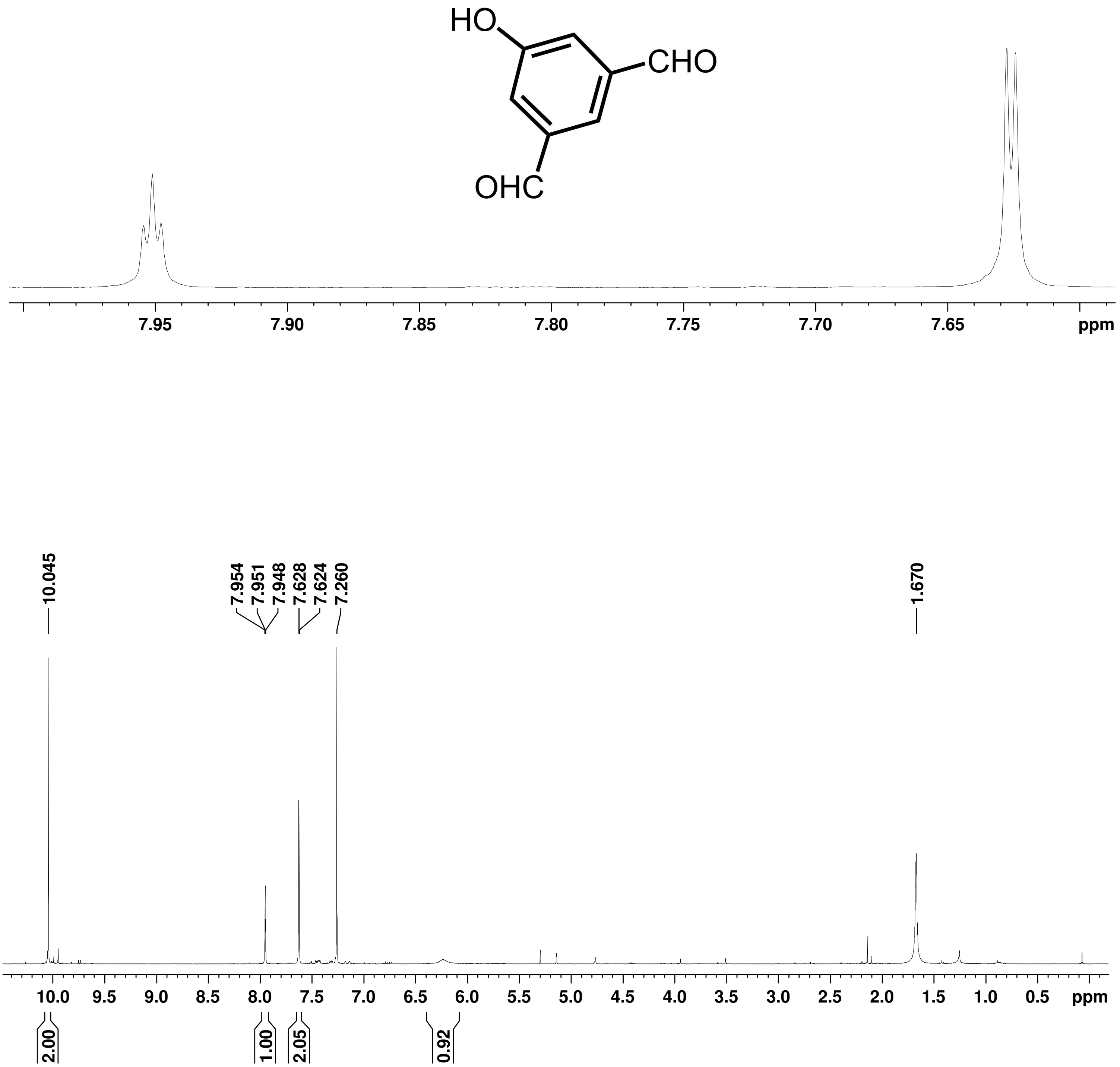

Figure S39. 400 MHz proton NMR spectrum of 5-hydroxy-1,3-benzene dicarbaldehyde (**14**) in $CDCl_3$.

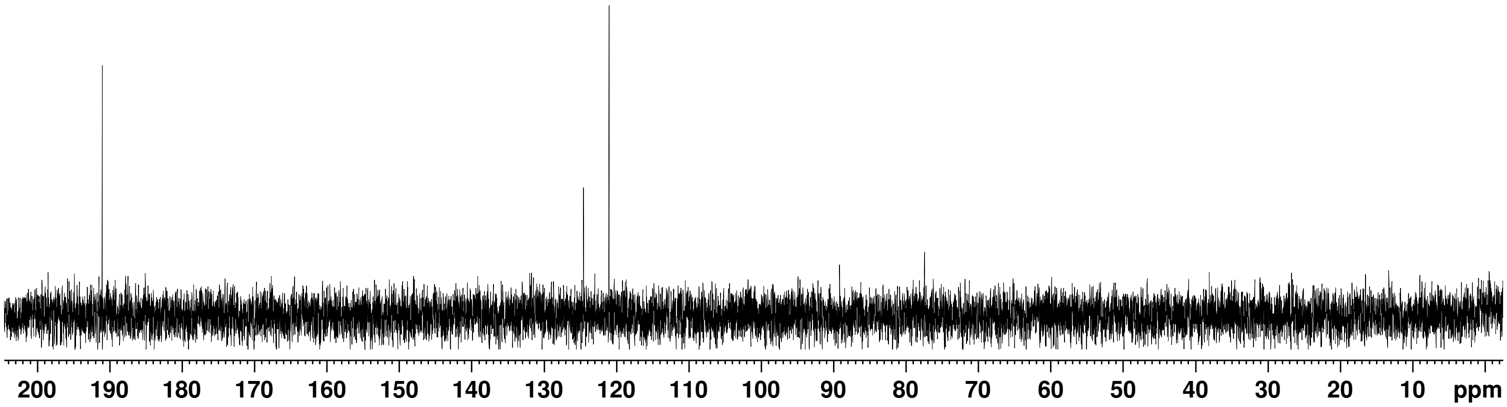

Figure S40. DEPT-135 NMR spectrum of 5-hydroxy-1,3-benzene dicarbaldehyde (**14**) in $CDCl_3$.

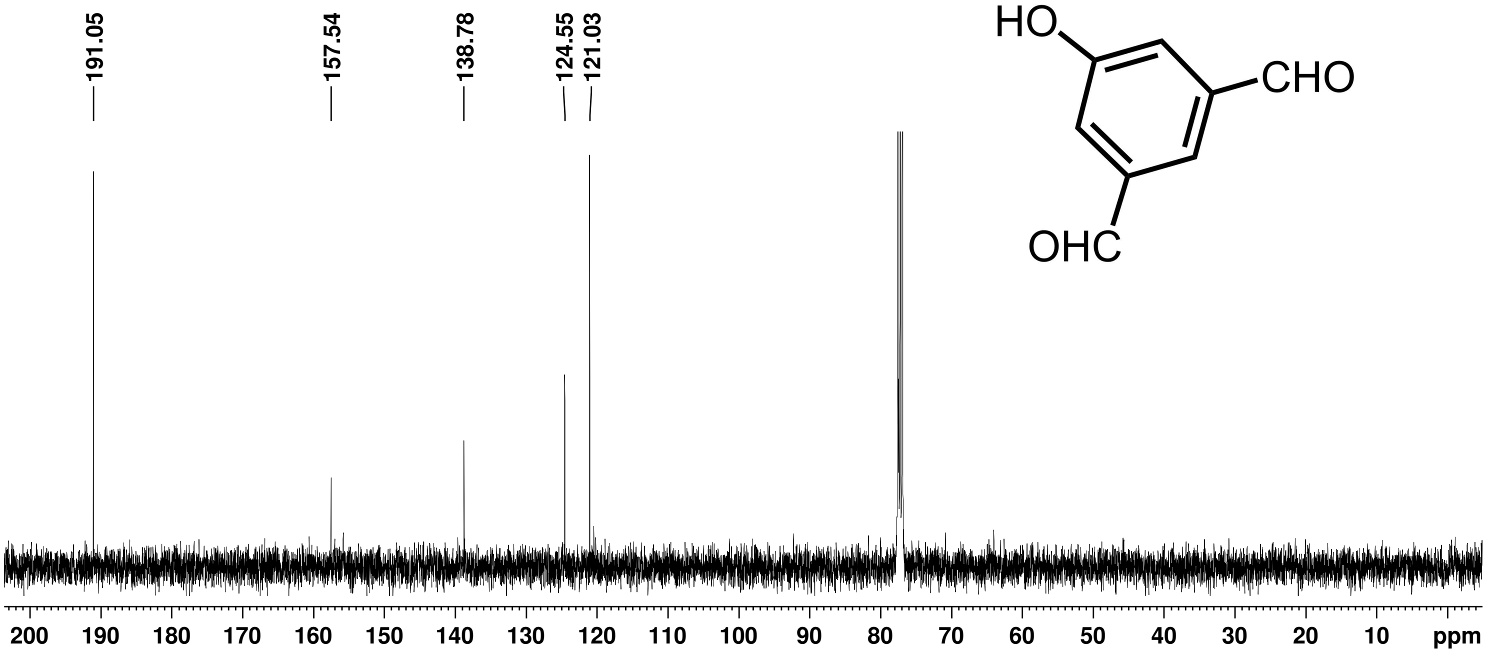

Figure S41. 100 MHz carbon-13 NMR spectrum of 5-hydroxy-1,3-benzene dicarbaldehyde (**14**) in $CDCl_3$.

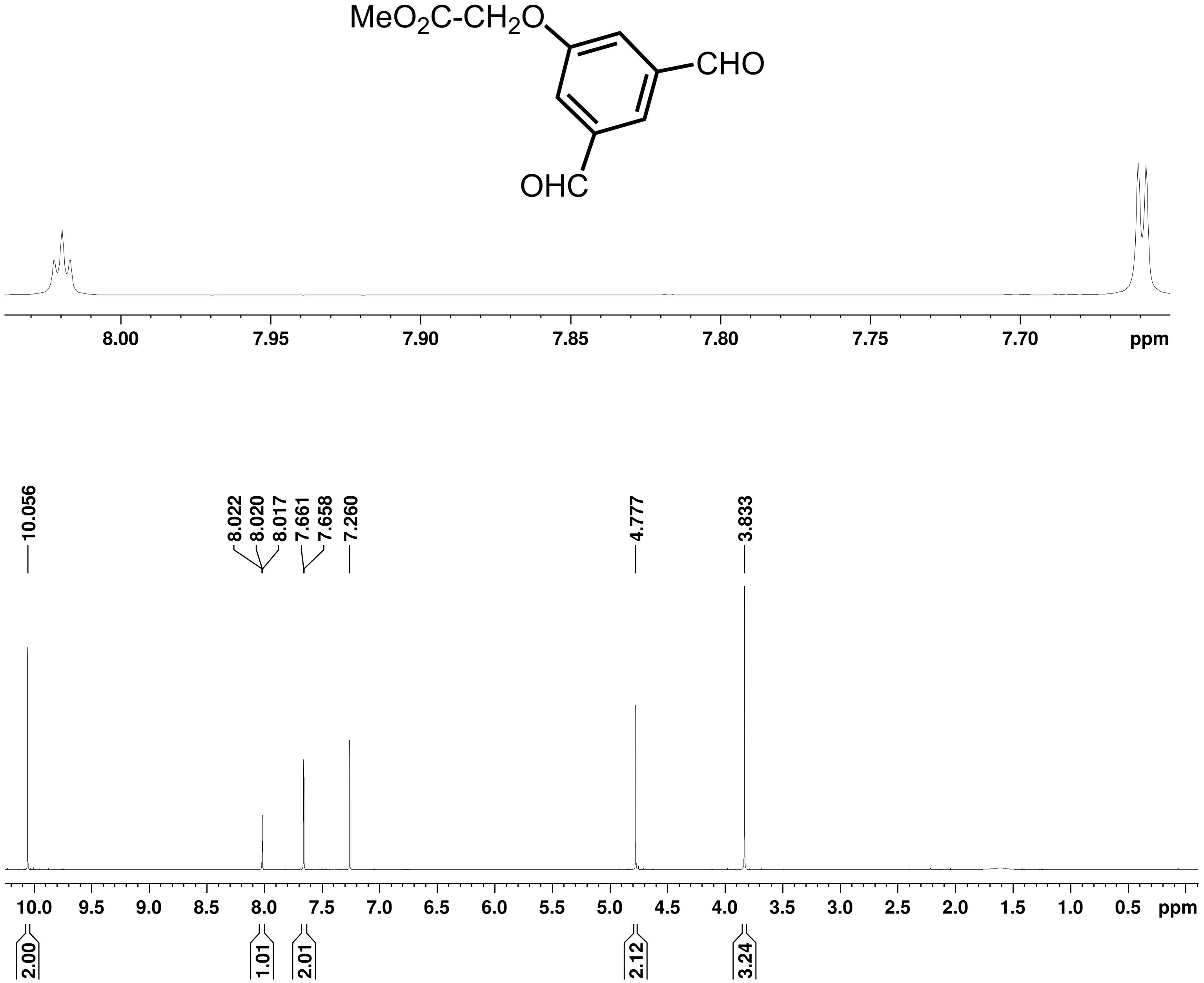

Figure S42. 500 MHz proton NMR spectrum of 5(methoxycarbonylmethyl)-1,3-benzene dicarbaldehyde (**8c**) in $CDCl_3$.

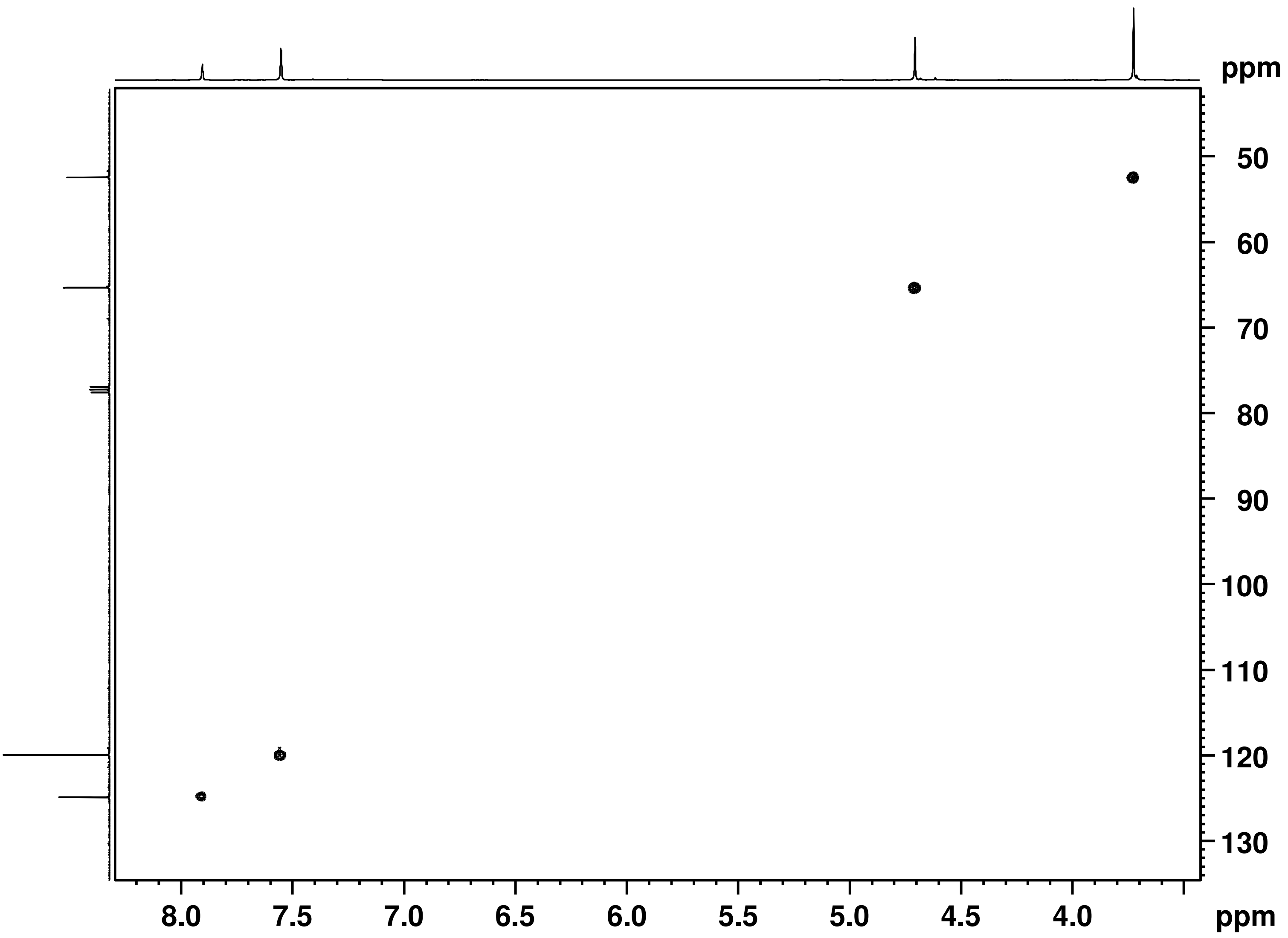

Figure S43. HSQC NMR spectrum of **8c** in $CDCl_3$.

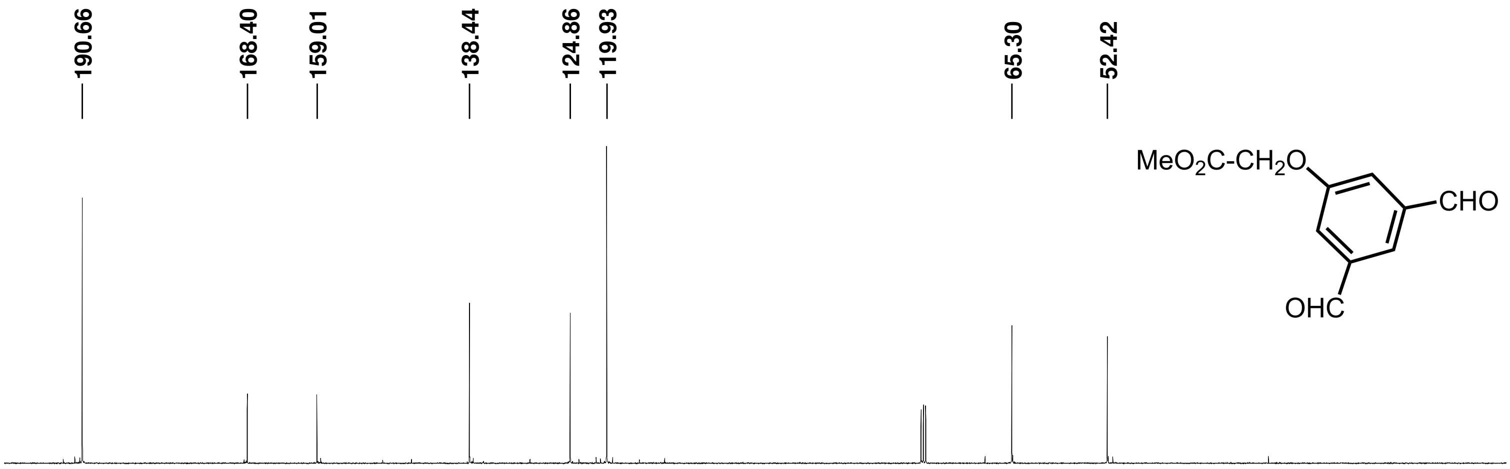

Figure S44. 100 MHz carbon-13 NMR spectrum of **8c** in $CDCl_3$.

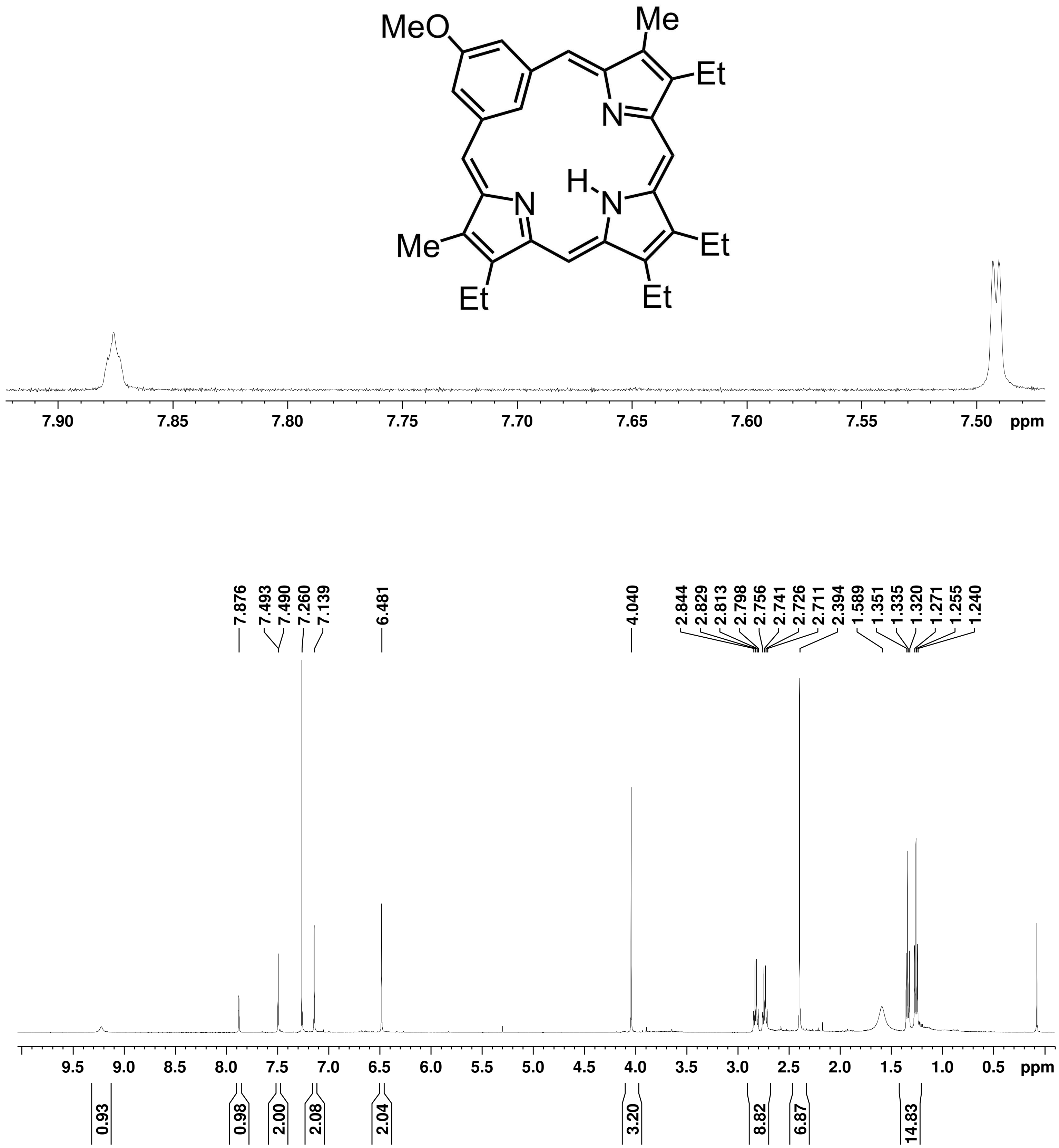

Figure S45. 500 MHz proton NMR spectrum of methoxybenziporphyrin **7a** in $CDCl_3$.

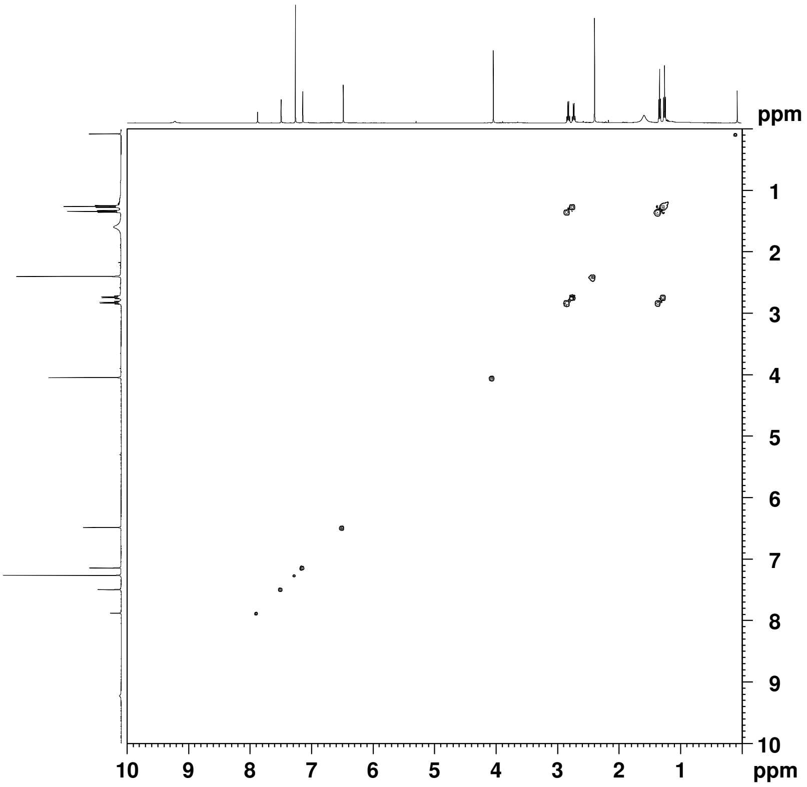

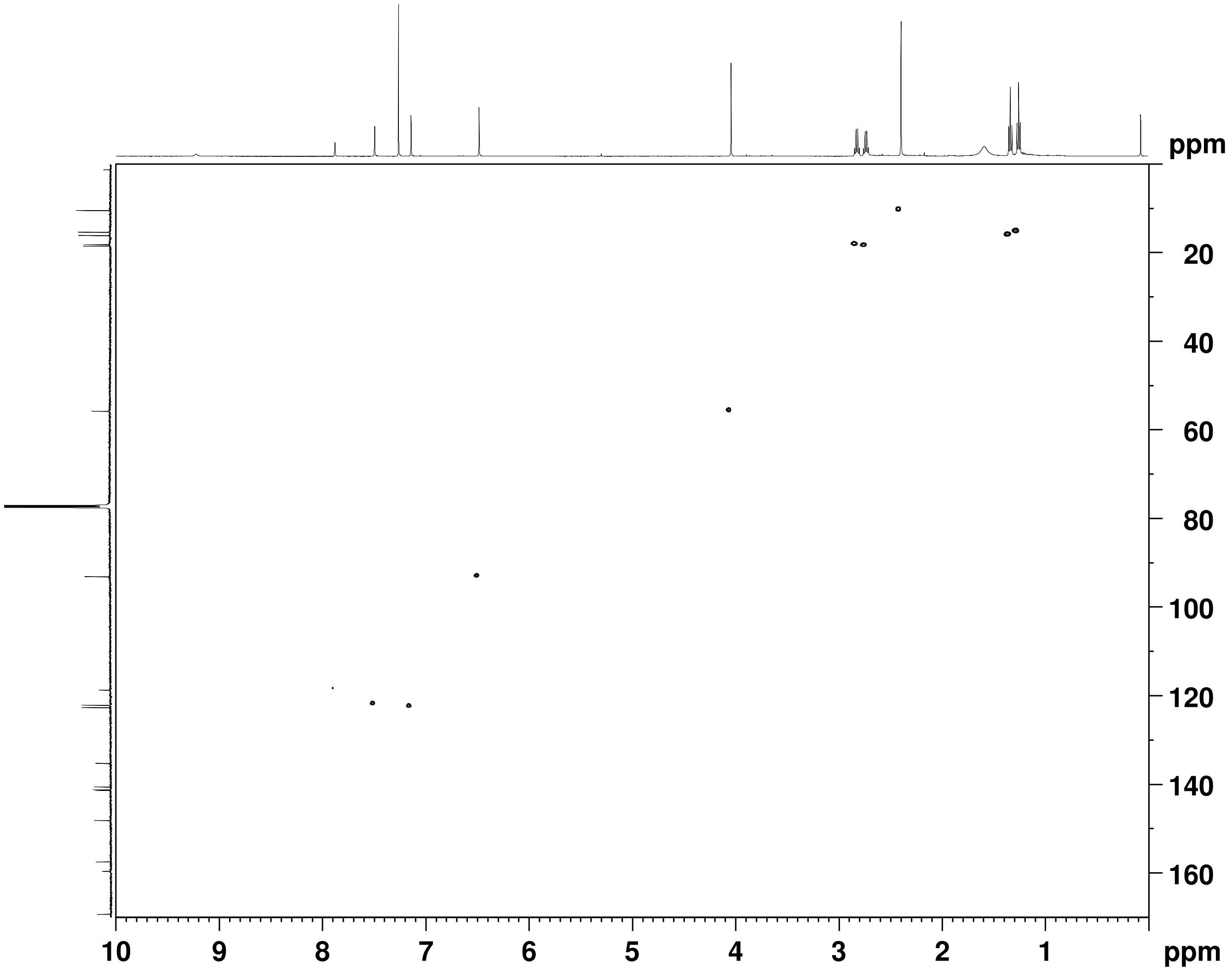

Figure S46. ^{1}H-^{1}H COSY (top) and HSQC (bottom) NMR spectra of methoxybenziporphyrin **7a** in $CDCl_3$.

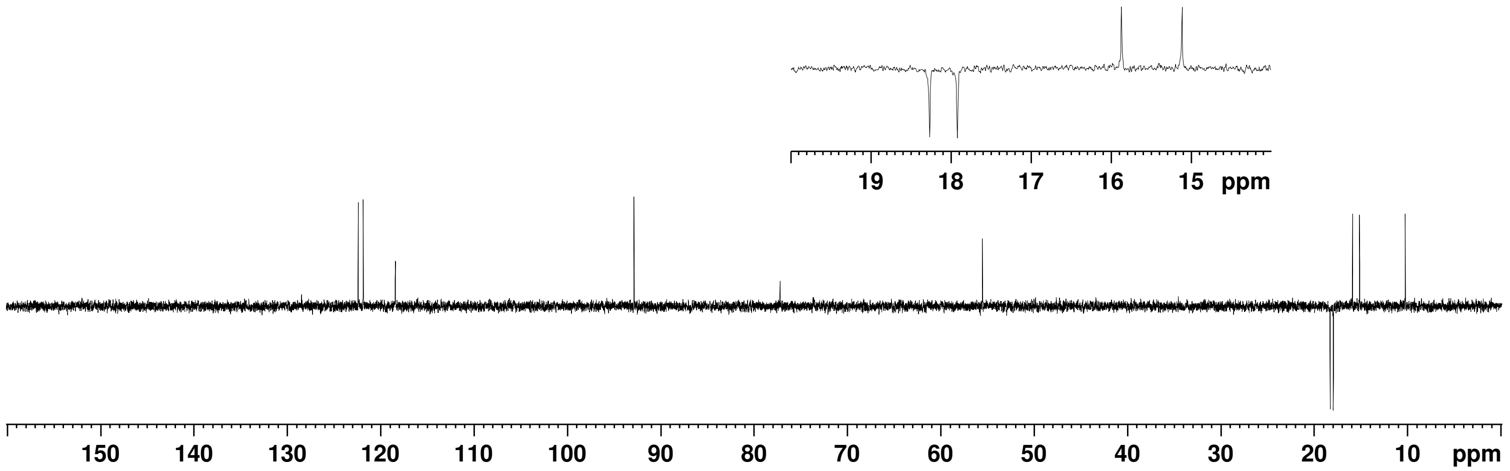

Figure S47. DEPT-135 NMR spectrum of **7a** in $CDCl_3$.

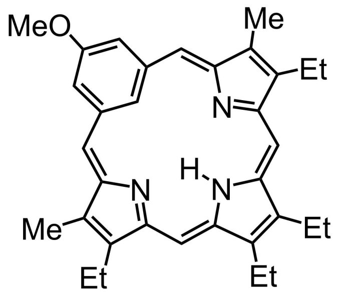

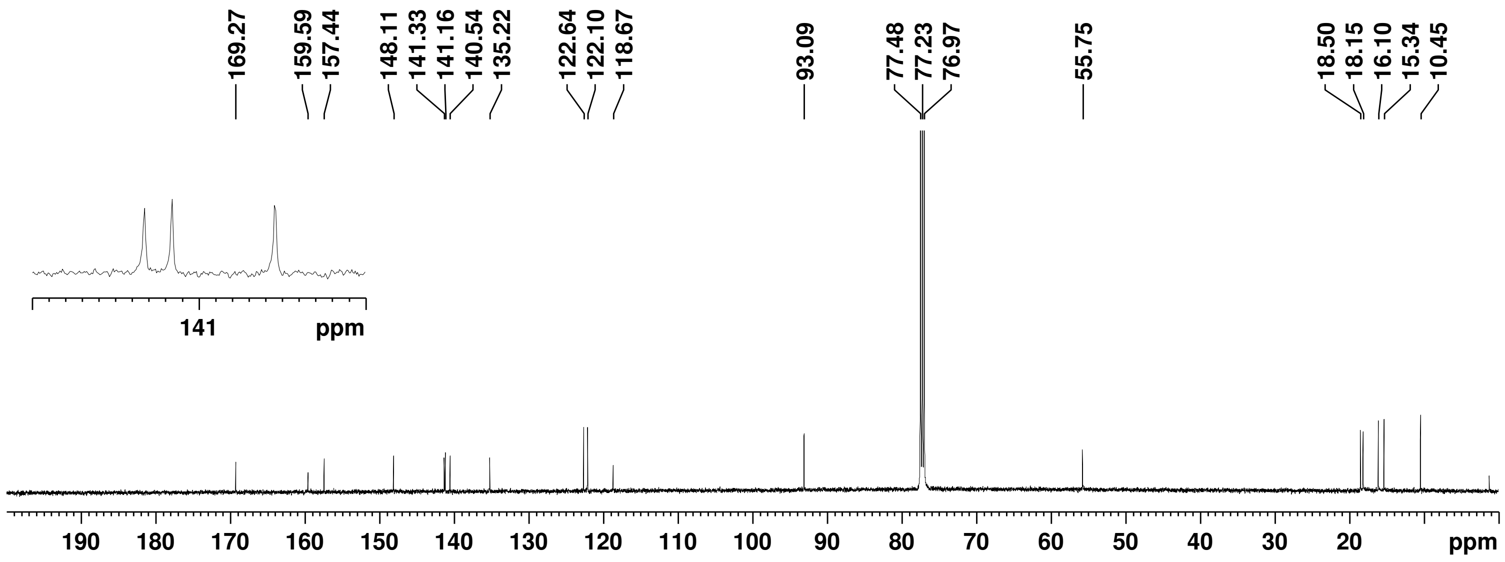

Figure S48. 125 MHz carbon-13 NMR spectrum of methoxybenziporphyrin **7a** in $CDCl_3$.

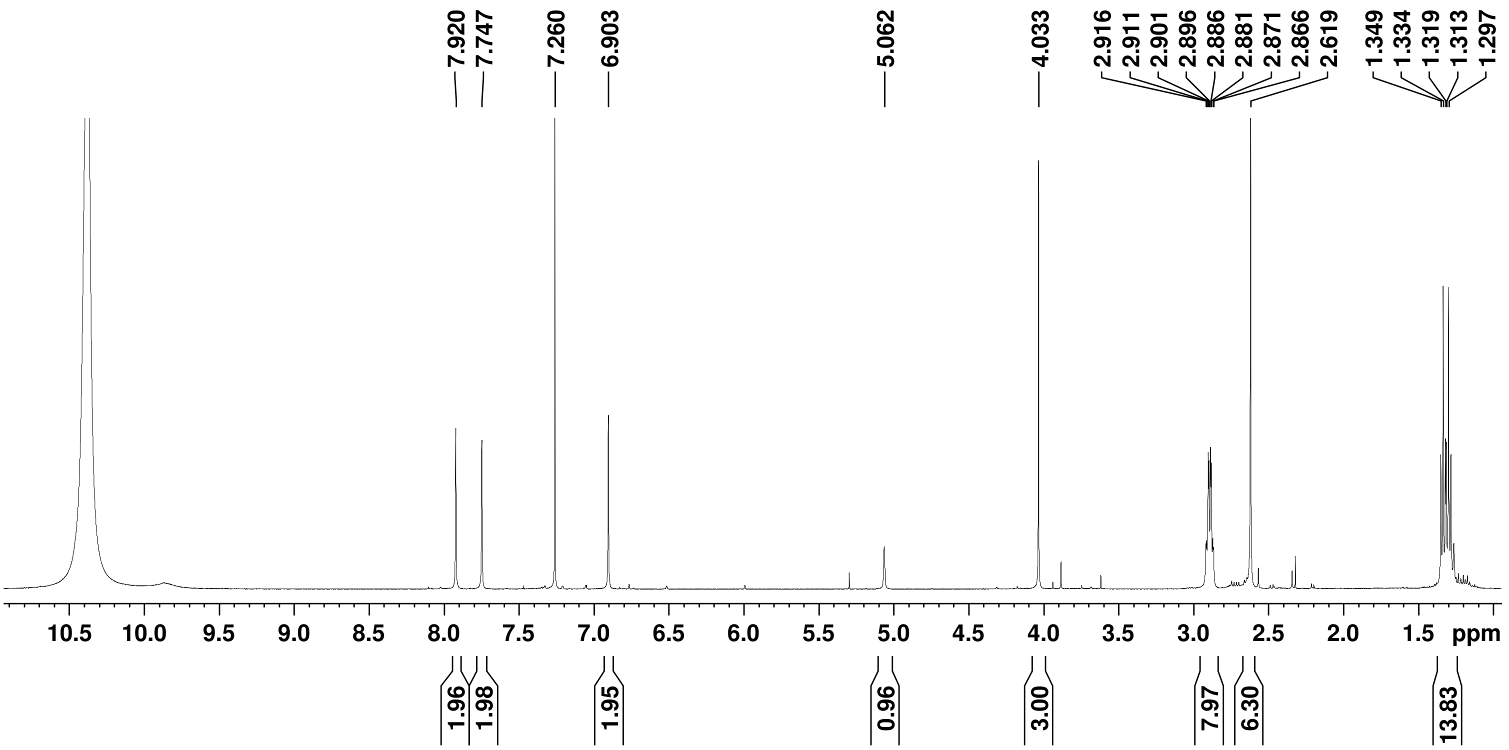

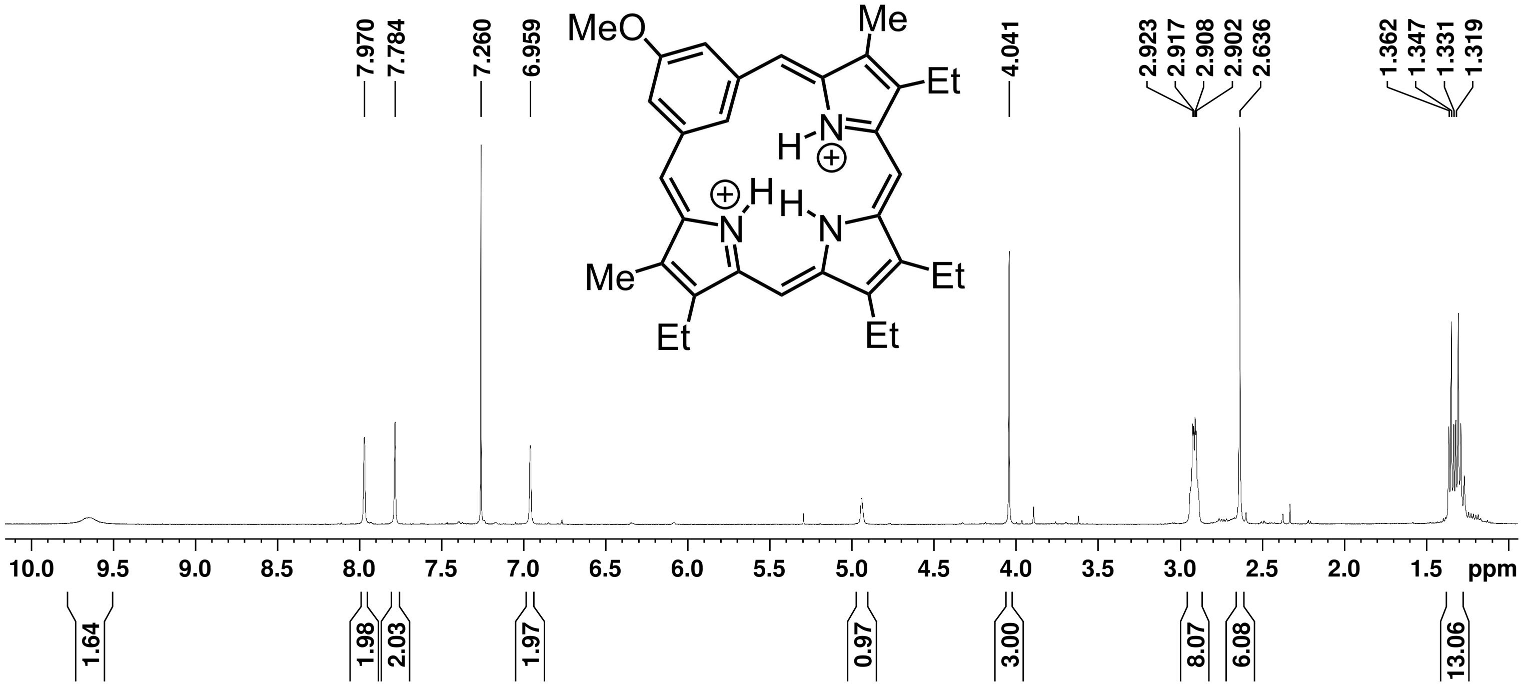

Figure S49. 500 MHz proton NMR spectra of **7a** in the presence of TFA and $CDCl_3$. The second spectrum shows the results from adding several additional drops of TFA.

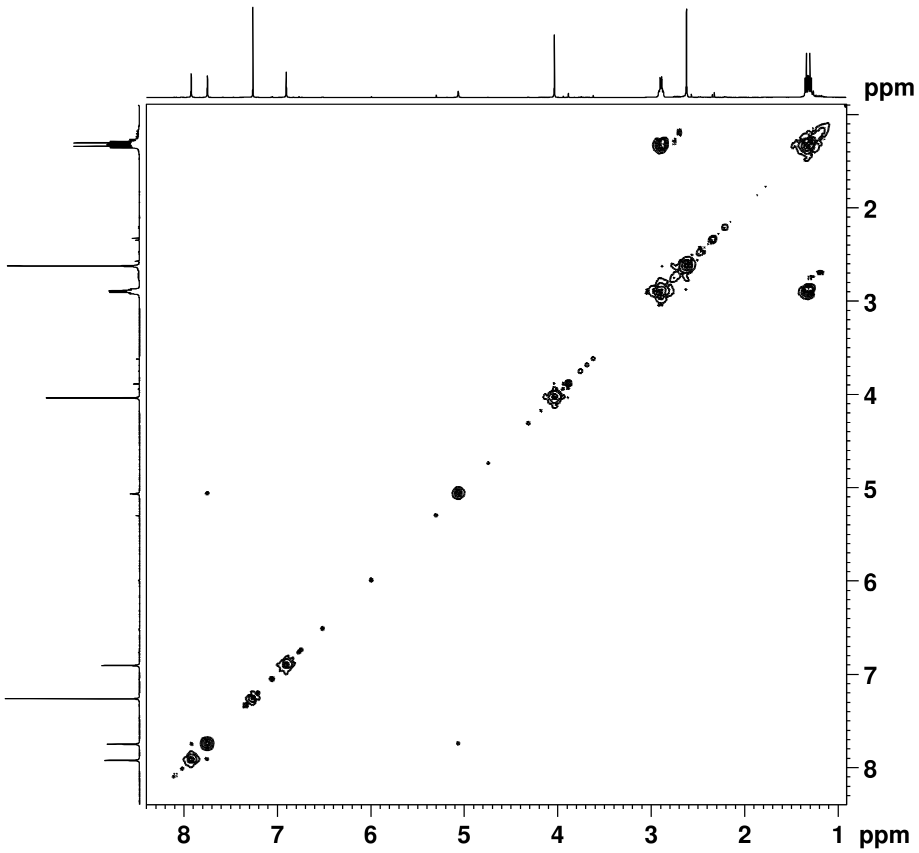

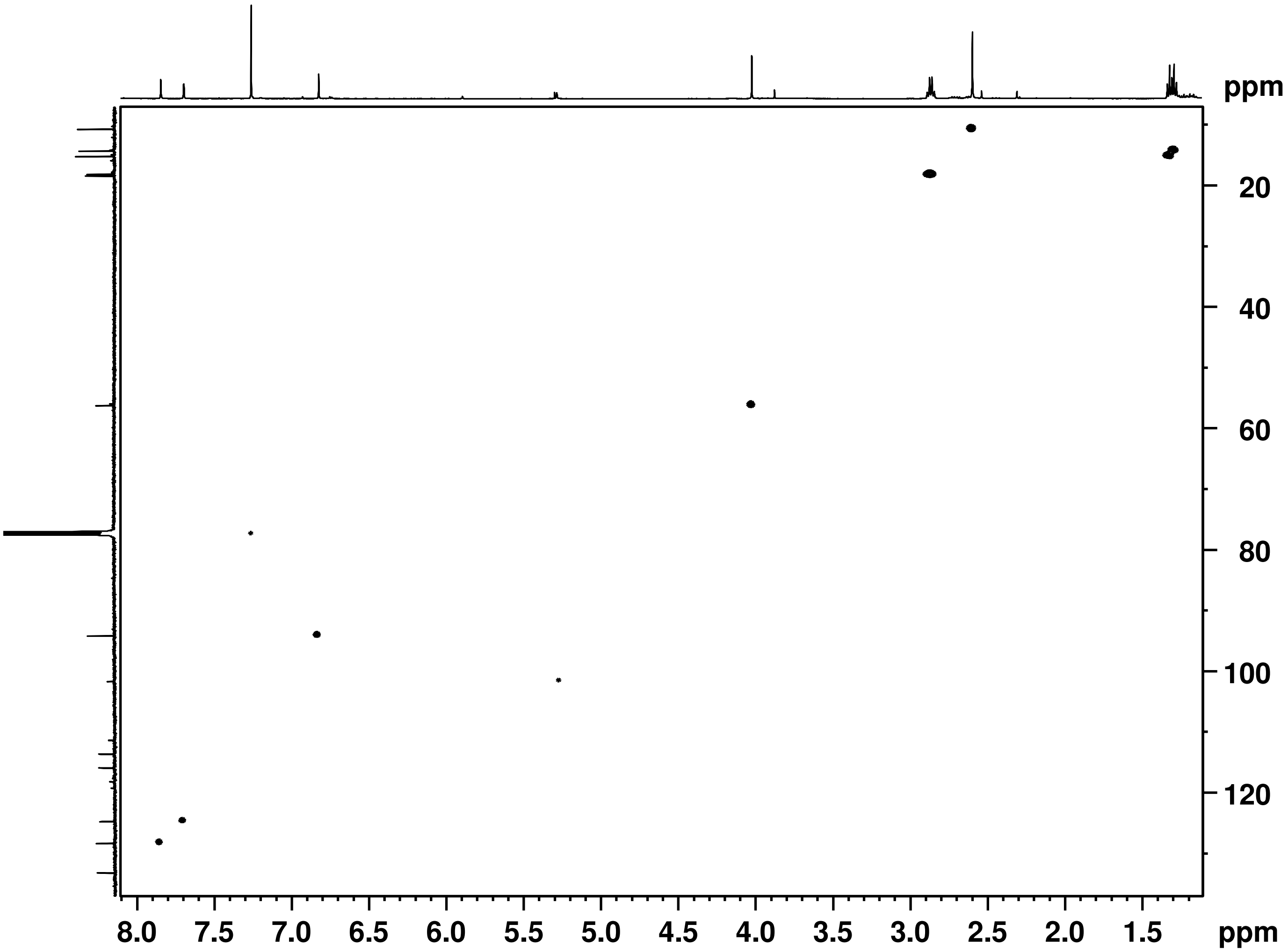

Figure S50. ^{1}H-^{1}H COSY (top) and HSQC (bottom) NMR spectra of **7a**H_2^{2+} in TFA-$CDCl_3$.

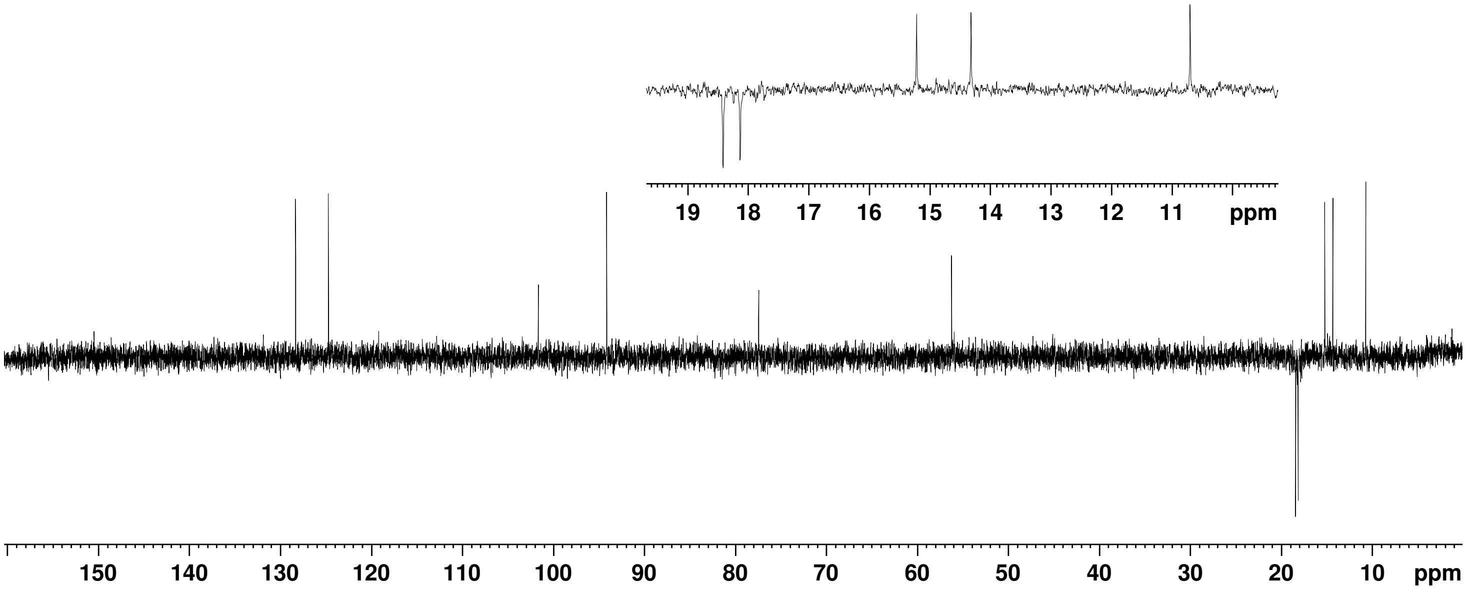

Figure S51. DEPT-135 NMR spectrum of **7a**H_2^{2+} in TFA-$CDCl_3$.

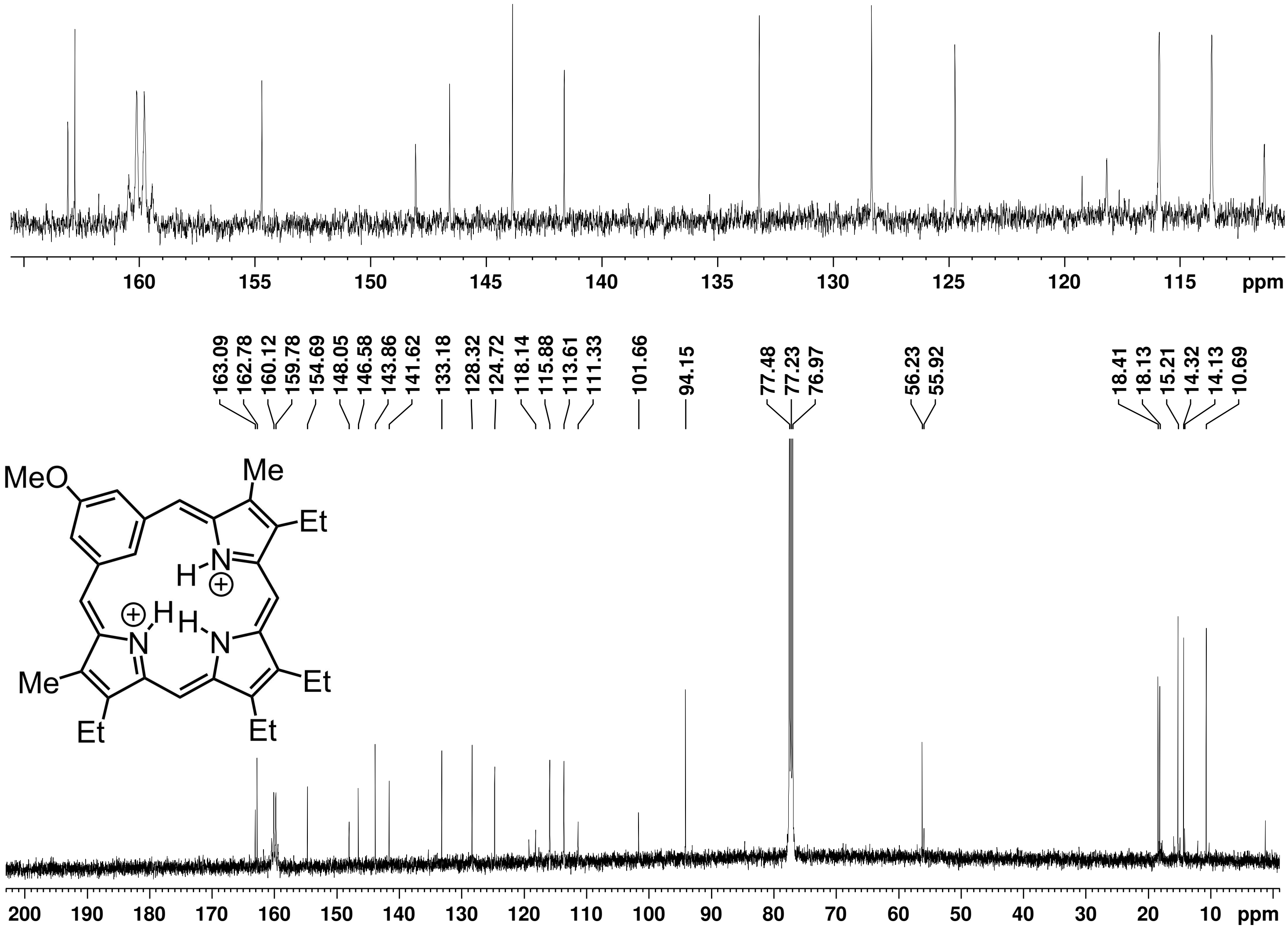

Figure S52. 125 MHz carbon-13 NMR spectrum of **7a**H_2^{2+} in TFA-$CDCl_3$.

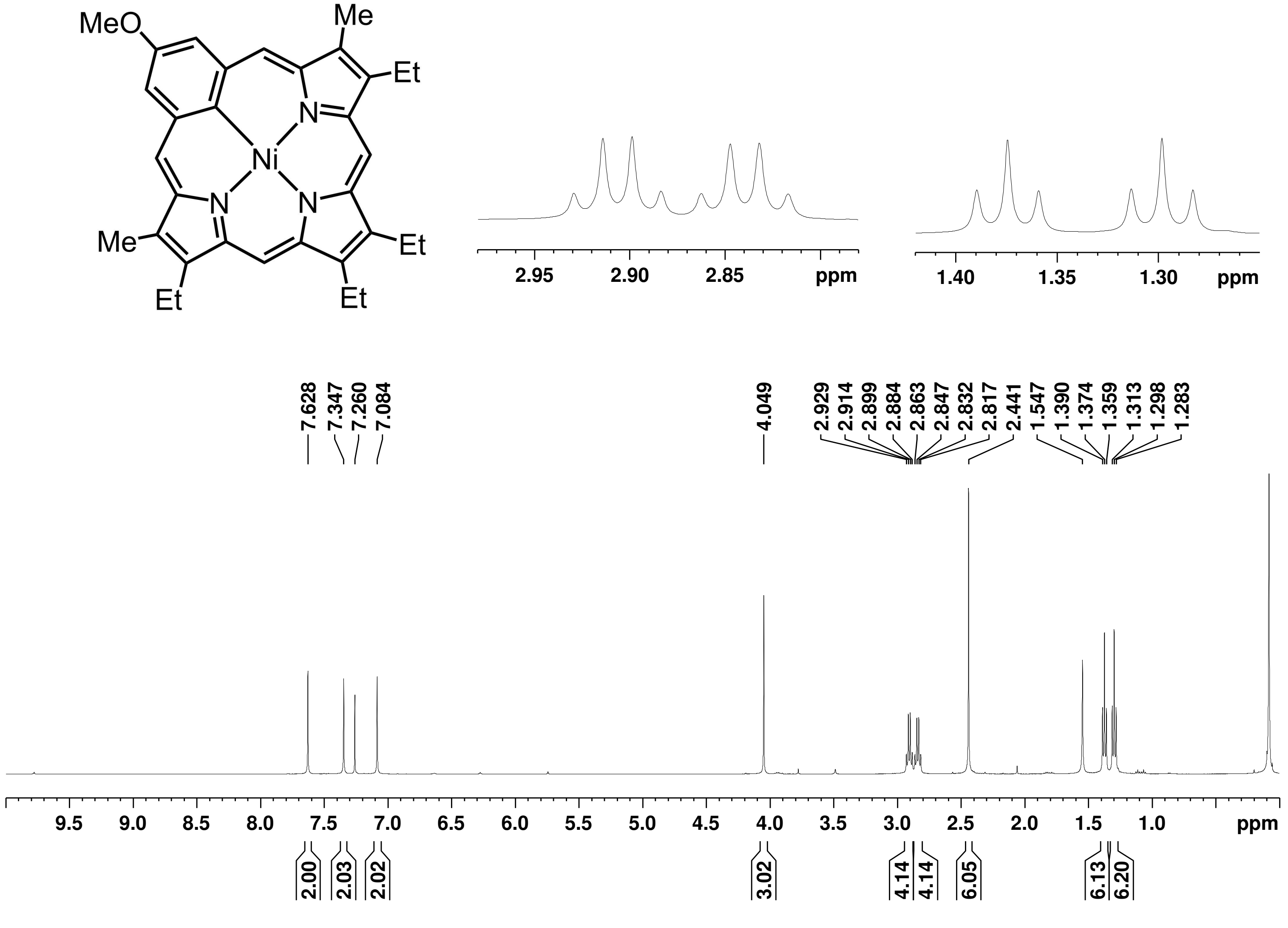

Figure S53, 500 MHz proton NMR spectrum of nickel(II) complex **7aNi i**n $CDCl_3$.

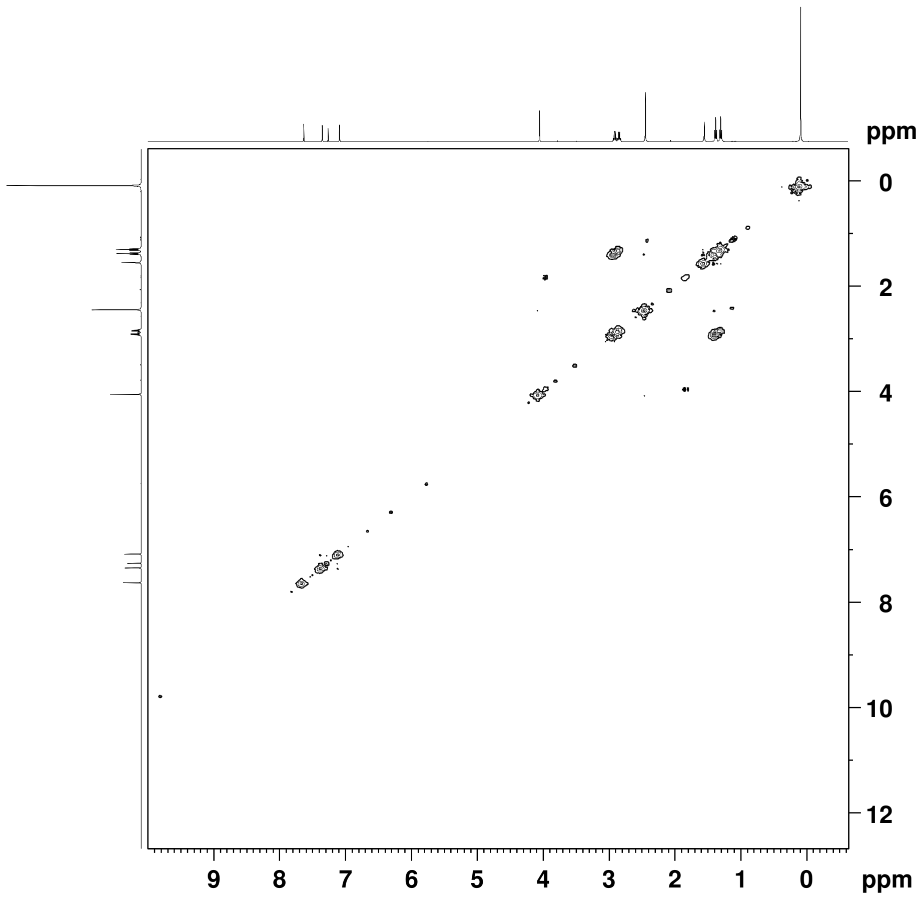

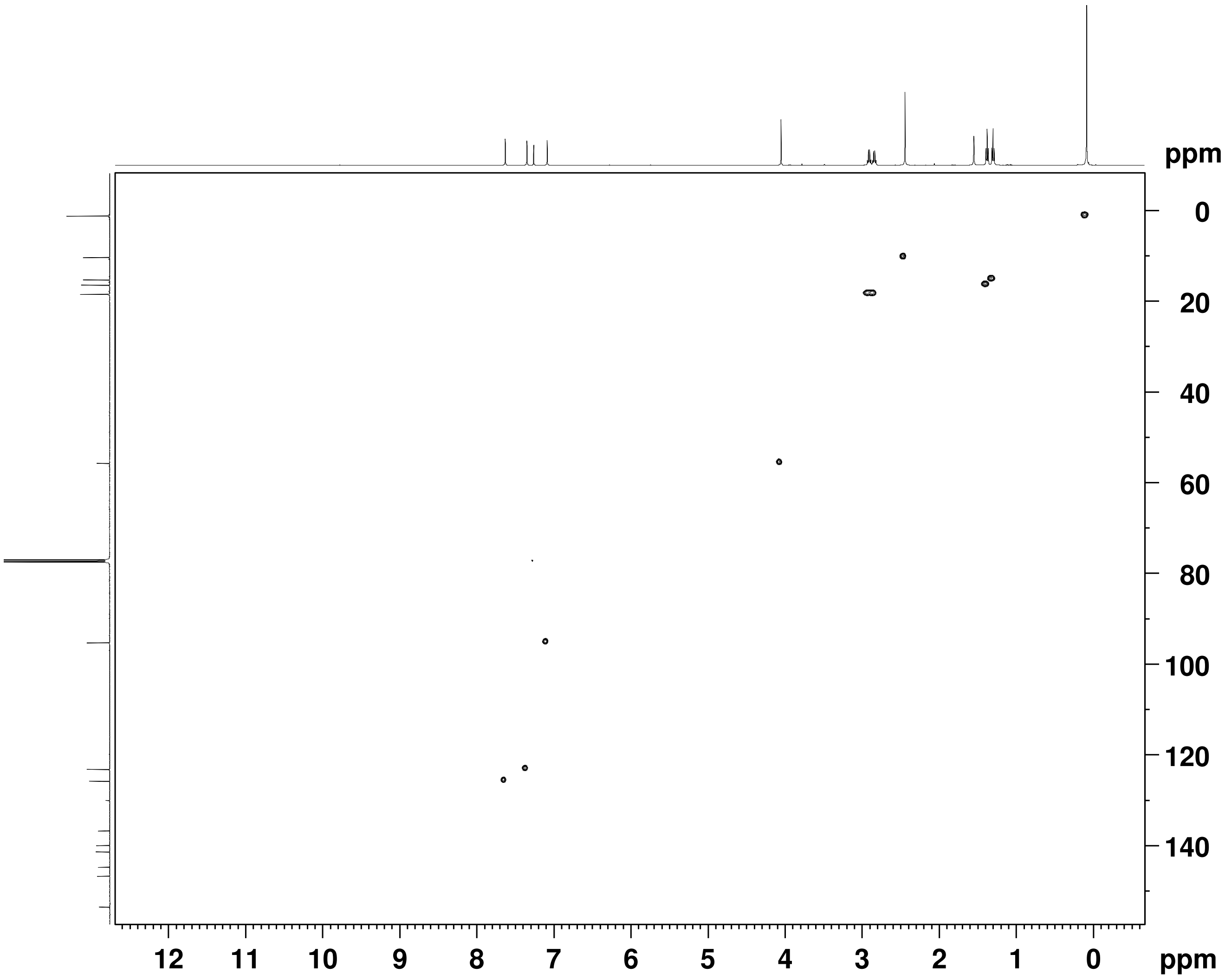

Figure S54. ^{1}H-^{1}H COSY (top) and HSQC (bottom) NMR spectra of **7aNi** in $CDCl_3$.

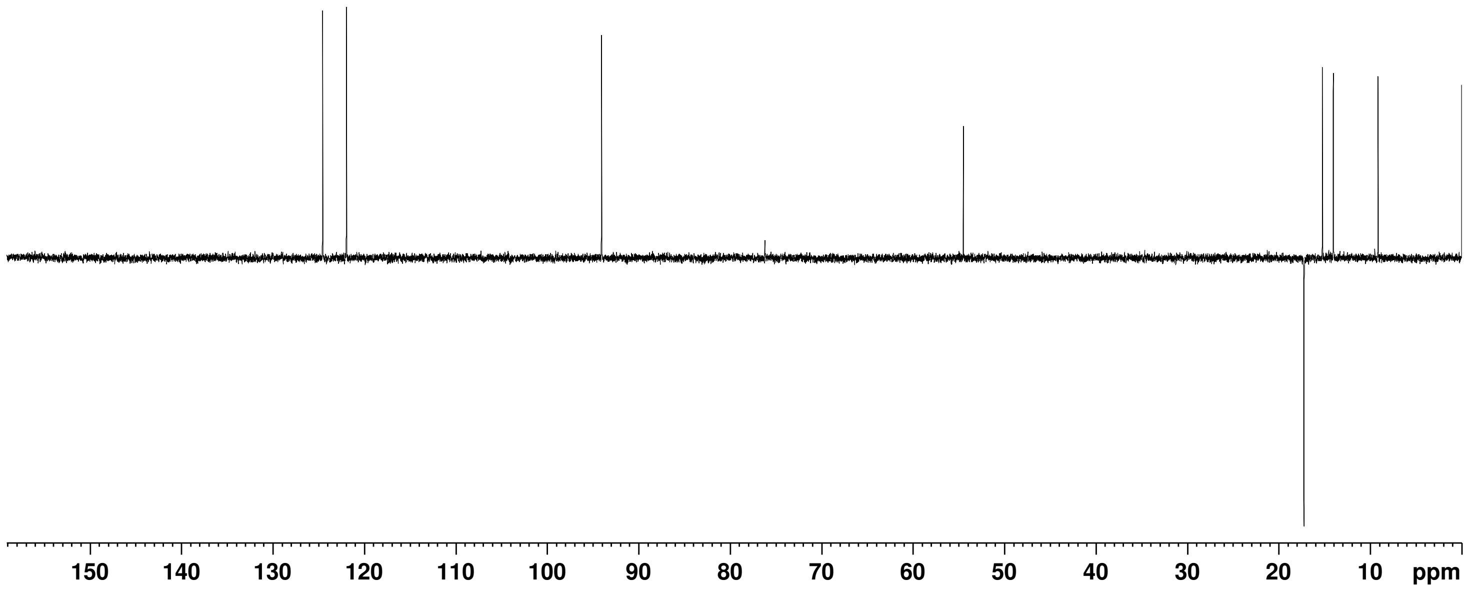

Figure S55. DEPT-135 NMR spectrum of **7aNi** in $CDCl_3$.

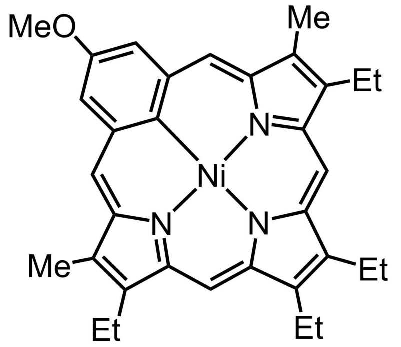

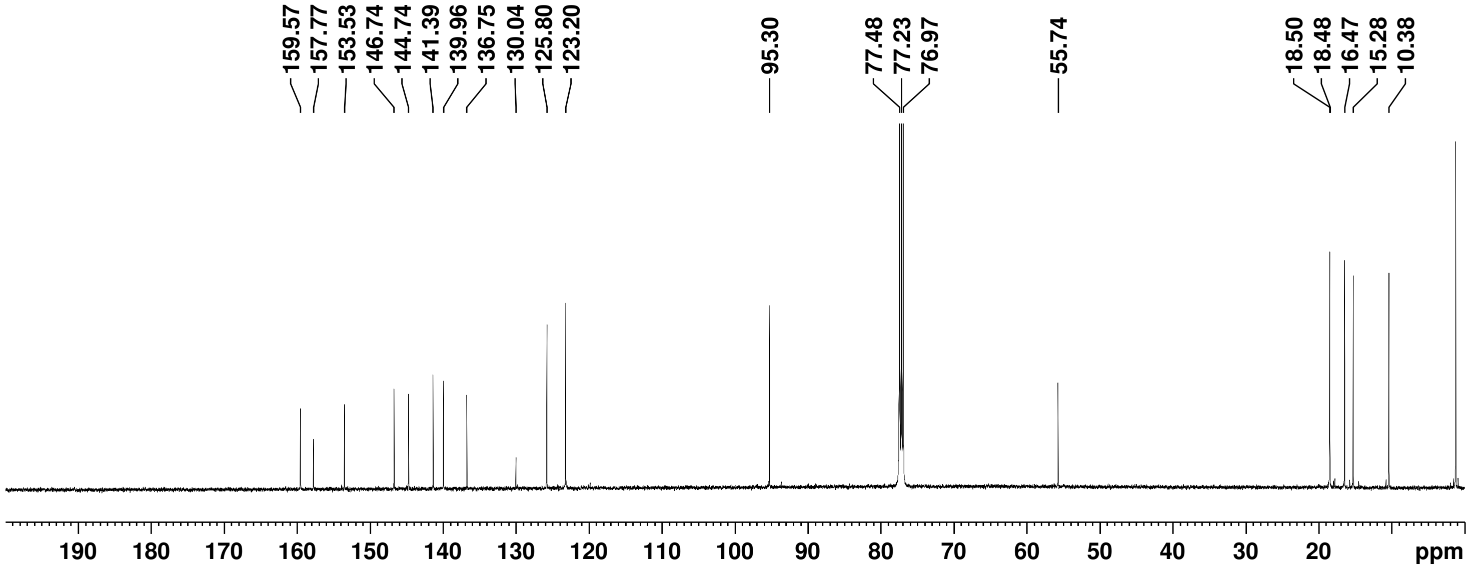

Figure S56. 125 MHz carbon-13 NMR spectrum of **7aNi** in $CDCl_3$.

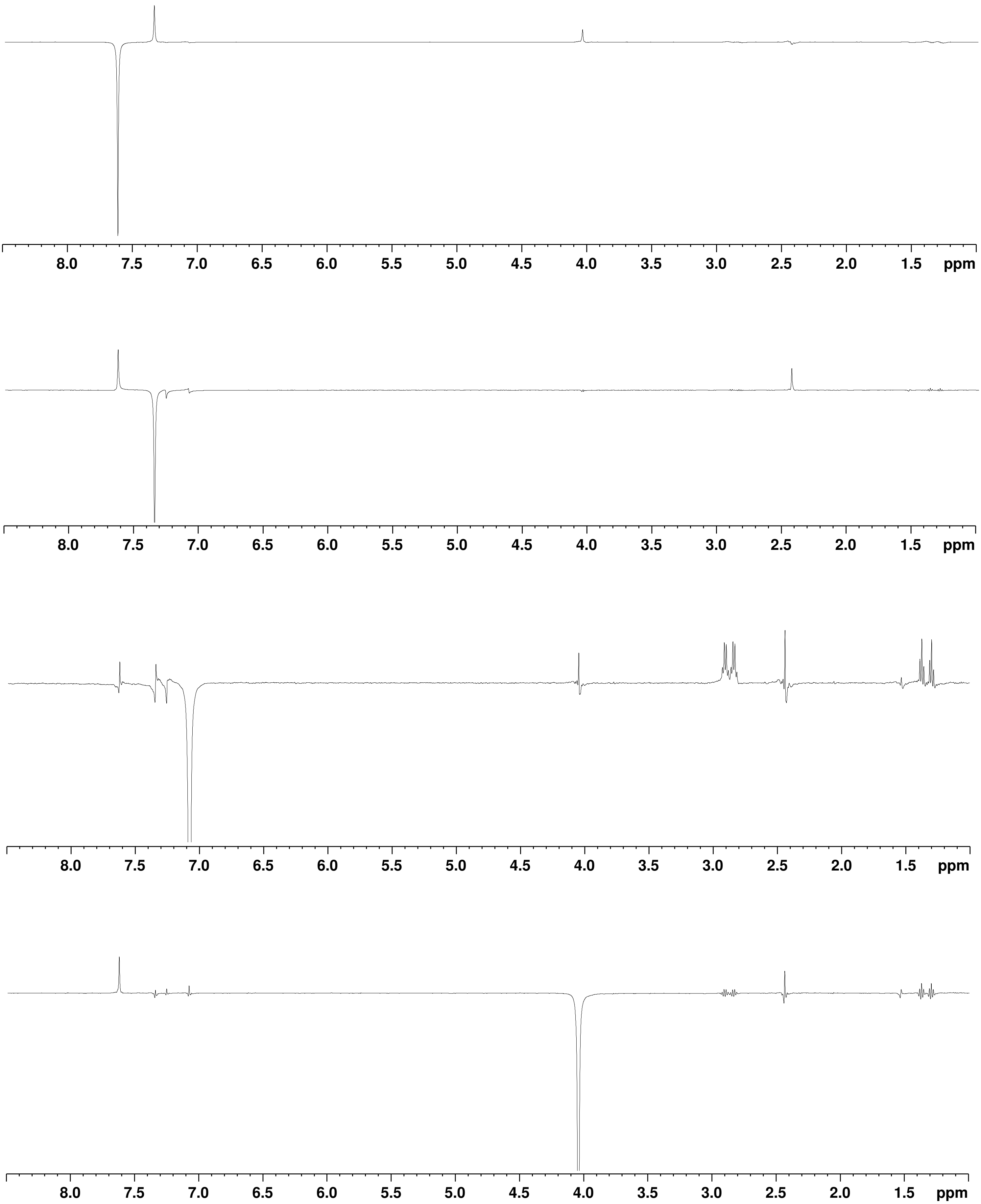

Figure S57. Selected nOe difference proton NMR spectra of **7aNi** in $CDCl_3$.

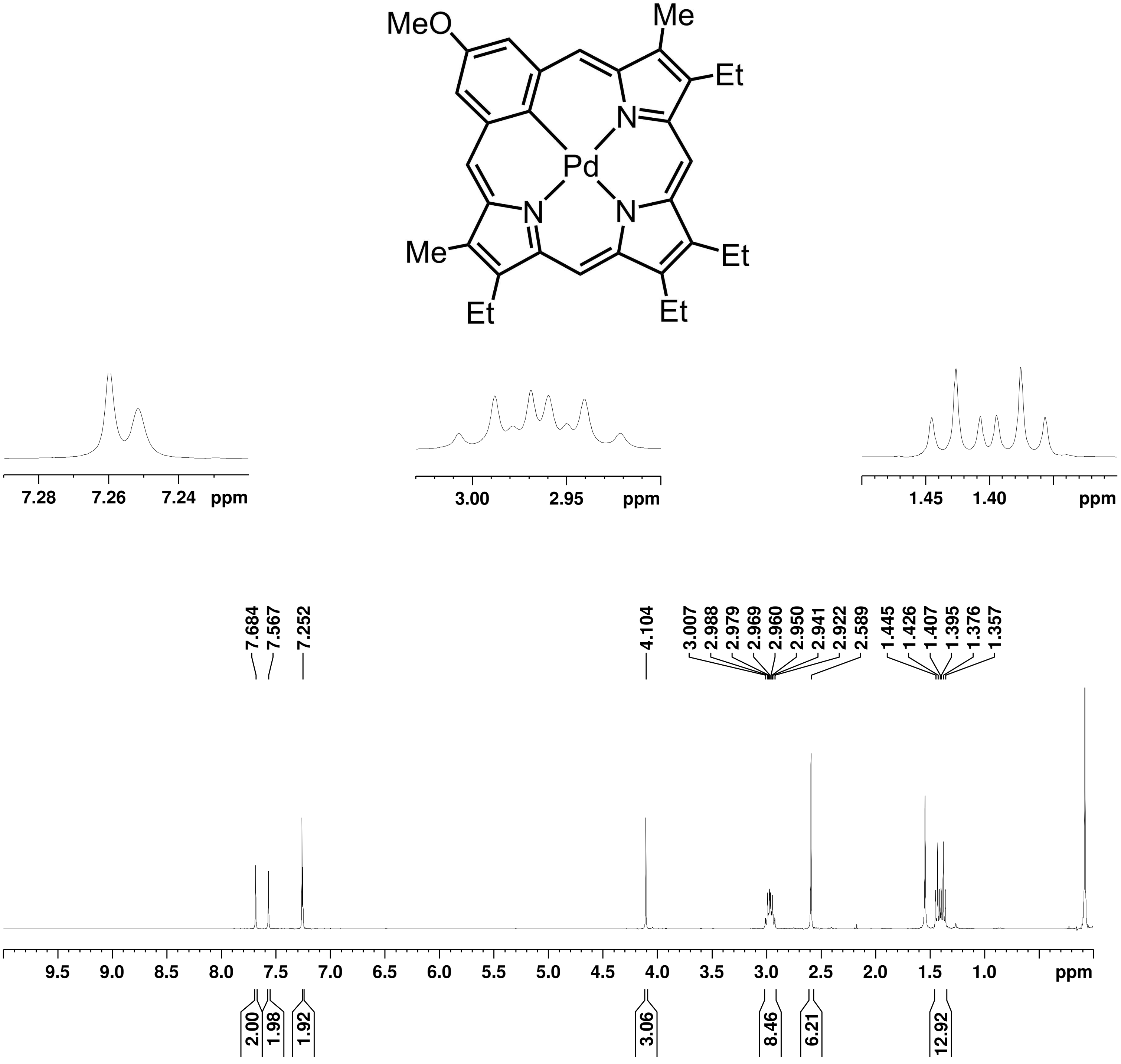

Figure S58. 500 MHz proton NMR spectrum of palladium(II) complex **7aPd** in $CDCl_3$.

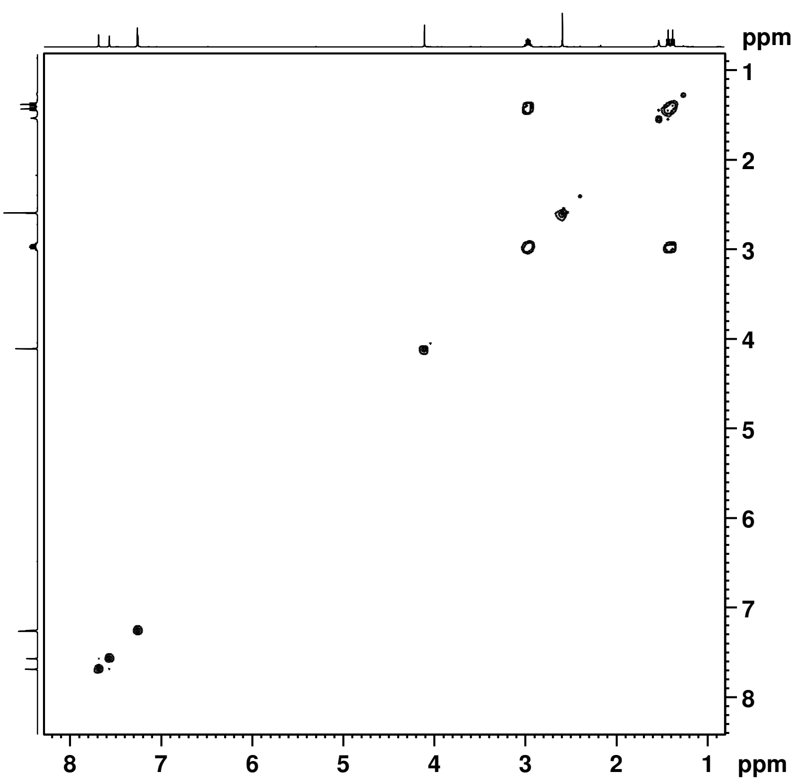

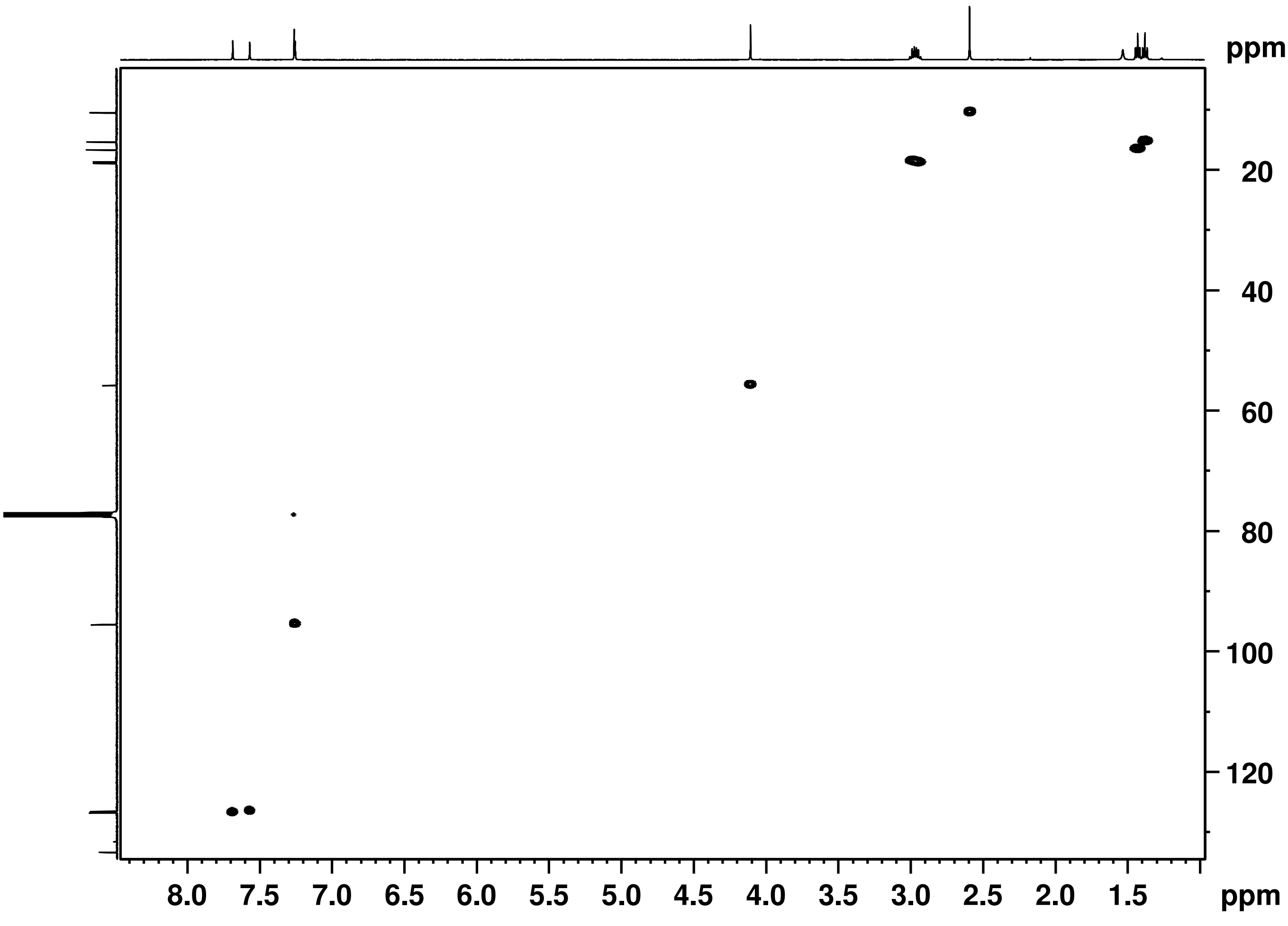

Figure S59. ^{1}H-^{1}H COSY (top) and HSQC (bottom) NMR spectra of **7aPd** in $CDCl_3$.

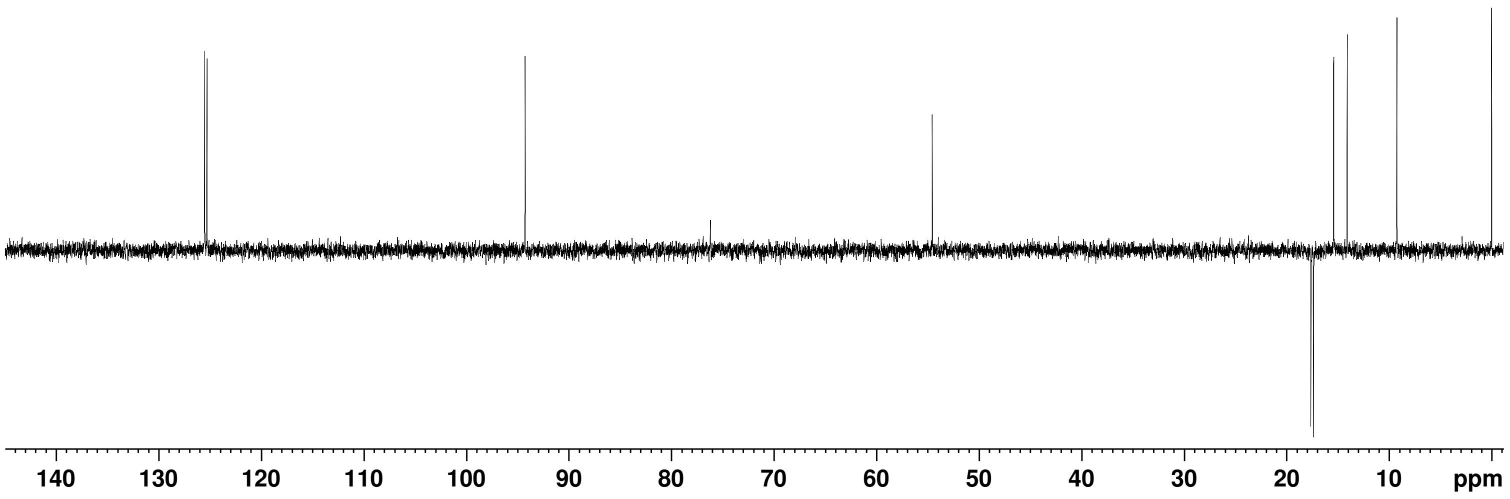

Figure S60. DEPT-135 NMR spectrum of **7aPd** in $CDCl_3$.

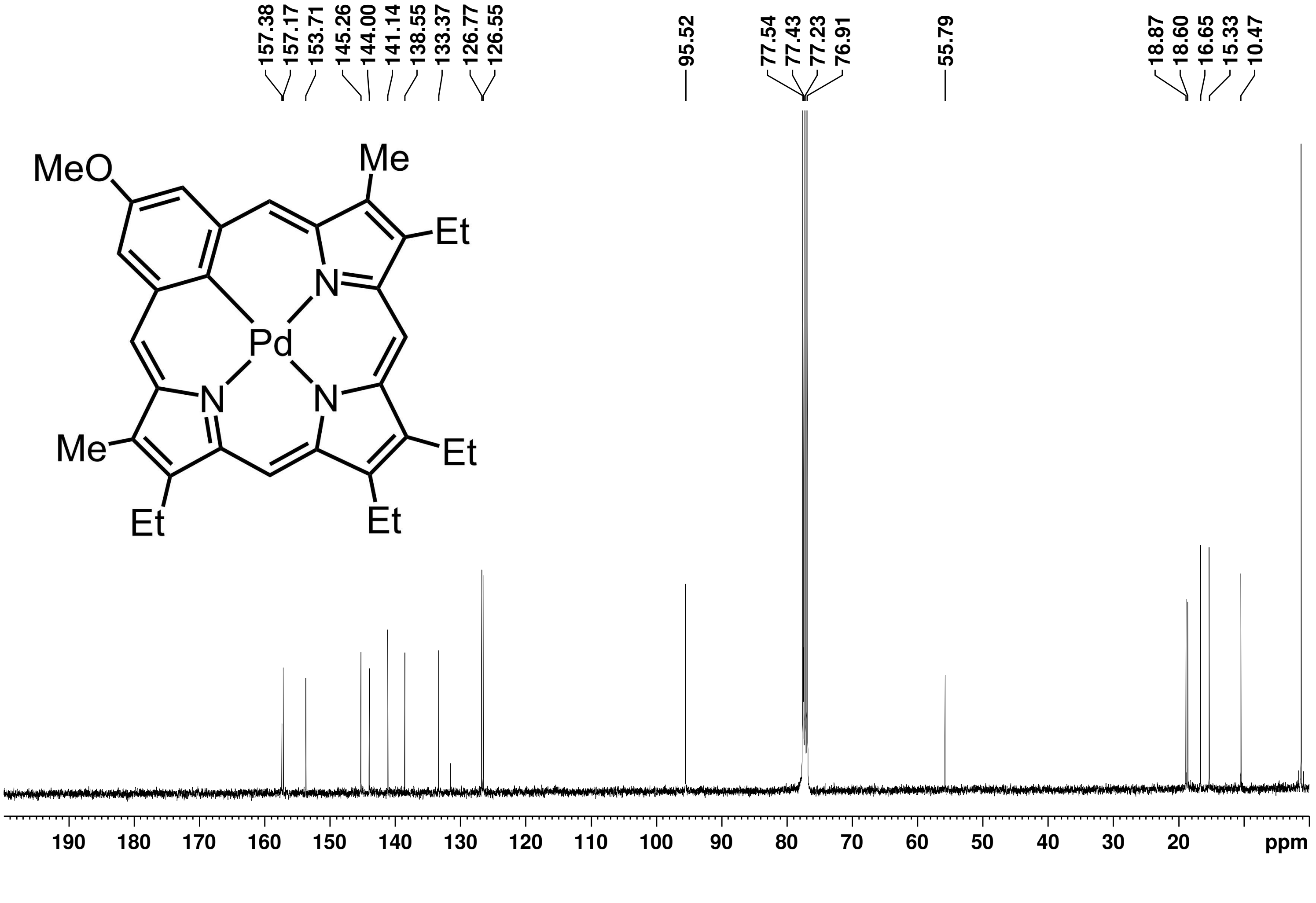

Figure S61. 125 MHz carbon-13 NMR spectrum of **7aPd** in $CDCl_3$.

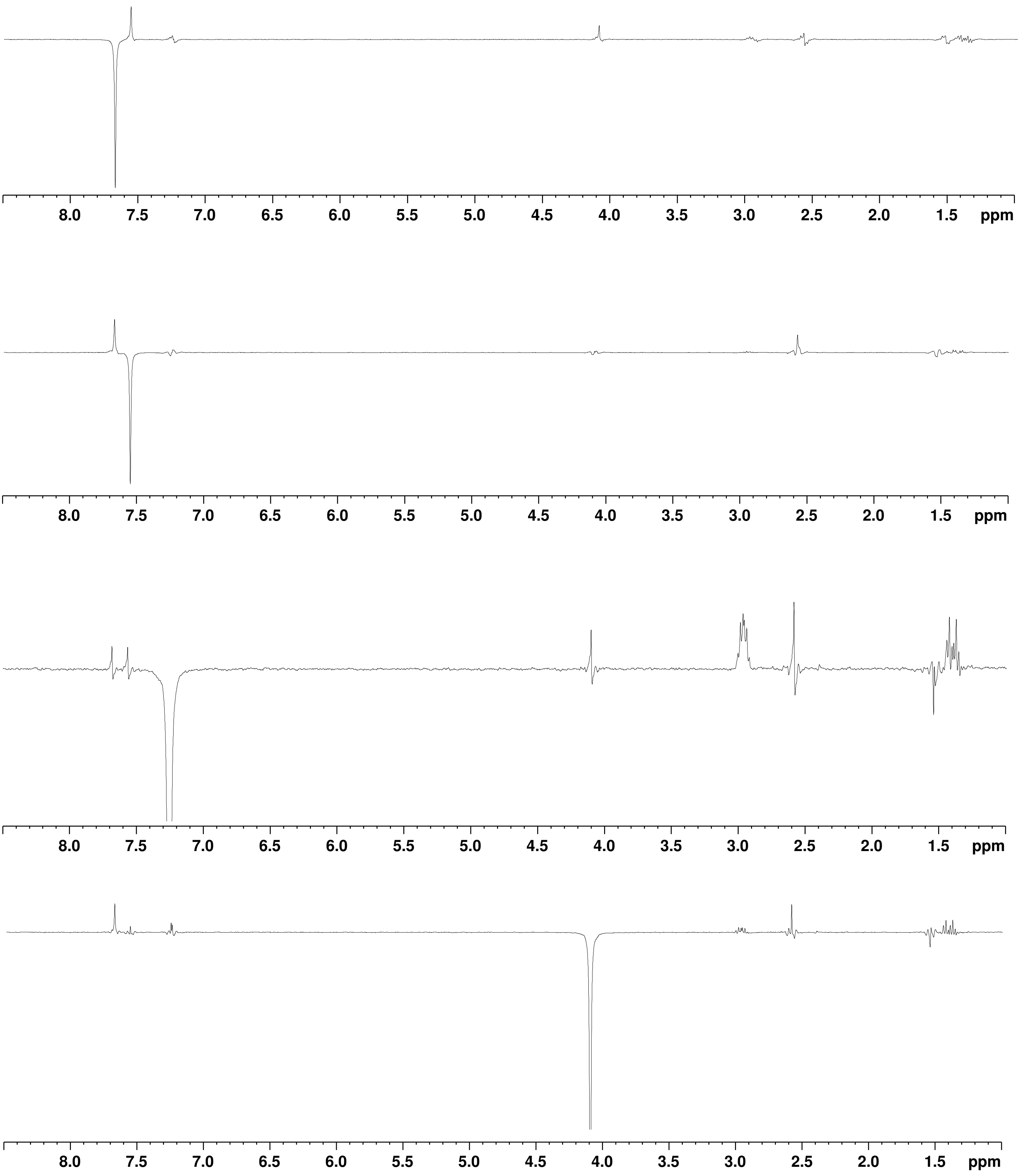

Figure S62. Selected nOe difference proton NMR spectra of **7a**Pd in $CDCl_3$.

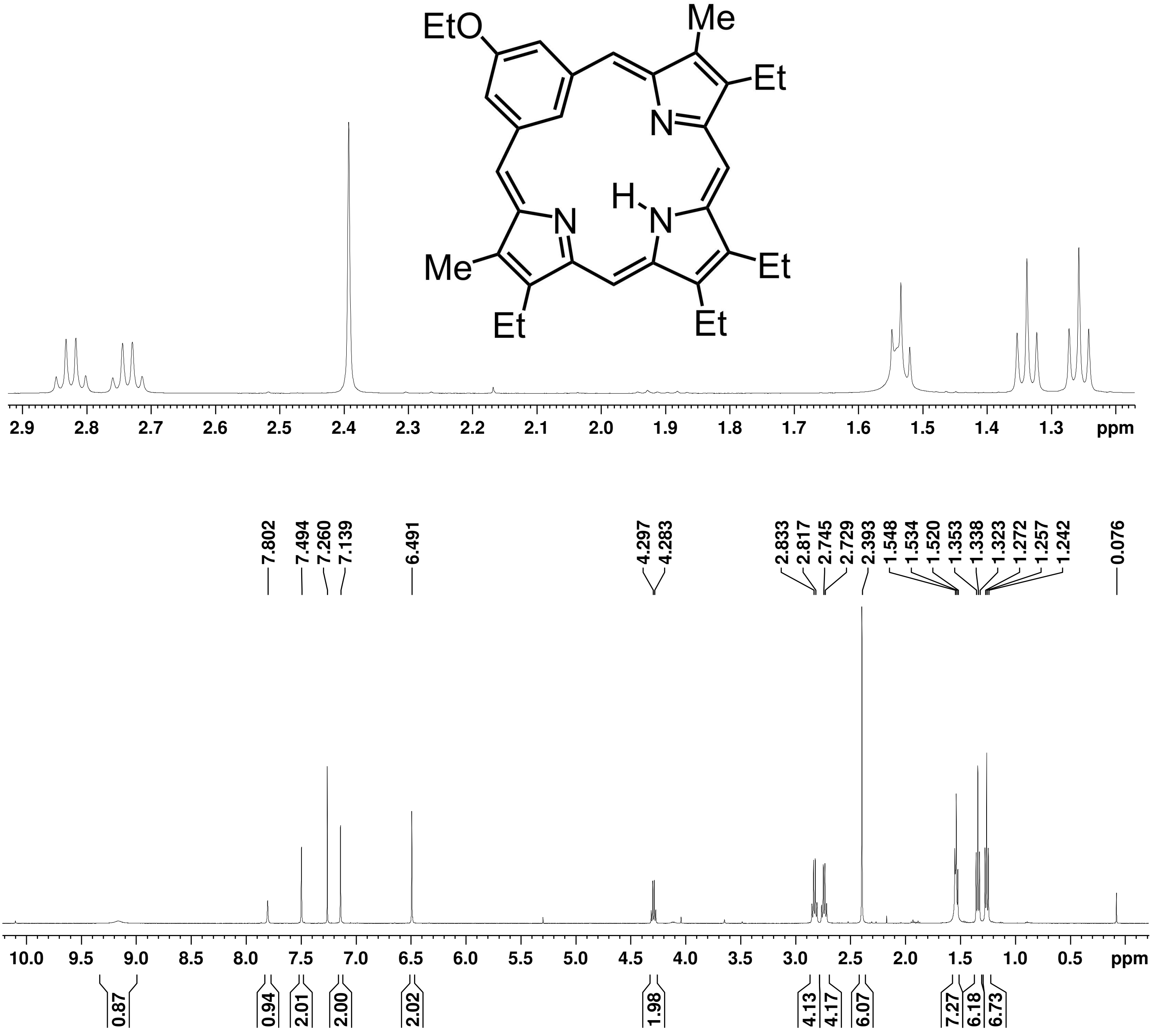

Figure 63. 500 MHz proton NMR spectrum of ethoxybenziporphyrin **7b** in $CDCl_3$.

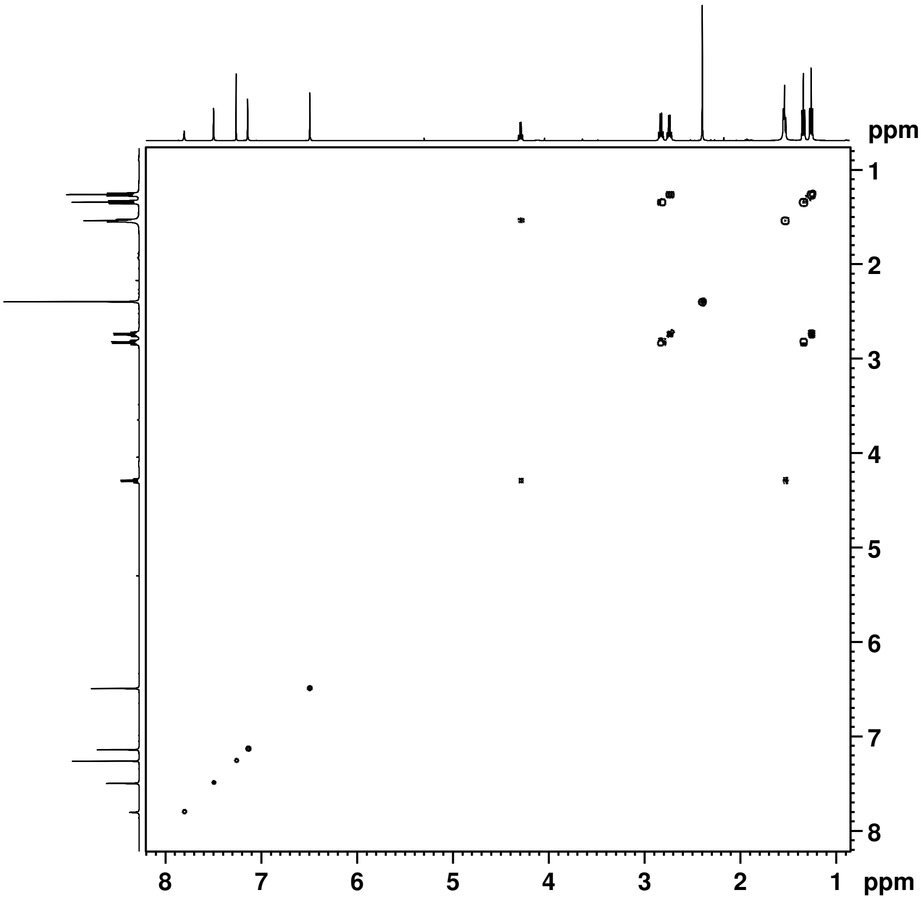

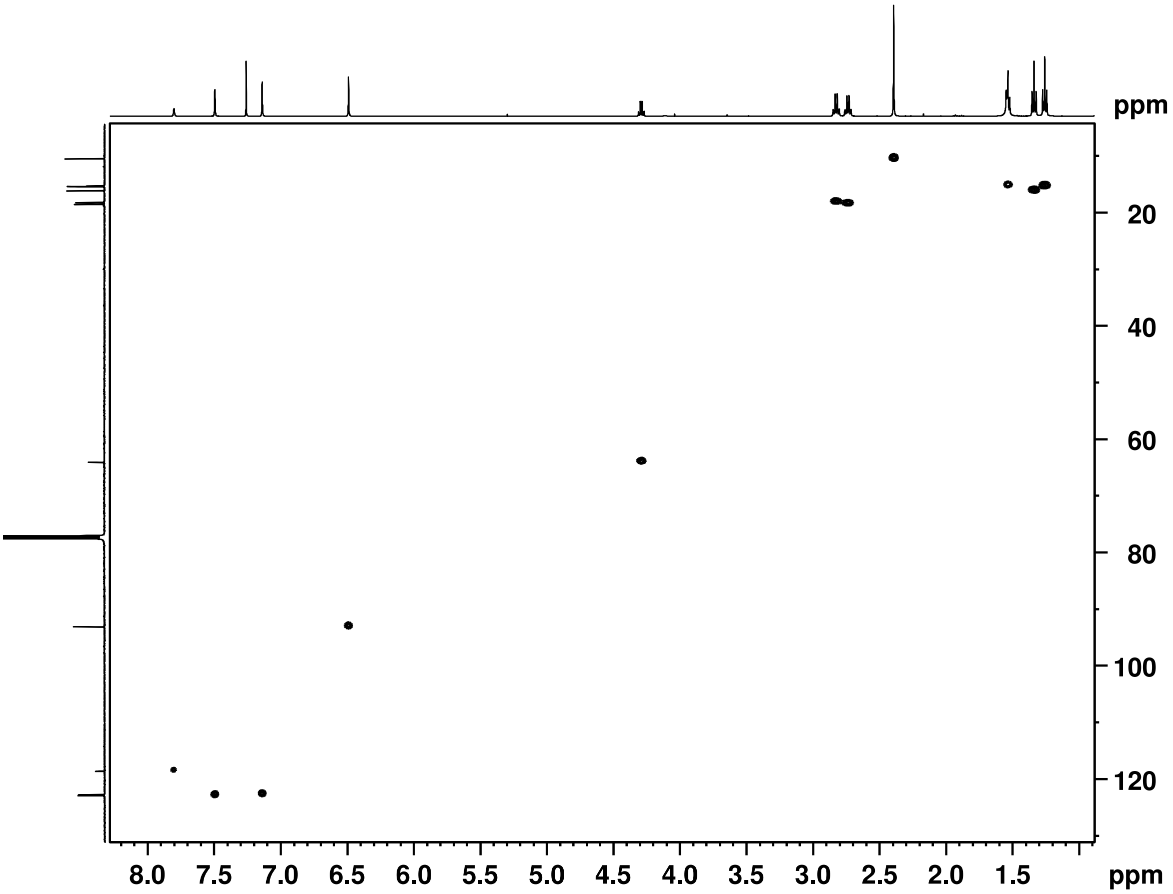

Figure S64. ^{1}H-^{1}H COSY (top) and HSQC (bottom) NMR spectra of **7b** in $CDCl_3$.

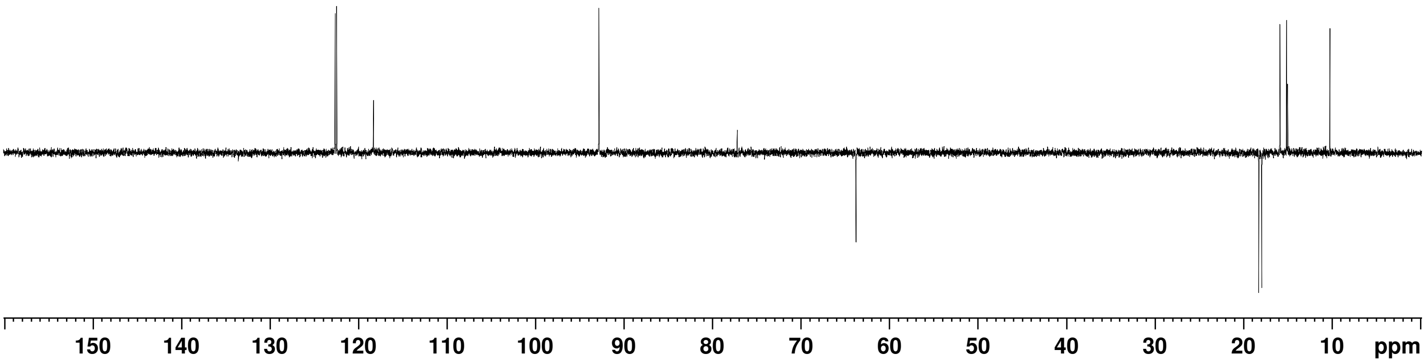

Figure S65. DEPT-135 NMR spectrum of **7b** in $CDCl_3$.

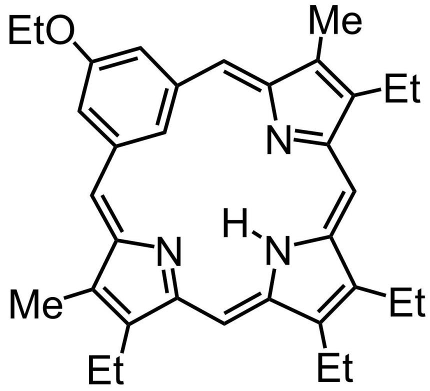

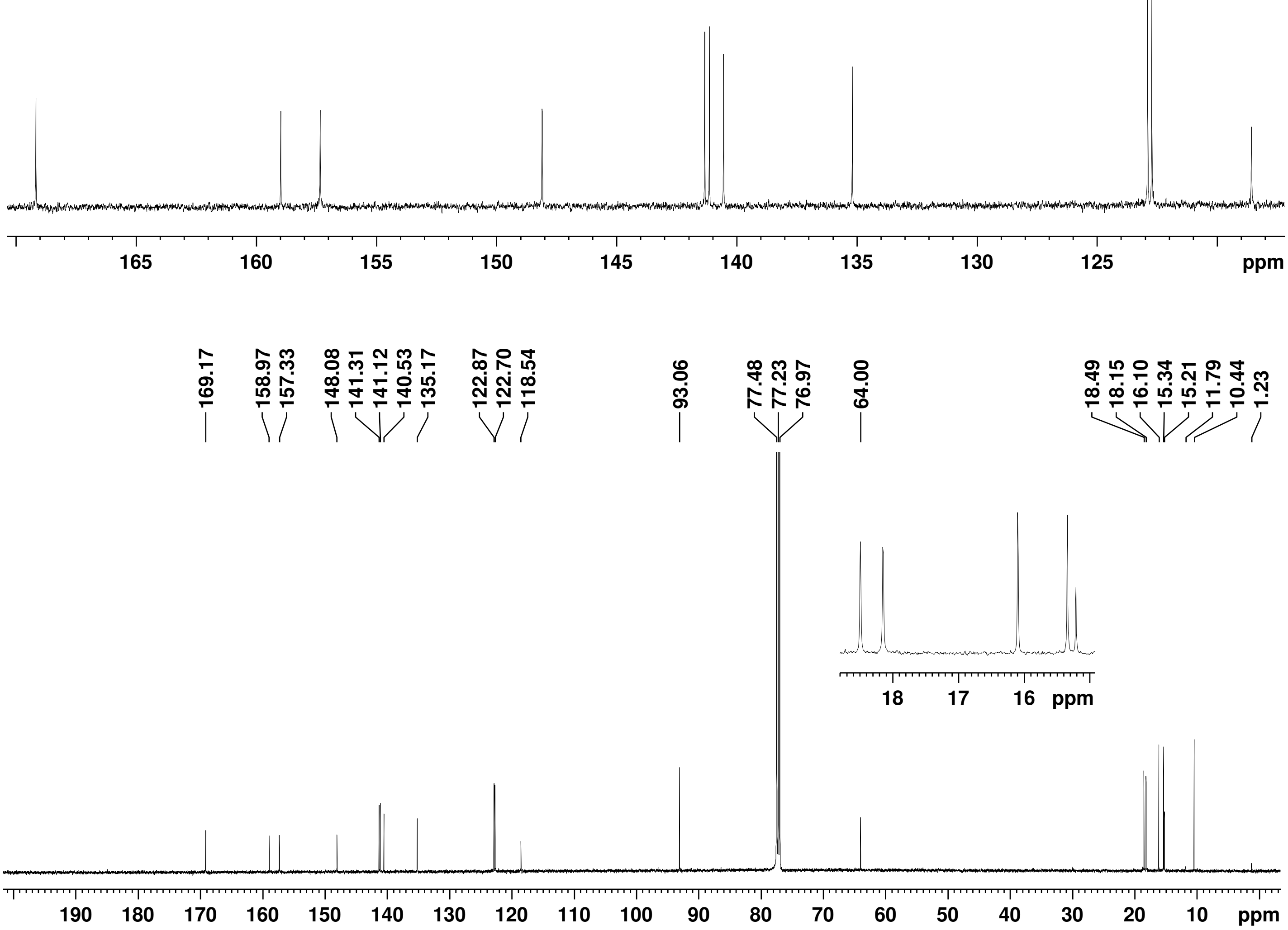

Figure S66. 125 MHz carbon-13 NMR spectrum of **7b** in $CDCl_3$.

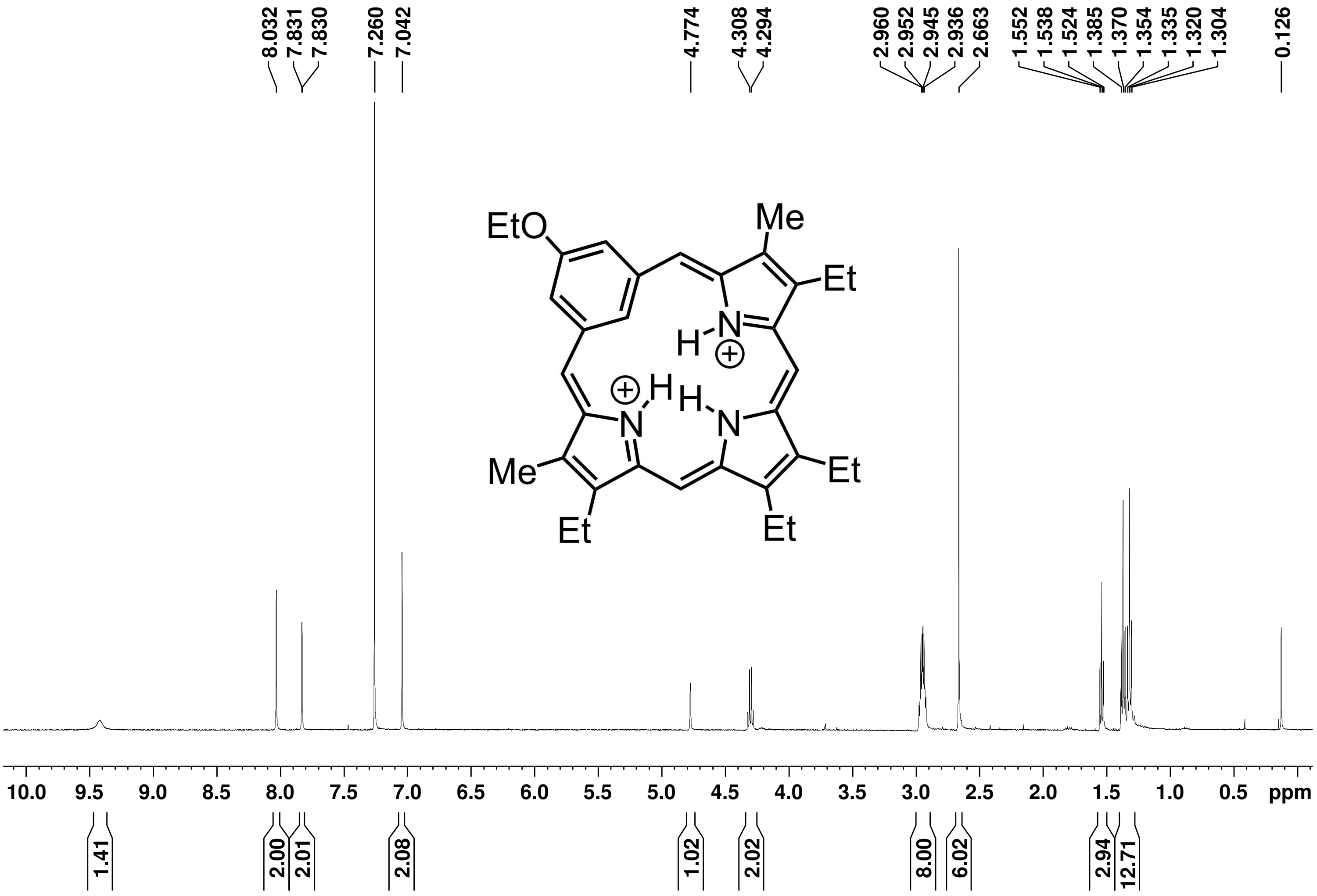

Figure S67. 500 MHz proton NMR spectrum of ethoxybenziporphyrin dication **7b**H_2^{2+} in TFA-$CDCl_3$.

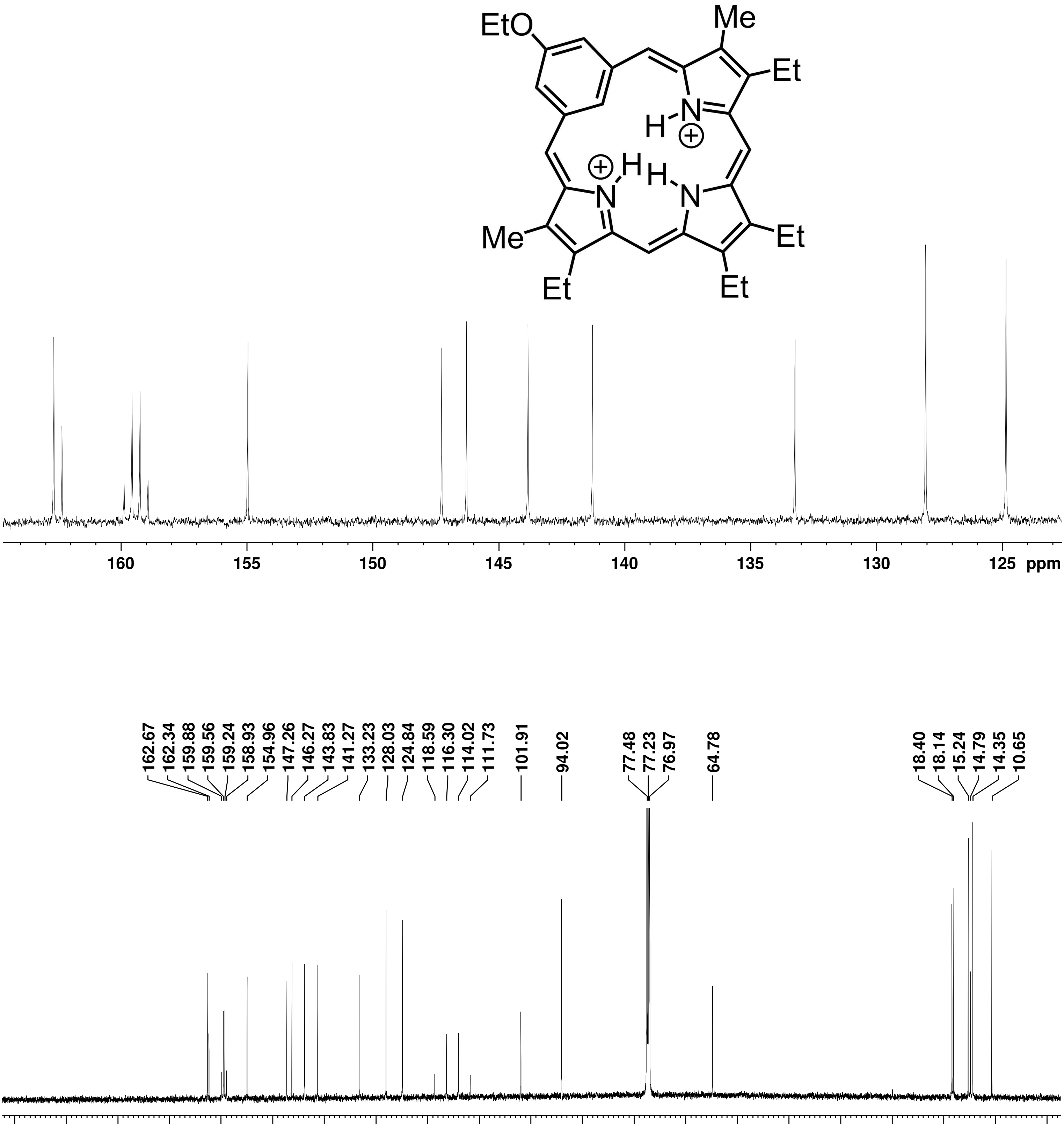

Figure S68. 125 MHz carbon-13 NMR spectrum of **7b**H_2^{2+} in TFA-$CDCl_3$.

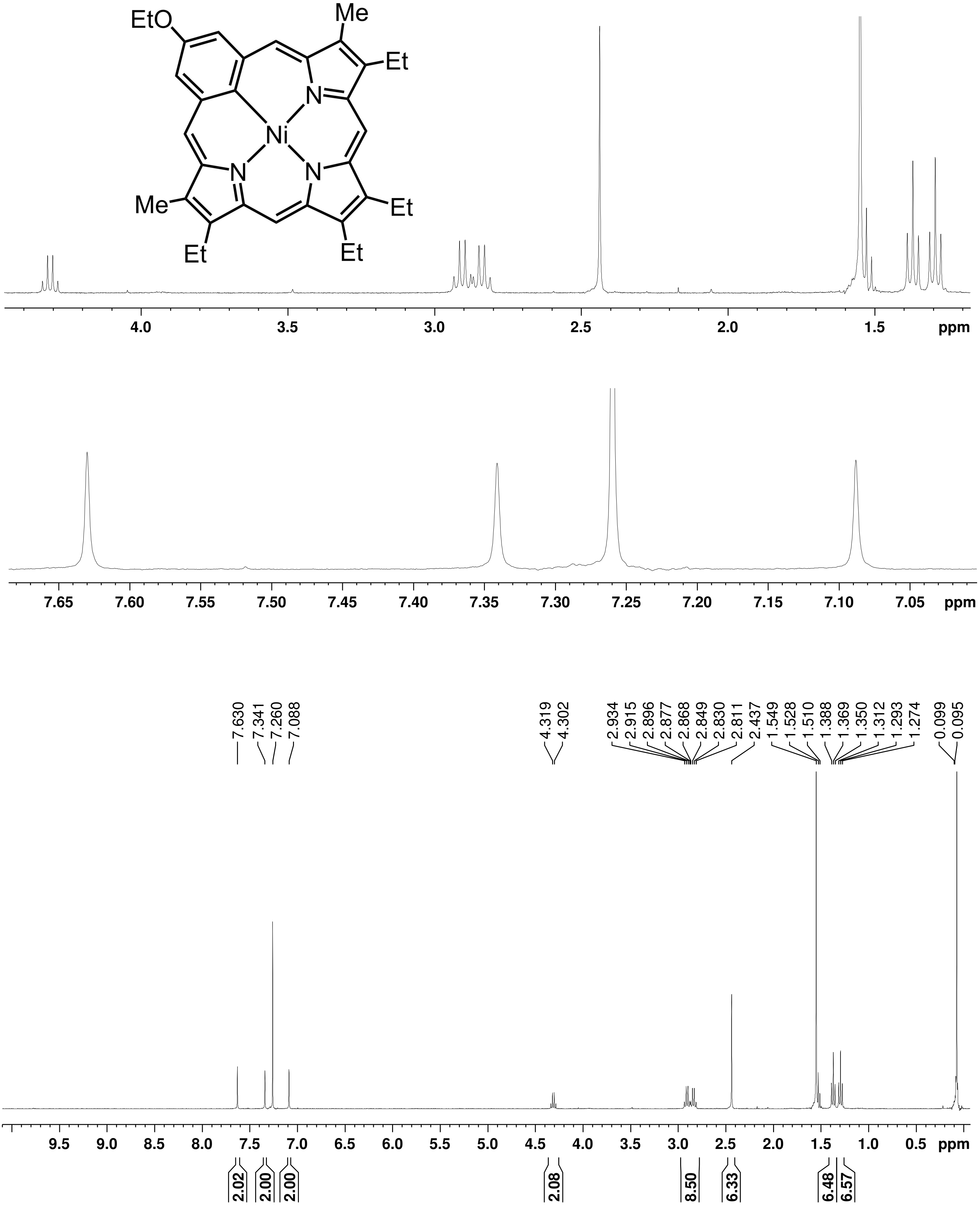

Figure S69. 500 MHz proton NMR spectrum of nickel(II) complex **7bNi** in $CDCl_3$.

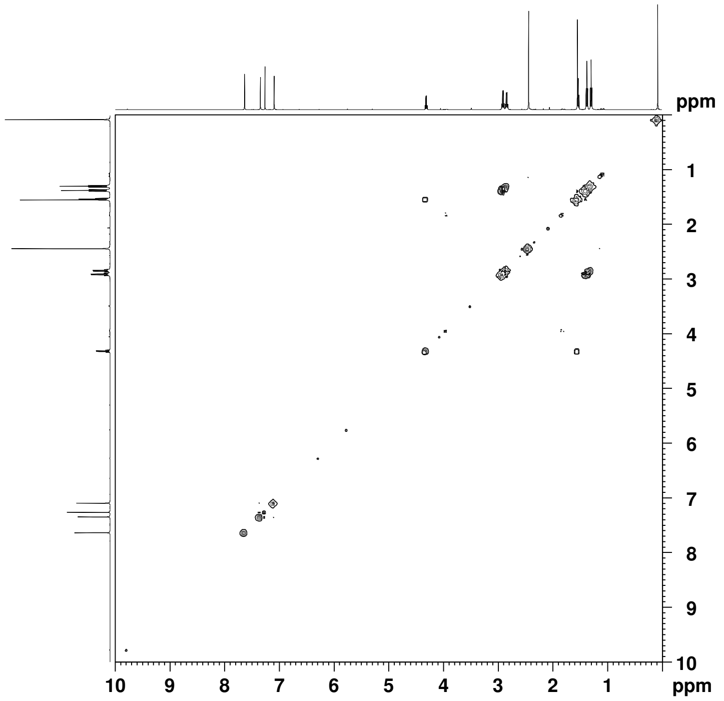

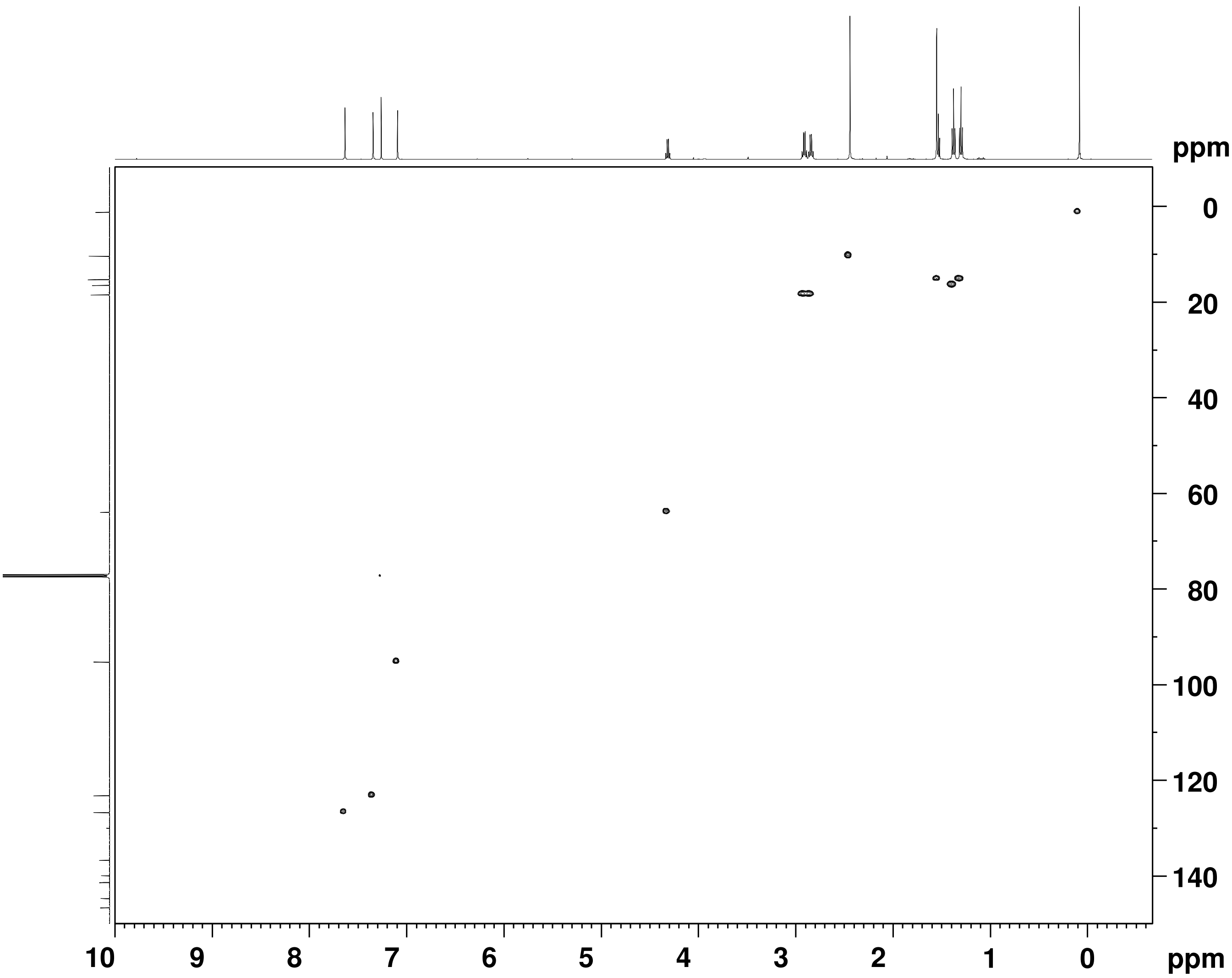

Figure S70. ^{1}H-^{1}H COSY (top) and HSQC (bottom) NMR spectra of **7bNi** in $CDCl_3$.

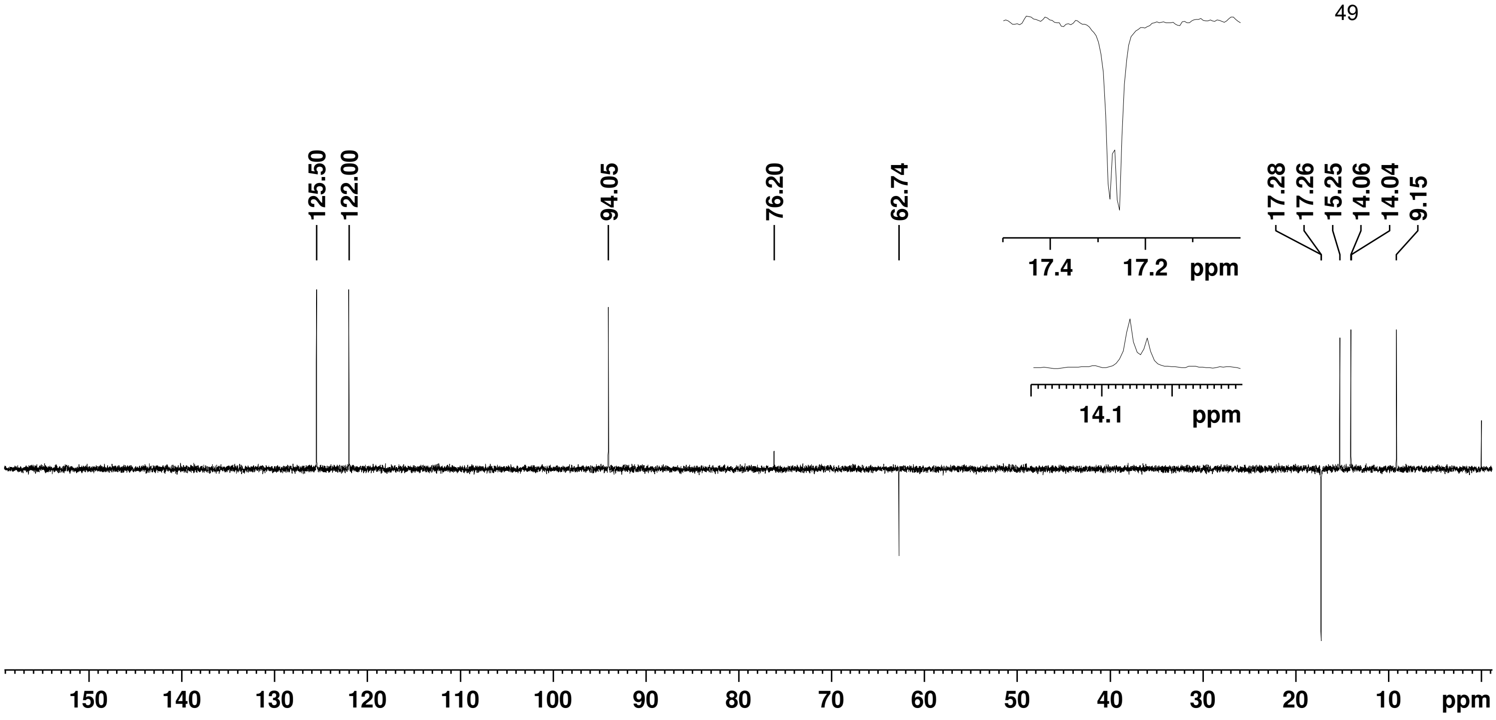

Figure S71. DEPT-135 NMR spectrum of **7bNi** in $CDCl_3$.

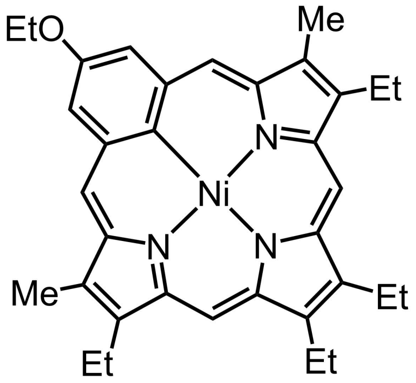

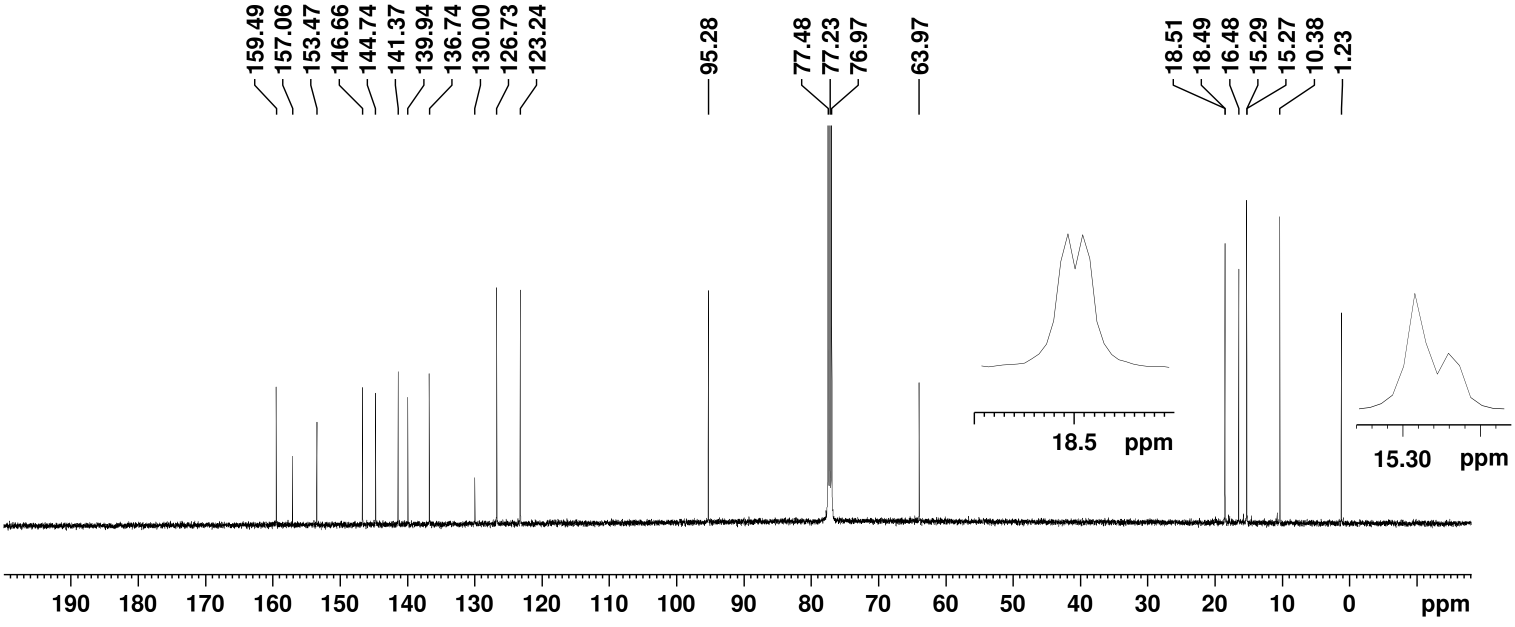

Figure S72. 125 MHz carbon-13 NMR spectrum of **7bNi** in $CDCl_3$.

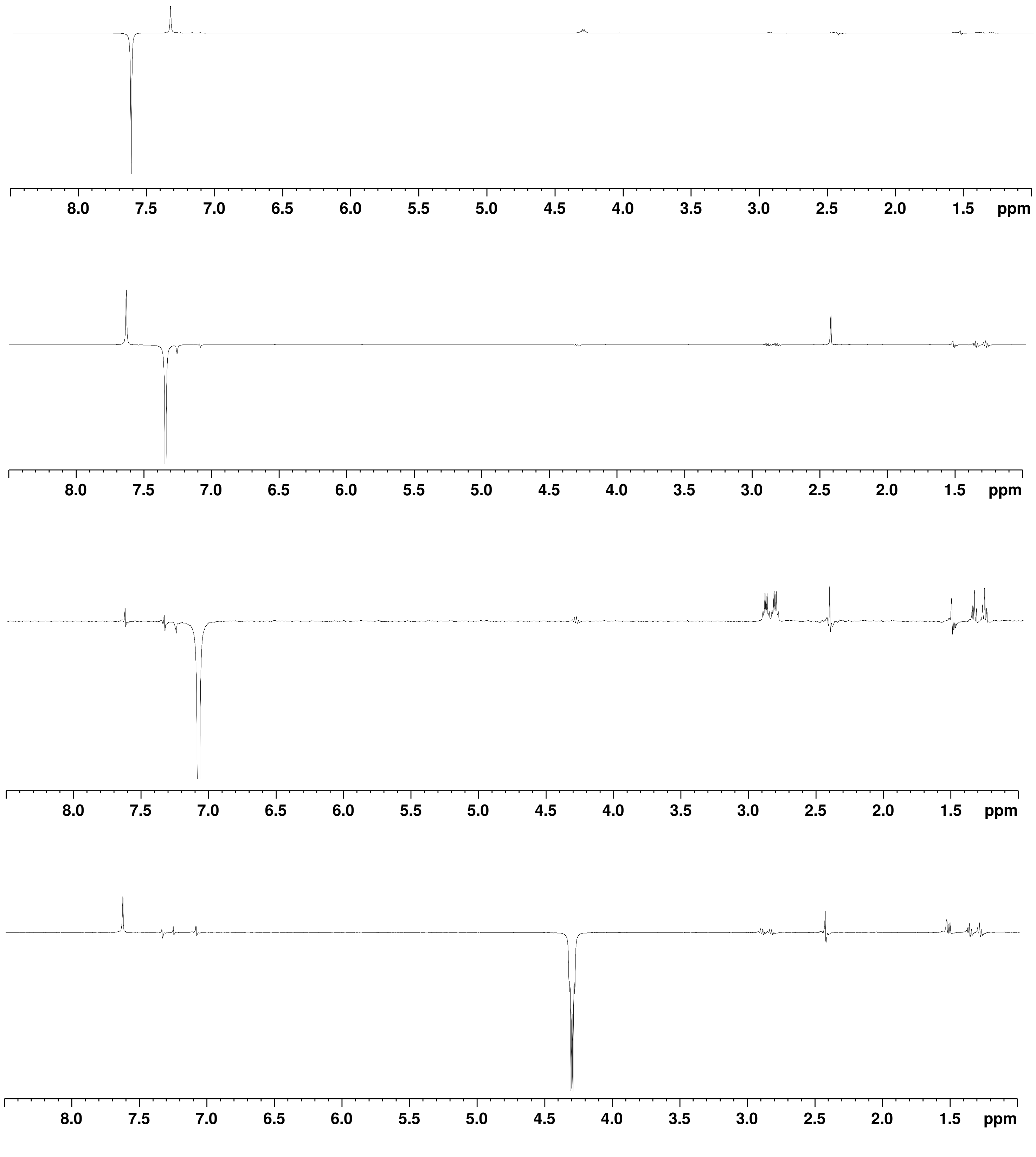

Figure S73. Selected nOe difference proton NMR spectra of **7bNi** in $CDCl_3$.

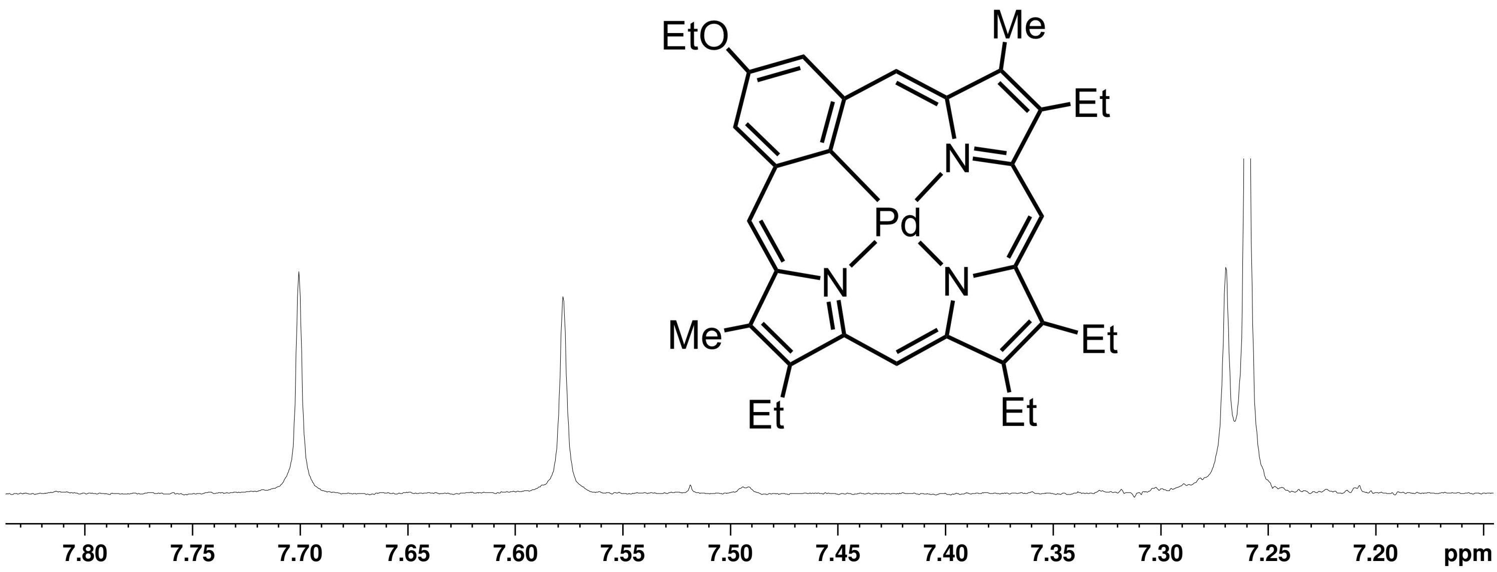

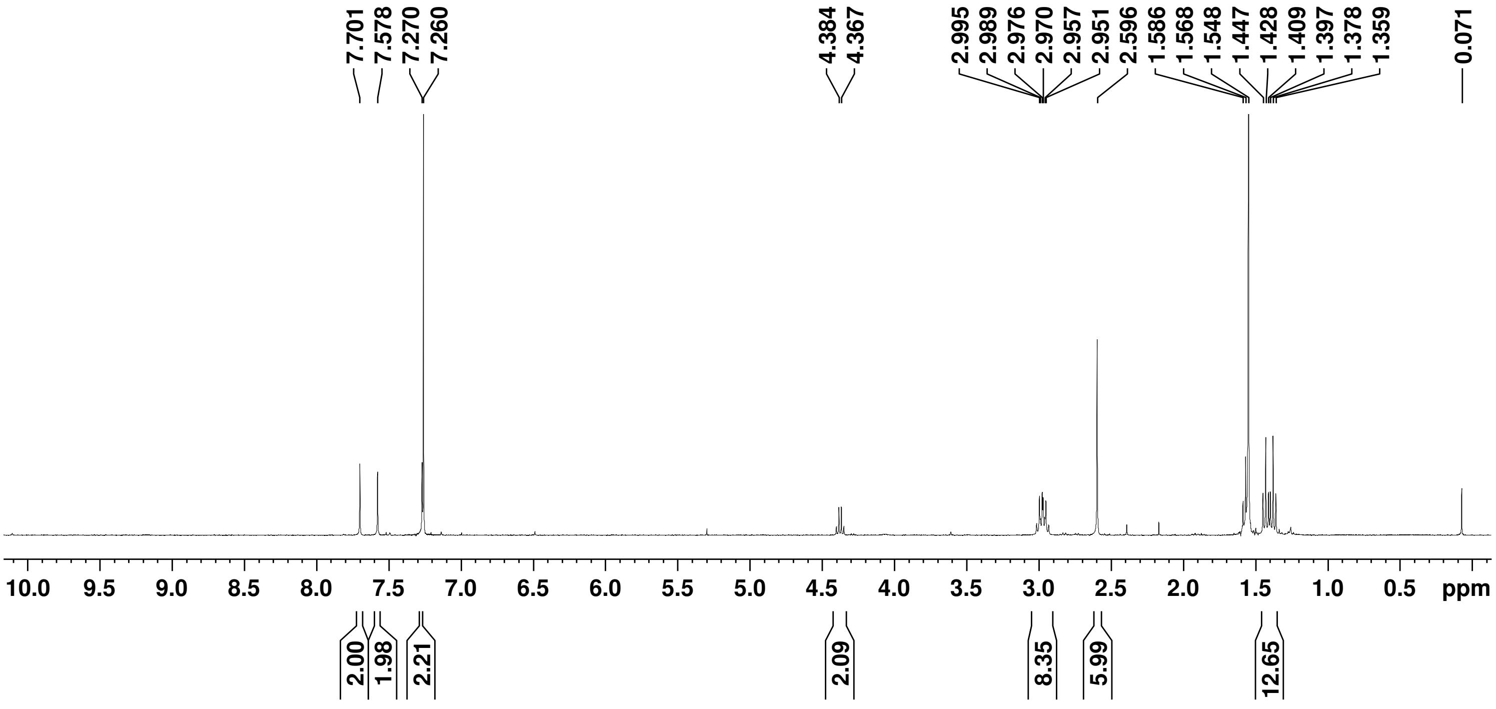

Figure S74. 500 MHz proton NMR spectrum of palladium complex **7bPd** in $CDCl_3$.

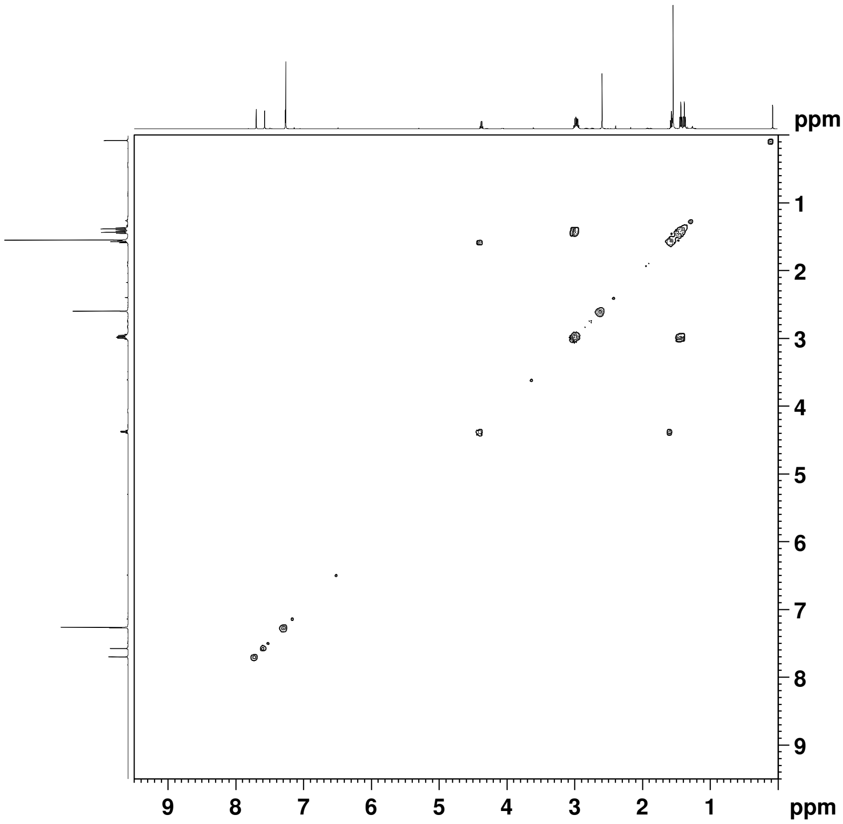

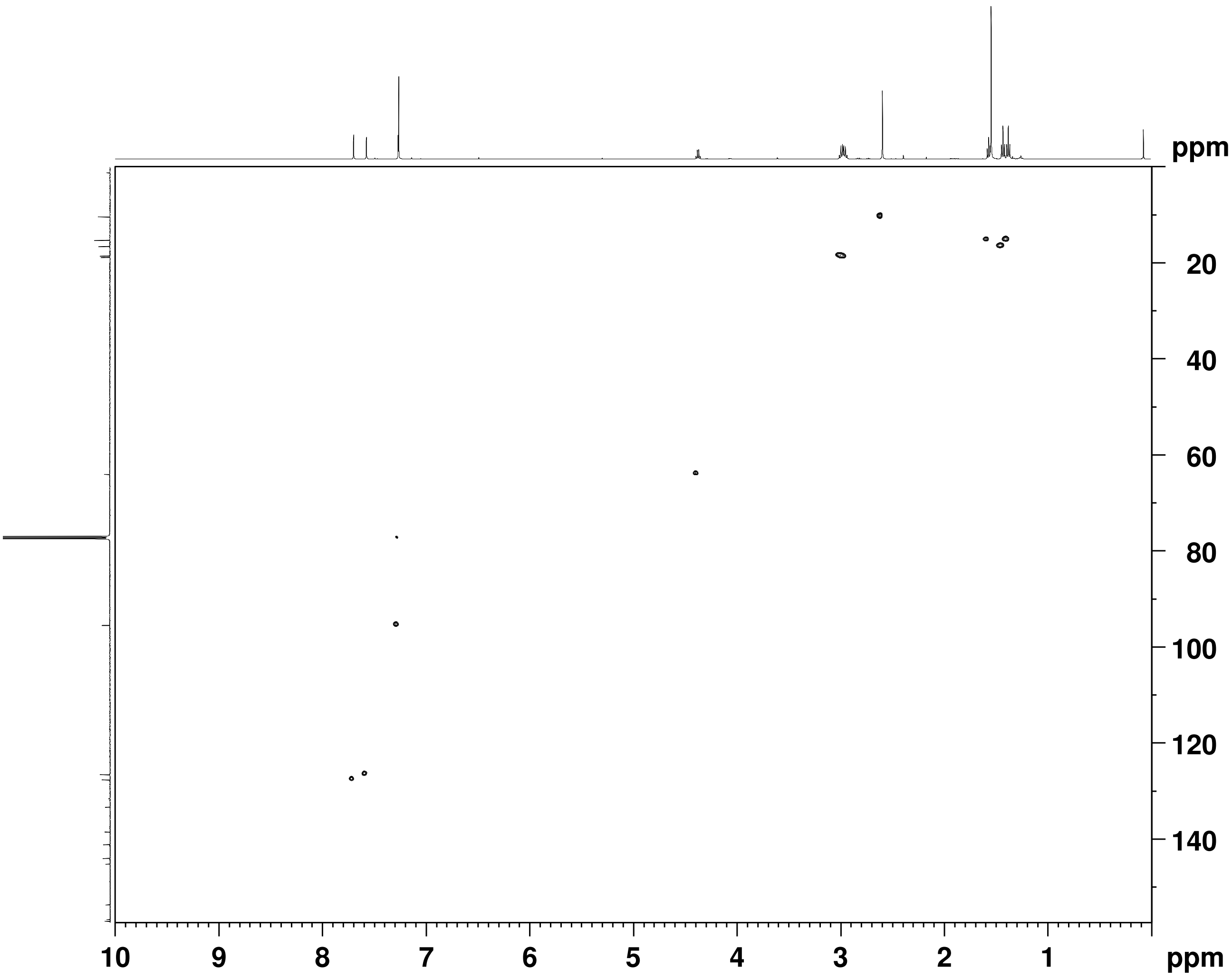

Figure S75. ^{1}H-^{1}H COSY (top) and HSQC (bottom) NMR spectra of **7bPd** in $CDCl_3$.

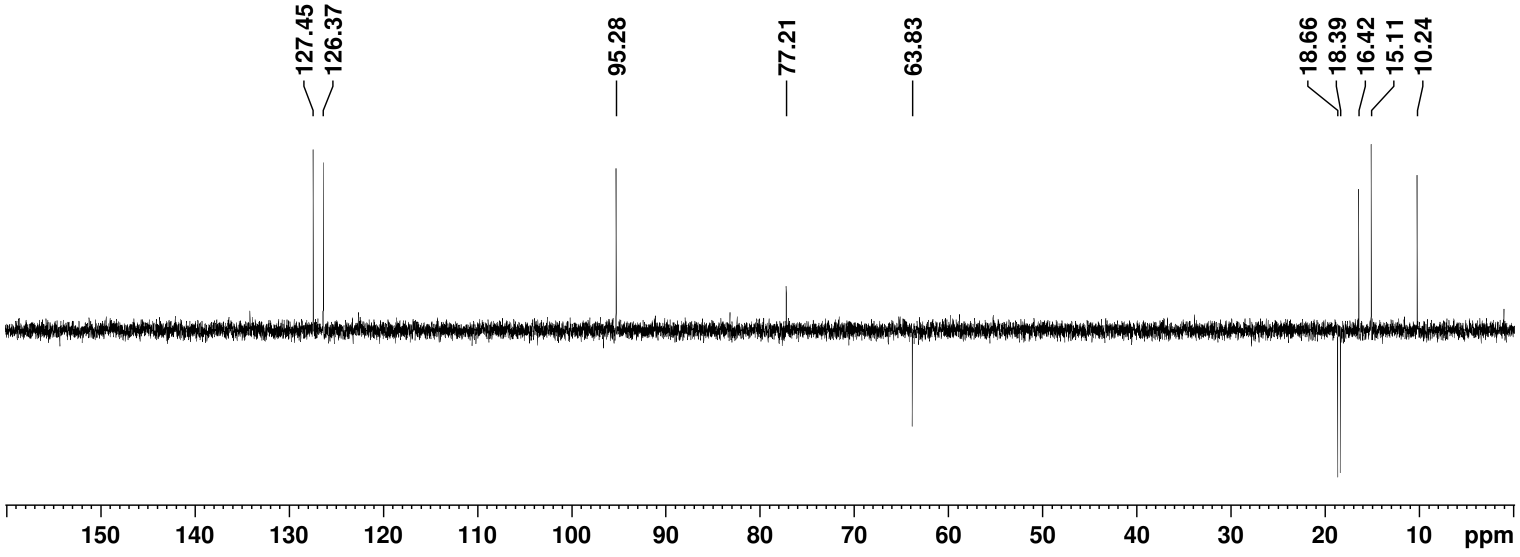

Figure S76. DEPT-135 NMR spectrum of **7bPd** in $CDCl_3$.

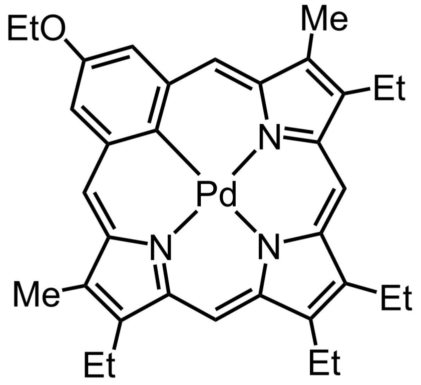

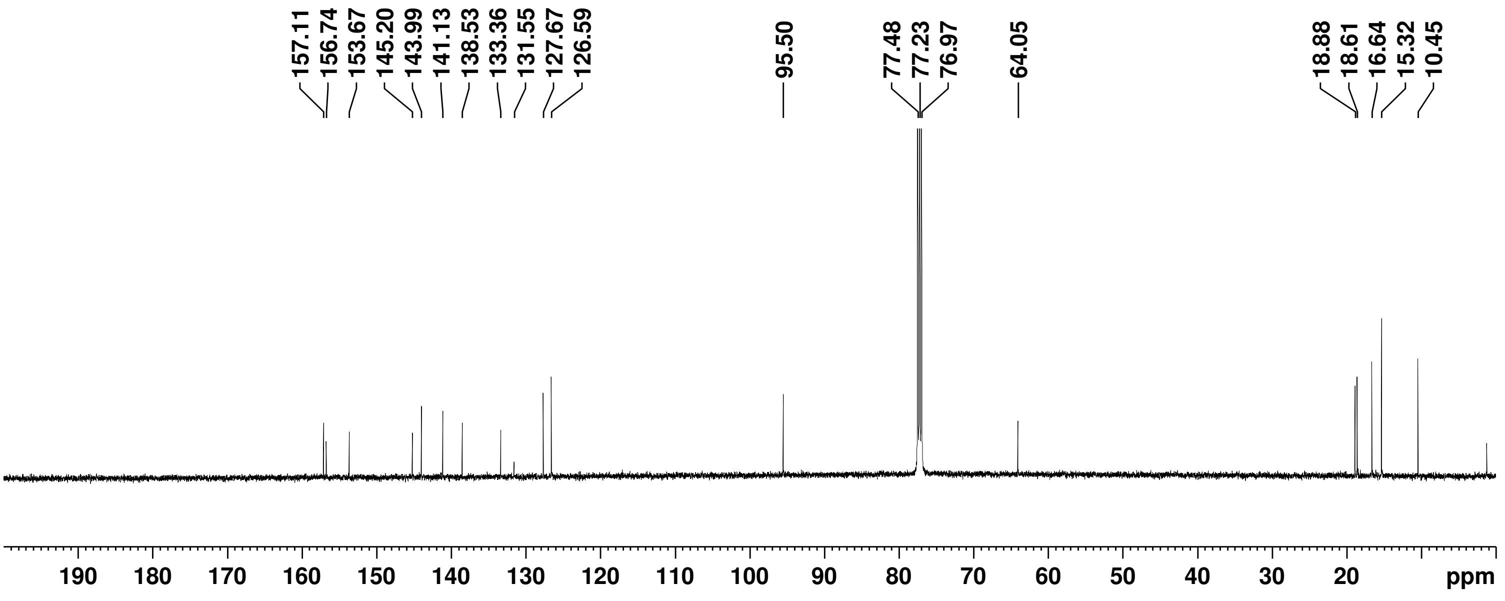

Figure S77. 125 MHz carbon-13 NMR spectrum of **7bPd** in $CDCl_3$.

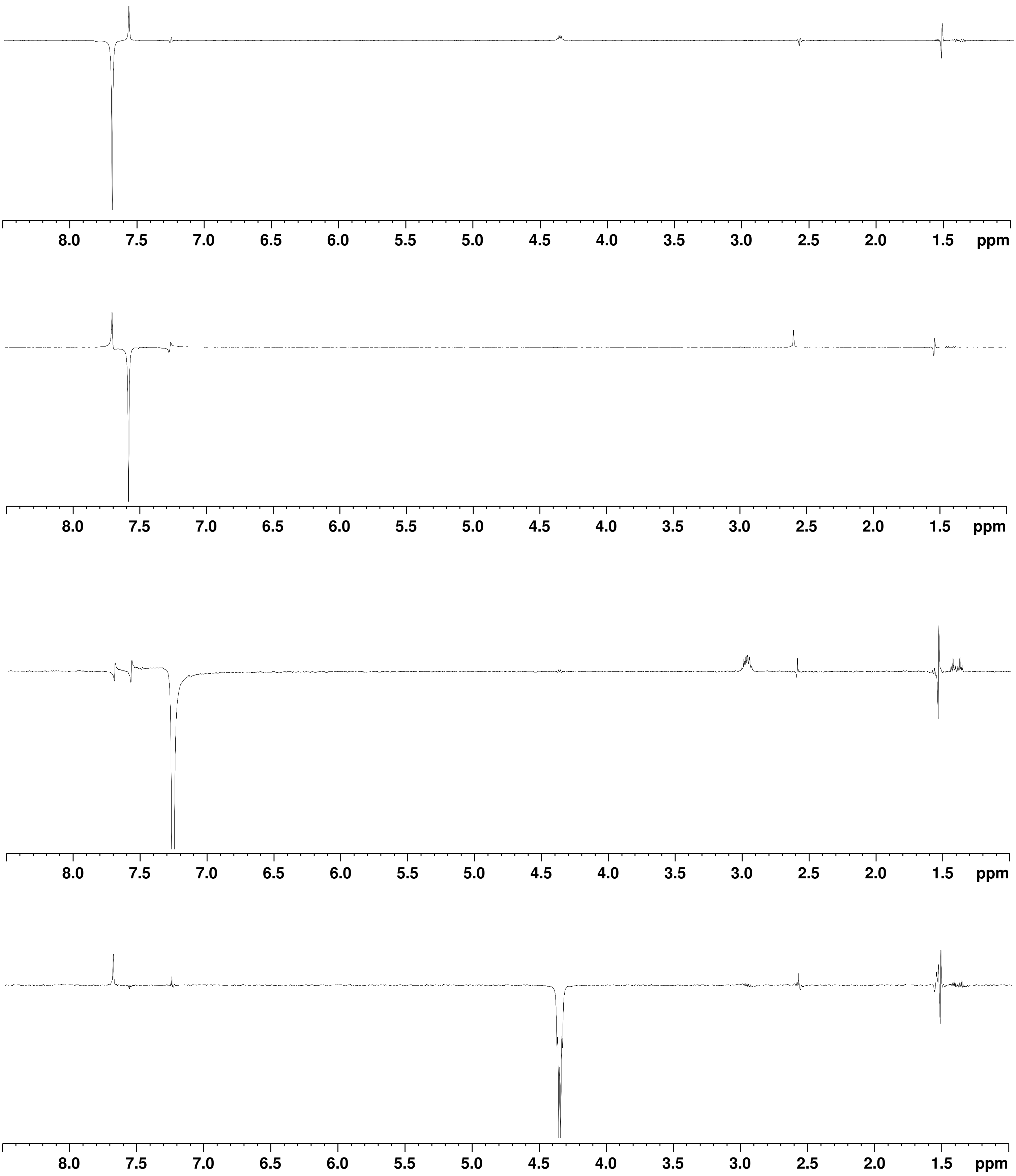

Figure S78. Selected nOe difference proton NMR spectra of **7bPd** in $CDCl_3$.

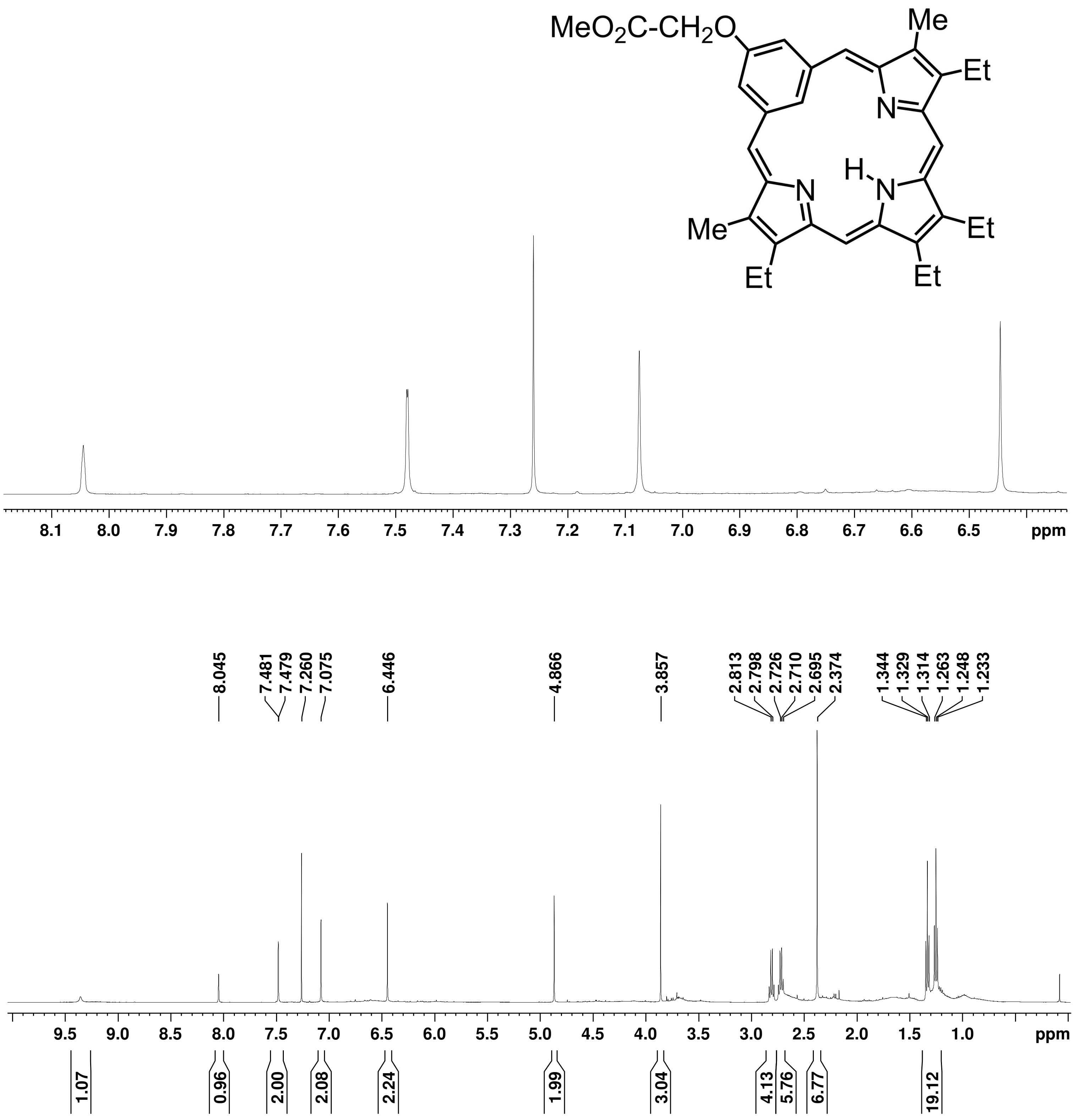

Figure S79. 500 MHz proton NMR spectrum of methoxycarbonylmethoxybenziporphyrin **7c** in $CDCl_3$.

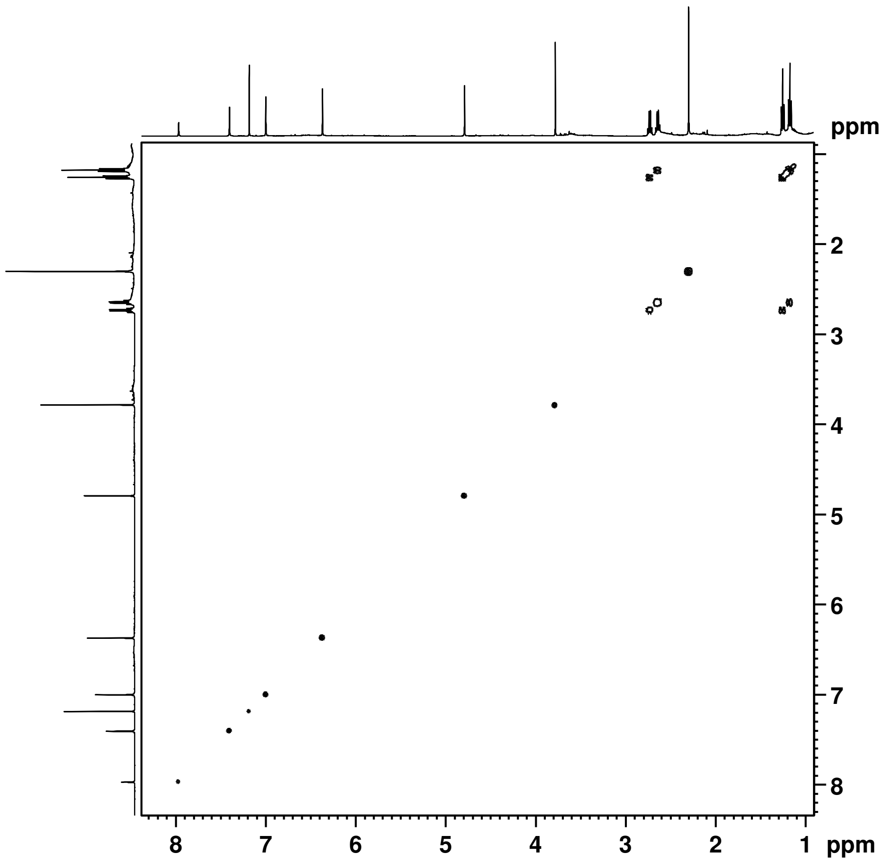

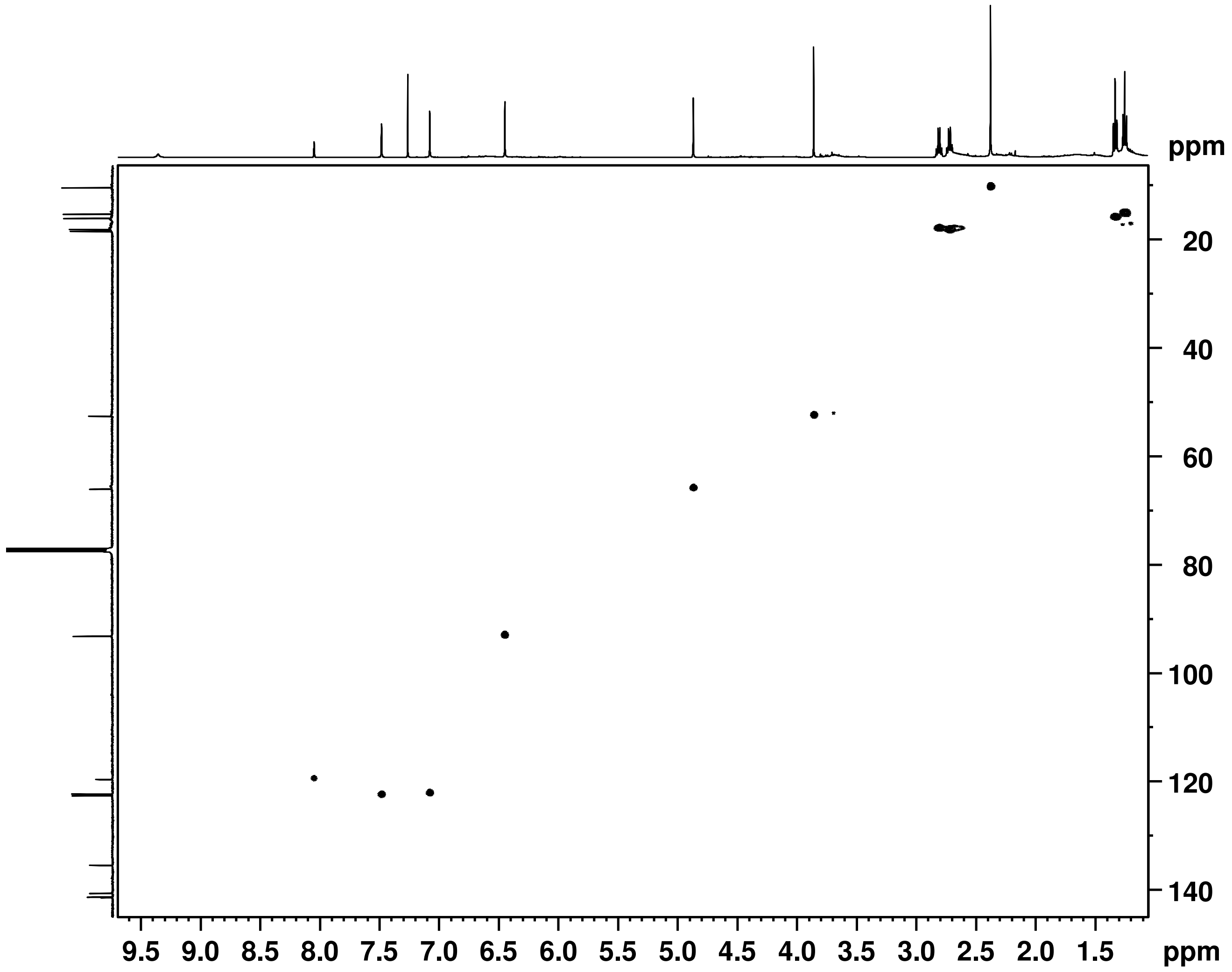

Figure S80. ^{1}H-^{1}H COSY (top) and HSQC (bottom) NMR spectra of **7c** in $CDCl_3$.

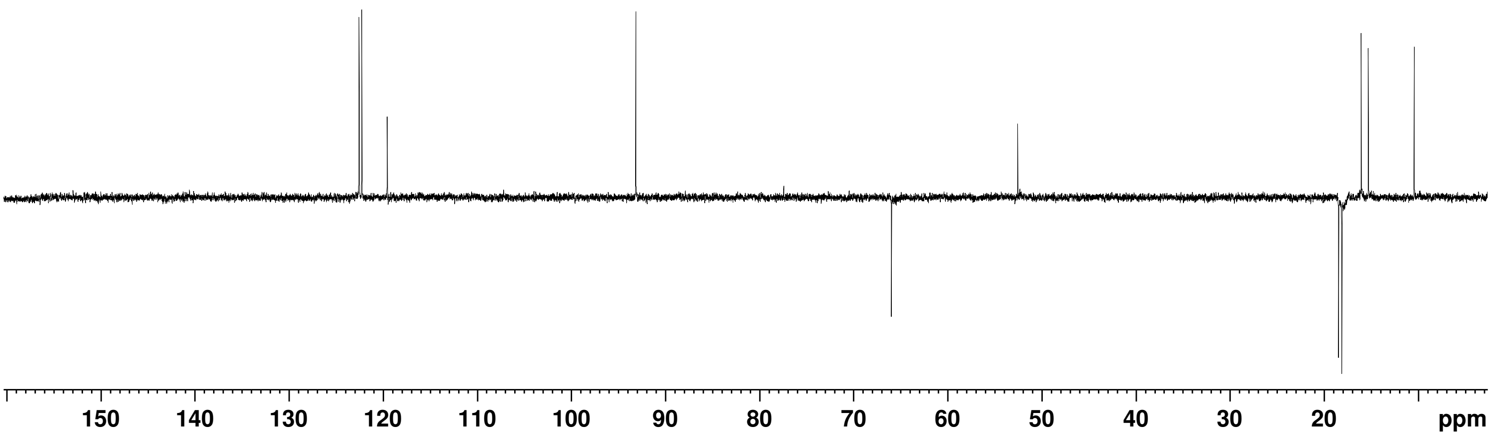

Figure S81. DEPT-135 NMR spectrum of **7c** in $CDCl_3$.

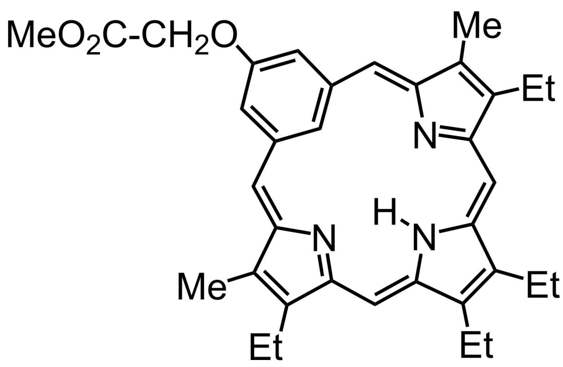

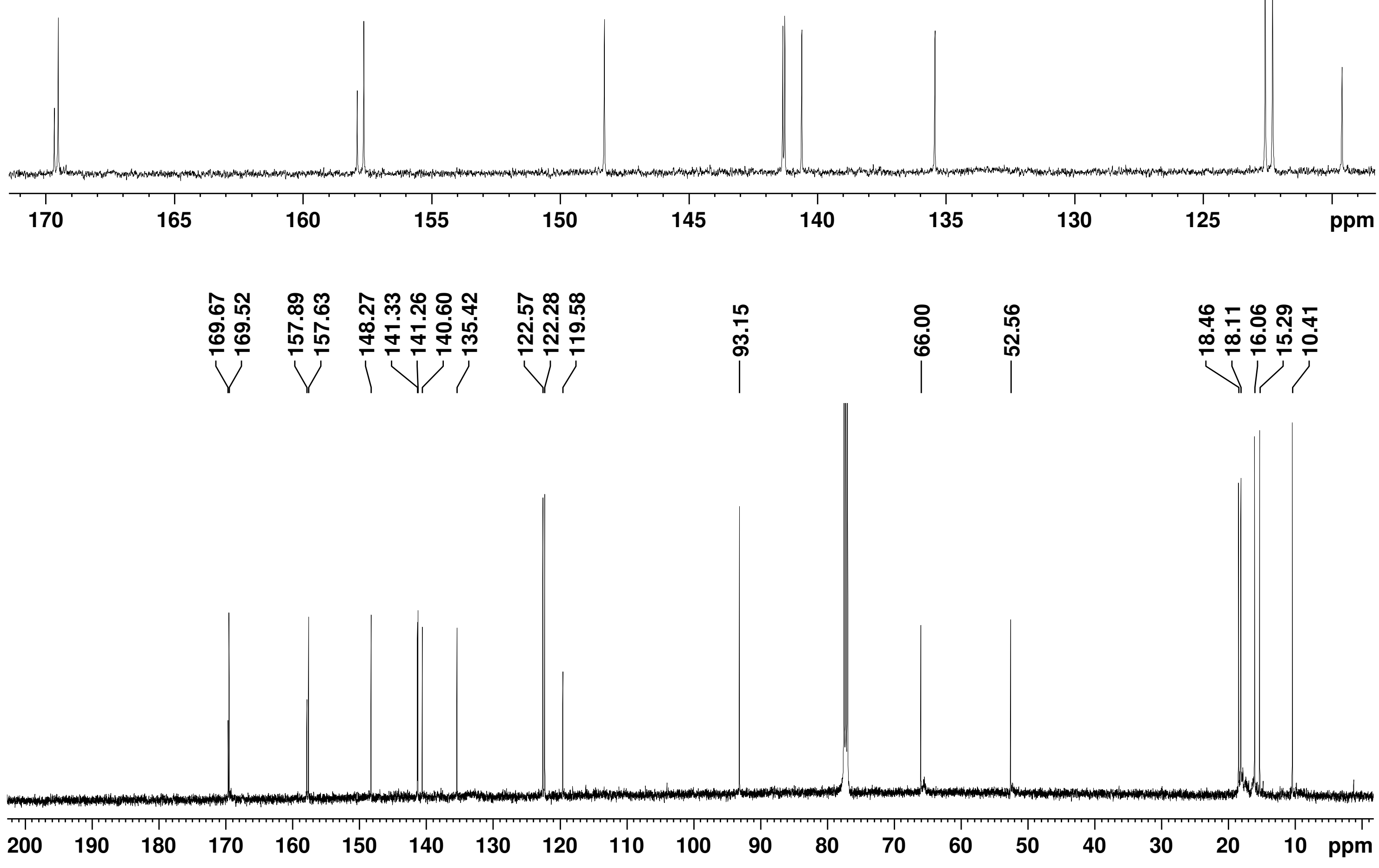

Figure S82. 125 MHz carbon-13 NMR spectrum of **7c** in $CDCl_3$.

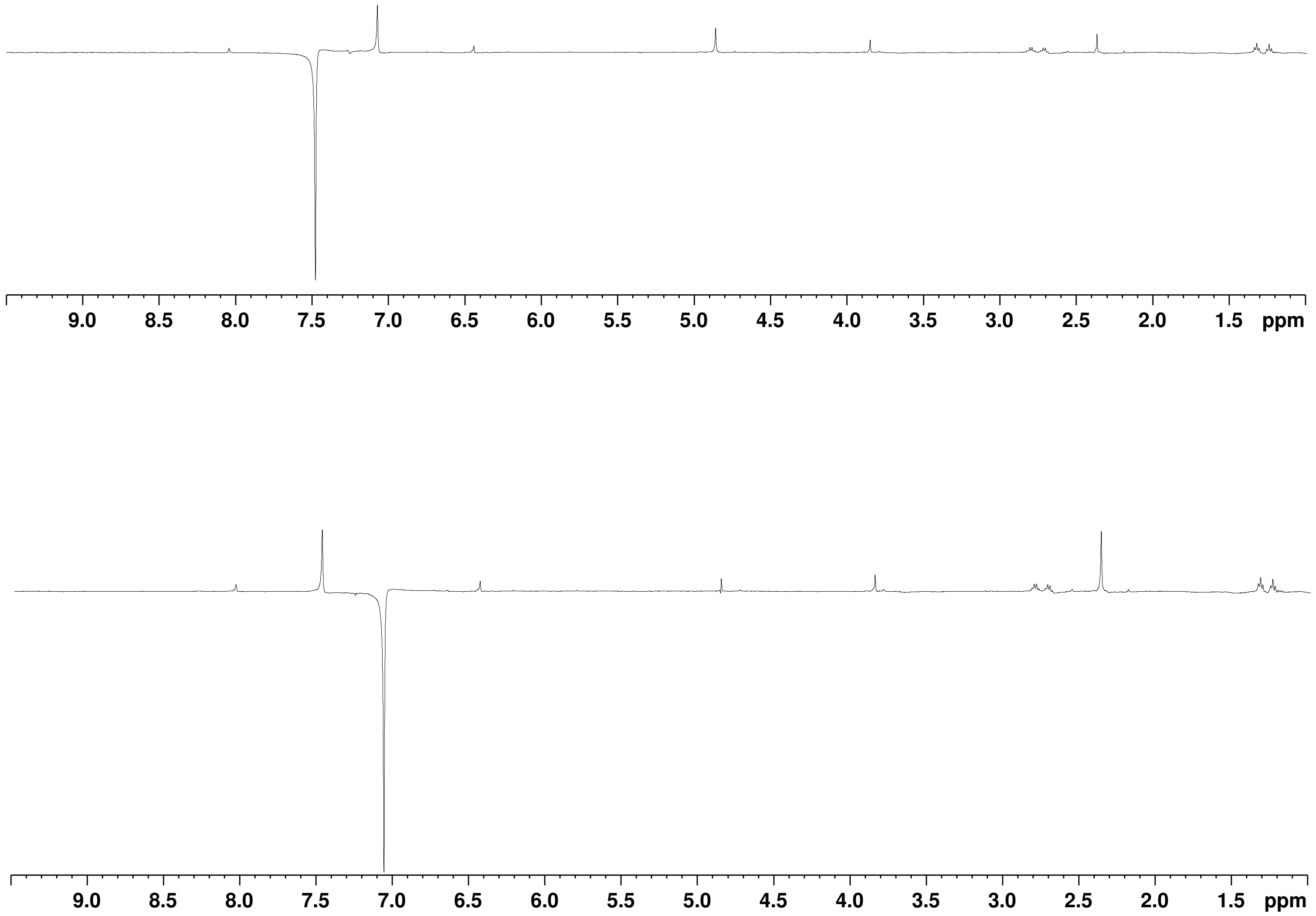

Figure S83. Selected nOe difference proton NMR spectra of **7c** in $CDCl_3$.

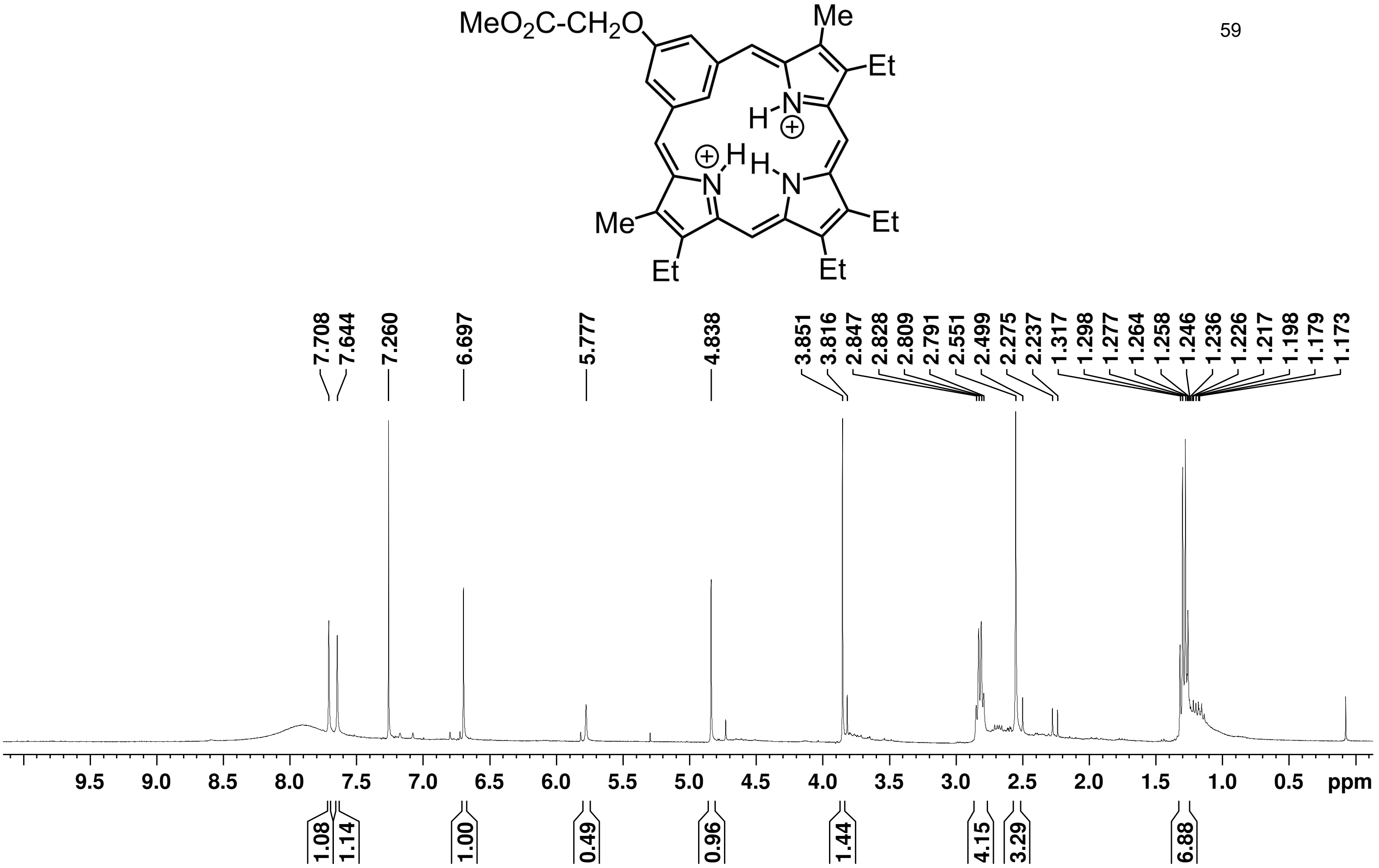

Figure S84. 500 MHz proton NMR spectrum of $\mathbf{7c}H_2^{2+}$ in TFA-CDCl$_3$.

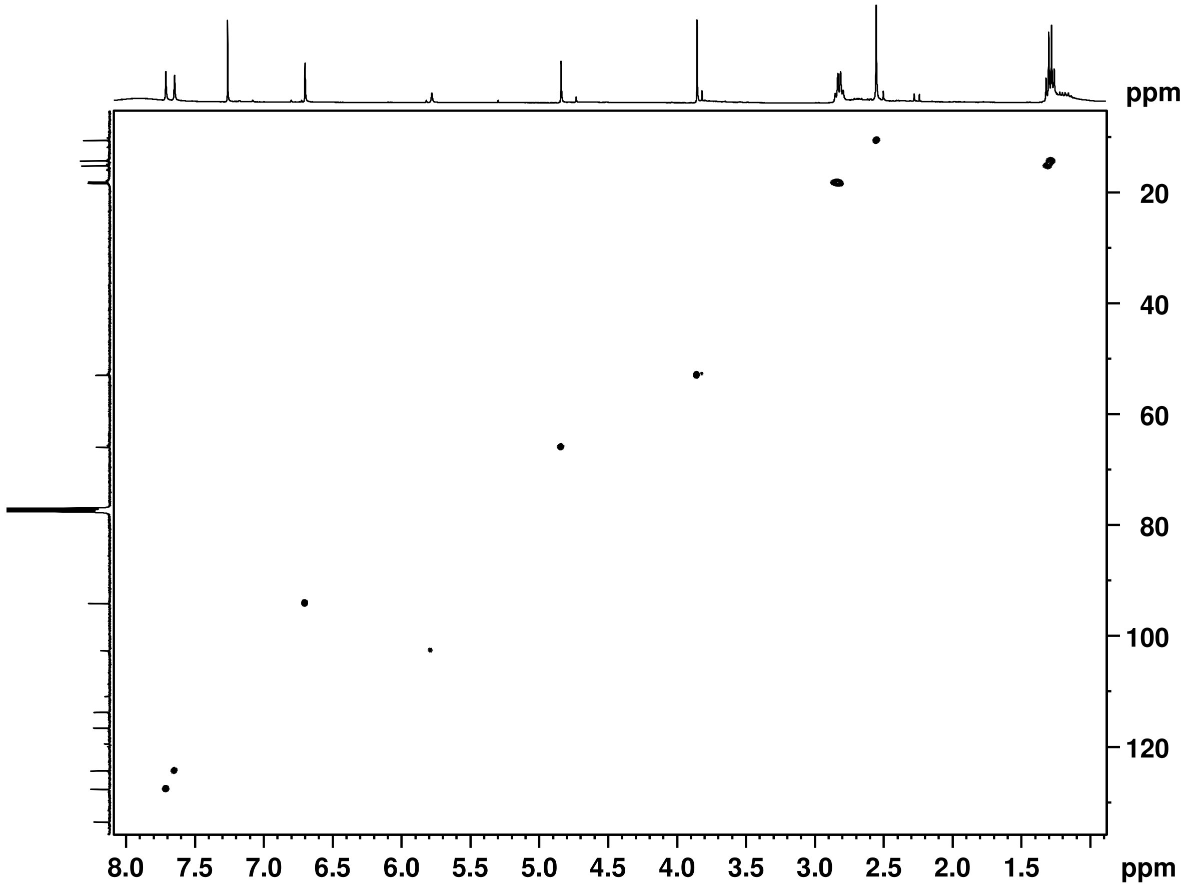

Figure S85. HSQC NMR spectrum of $\mathbf{7c}H_2^{2+}$ in TFA-CDCl$_3$.

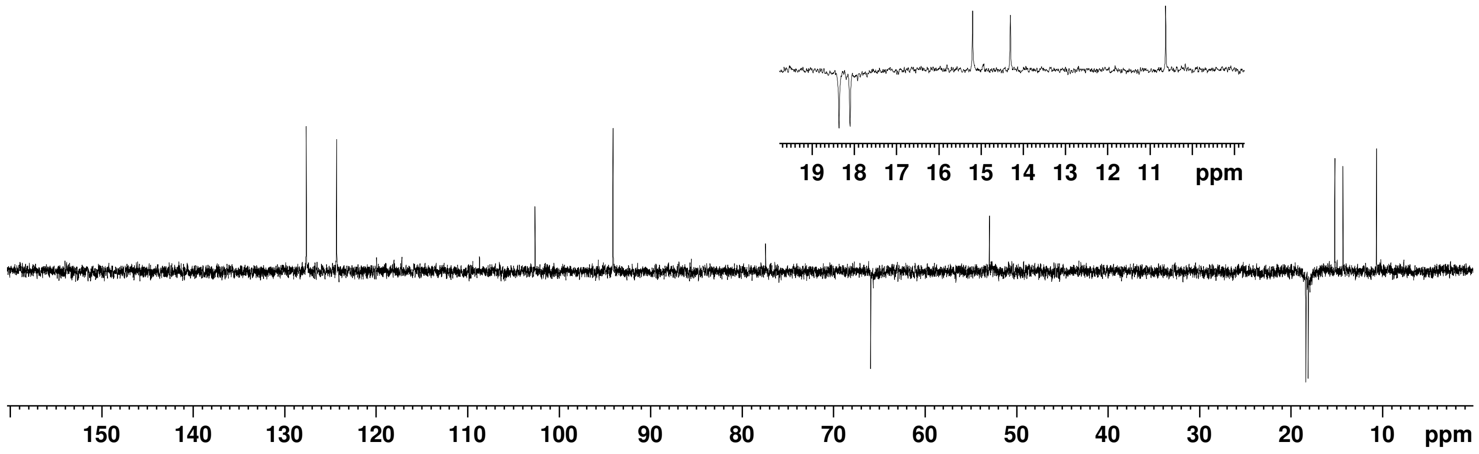

Figure S86. DEPT-135 NMR spectrum of **7c**H_2^{2+} in TFA-CDCl$_3$.

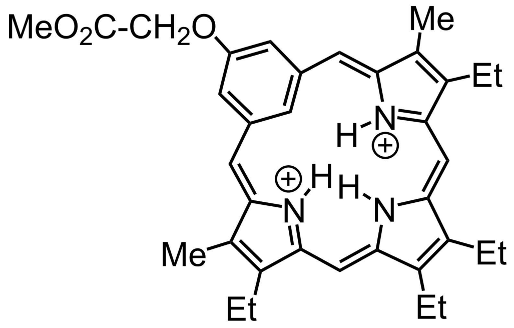

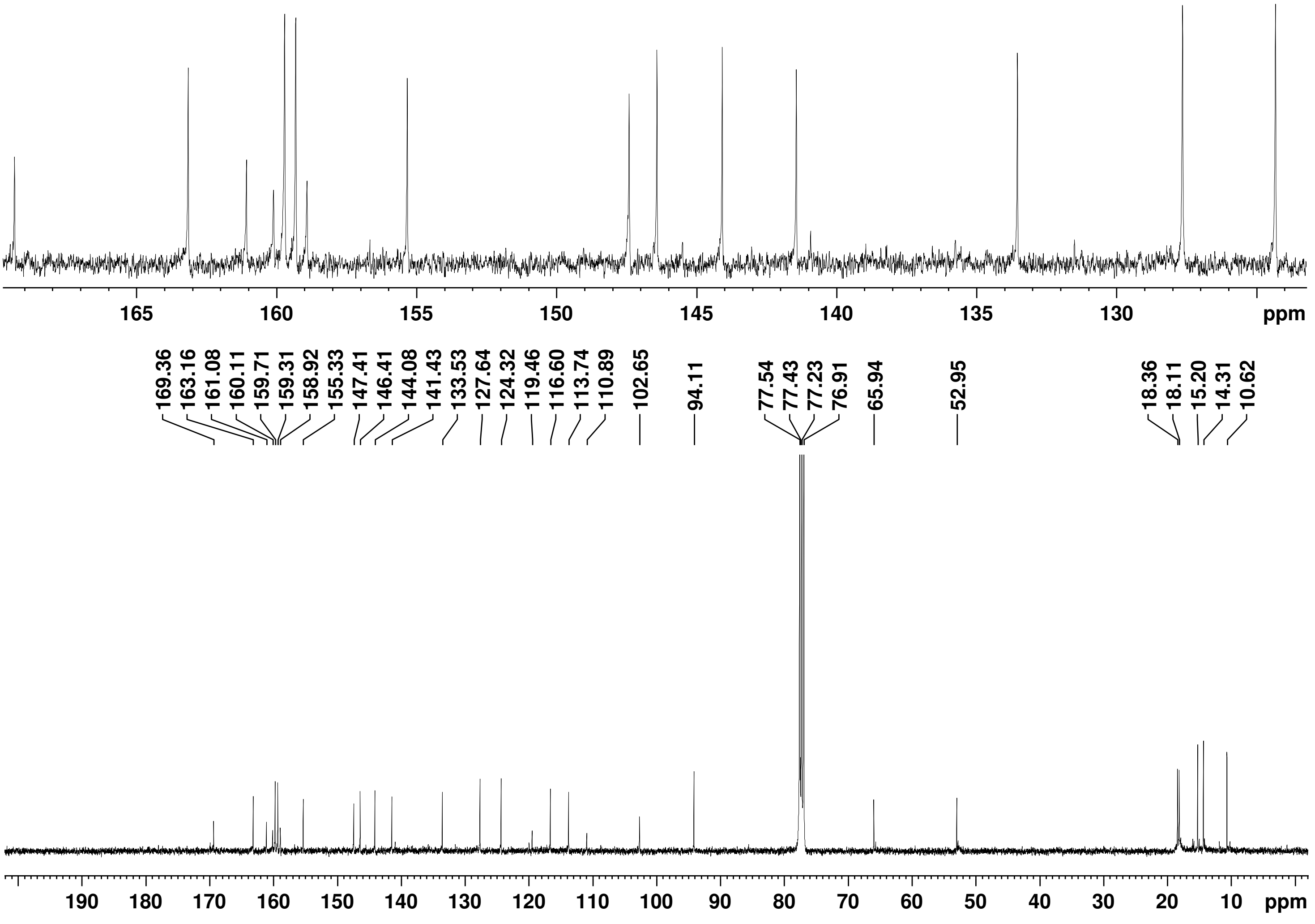

Figure S87. 125 MHz carbon-13 NMR spectrum of **7c**H_2^{2+} in TFA-CDCl$_3$.

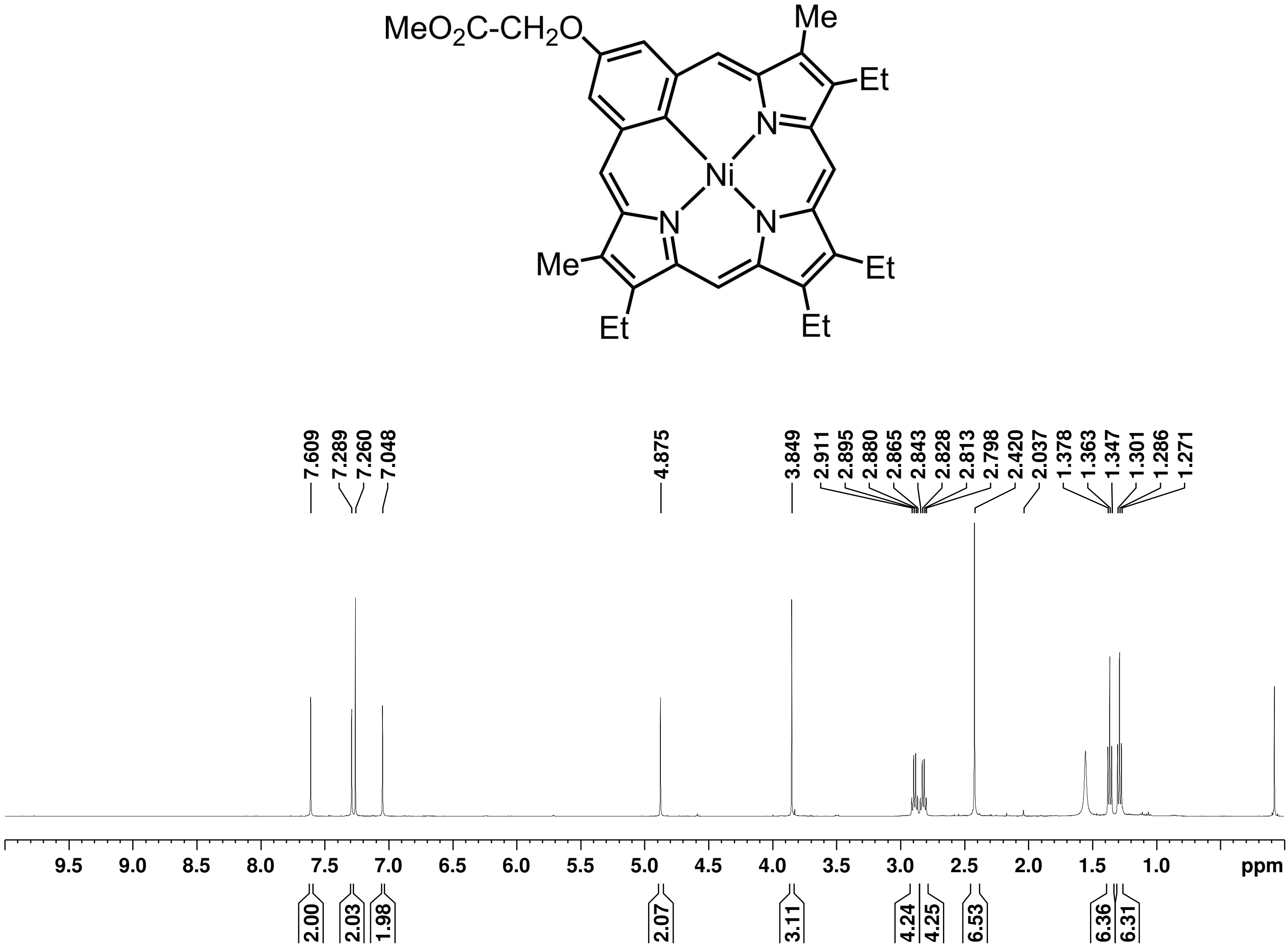

Figure S88. 500 MHz proton NMR spectrum of nickel(II) complex **7c**Ni in $CDCl_3$.

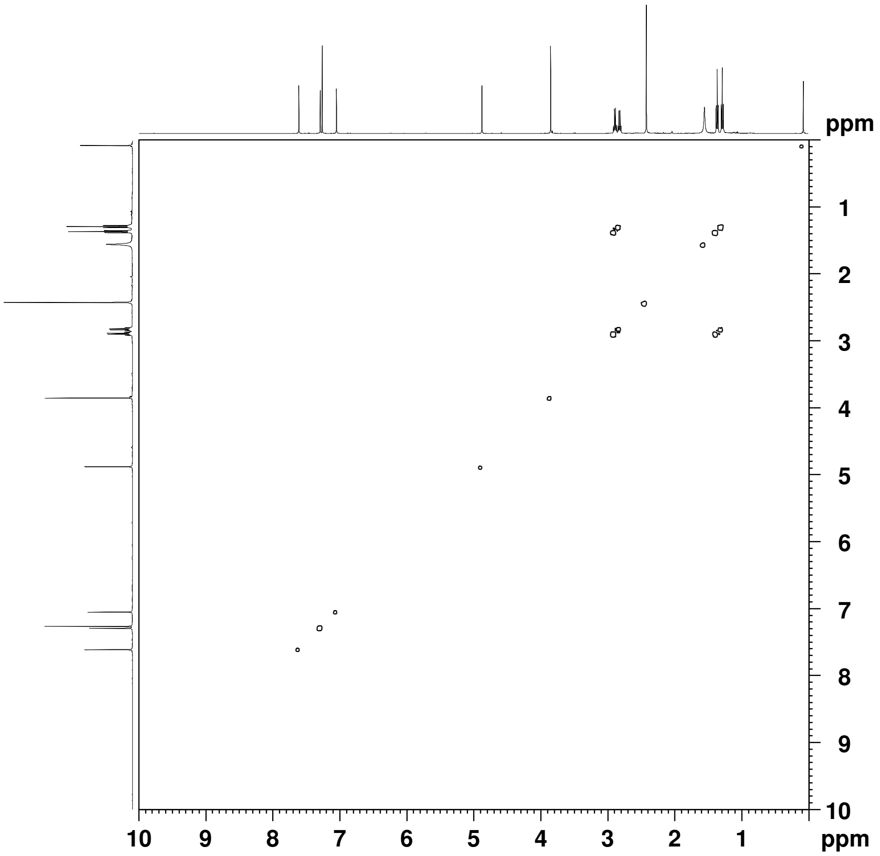

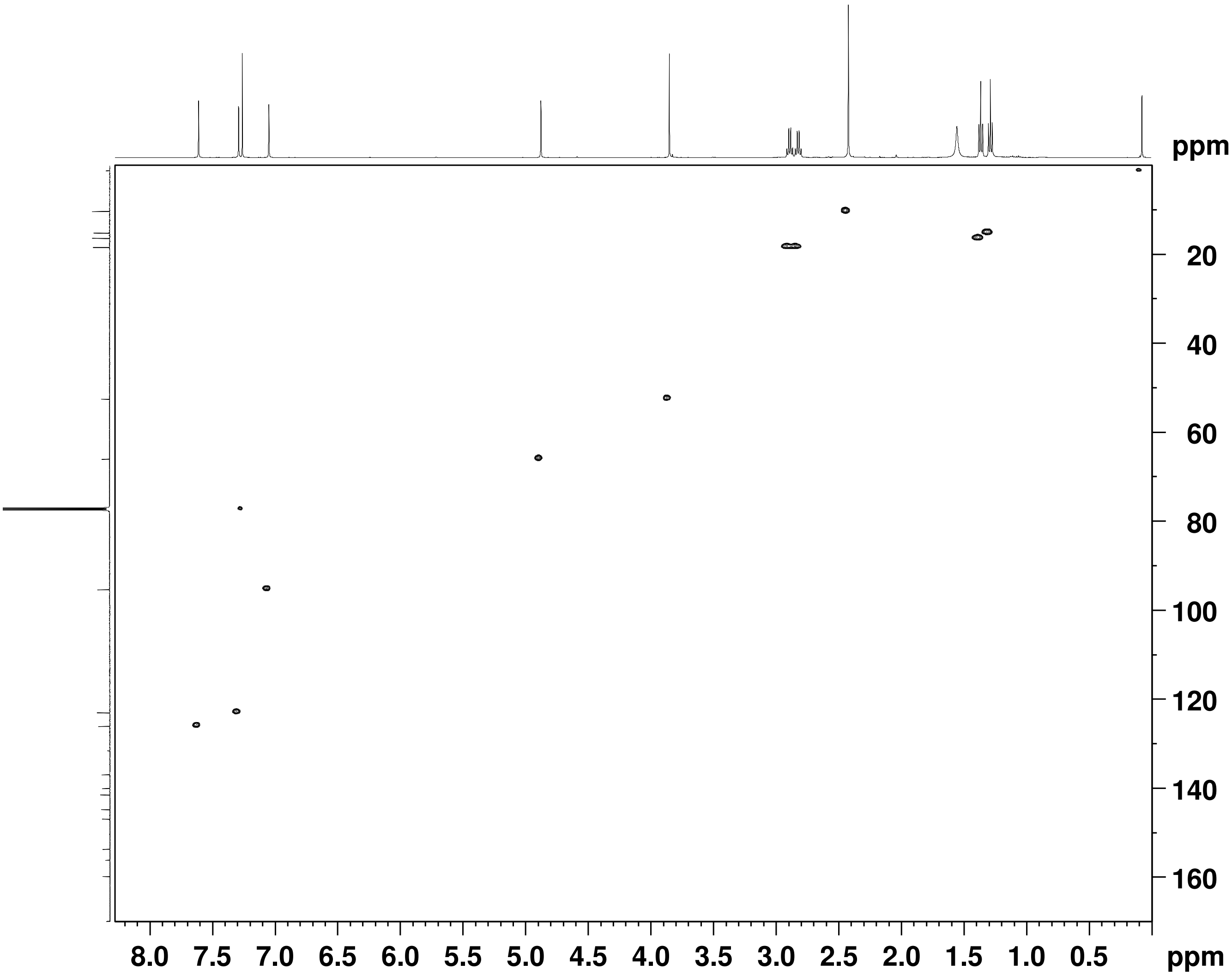

Figure S89. ^{1}H-^{1}H COSY (top) and HSQC (bottom) NMR spectra of **7cNi** in $CDCl_3$.

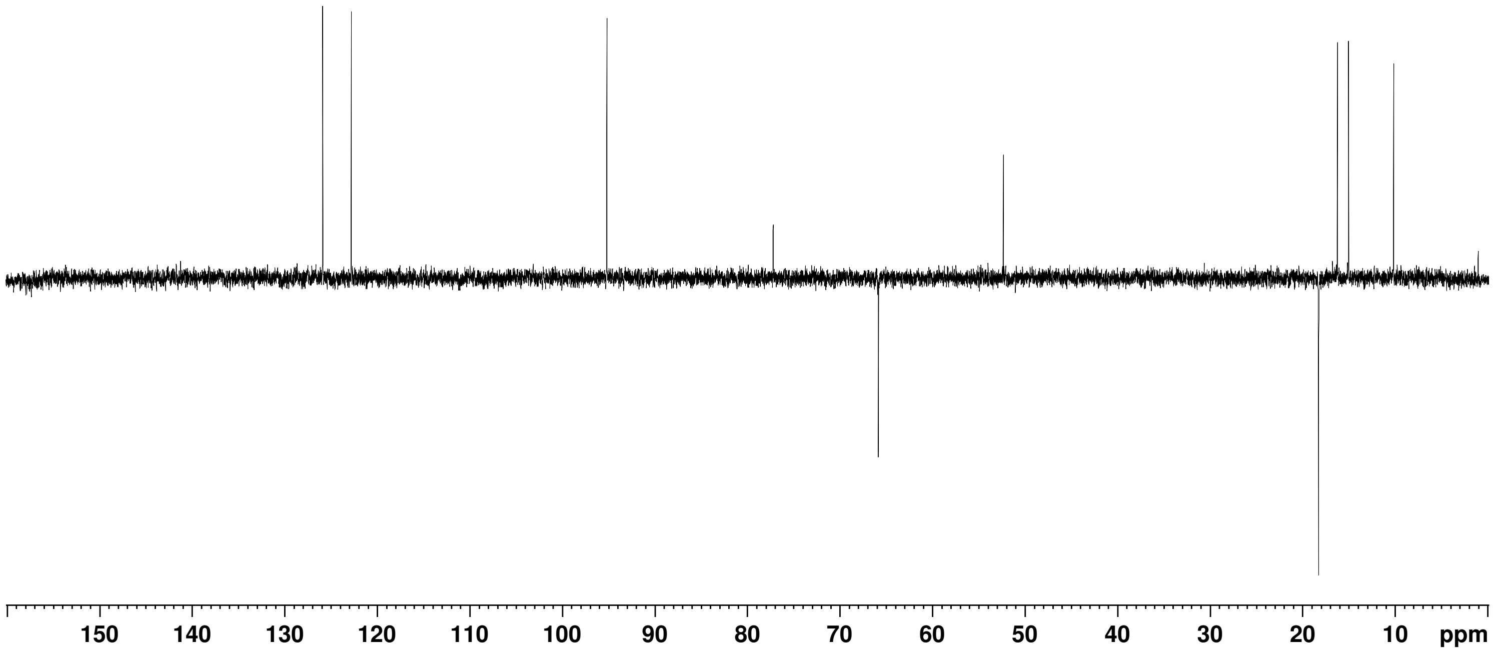

Figure S90. DEPT-135 NMR spectrum of **7cNi** in $CDCl_3$.

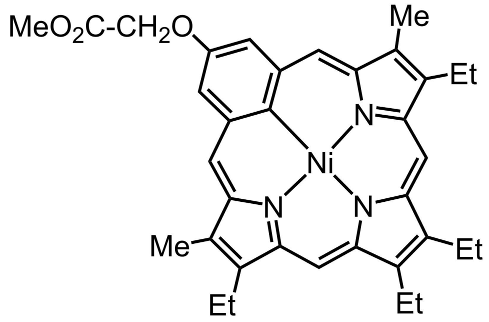

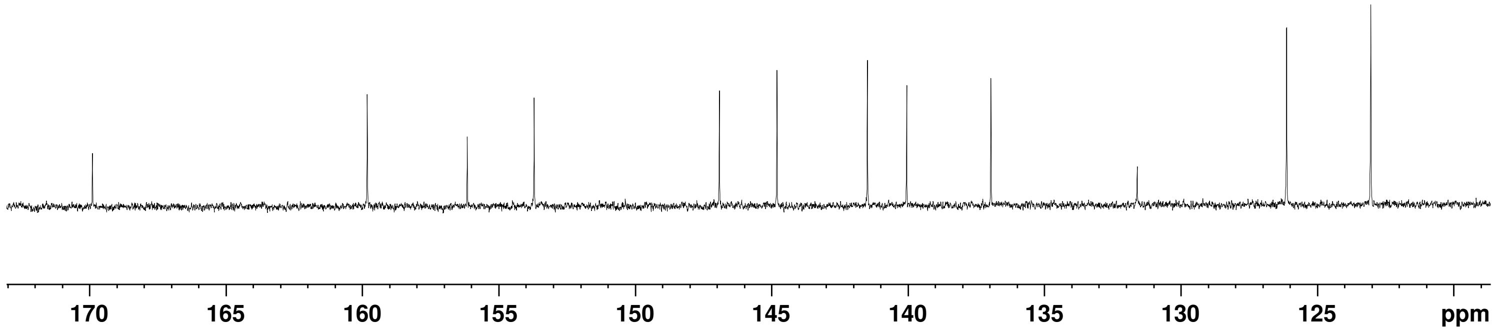

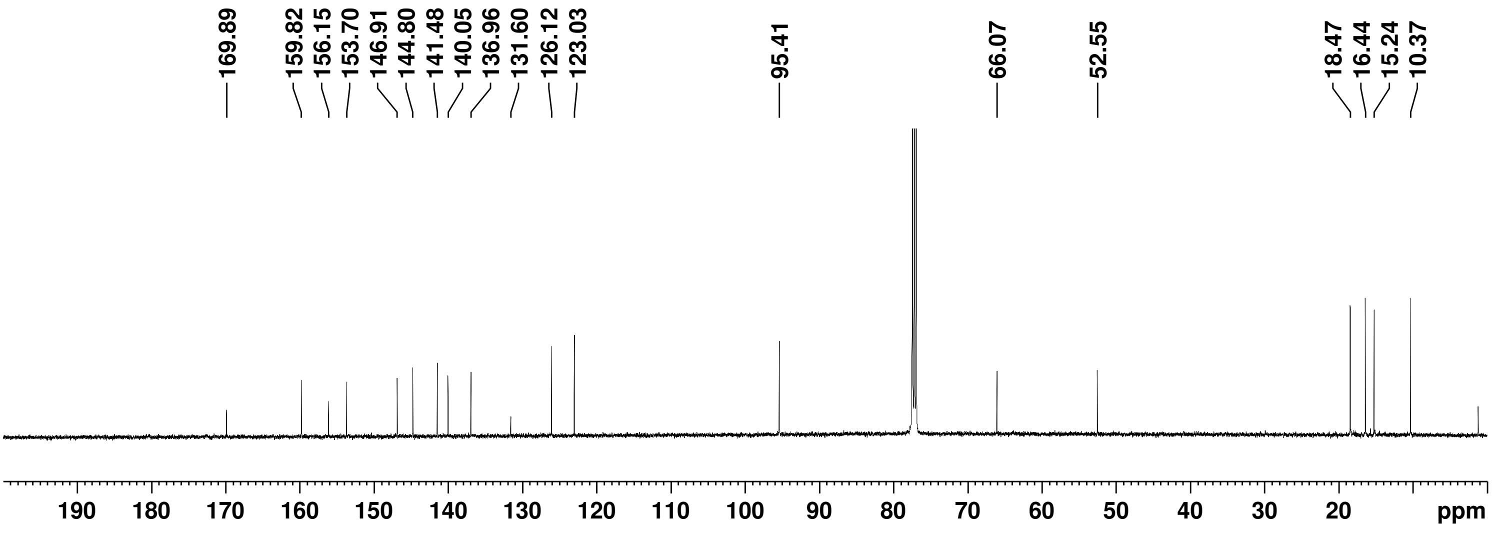

Figure S91. 125 MHz carbon-13 NMR spectrum of **7cNi** in $CDCl_3$.

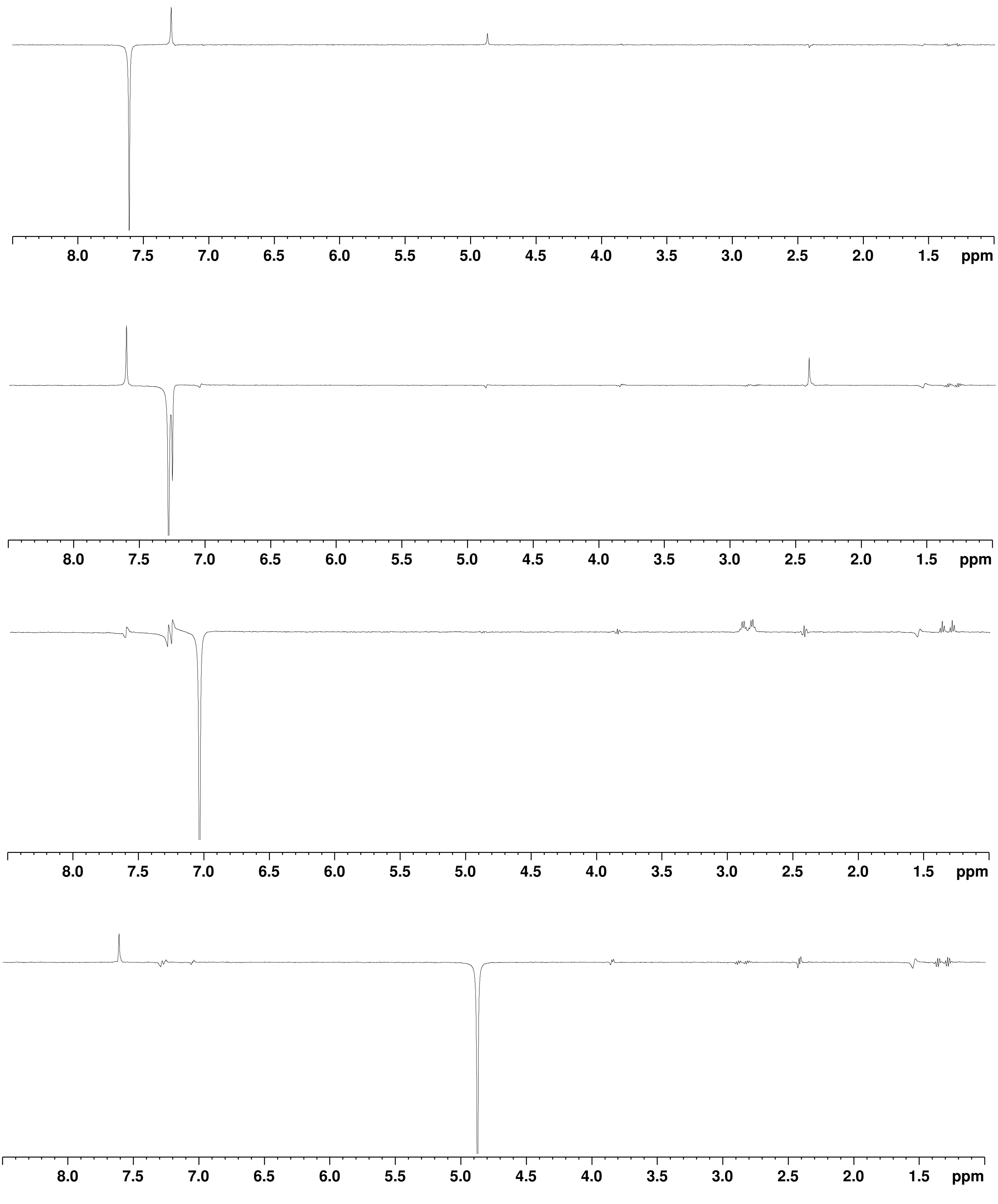

Figure S92. Selected nOe difference proton NMR spectra of **7cNi** in $CDCl_3$.

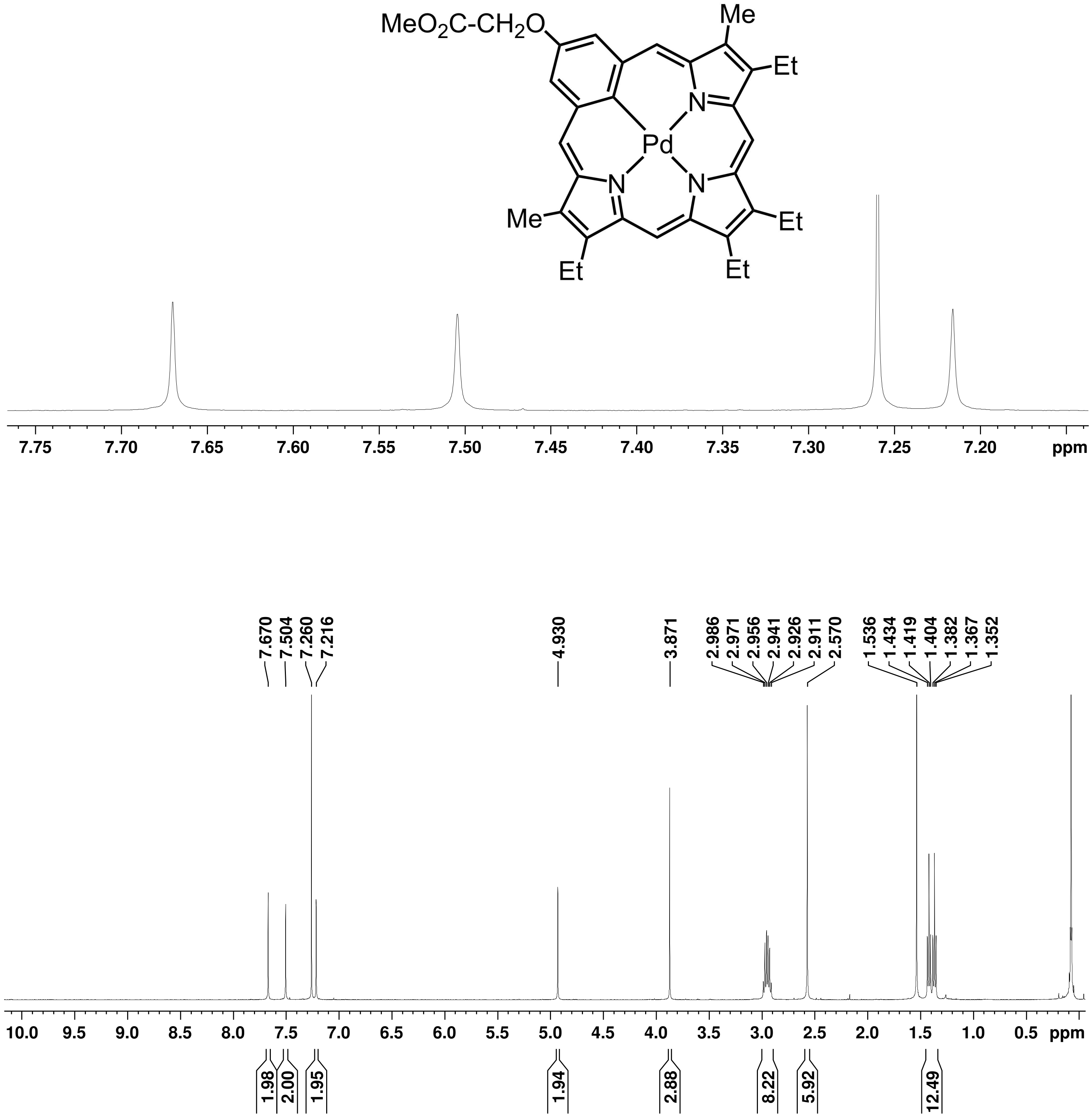

Figure S93. 500 MHz proton NMR spectrum of palladium(II) complex **7cPd** in $CDCl_3$.

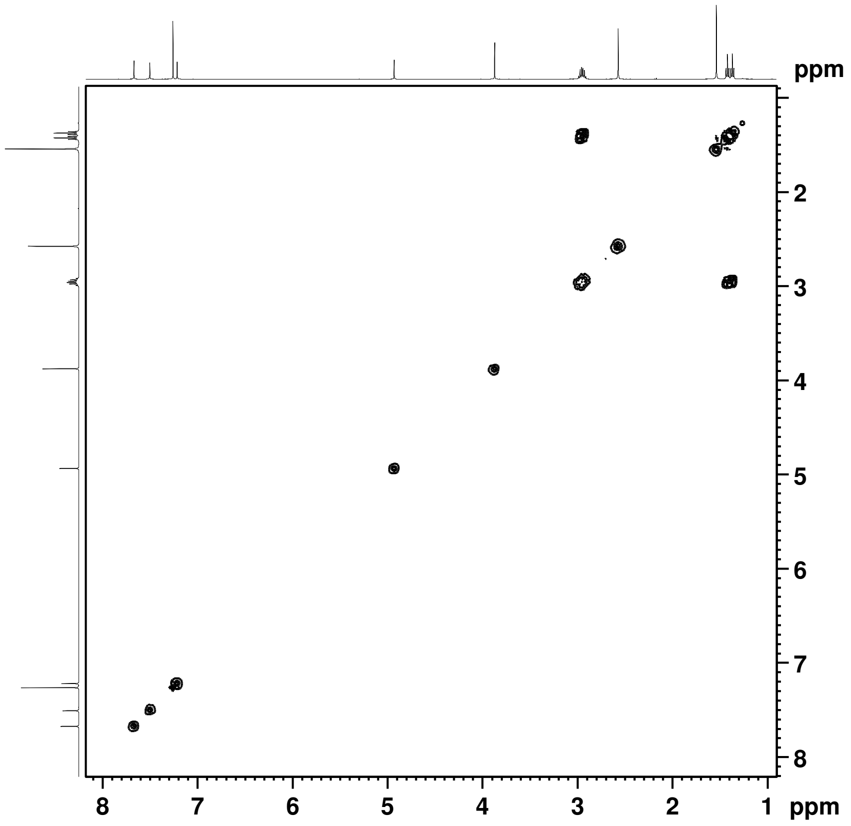

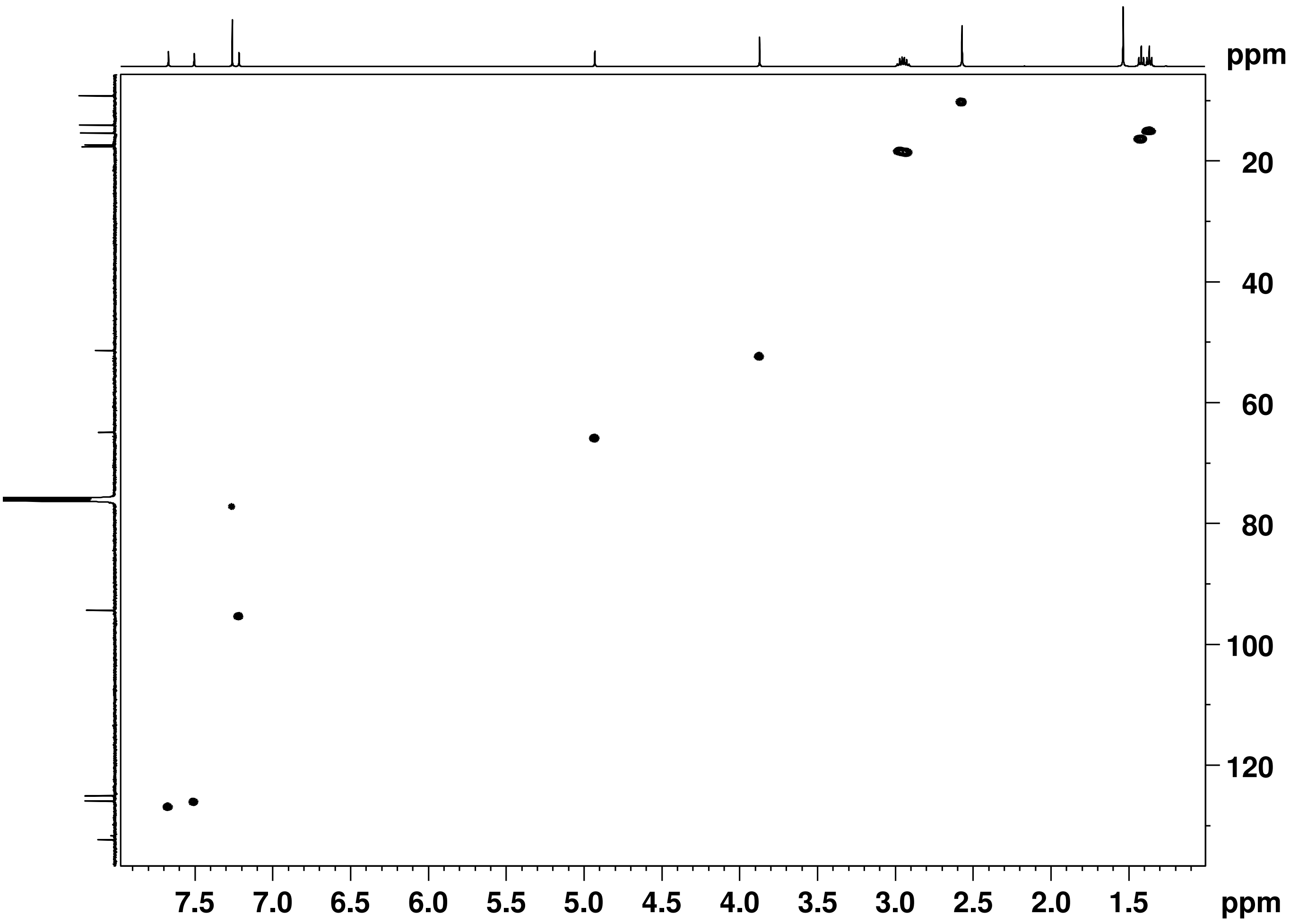

Figure S94. ^{1}H-^{1}H COSY (top) and HSQC (bottom) spectra of **7cPd** in $CDCl_3$.

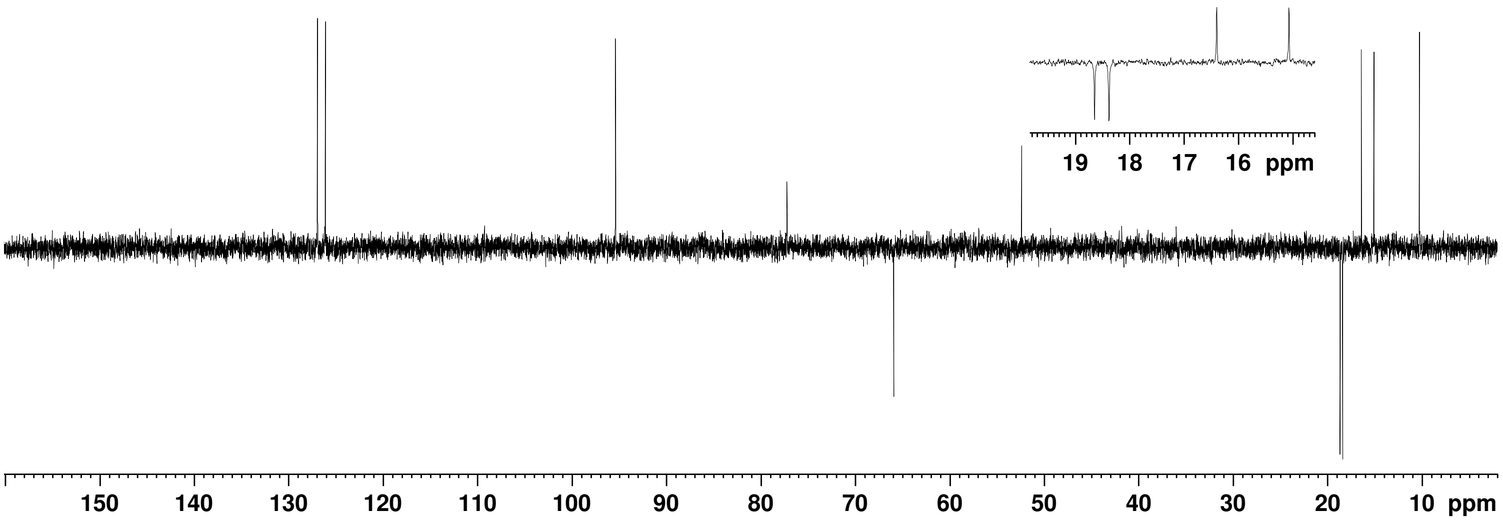

Figure S95. DEPT-135 NMR spectrum of **7cPd** in $CDCl_3$.

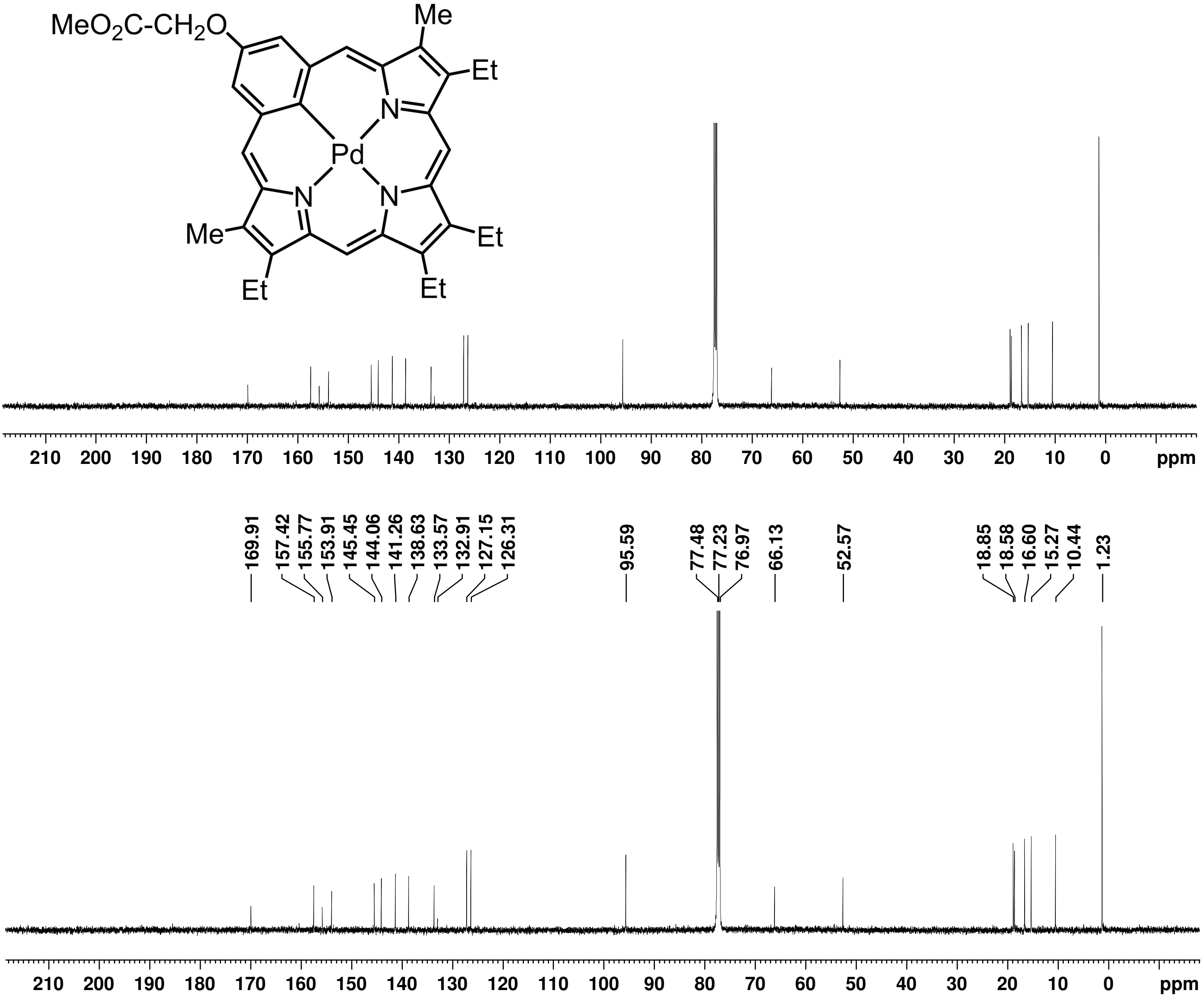

Figure S96. 125 MHz carbon-13 NMR spectrum of **7cPd** in $CDCl_3$.

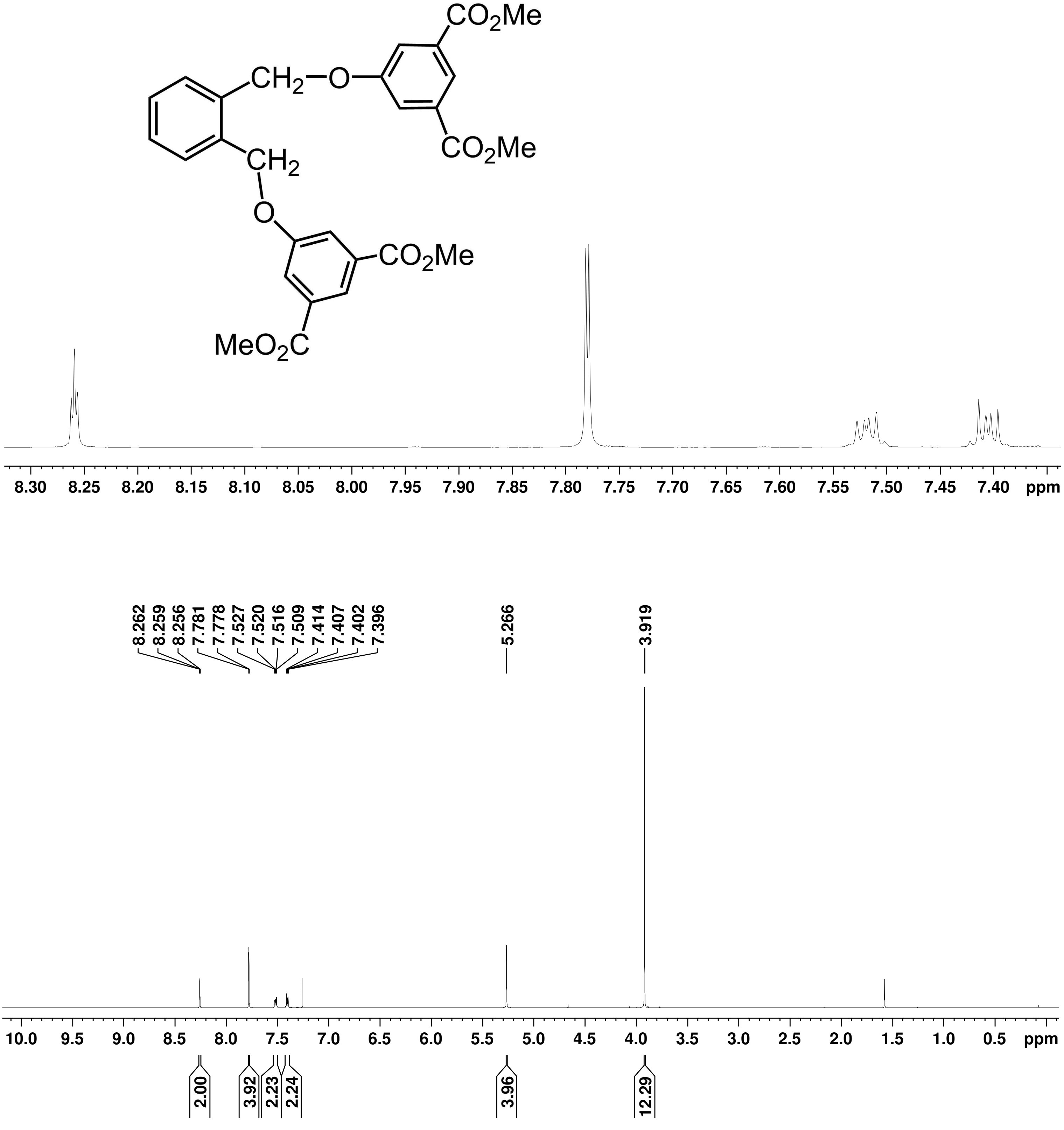

Figure S97. 500 MHz proton NMR spectrum of tetraester **16a** in $CDCl_3$.

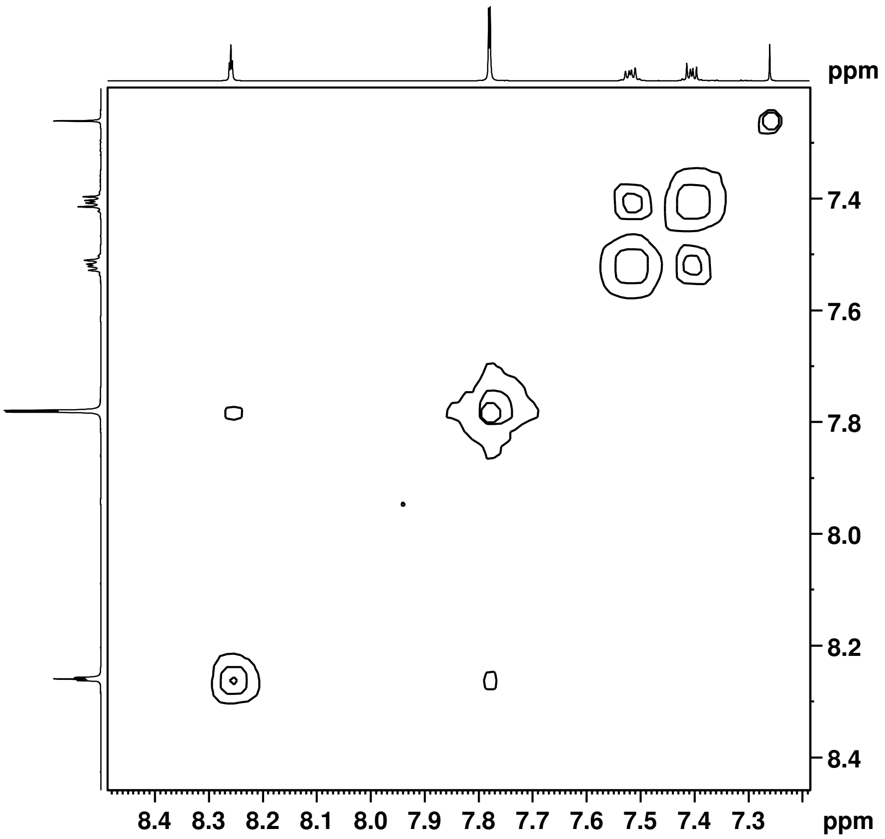

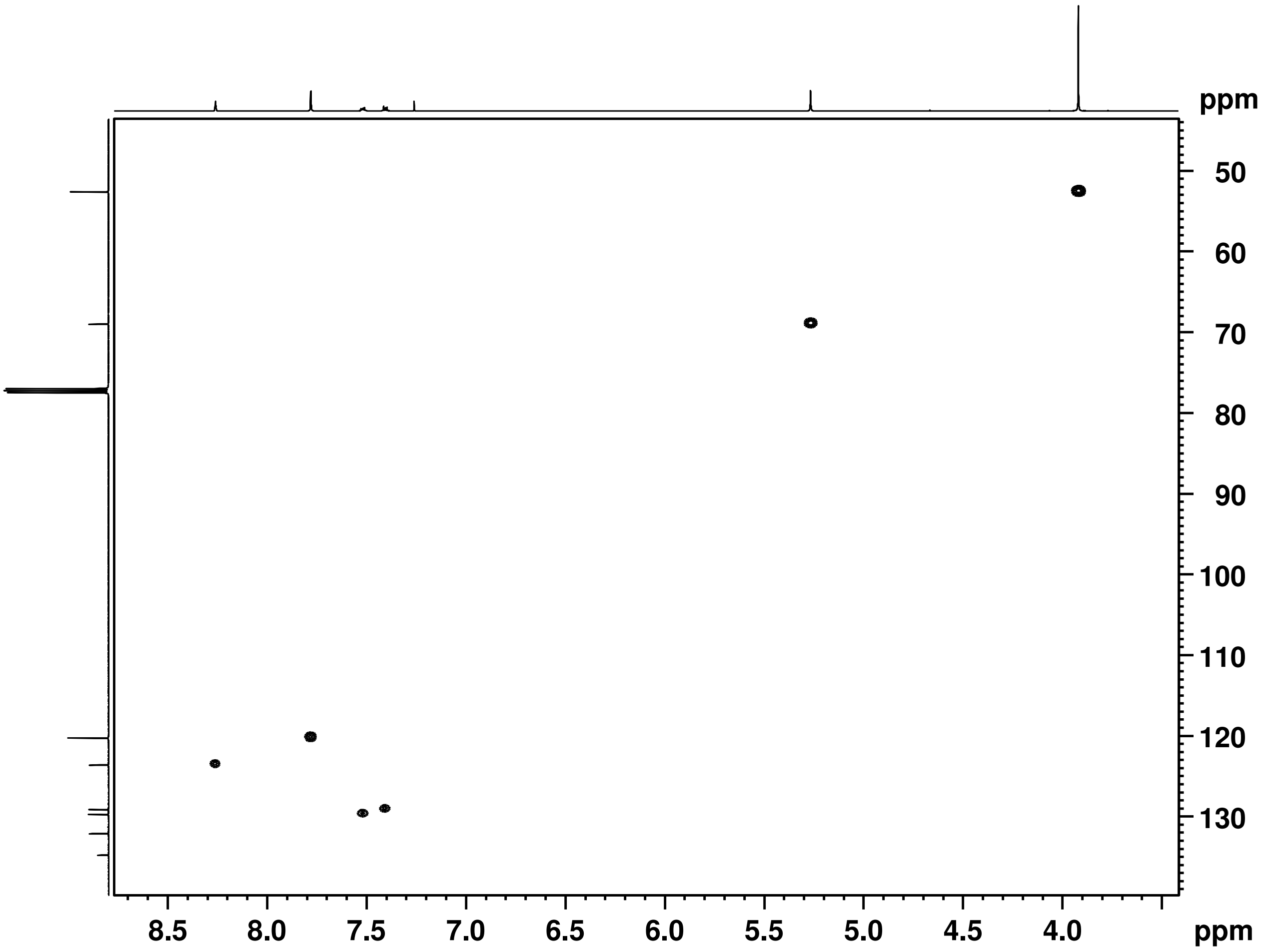

Figure S98. ^{1}H-^{1}H COSY and HSQC NMR spectra of **16a** in $CDCl_3$.

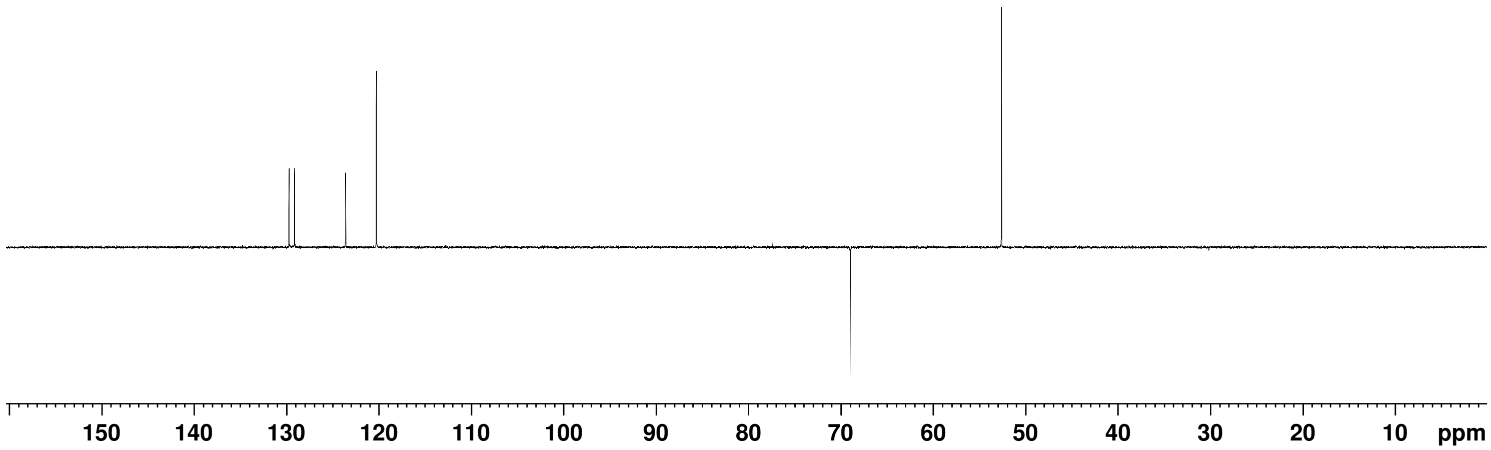

Figure S99. DEPT-135 NMR spectrum of **16a** in $CDCl_3$.

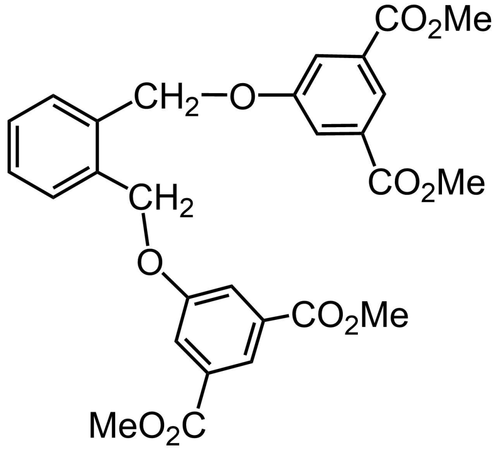

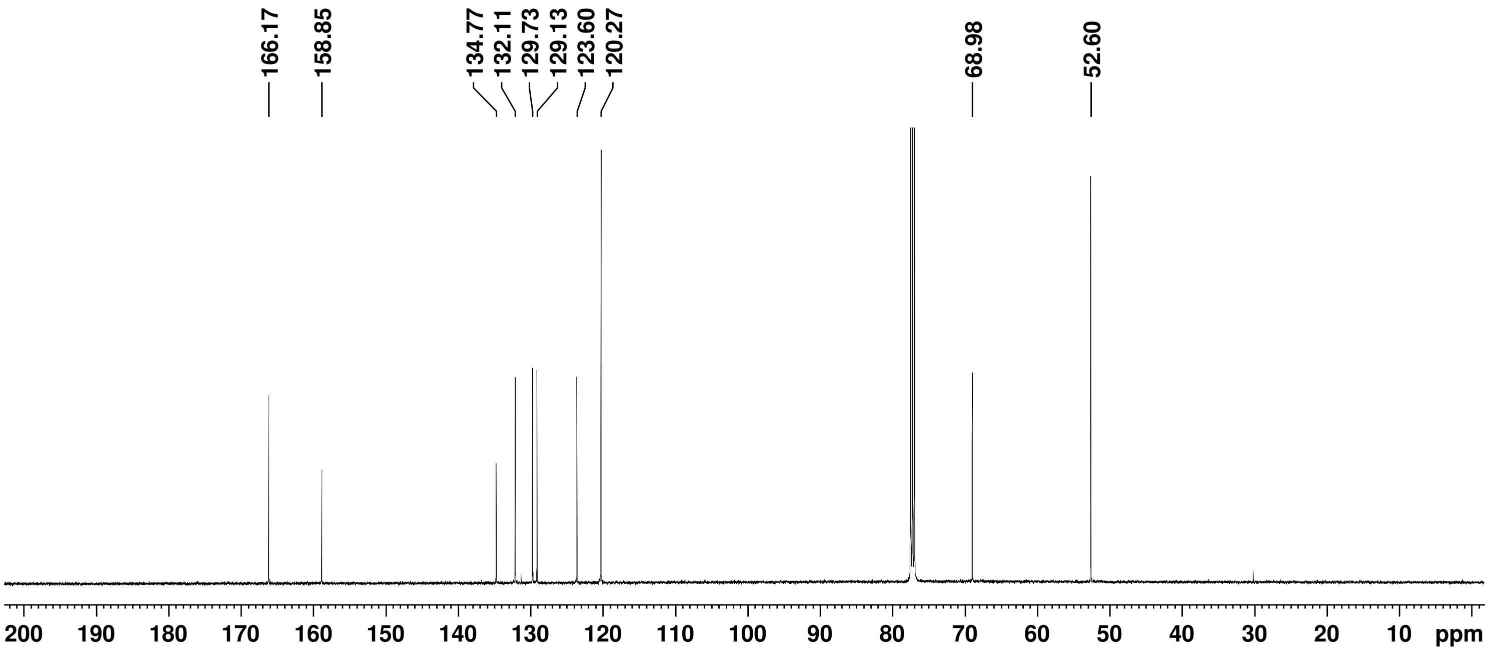

Figure S100. 125 MHz carbon-13 NMR spectrum of **16a** in $CDCl_3$.

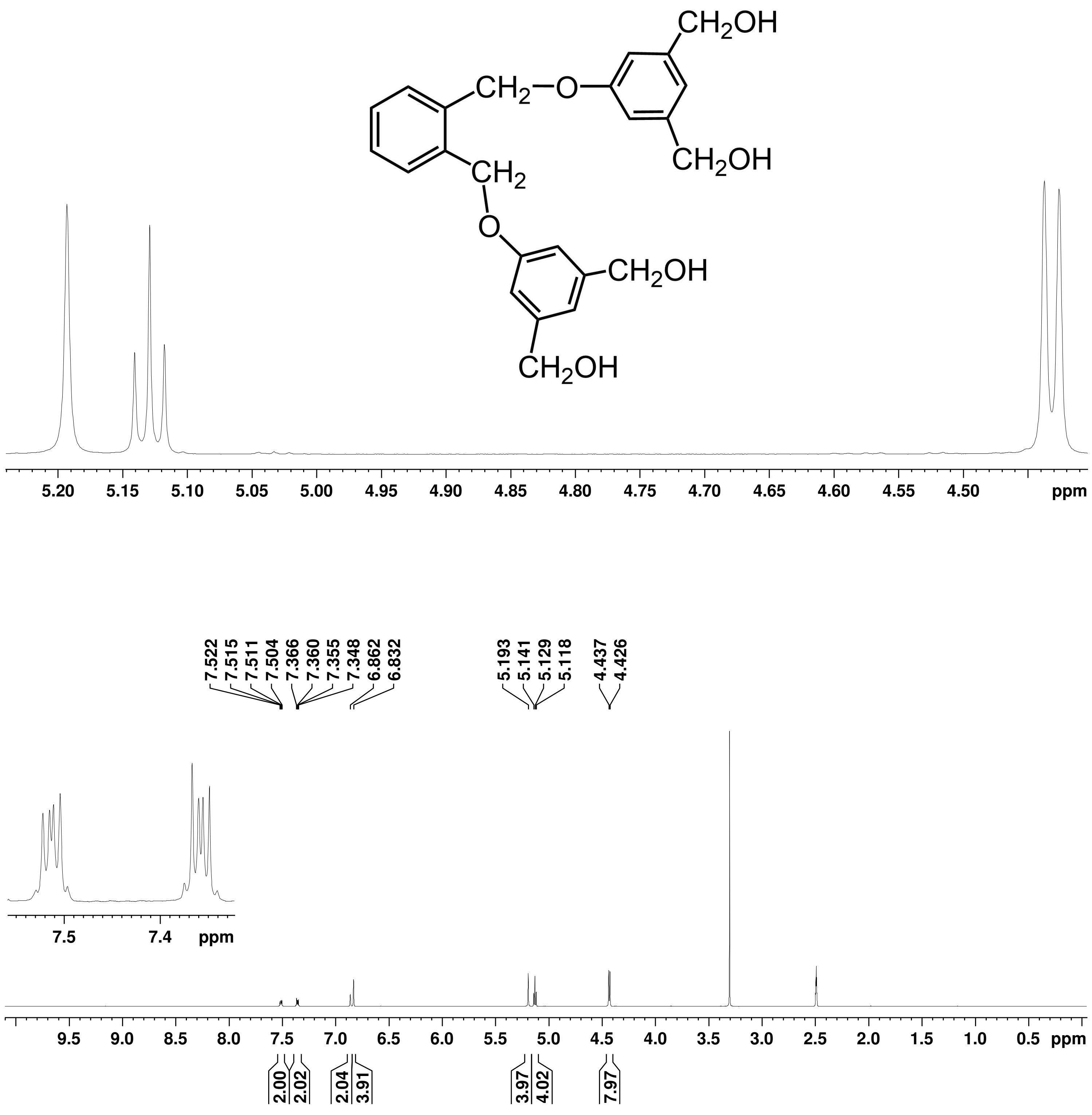

Figure S101. 500 MHz proton NMR spectrum of tetraalcohol **17a** in DMSO-d_6.

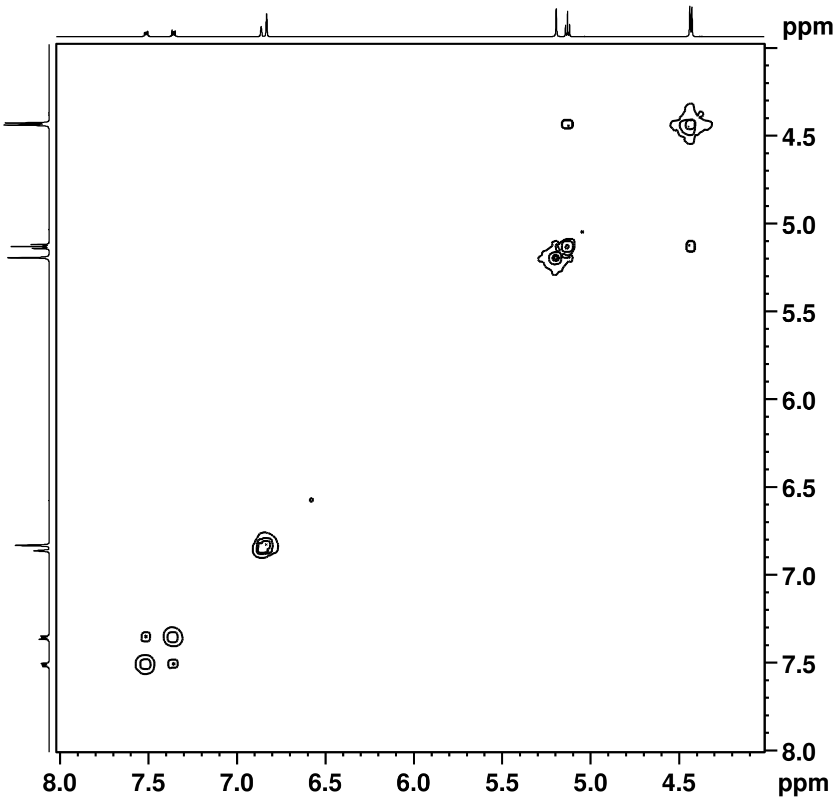

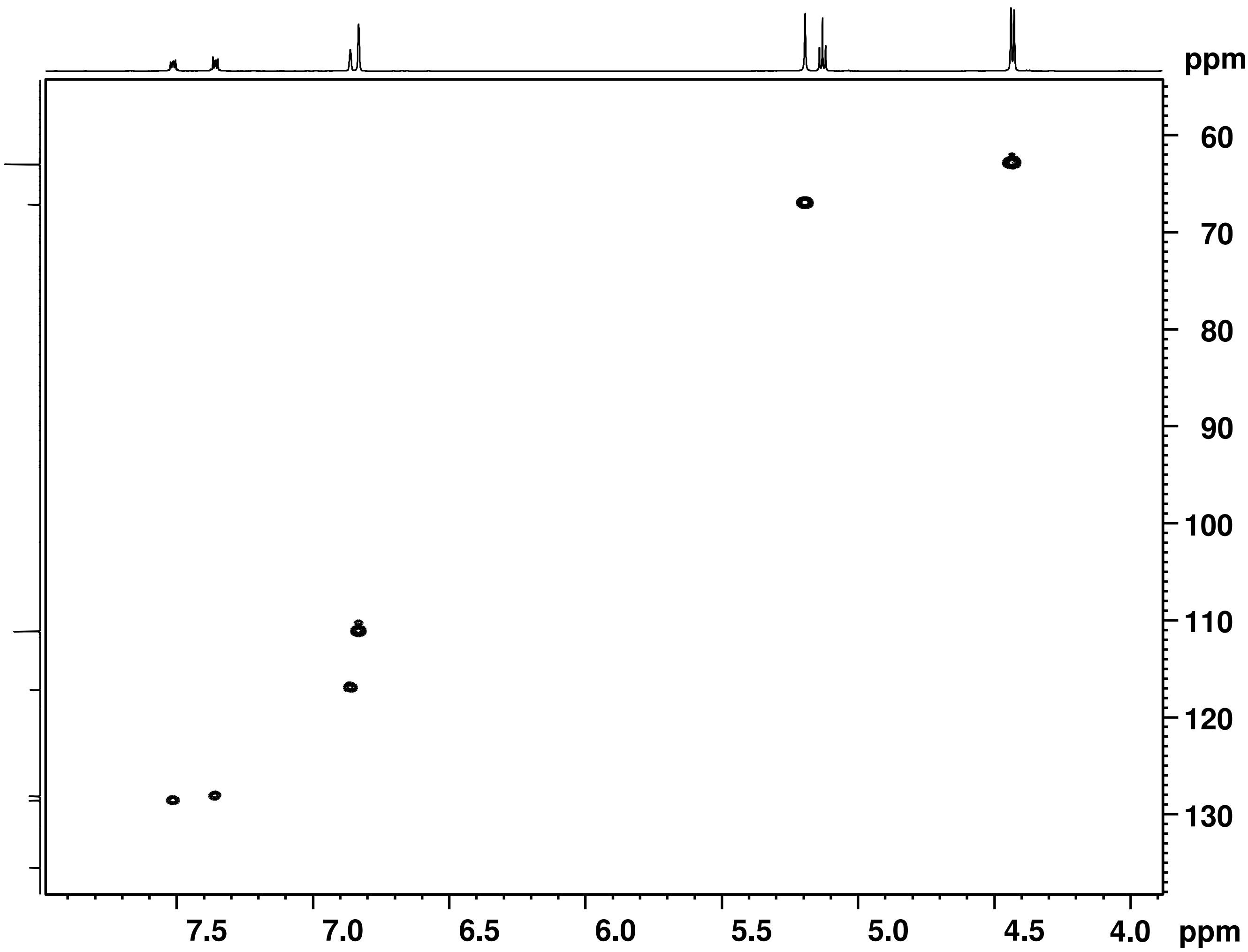

Figure S102. ^{1}H-^{1}H COSY (top) and HSQC (bottom) NMR spectra of **17a** in DMSO-d_6.

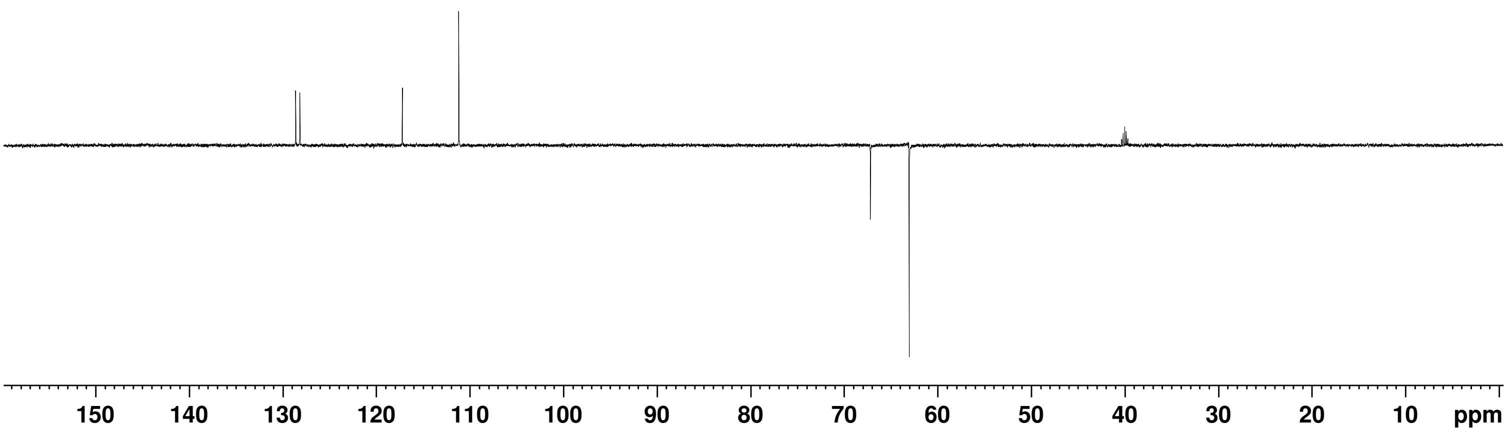

Figure 103. DEPT-135 NMR spectrum of **17a** in DMSO-d_6.

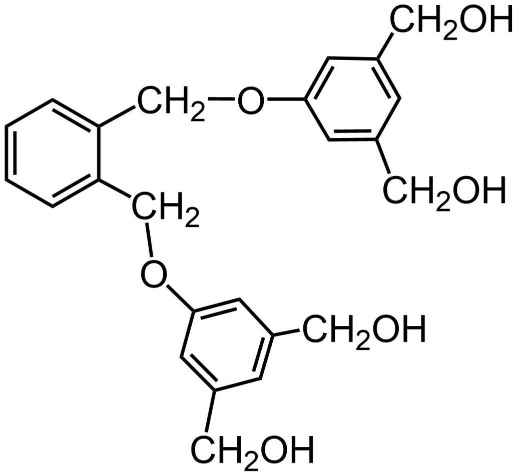

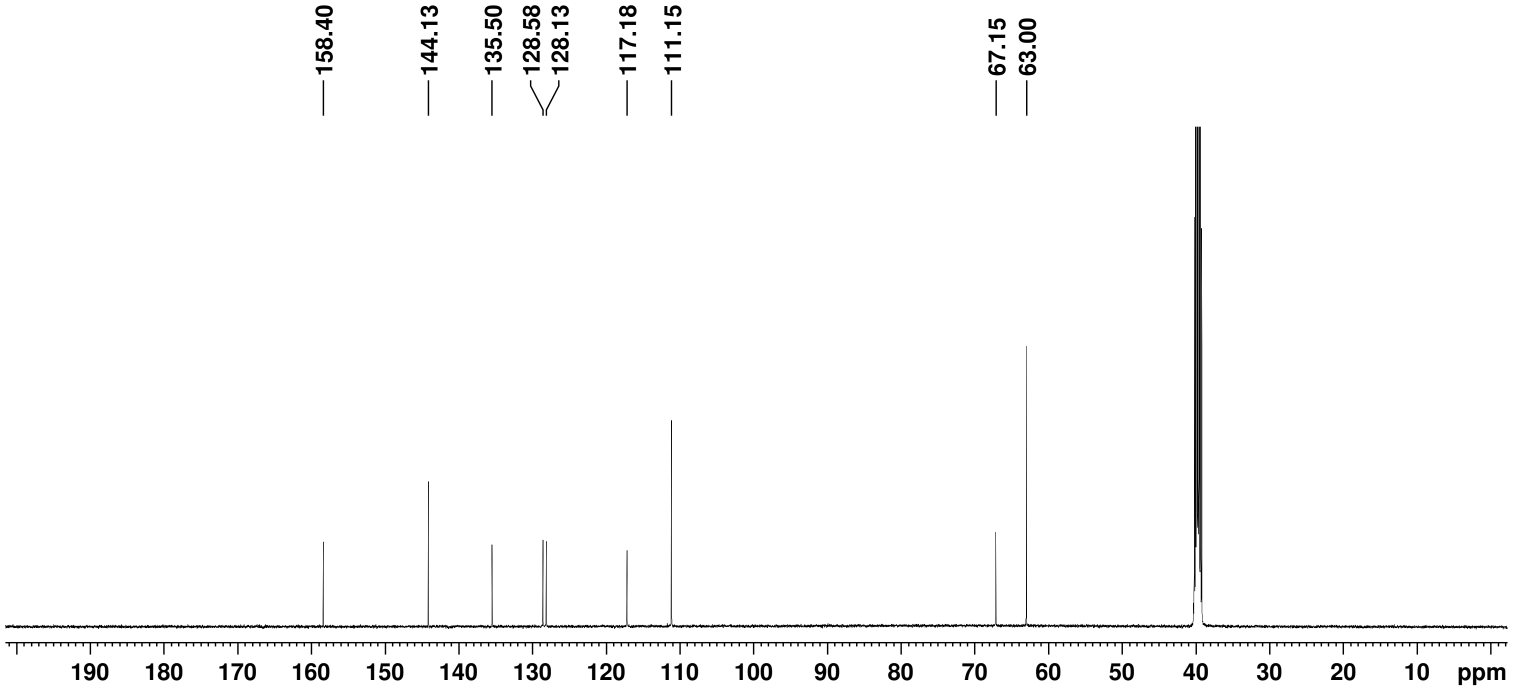

Figure S104. 125 MHz carbon-13 NMR spectrum of **17a** in DMSO-d_6.

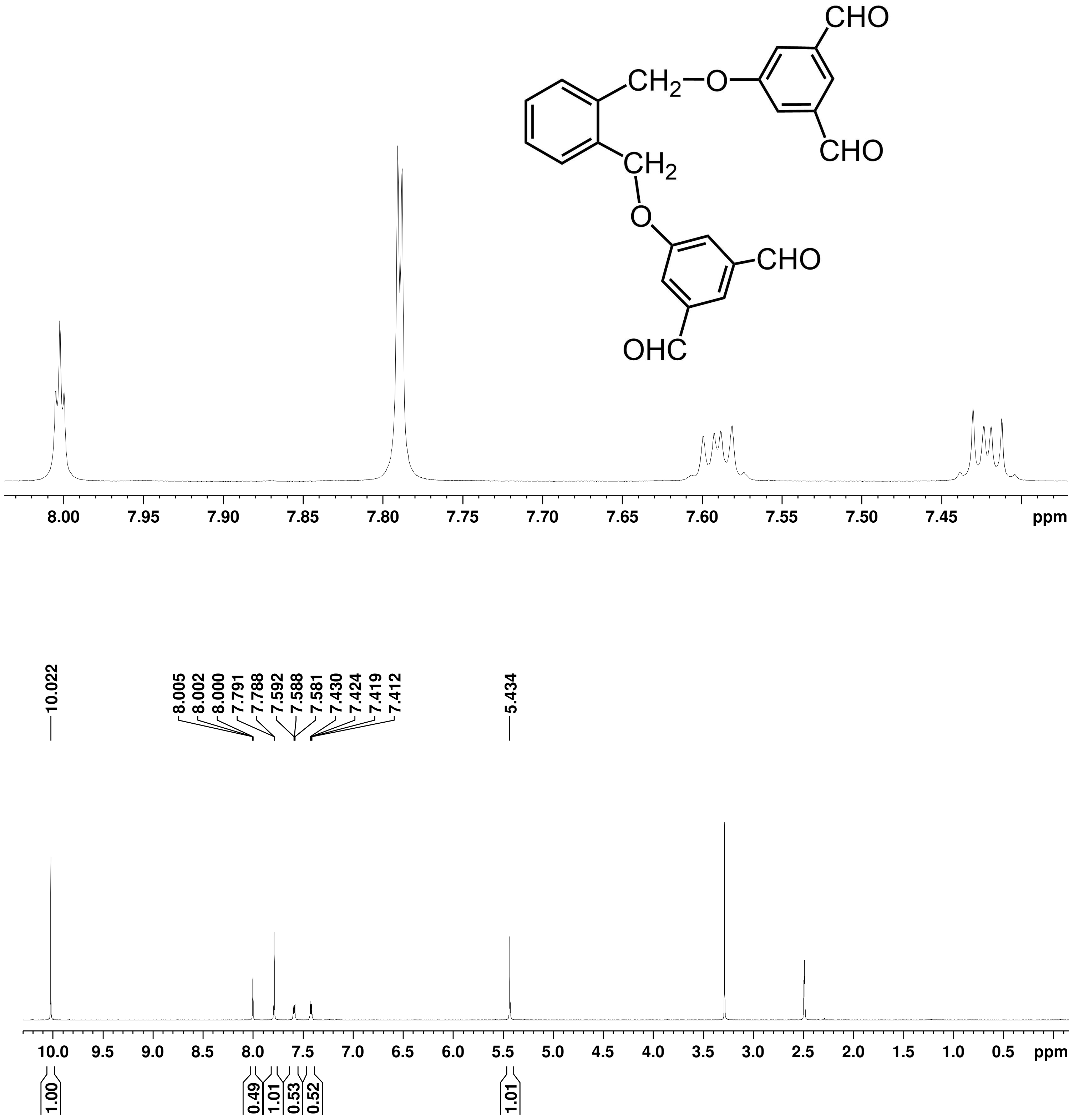

Figure S105. Proton NMR spectrum of tetraaldehyde **18a** in DMSO-d_6.

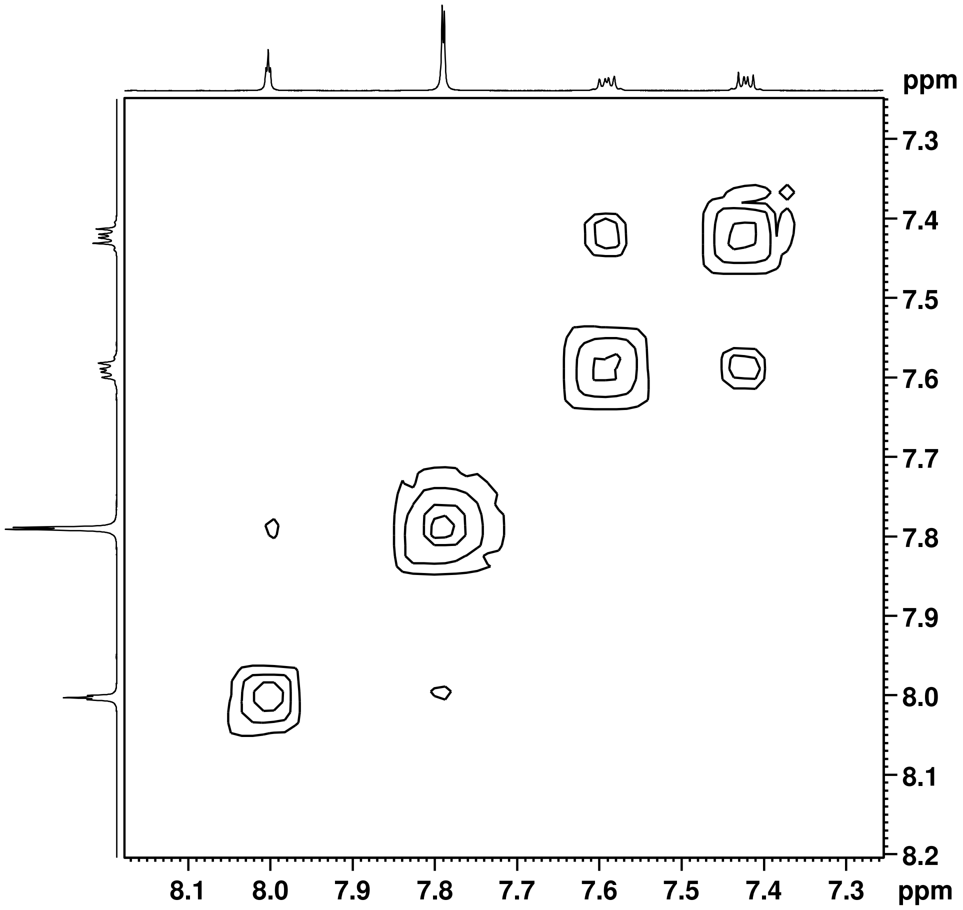

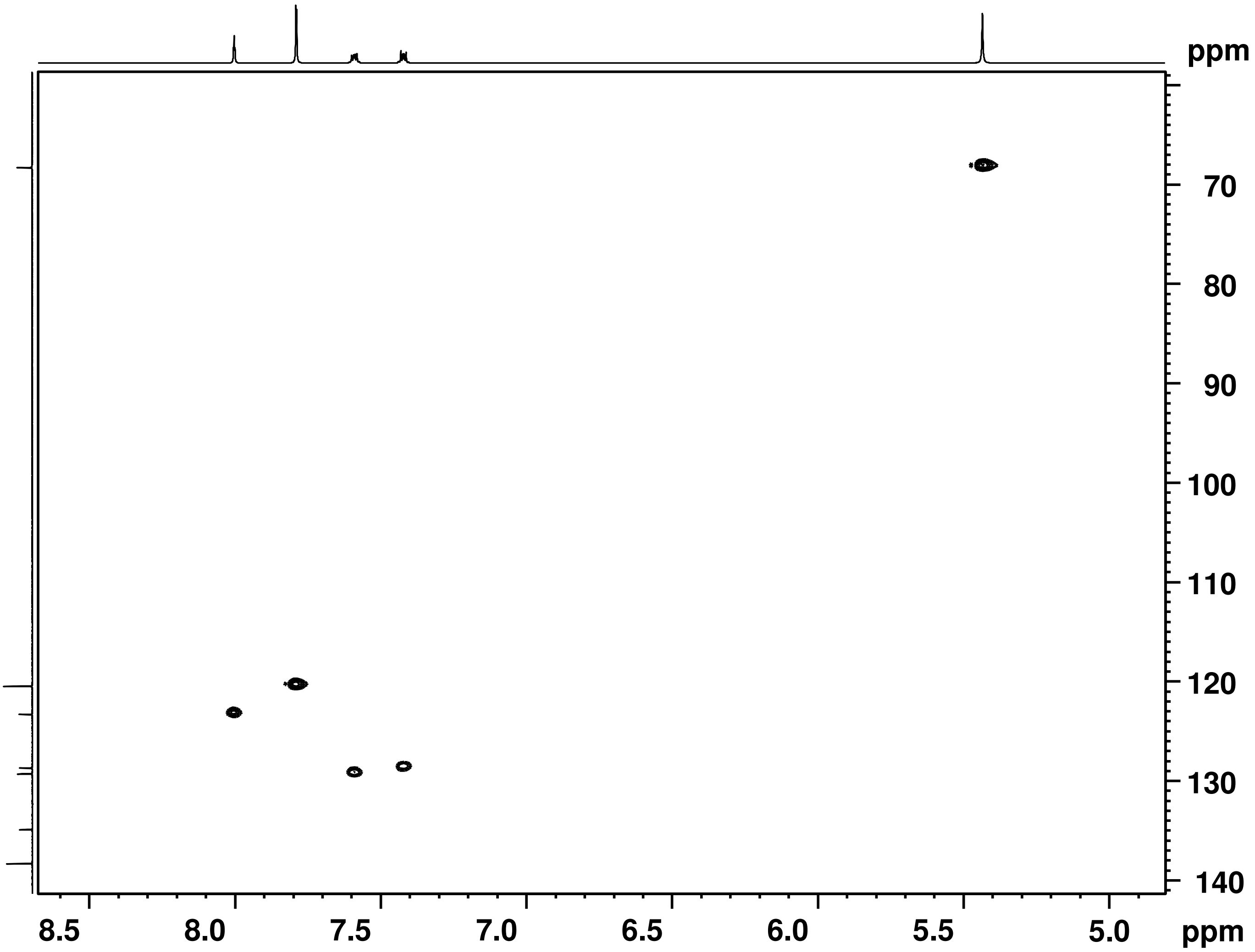

Figure S106. ^{1}H-^{1}H COSY (top) and HSQC (bottom) NMR spectra of **18a** in DMSO-d_6.

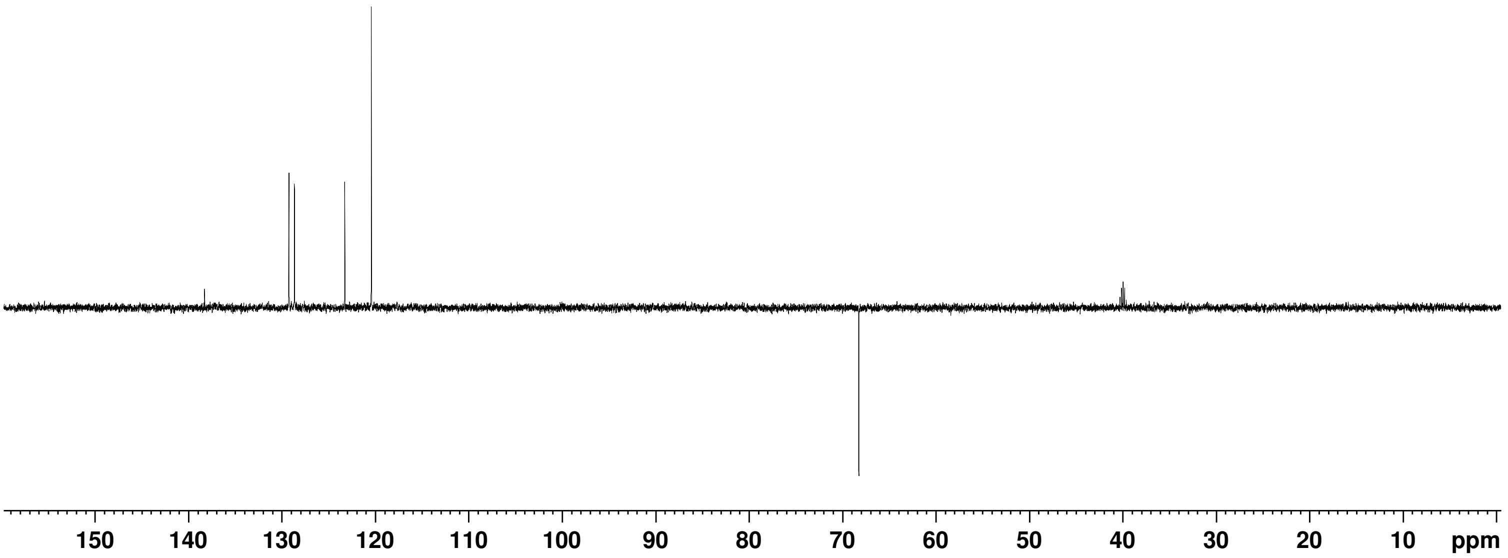

Figure S107. DEPT-135 NMR spectrum of **18a** in DMSO-d_6.

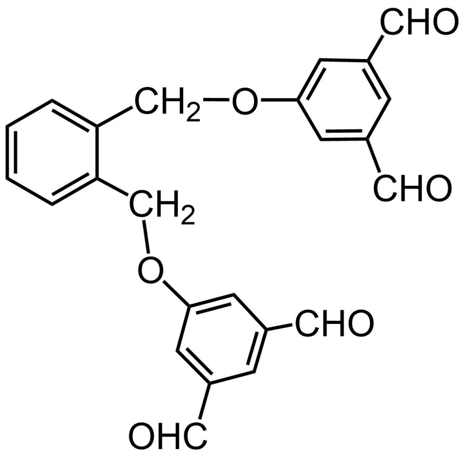

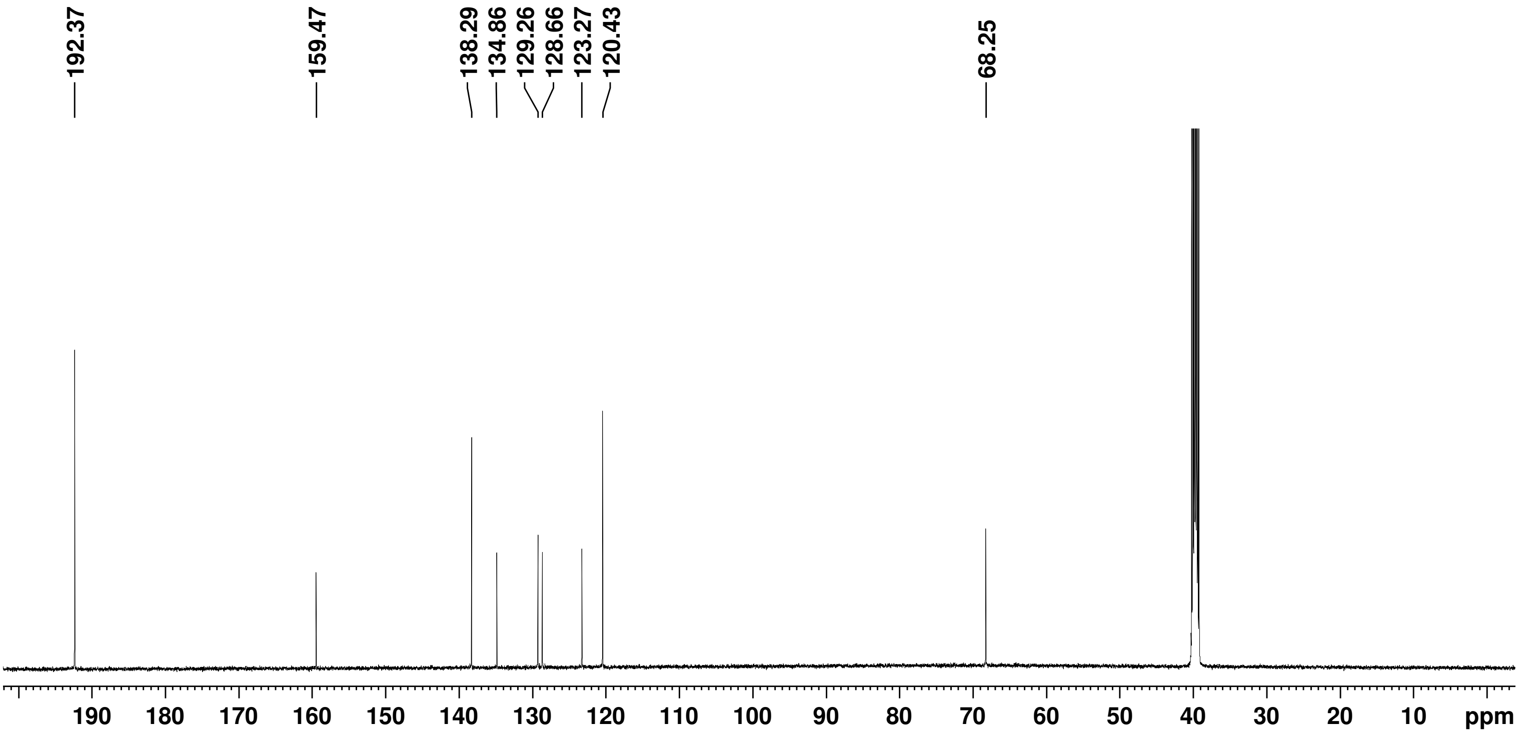

Figure S108. 125 MHz carbon-13 NMR spectrum of **18a** in DMSO-d_6.

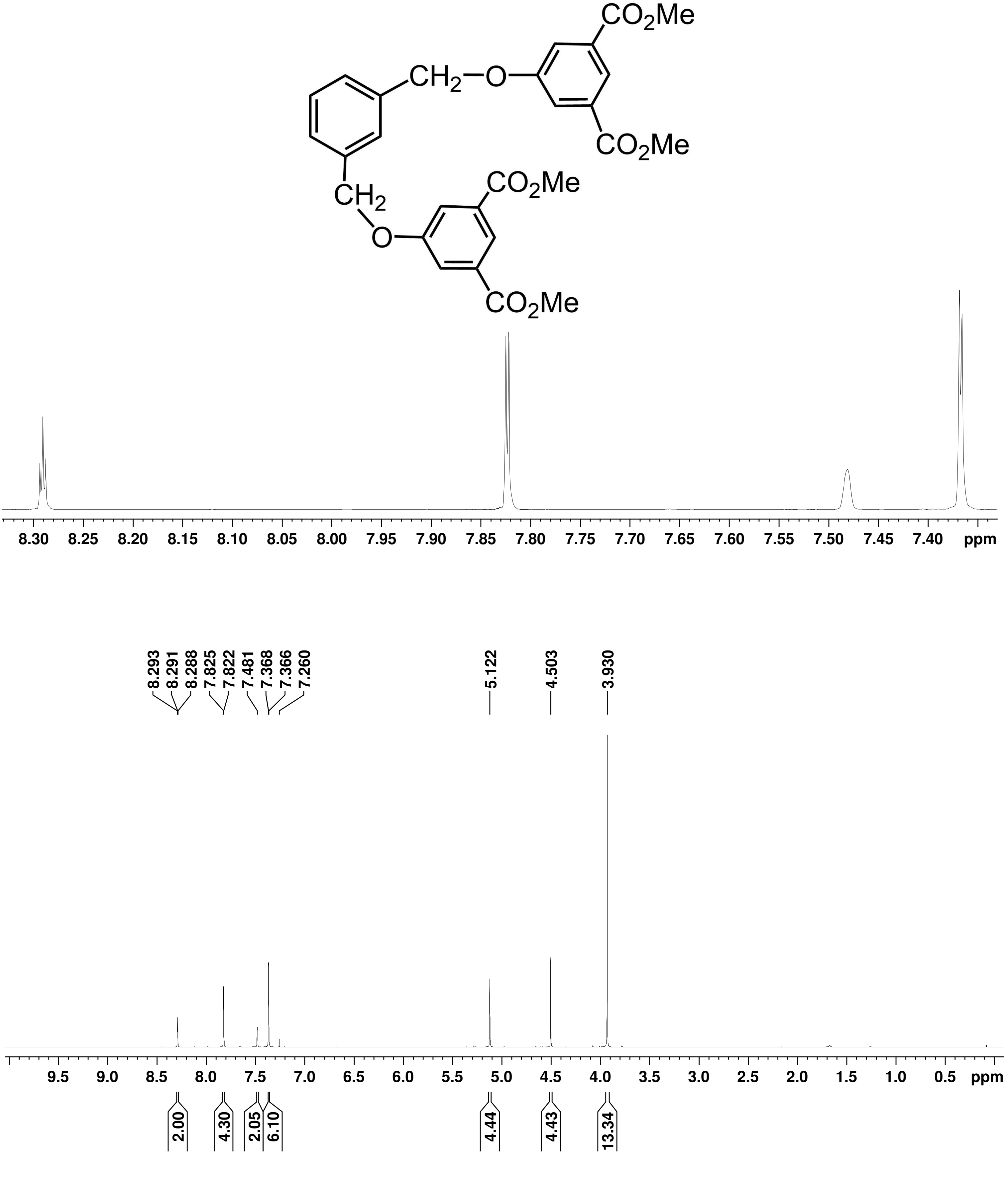

Figure S109. 500 MHz proton NMR spectrum of tetraester **16b** in $CDCl_3$.

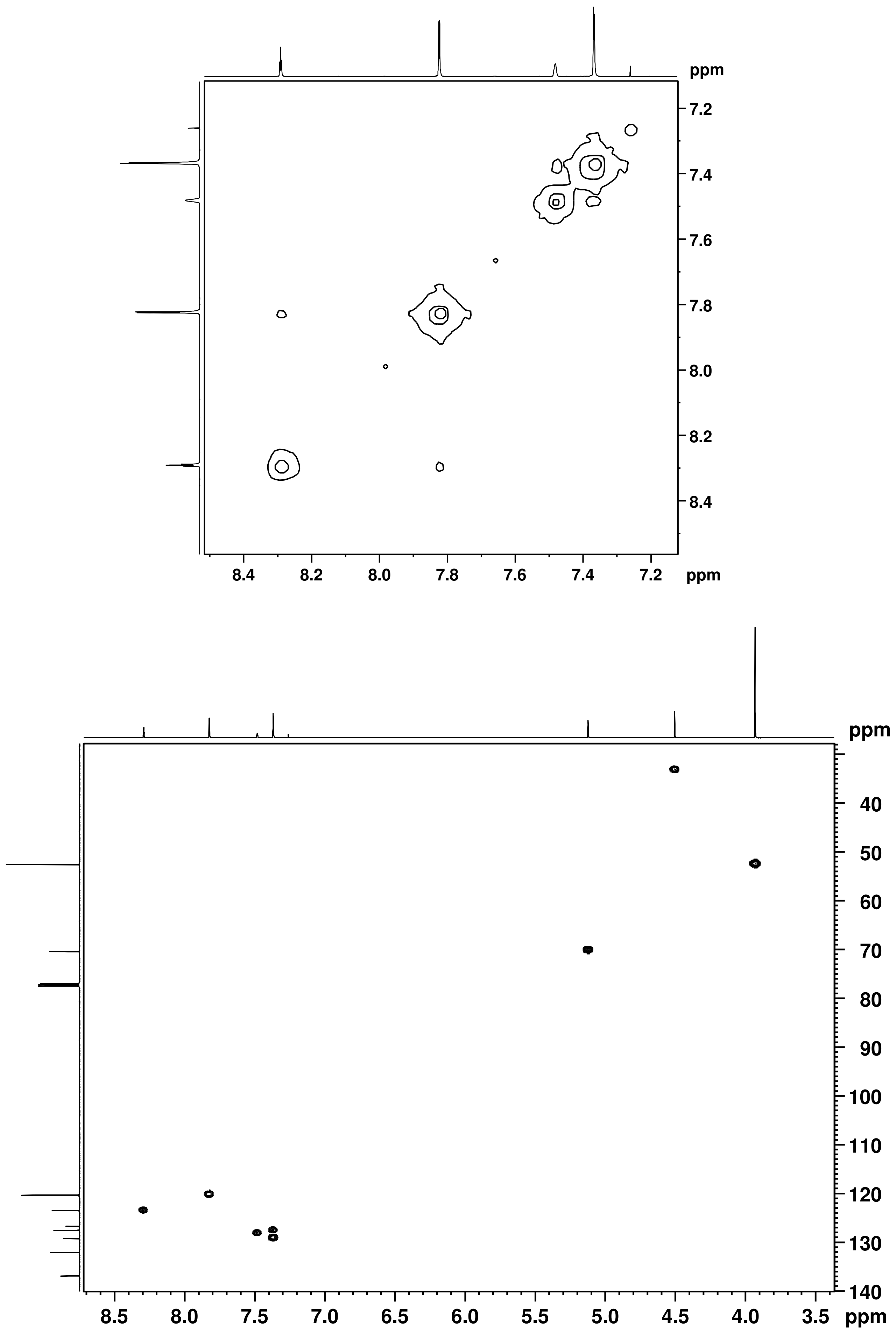

Figure S110. ^{1}H-^{1}H COSY (top) and HSQC (bottom) NMR spectra of **16b** in $CDCl_3$.

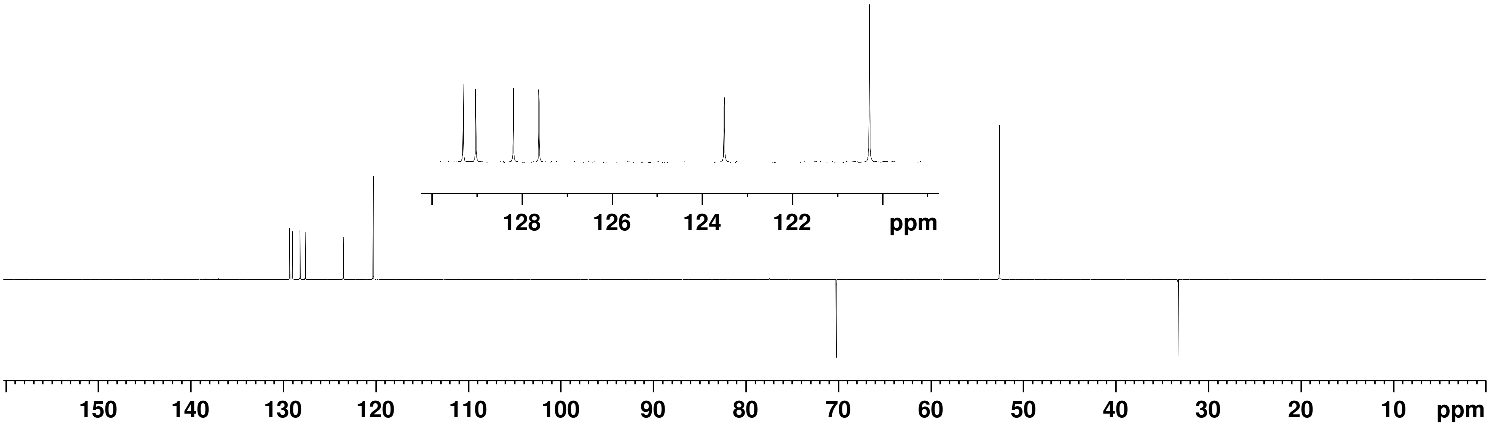

Figure S111. DEPT-135 NMR spectrum of **16b** in $CDCl_3$.

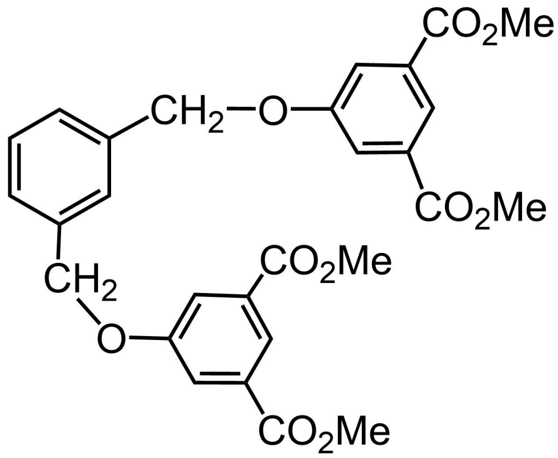

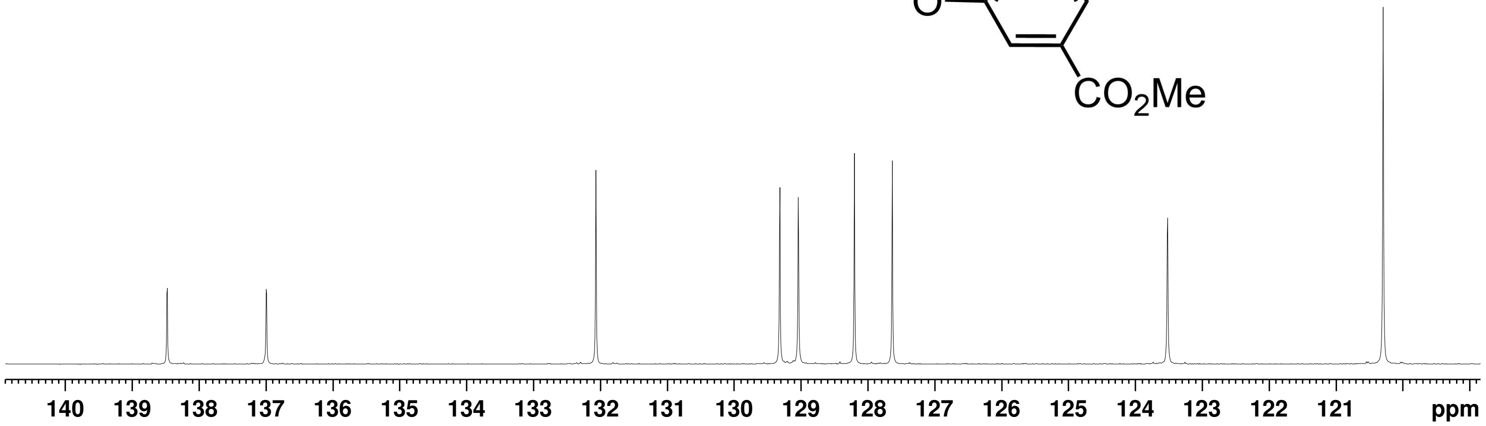

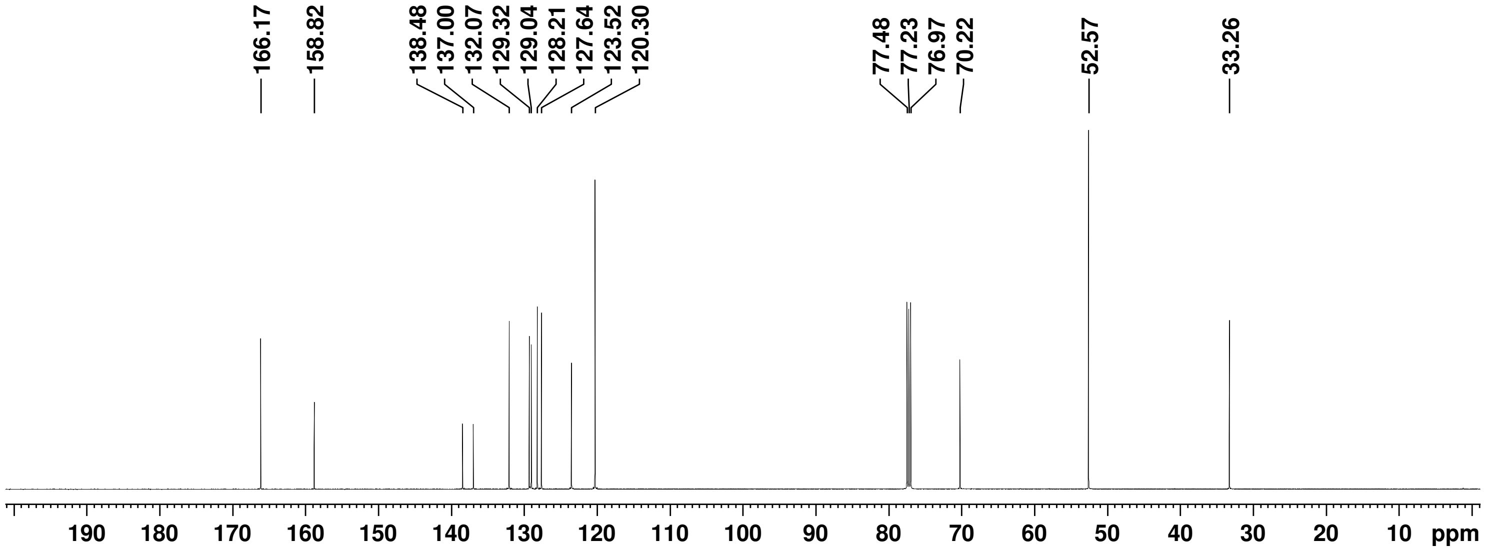

Figure S112. 125 MHz carbon-13 NMR spectrum of **16b** in $CDCl_3$.

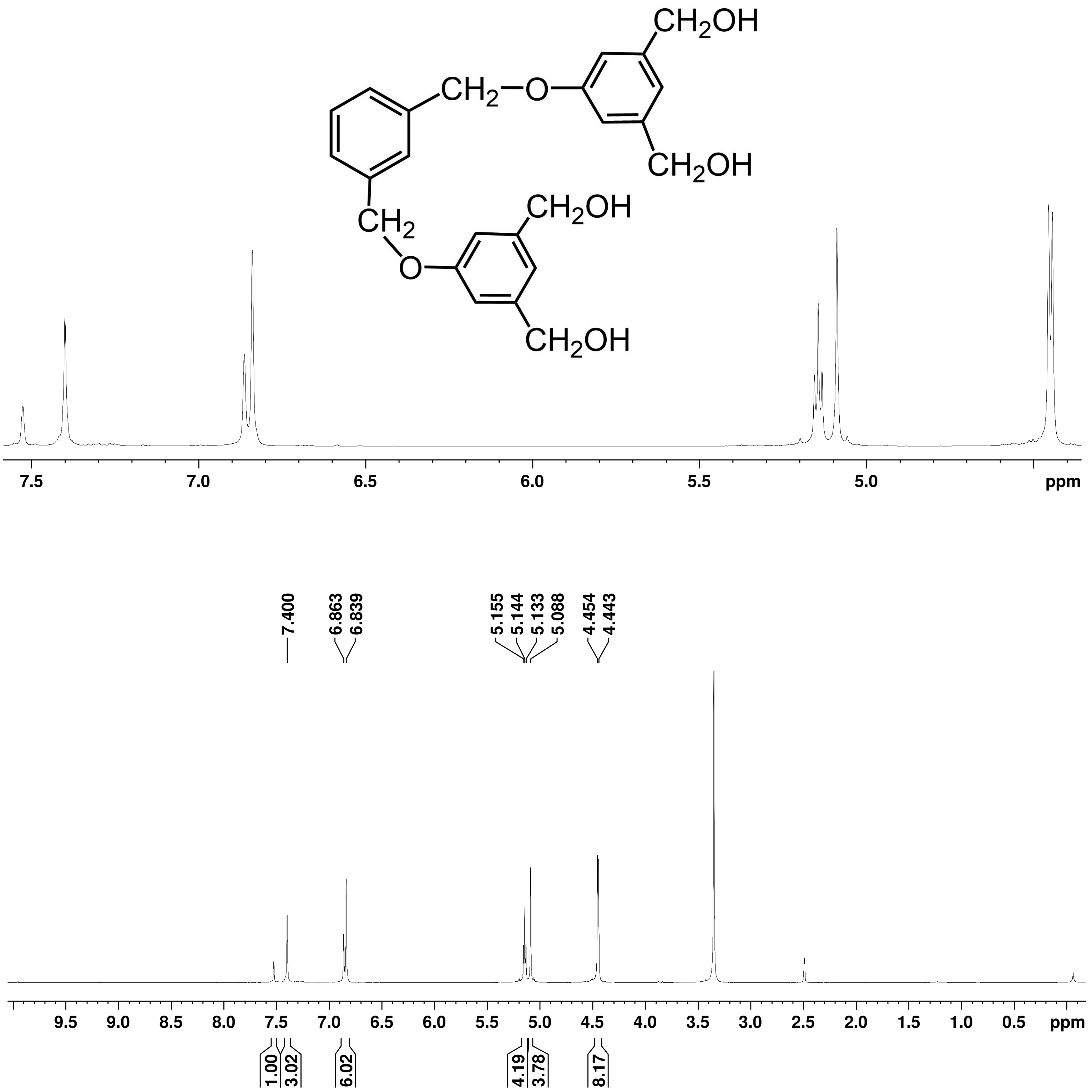

Figure S113. 500 MHz proton NMR spectrum of tetraalcohol **17b** in DMSO-d_6.

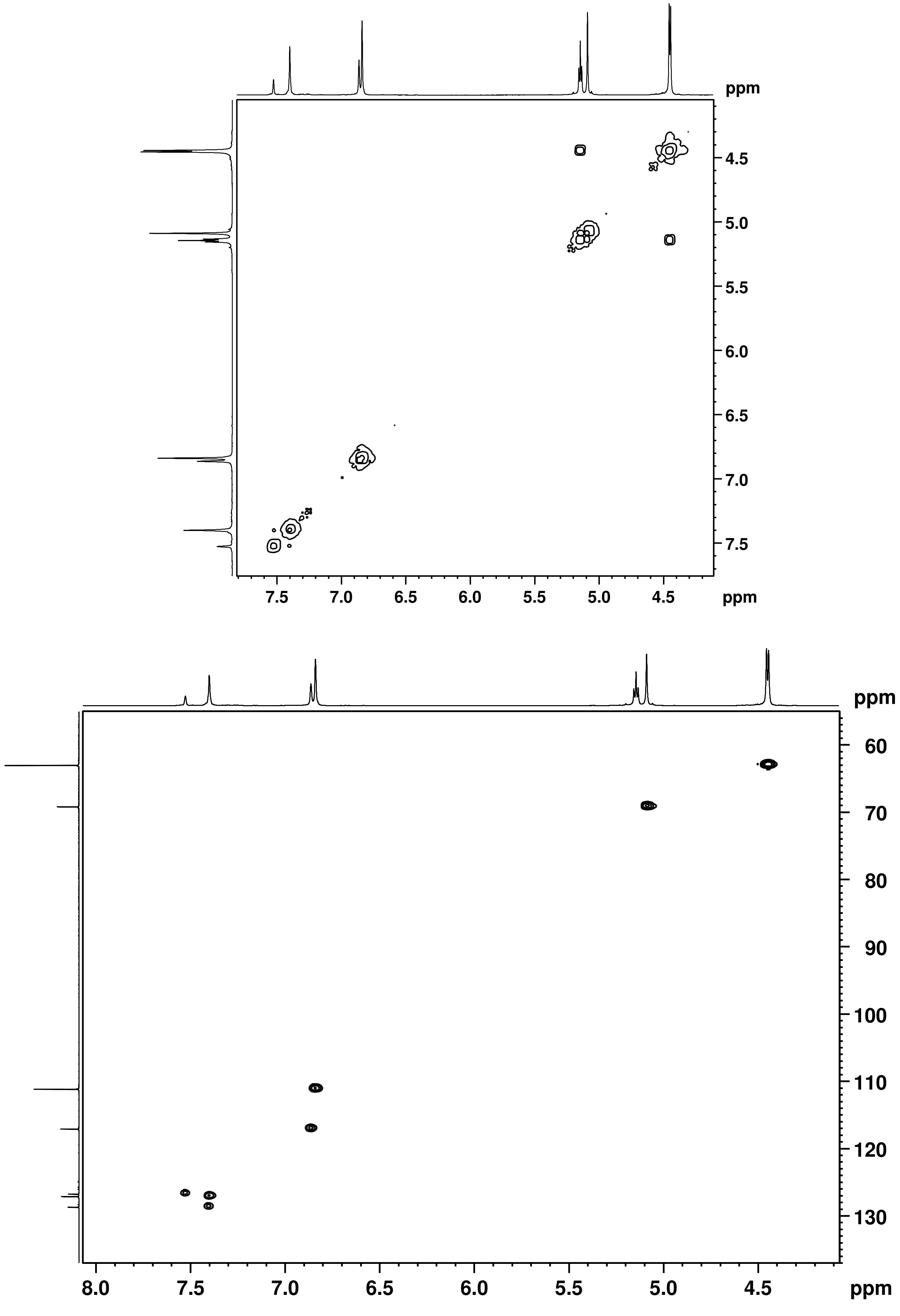

Figure S114. ^{1}H-^{1}H COSY (top) and HSQC (bottom) NMR spectra of **17b** in DMSO-d_6.

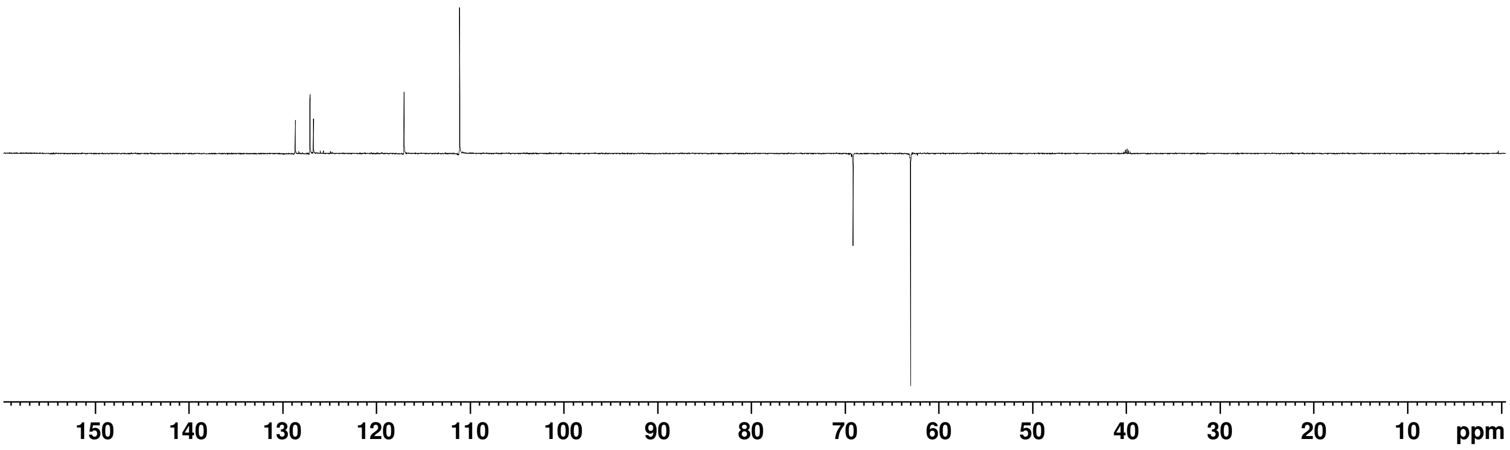

Figure S115. DEPT-135 NMR spectrum of **17b** in DMSO-d_6.

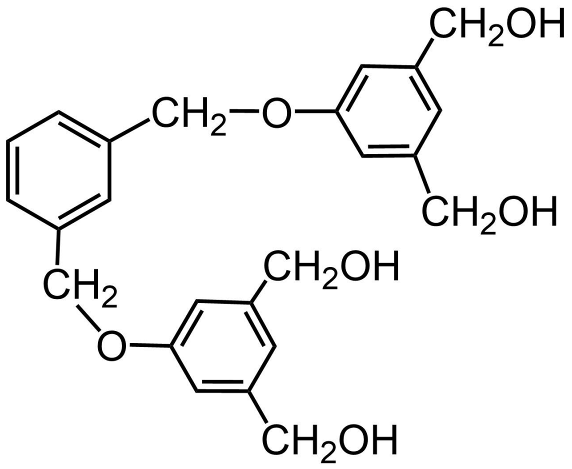

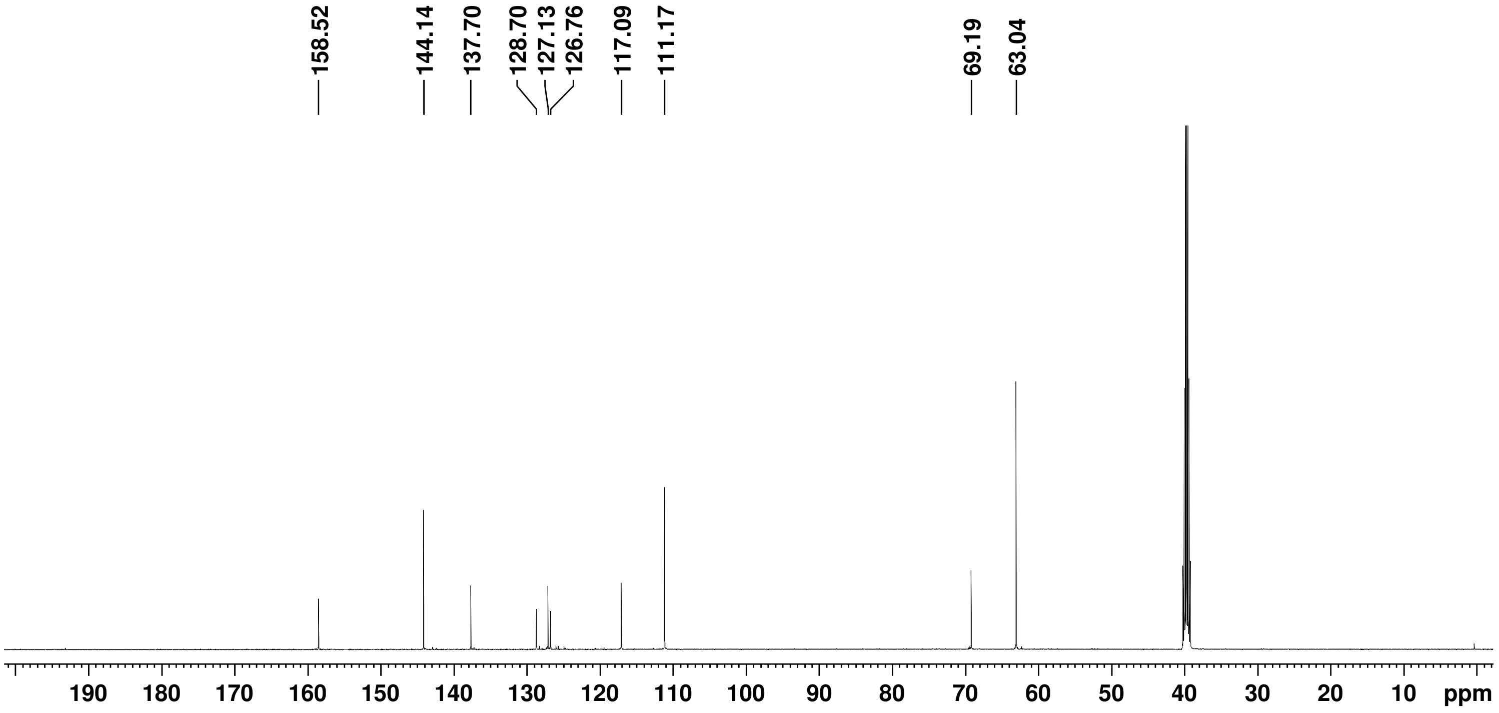

Figure S116. Carbon-13 NMR spectrum of **17b** in DMSO-d_6.

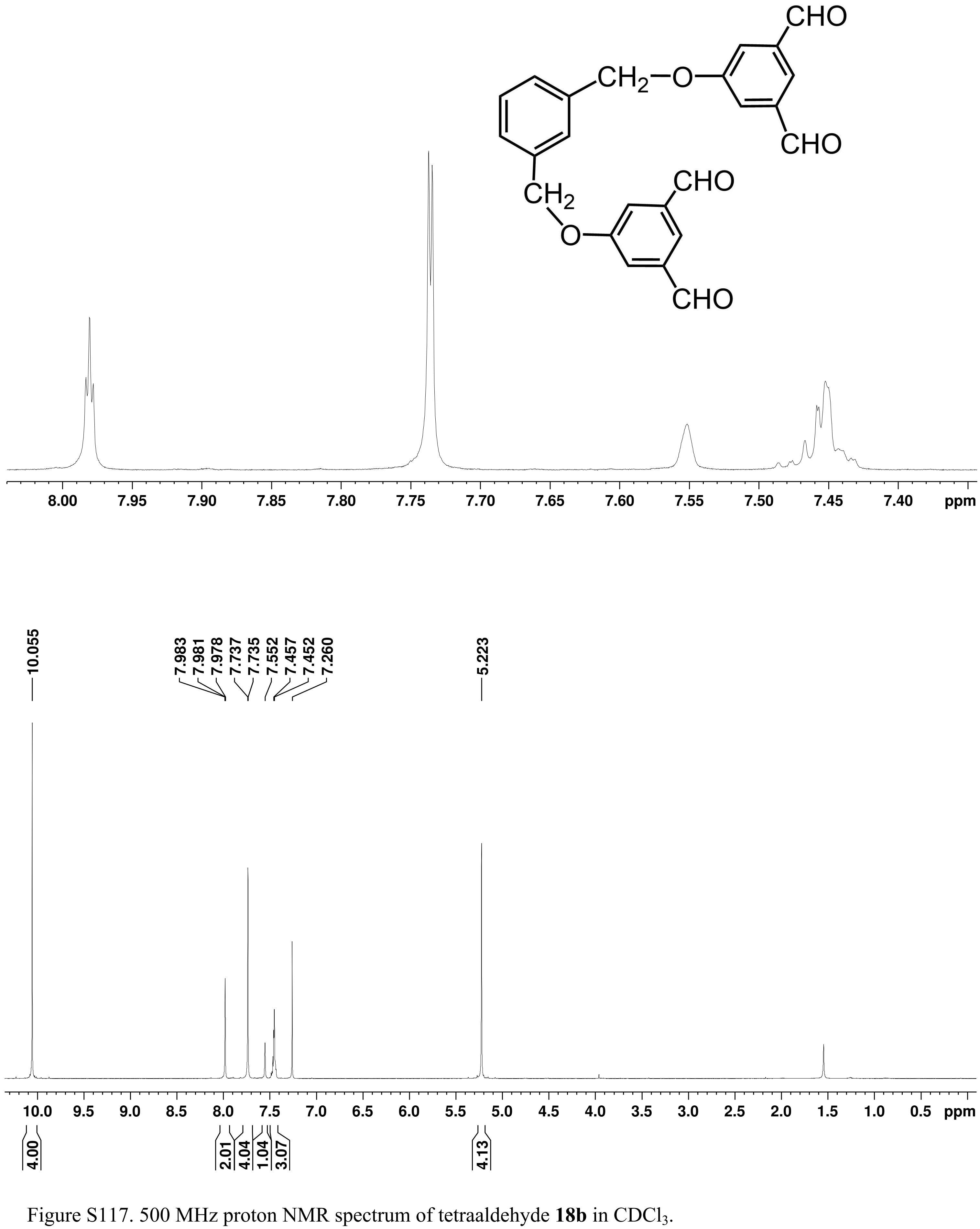

Figure S117. 500 MHz proton NMR spectrum of tetraaldehyde **18b** in $CDCl_3$.

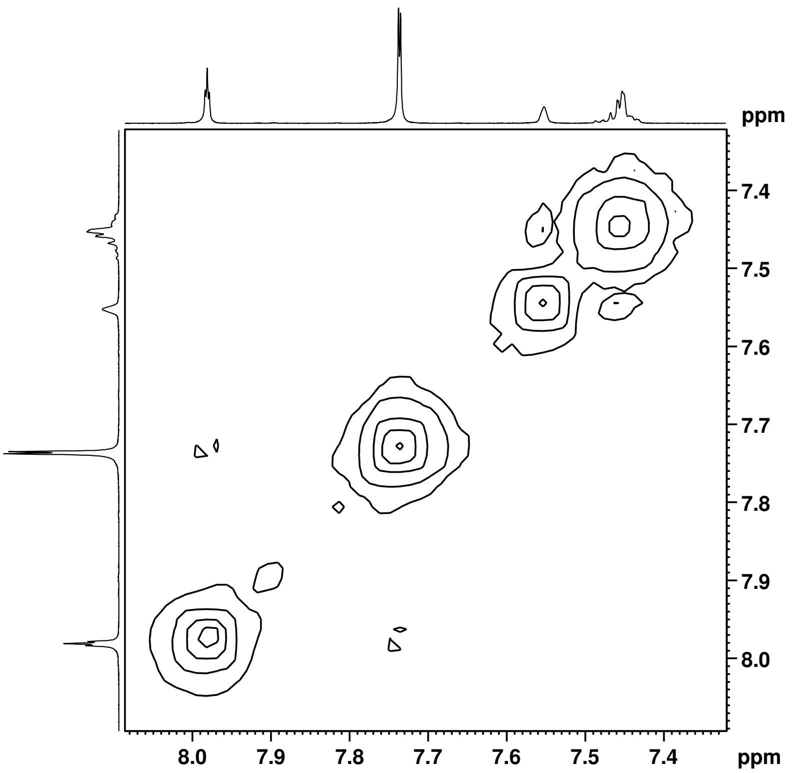

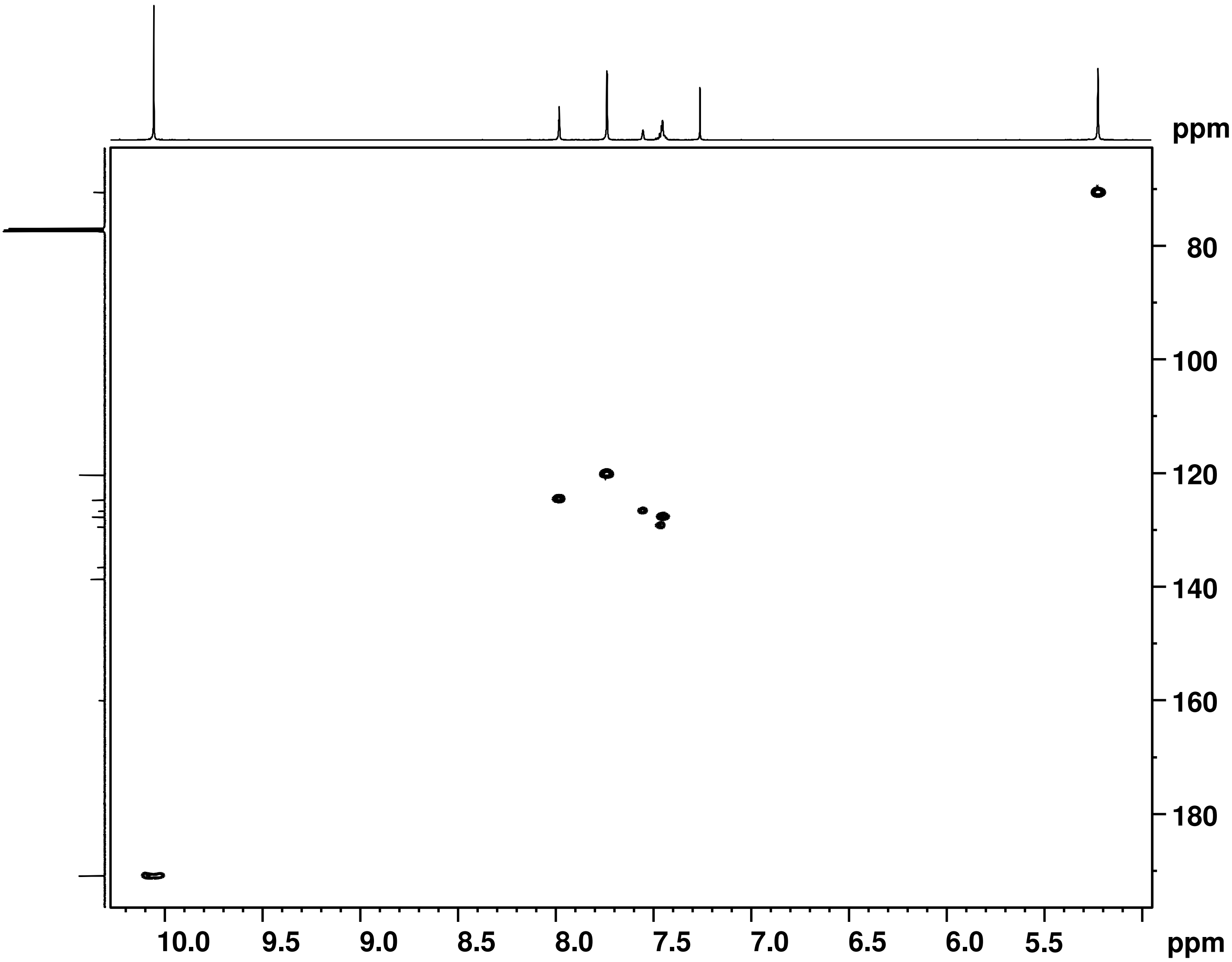

Figure S118. 1H-1H COSY (top) and HSQC (bottom) NMR spectra of **18b** in $CDCl_3$.

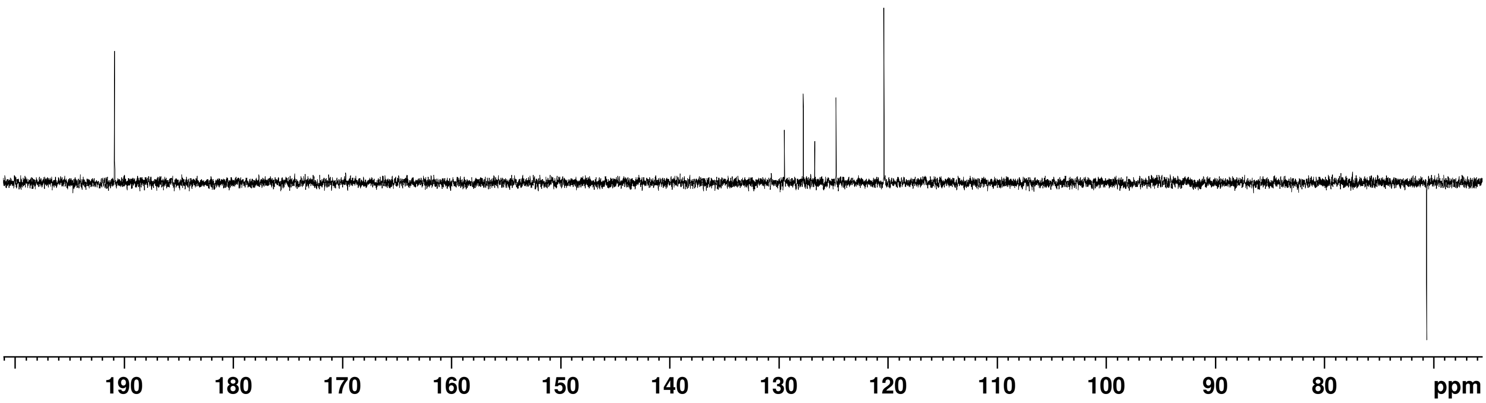

Figure S119. DEPT-135 NMR spectrum of **18b** in $CDCl_3$.

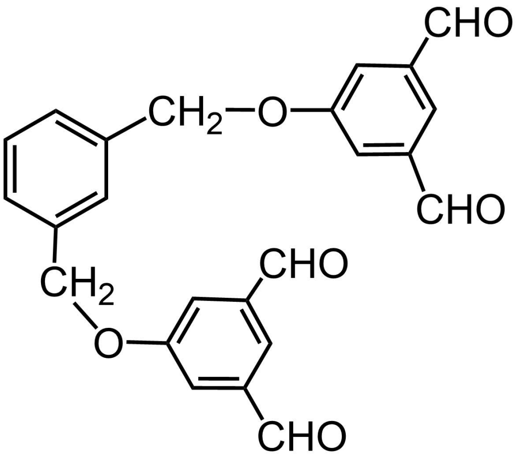

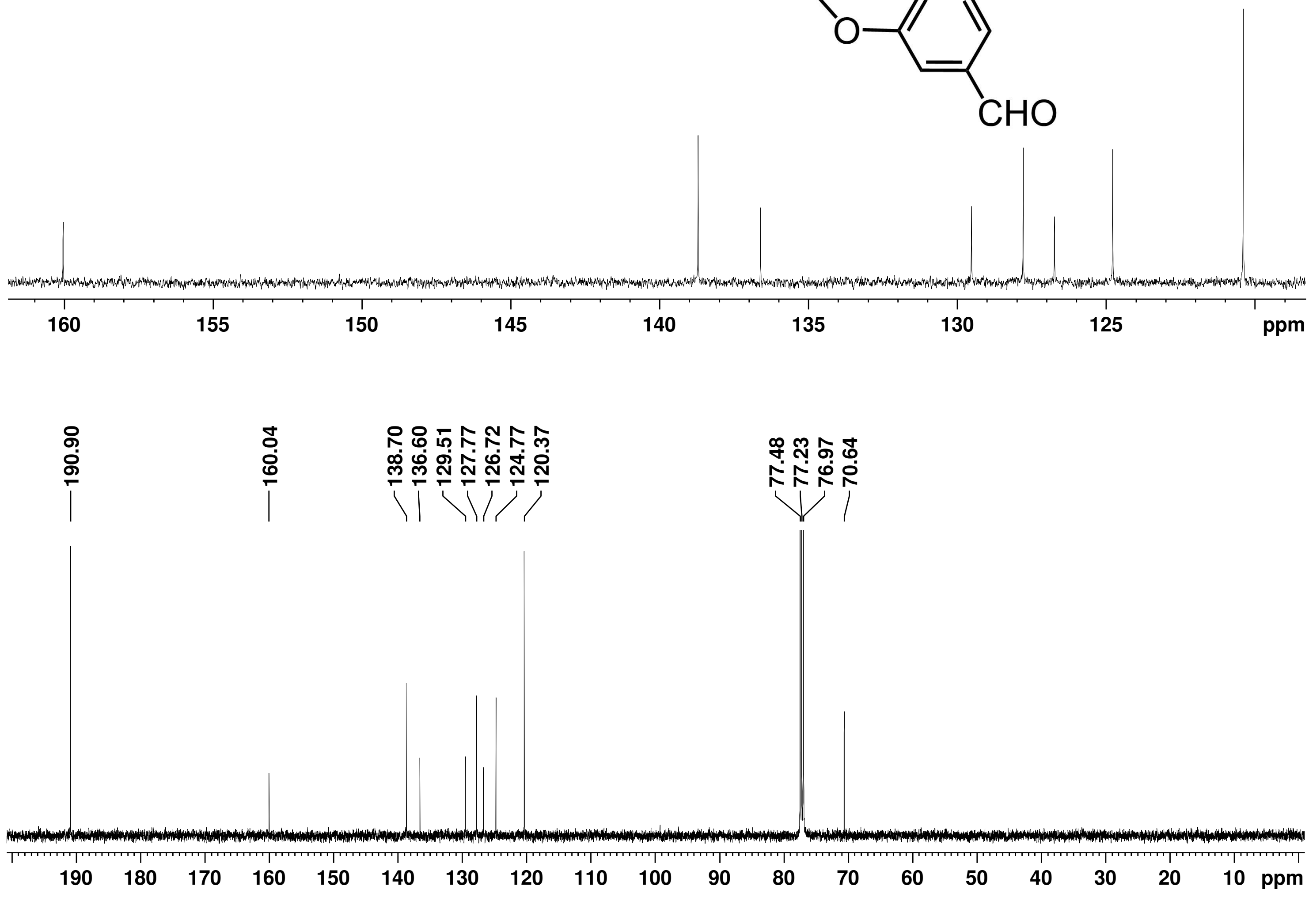

Figure S120. 125 MHz carbon-13 NMR spectrum of **18b** in $CDCl_3$.

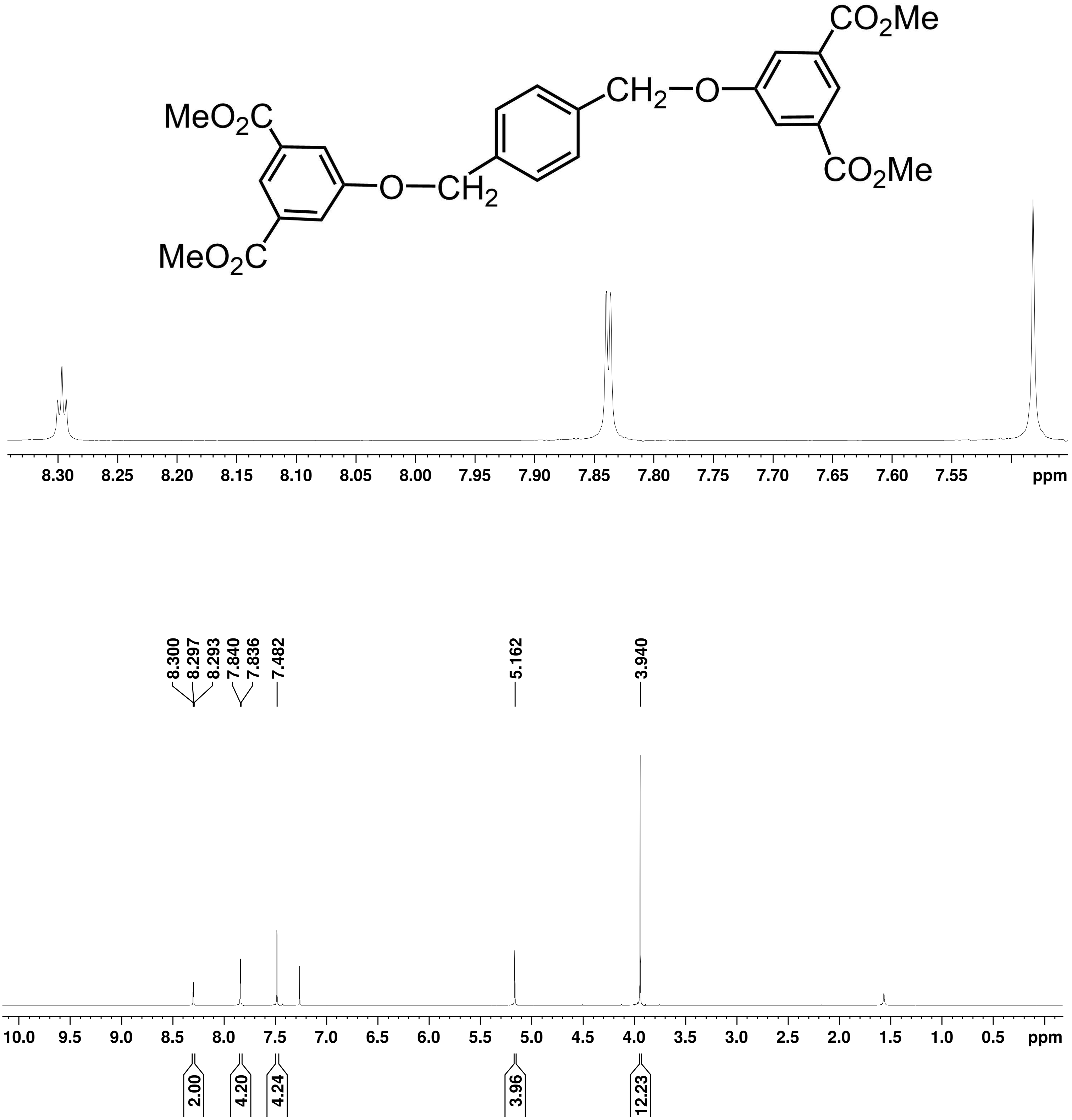

Figure S121. 500 MHz proton NMR spectrum of tetraester **16c** in $CDCl_3$.

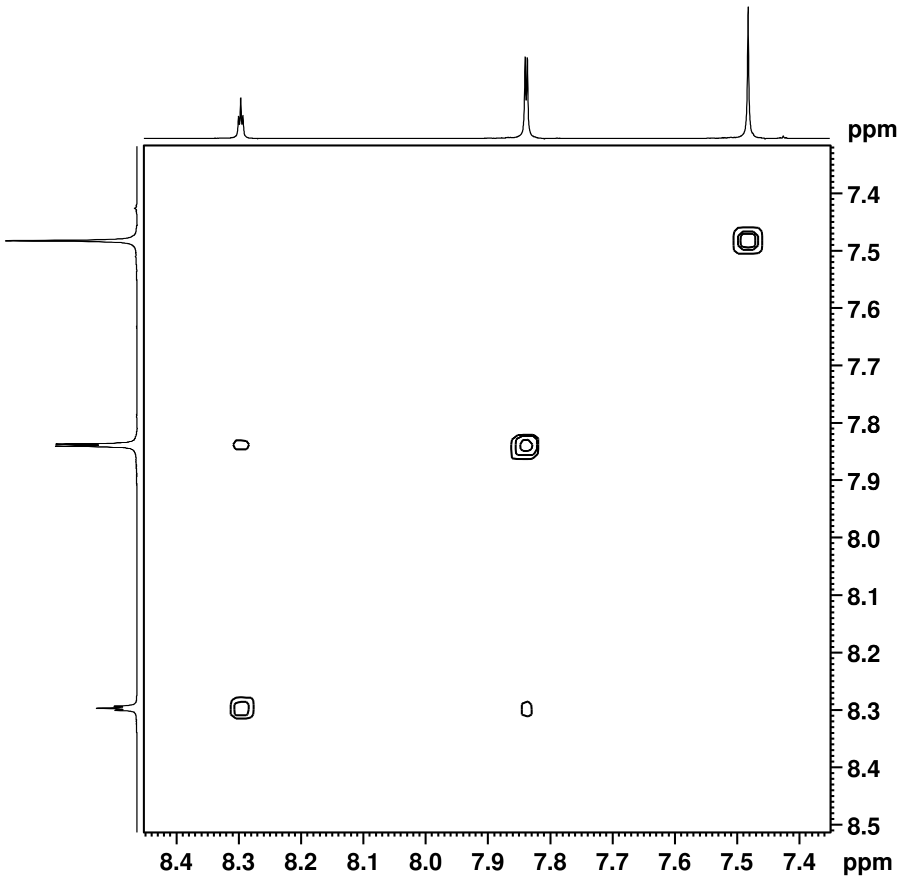

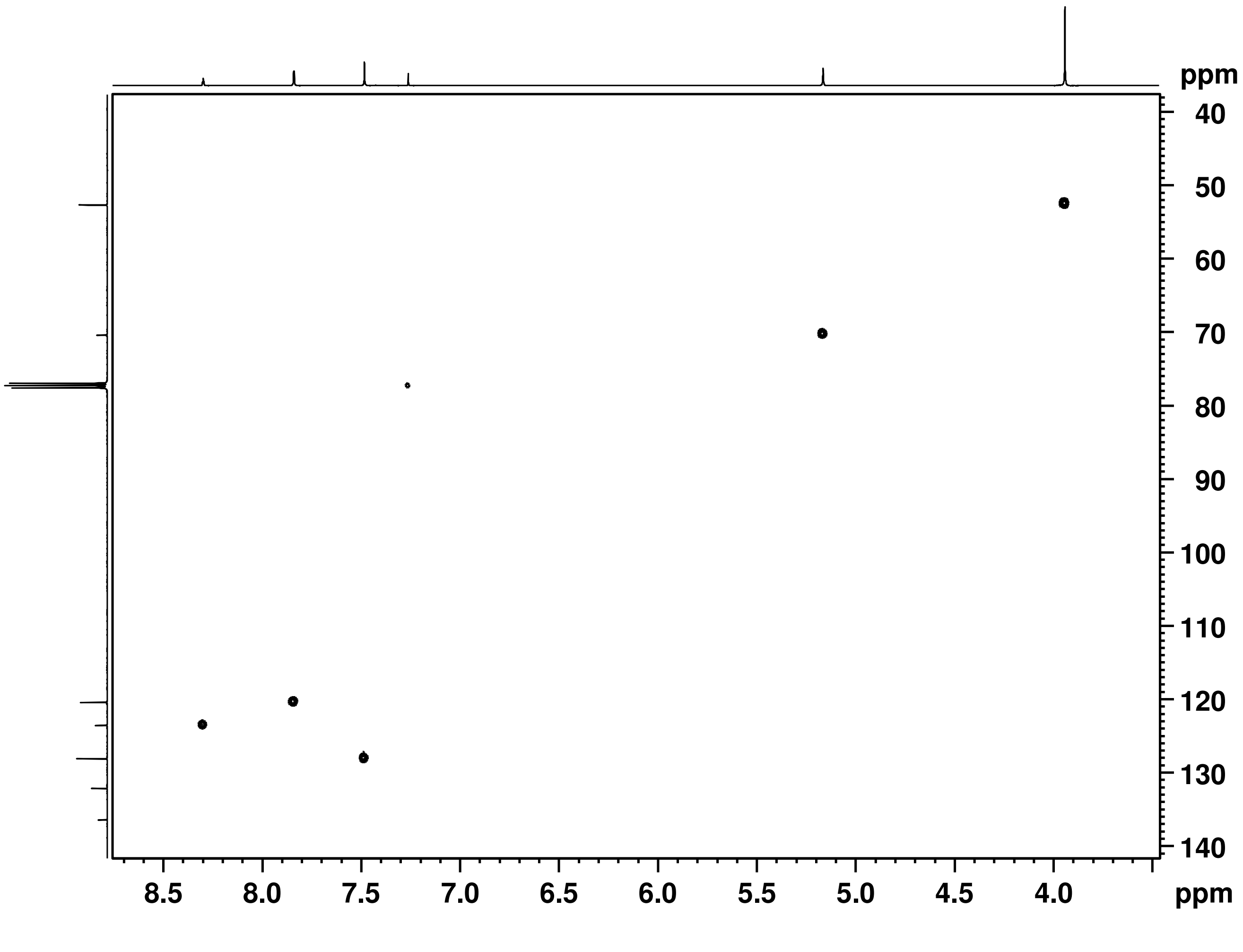

Figure S122. ^{1}H-^{1}H COSY (top) and HSQC (bottom) NMR spectra of **16c** in $CDCl_3$.

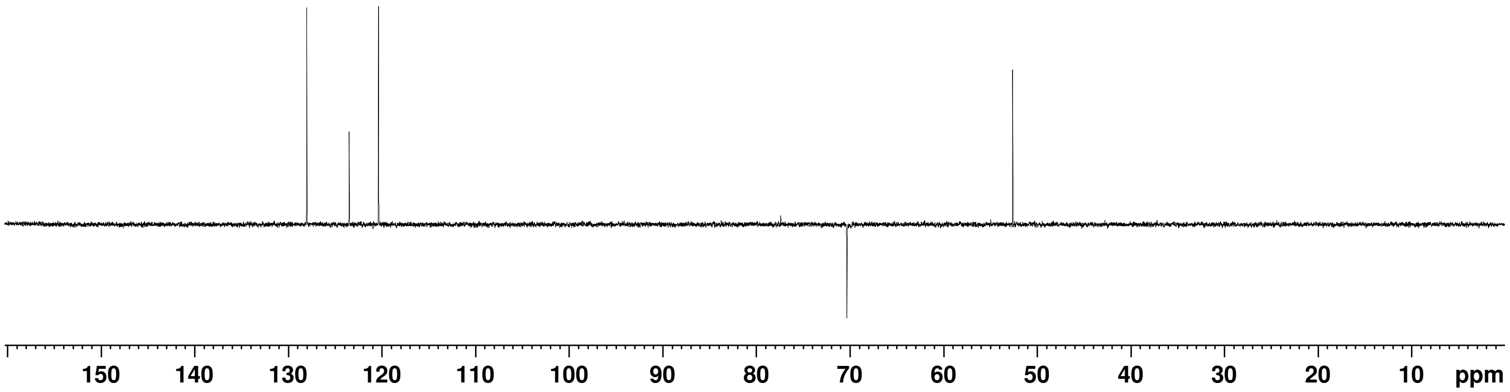

Figure S123. DEPT-135 NMR spectrum of **16c** in $CDCl_3$.

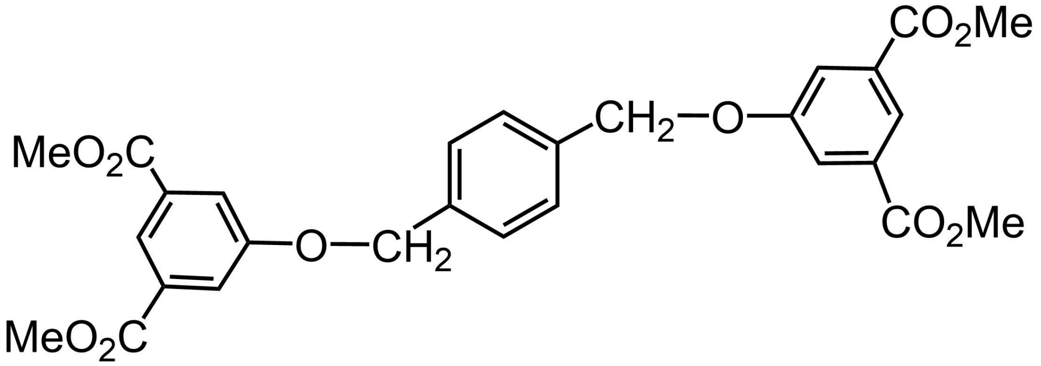

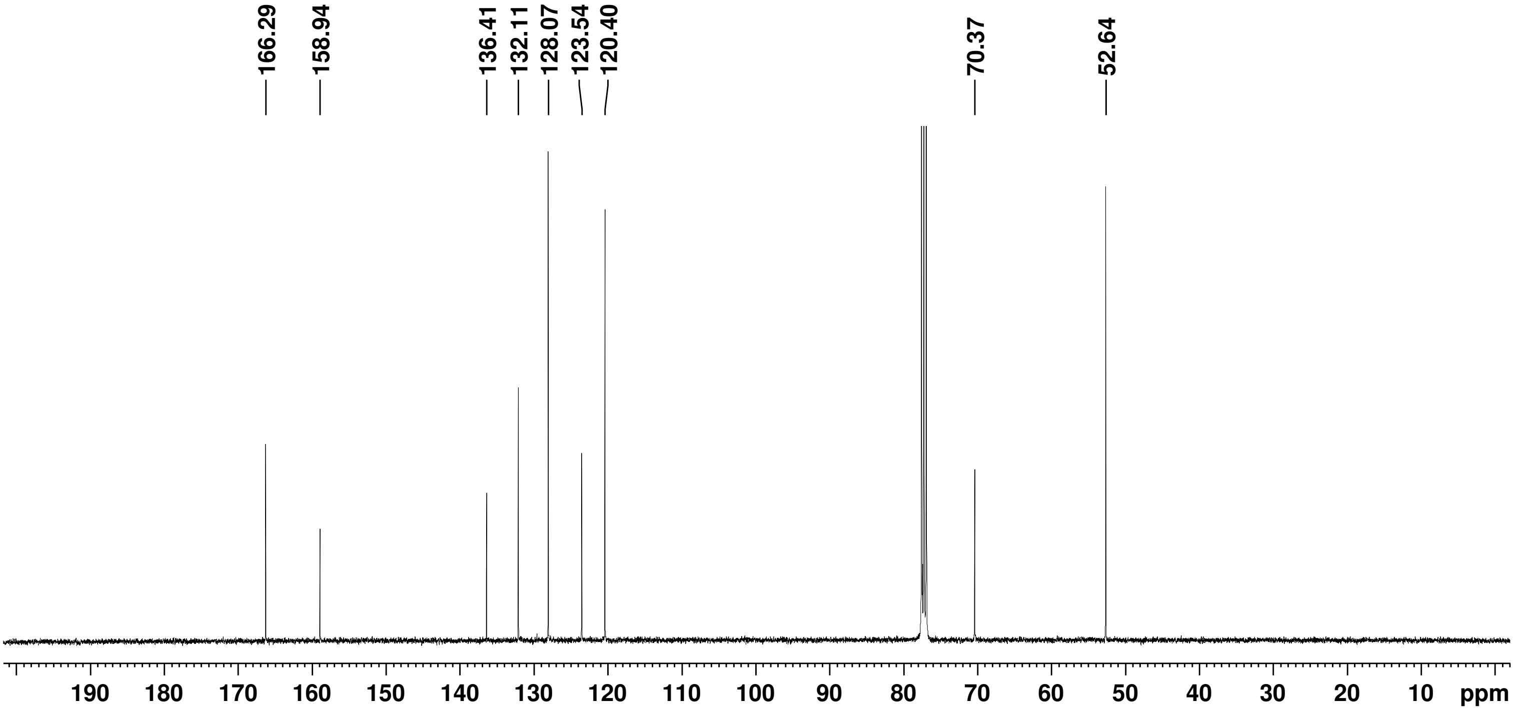

Figure S124. 125 MHz carbon-13 NMR spectrum of dimer **16c** in $CDCl_3$.

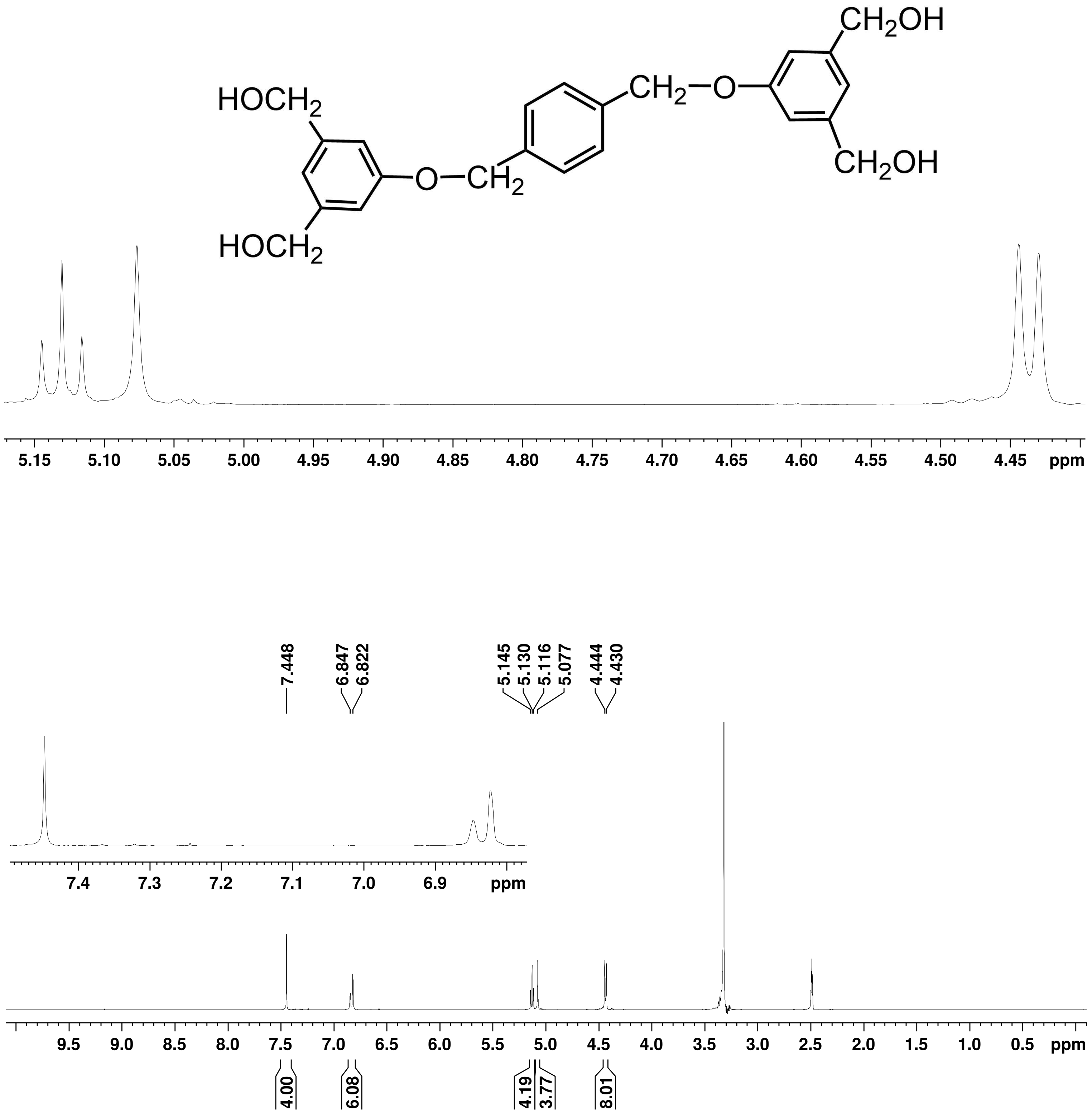

Figure S125. 400 MHz proton NMR spectrum of tetra-alcohol **17c** in DMSO-d_6.

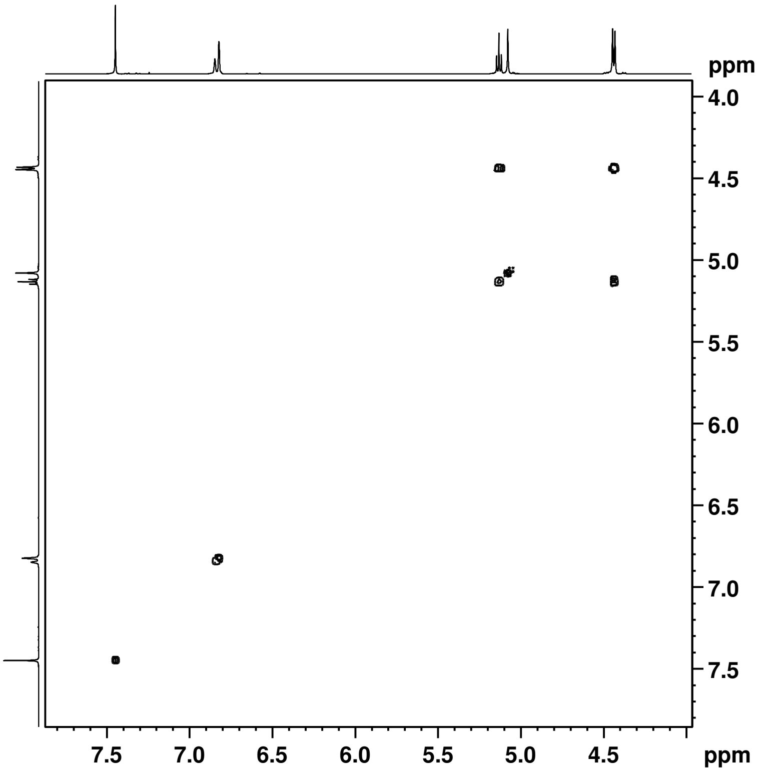

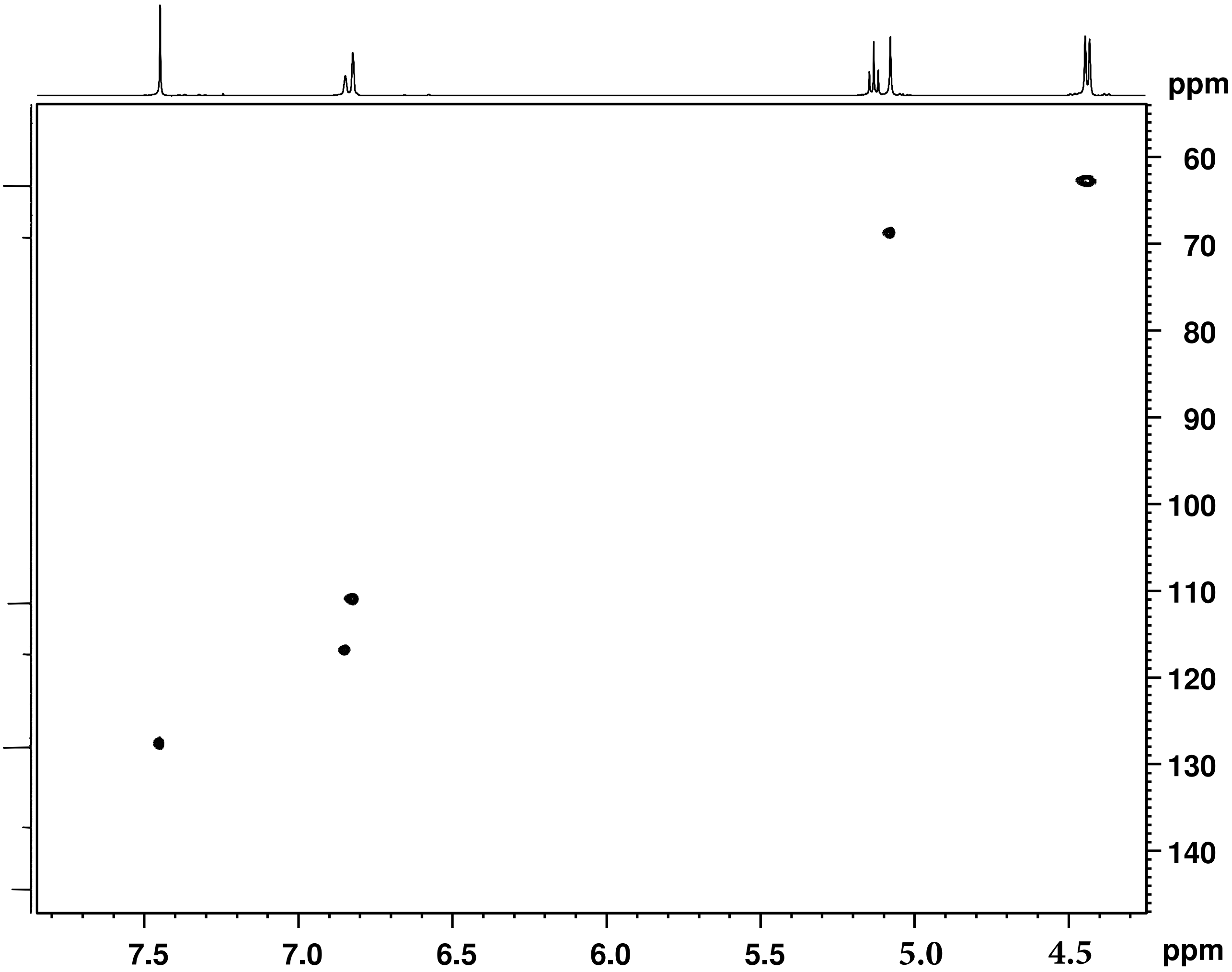

Figure S126. ^{1}H-^{1}H COSY (top) and HSQC (bottom) NMR spectra of tetra-alcohol **17c** in DMSO-d_6.

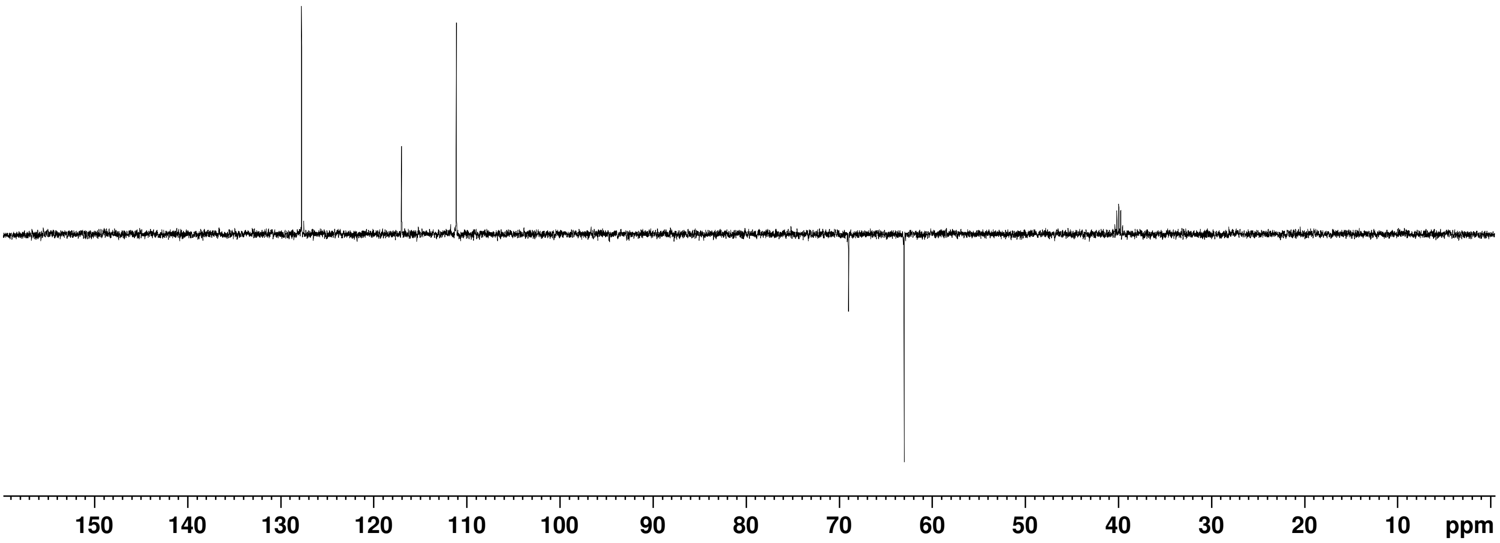

Figure S127. DEPT-135 NMR spectrum of tetra-alcohol **17c** in in DMSO-d_6.

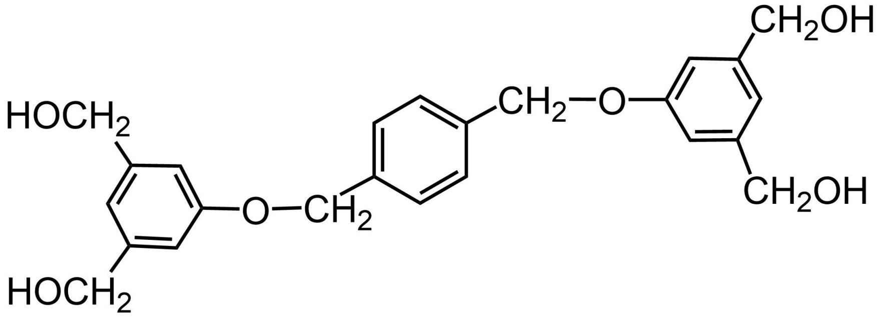

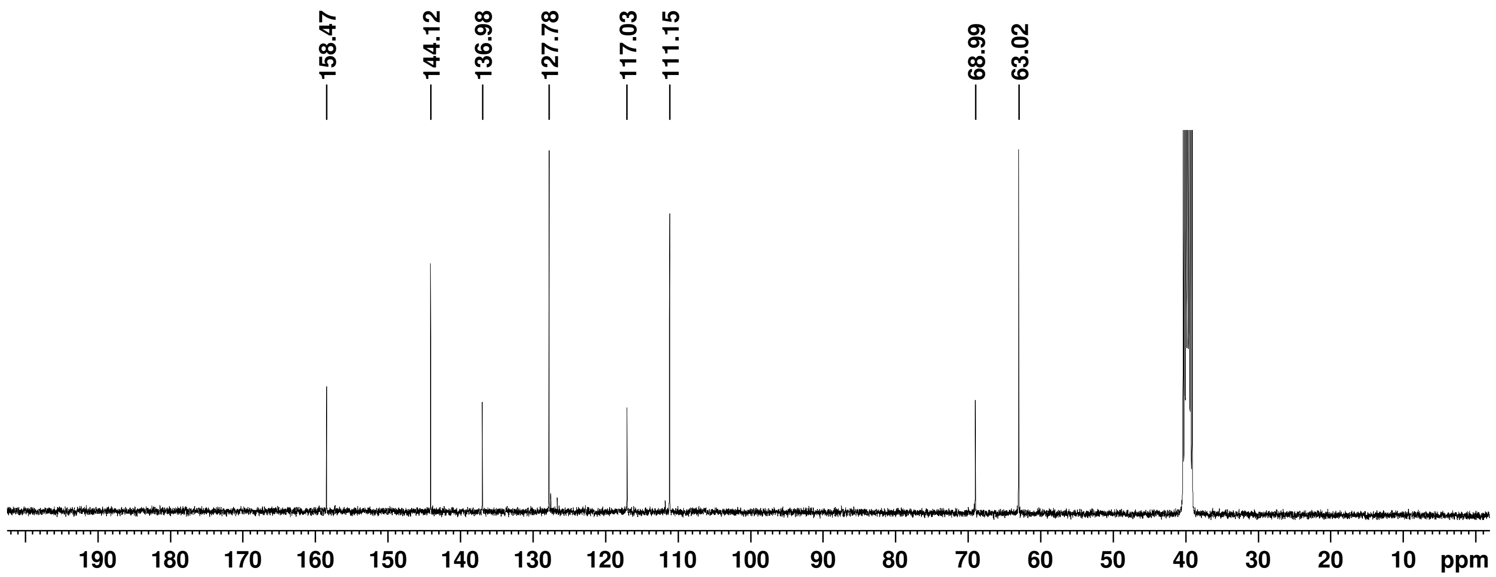

Figure S128. 100 MHz carbon-13 NMR spectrum of tetra-alcohol **17c** in DMSO-d_6.

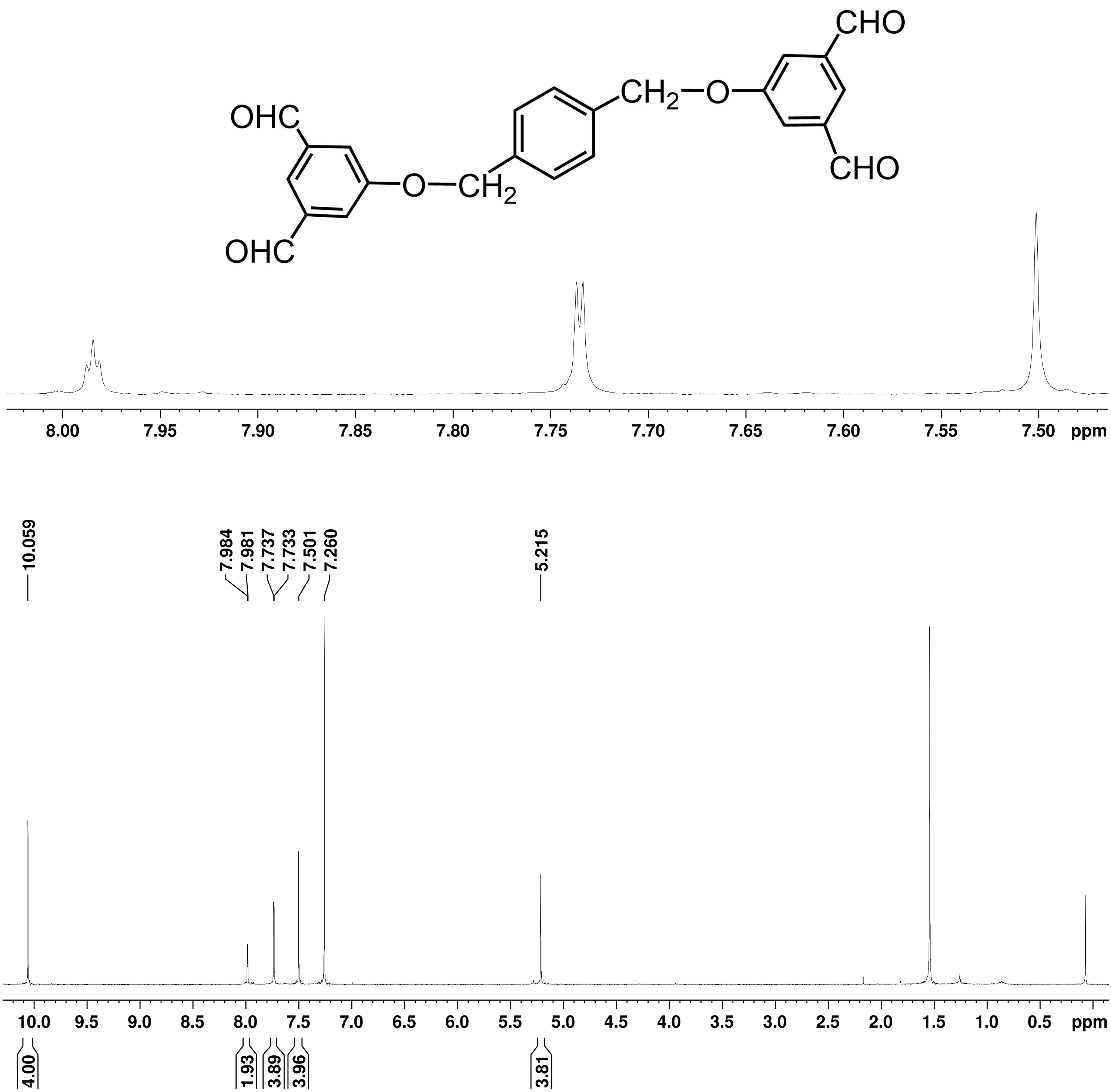

Figure S129. Proton NMR spectrum of *p*-phenylene-linked tetraaldehyde in $CDCl_3$.

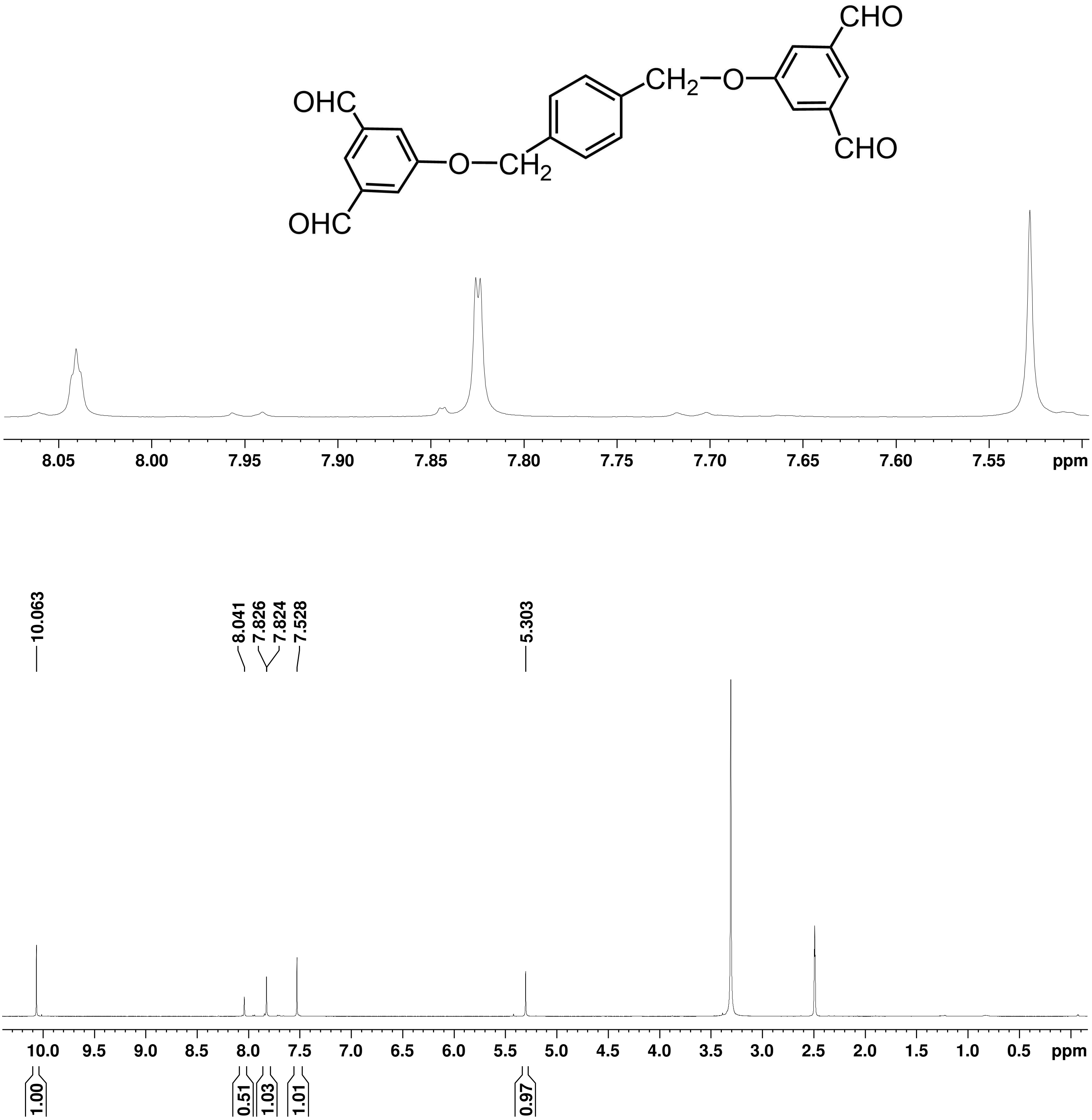

Figure S130. Proton NMR spectrum of *p*-phenylene-linked tetraaldehyde **18c** in DMSO-d_6.

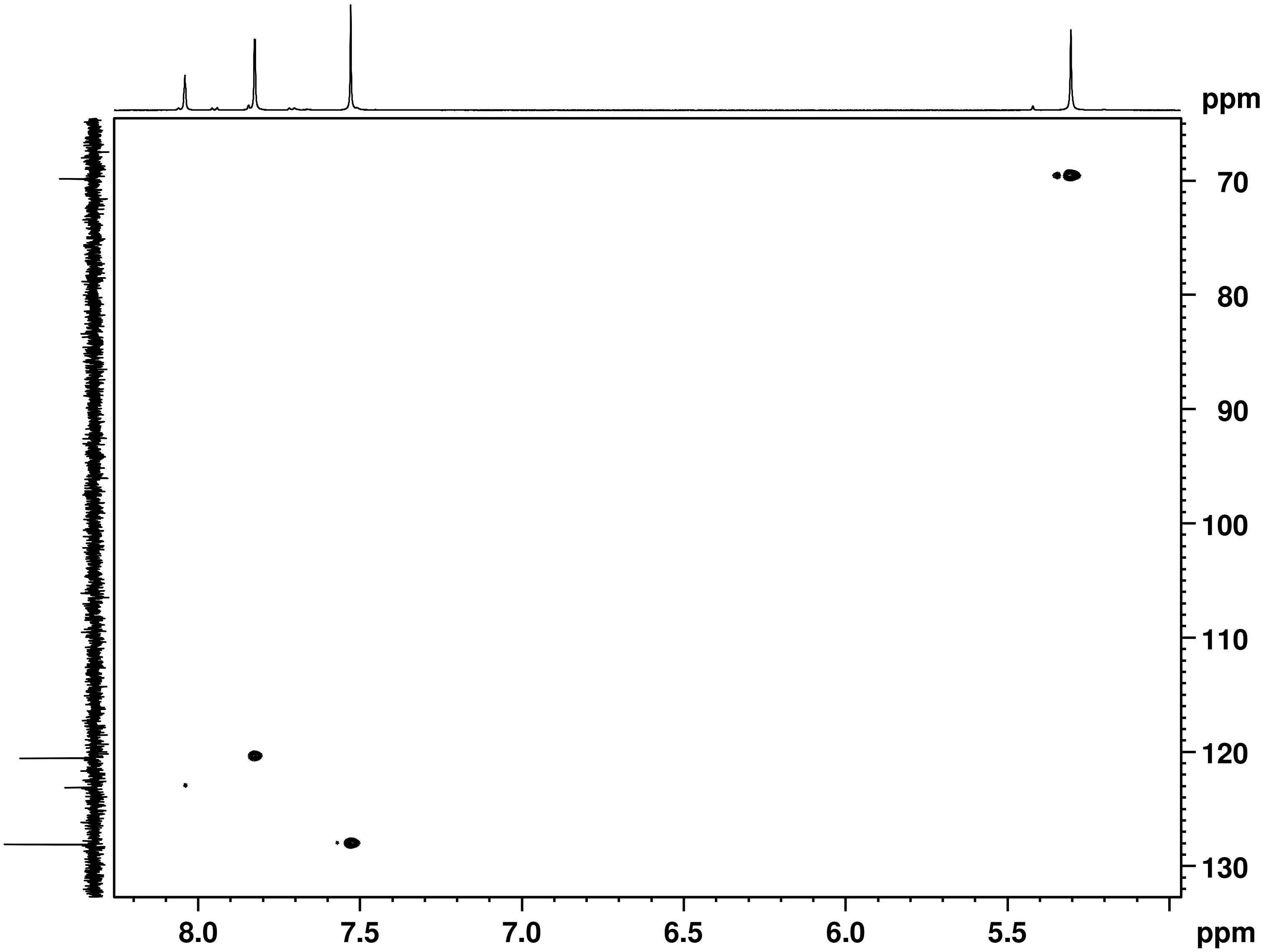

Figure S131. ^{1}H-^{1}H COSY NMR spectrum of *p*-phenylene-linked tetraaldehyde **18c** in DMSO-d_6.

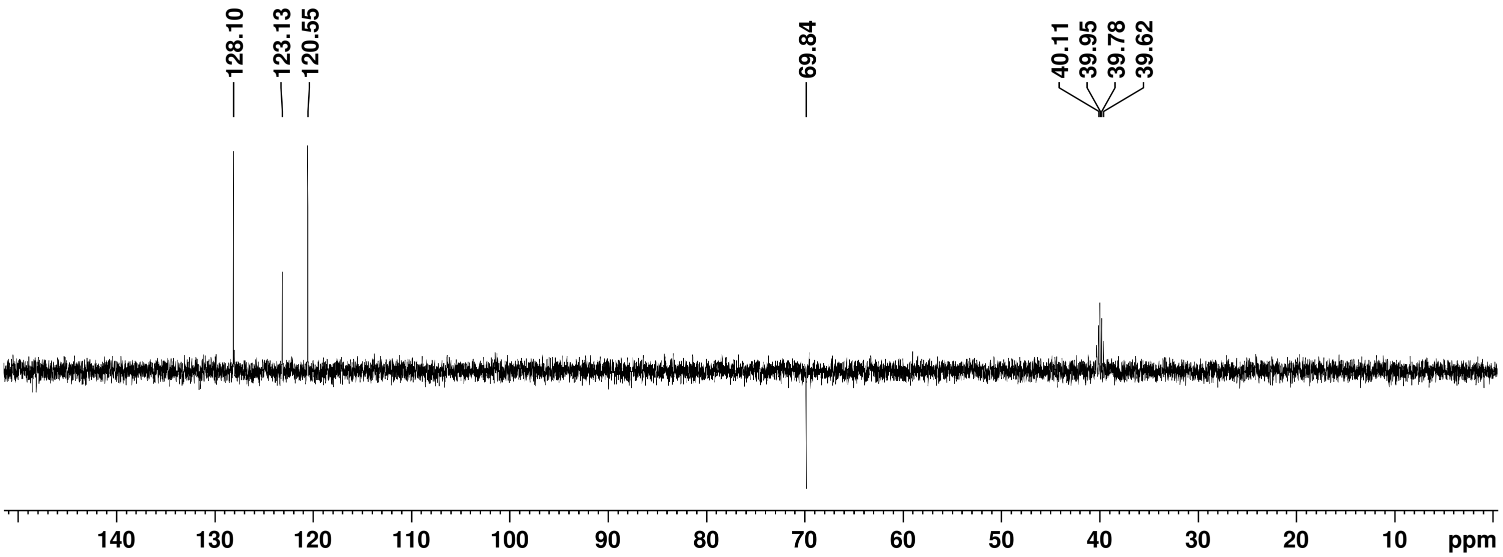

Figure S132. DEPT-135 NMR spectrum of *p*-phenylene-linked tetraaldehyde **18c** in DMSO-d_6.

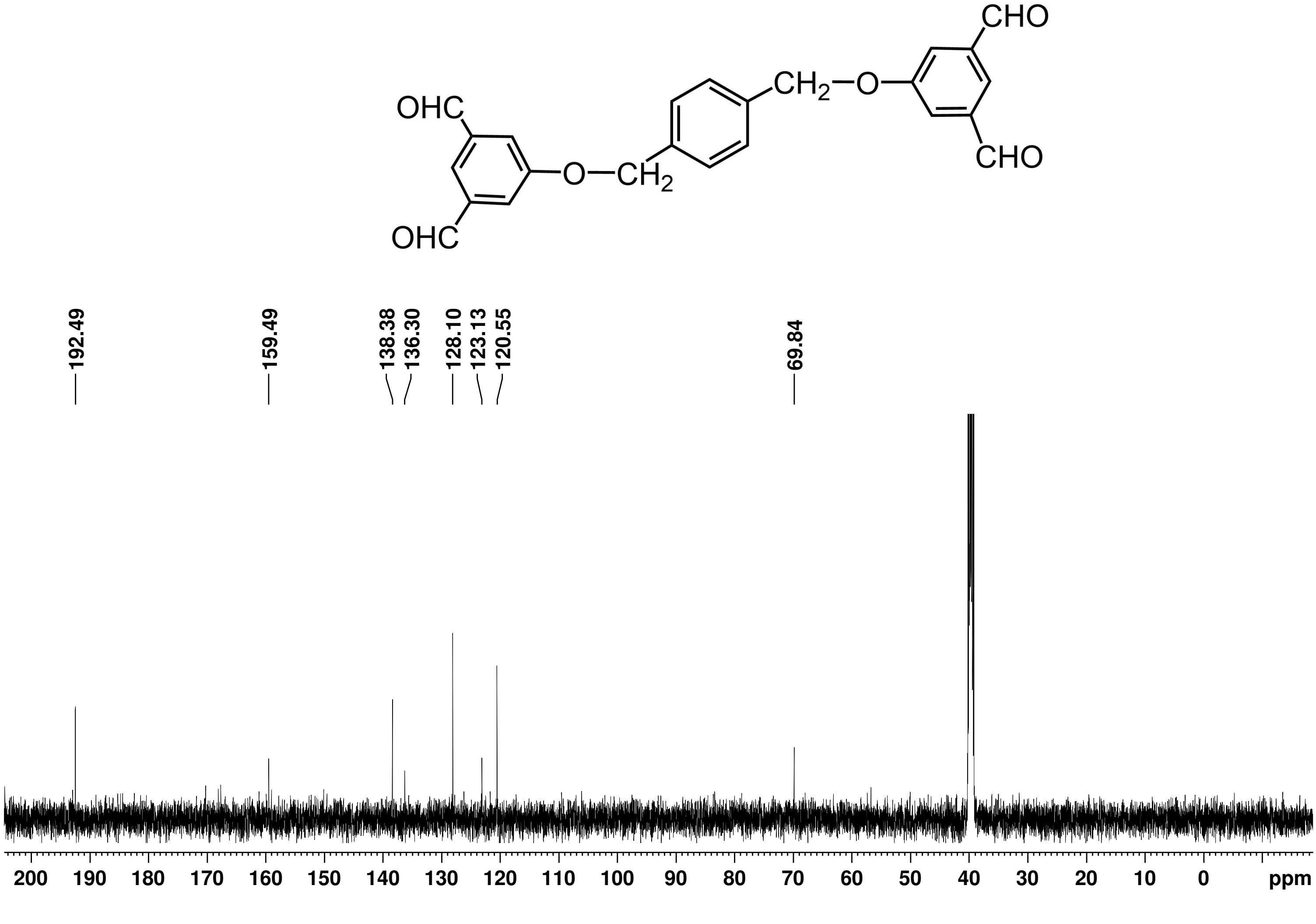

Figure S133. 100 MHz carbon-13 NMR spectrum of *p*-phenylene-linked tetraaldehyde **18c** in DMSO-d_6.

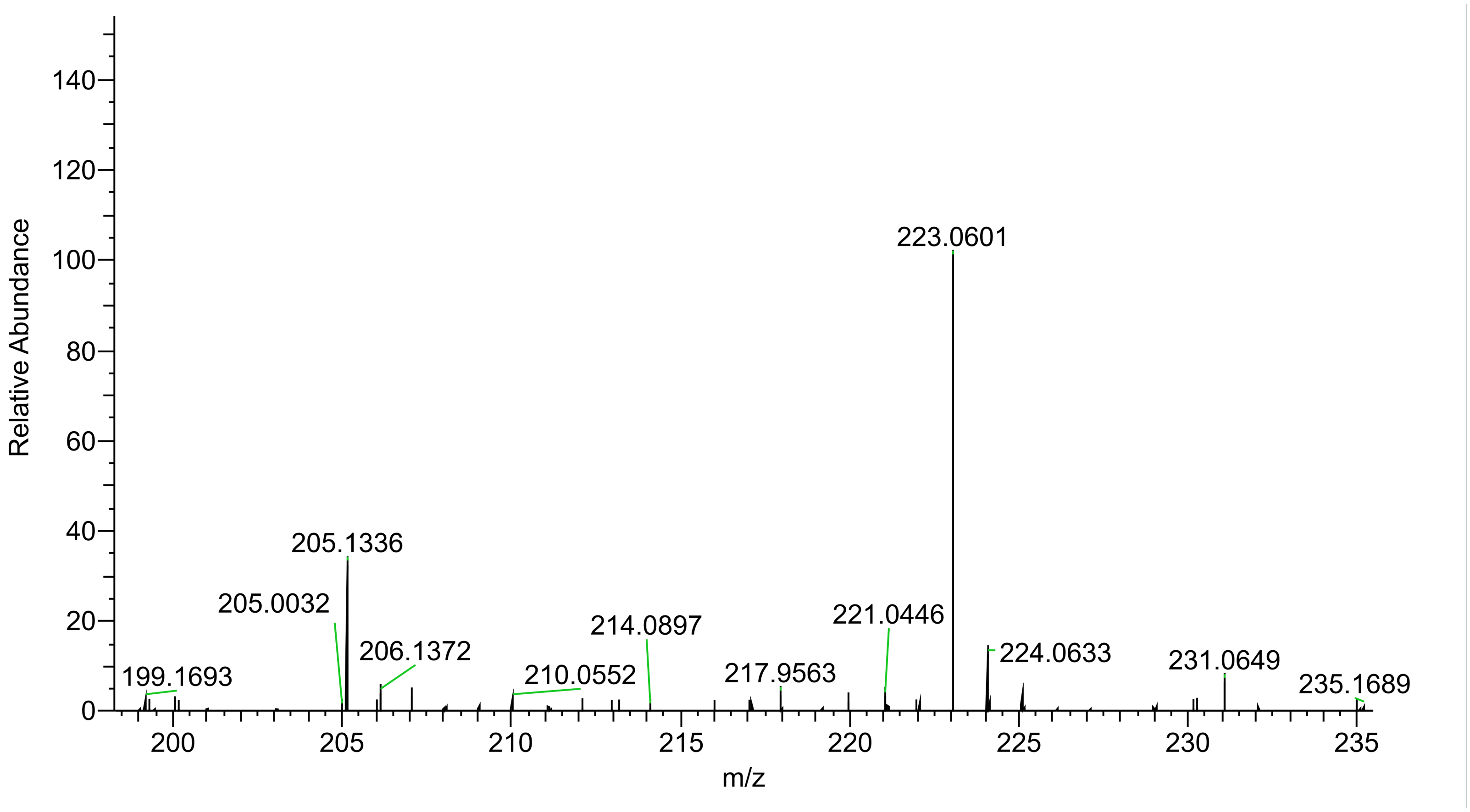

Figure S134. High resolution time-of-flight electrospray ionization mass spectrum of dialdehyde **8c**.

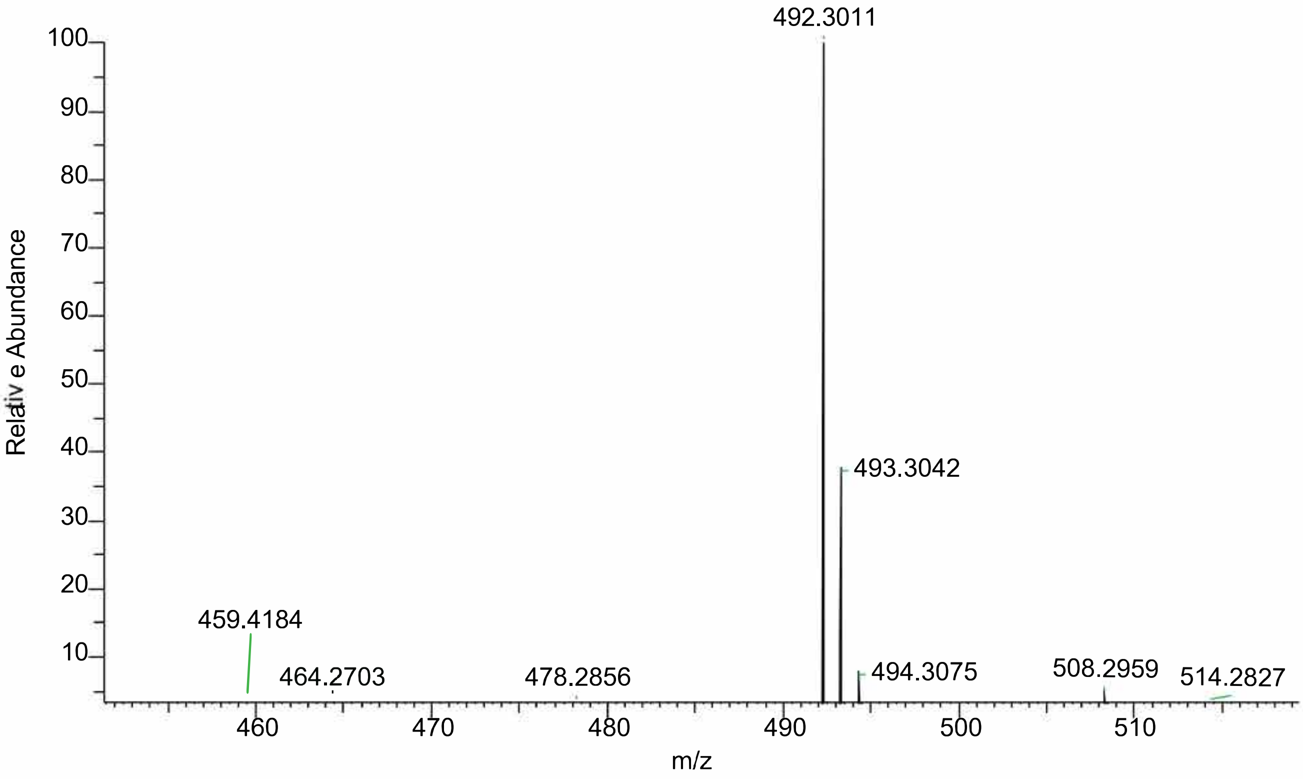

Figure S135. High resolution time-of-flight electrospray ionization mass spectrum of methoxybenziporphyrin **7a**.

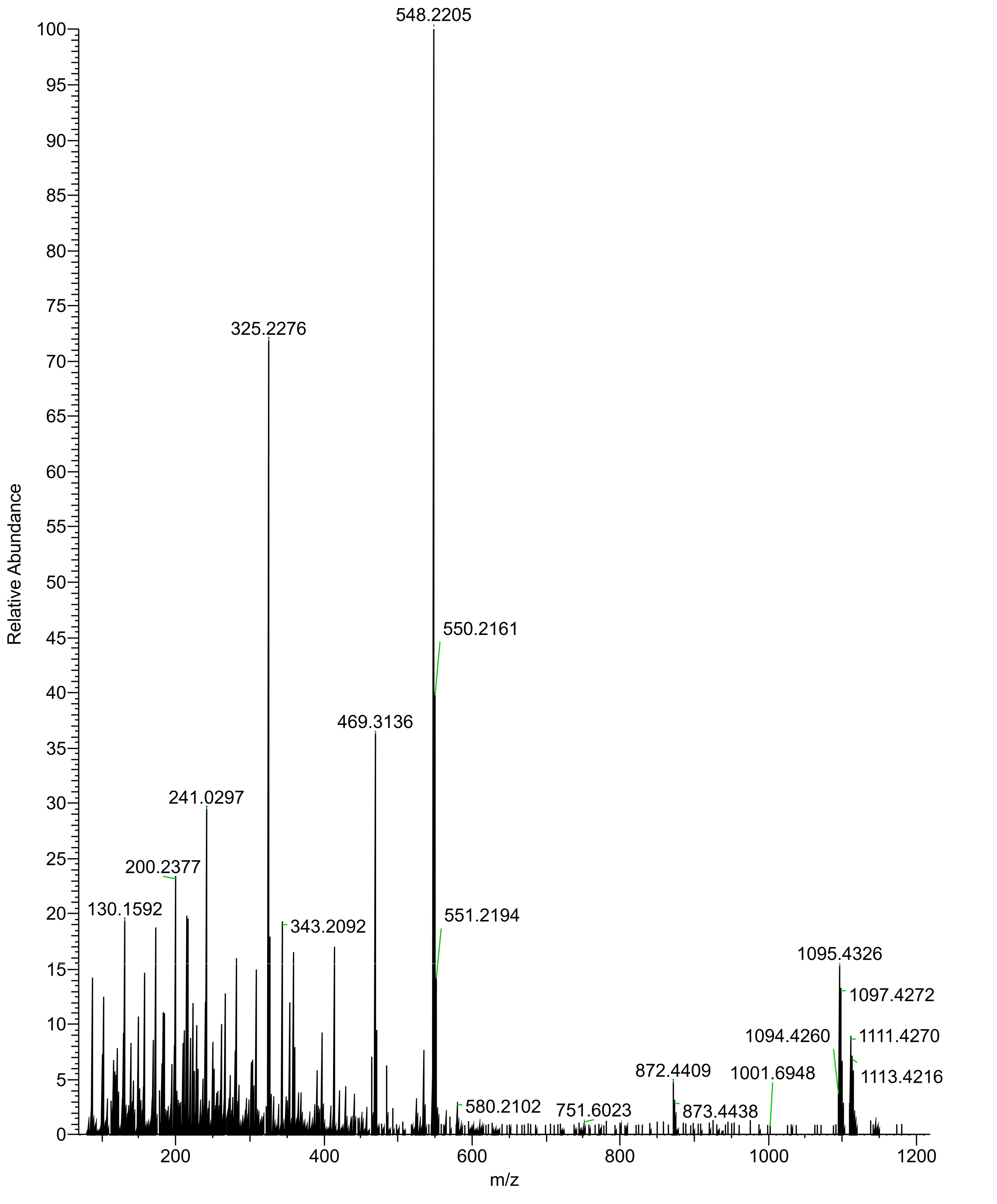

Figure S136. High resolution time-of-flight electrospray ionization mass spectrum of nickel complex **7aNi**.

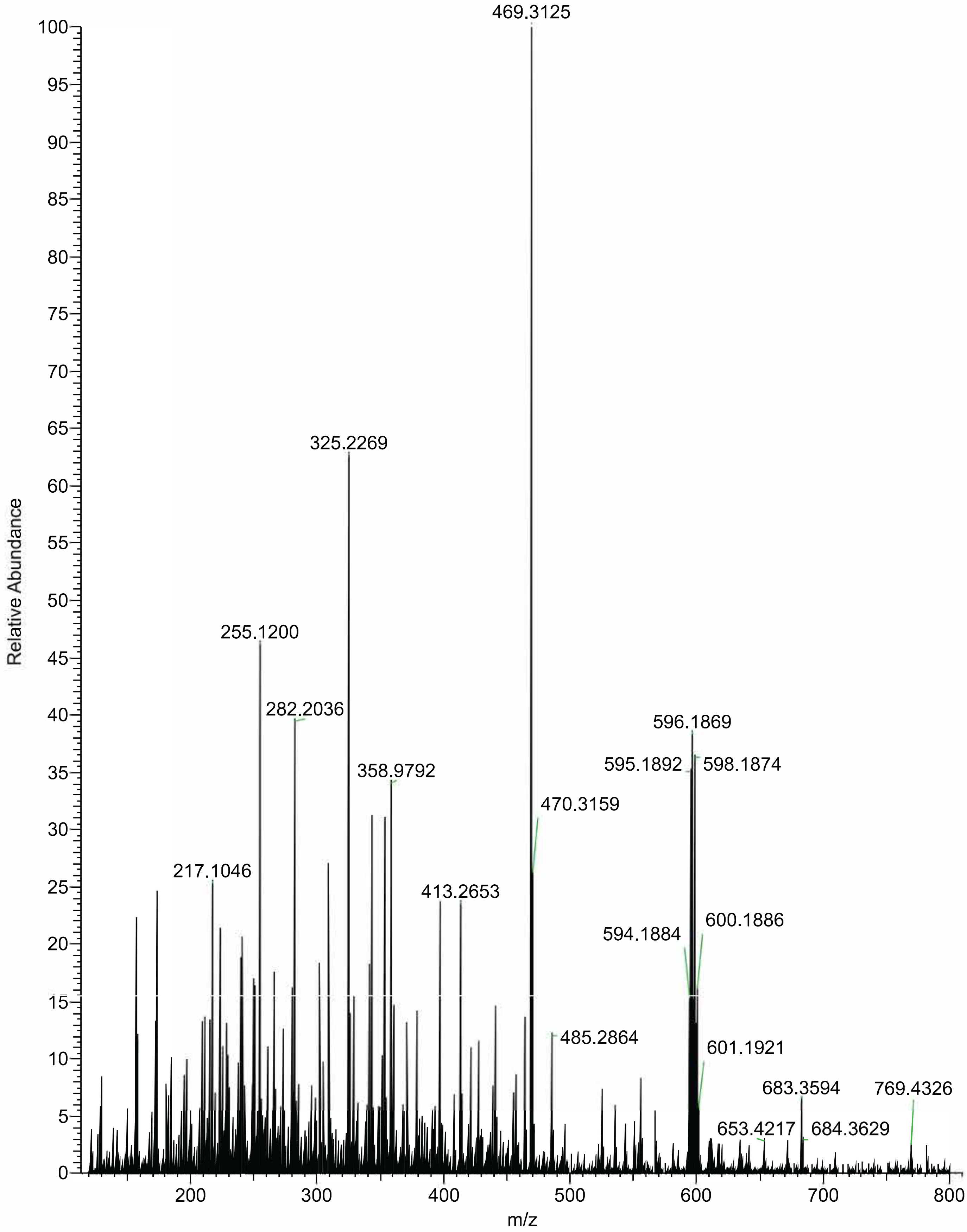

Figure S137. High resolution time-of-flight electrospray ionization mass spectrum of palladium complex **7aPd**.

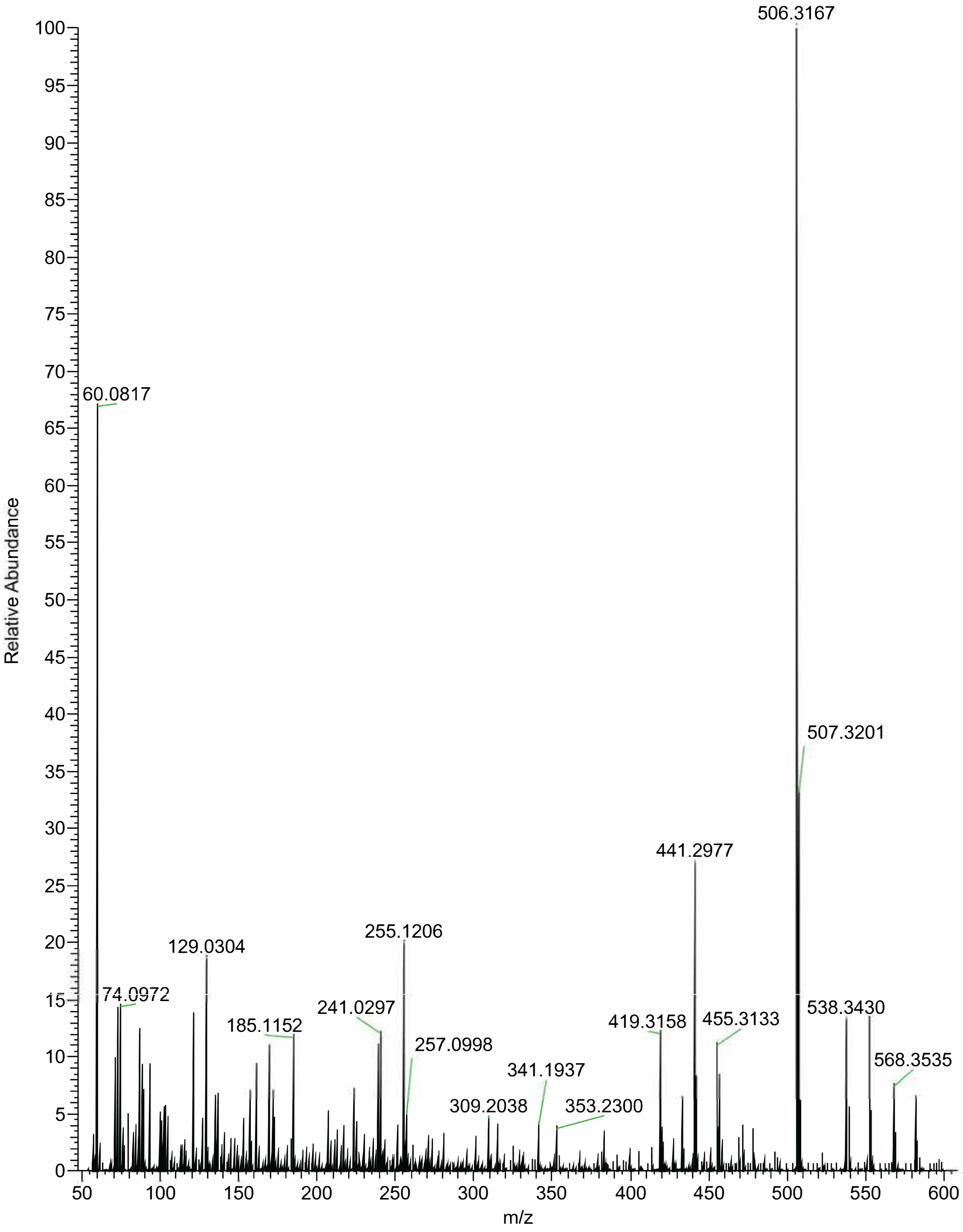

Figure S138. High resolution time-of-flight electrospray ionization mass spectrum of ethoxybenziporphyrin **7b**.

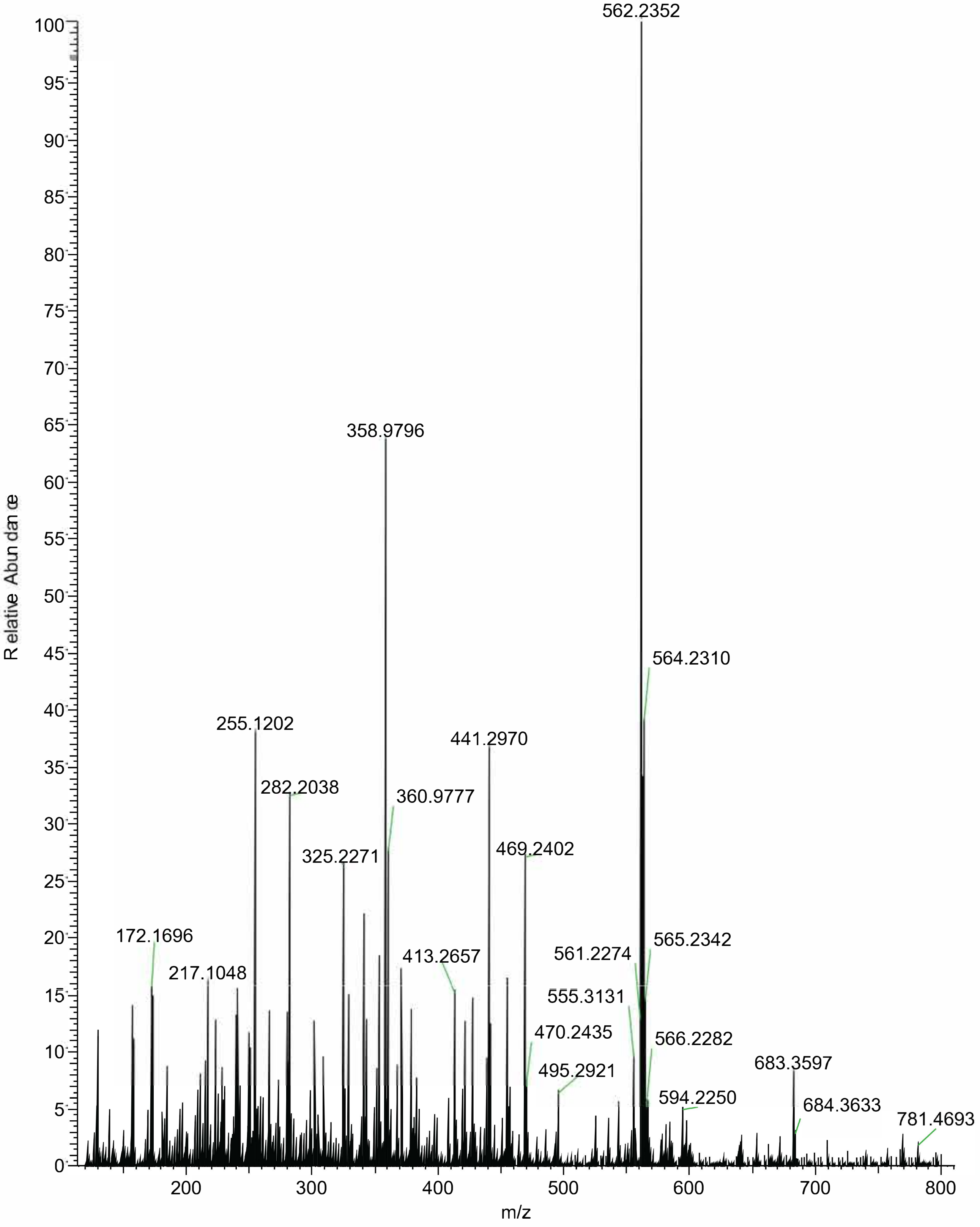

Figure S139. High resolution time-of-flight electrospray ionization mass spectrum of nickel complex **7bNi**.

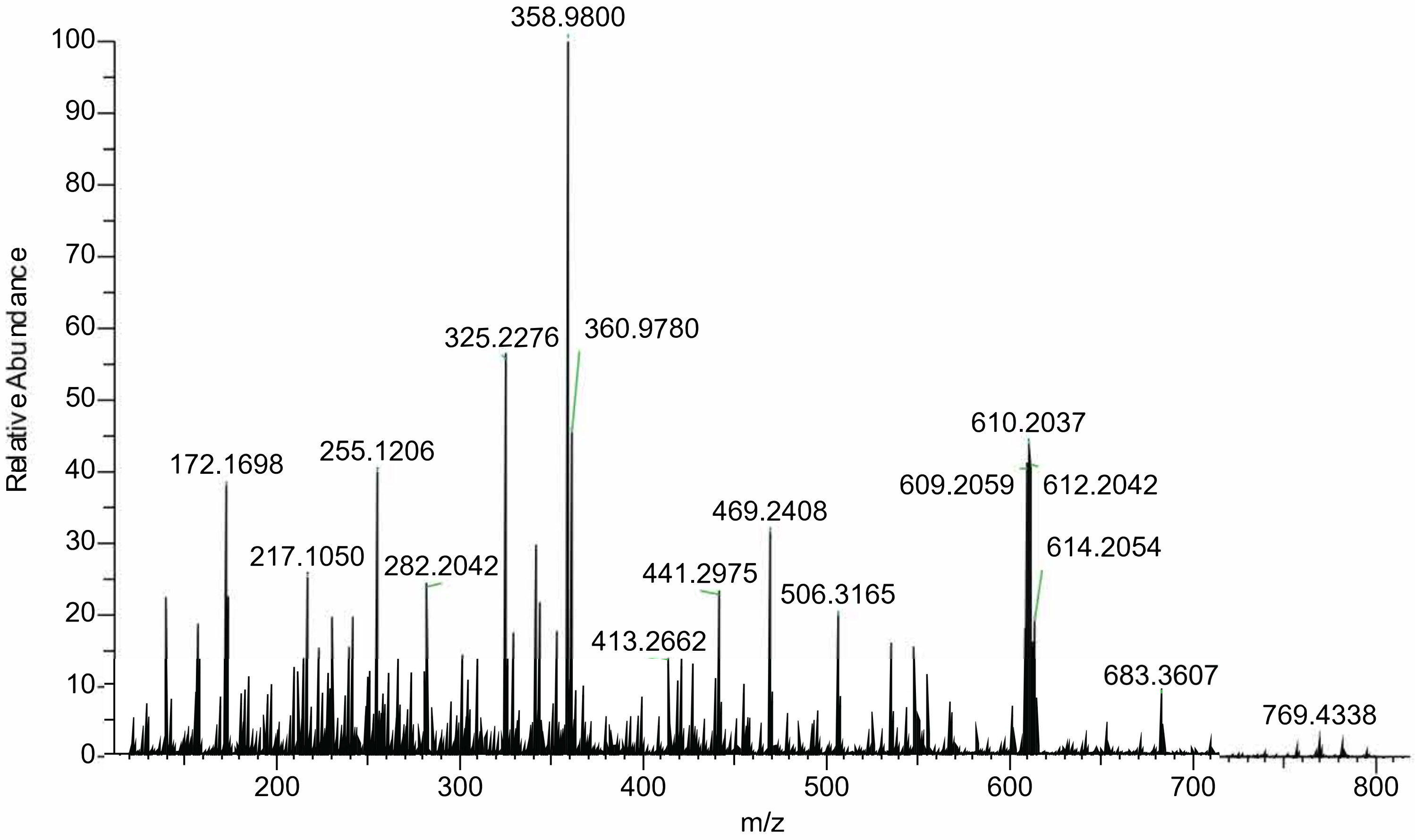

Figure S140. High resolution time-of-flight electrospray ionization mass spectrum of palladium complex **7bPd**.

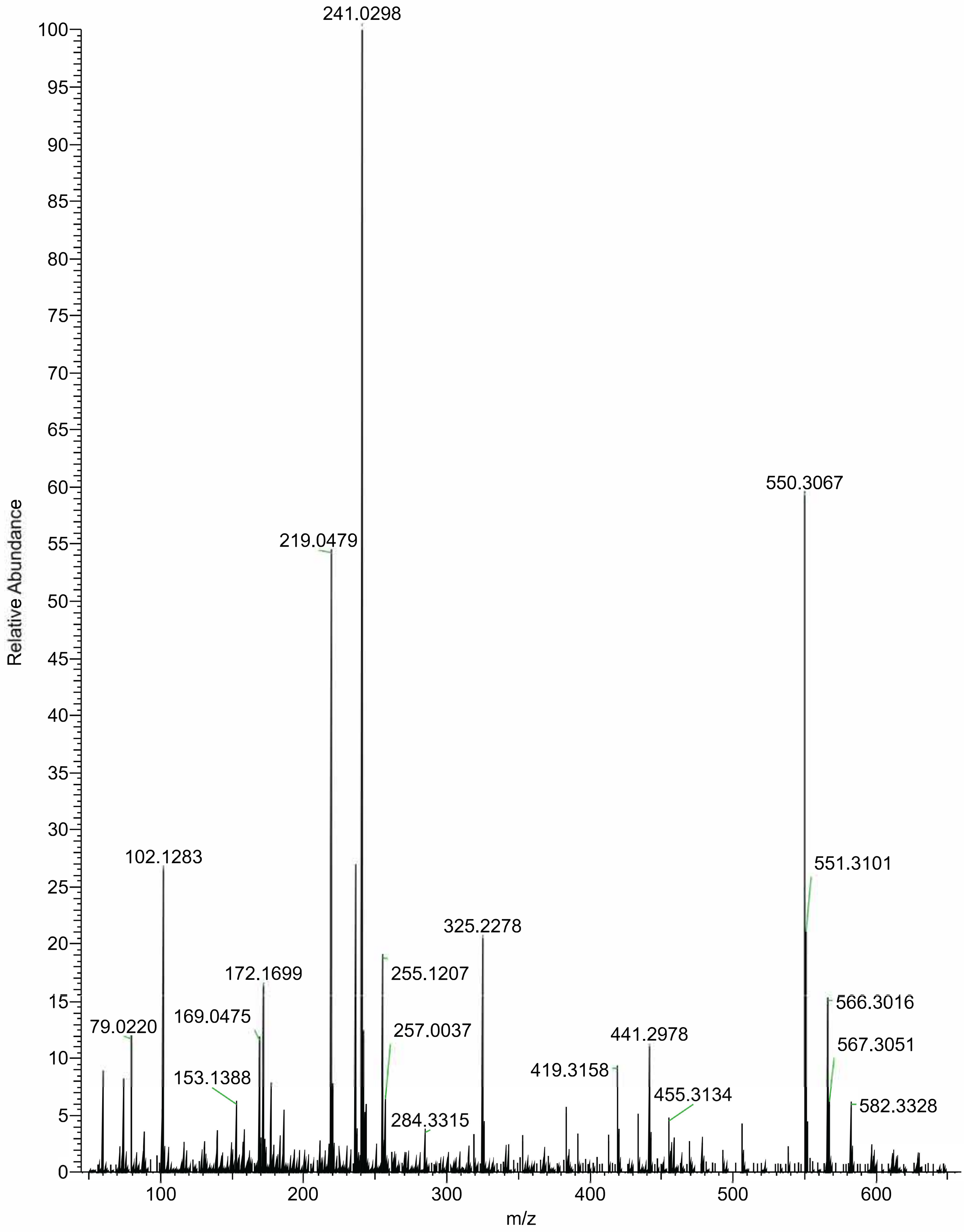

Figure S141. High resolution time-of-flight electrospray ionization mass spectrum of benziporphyrin **7c**.

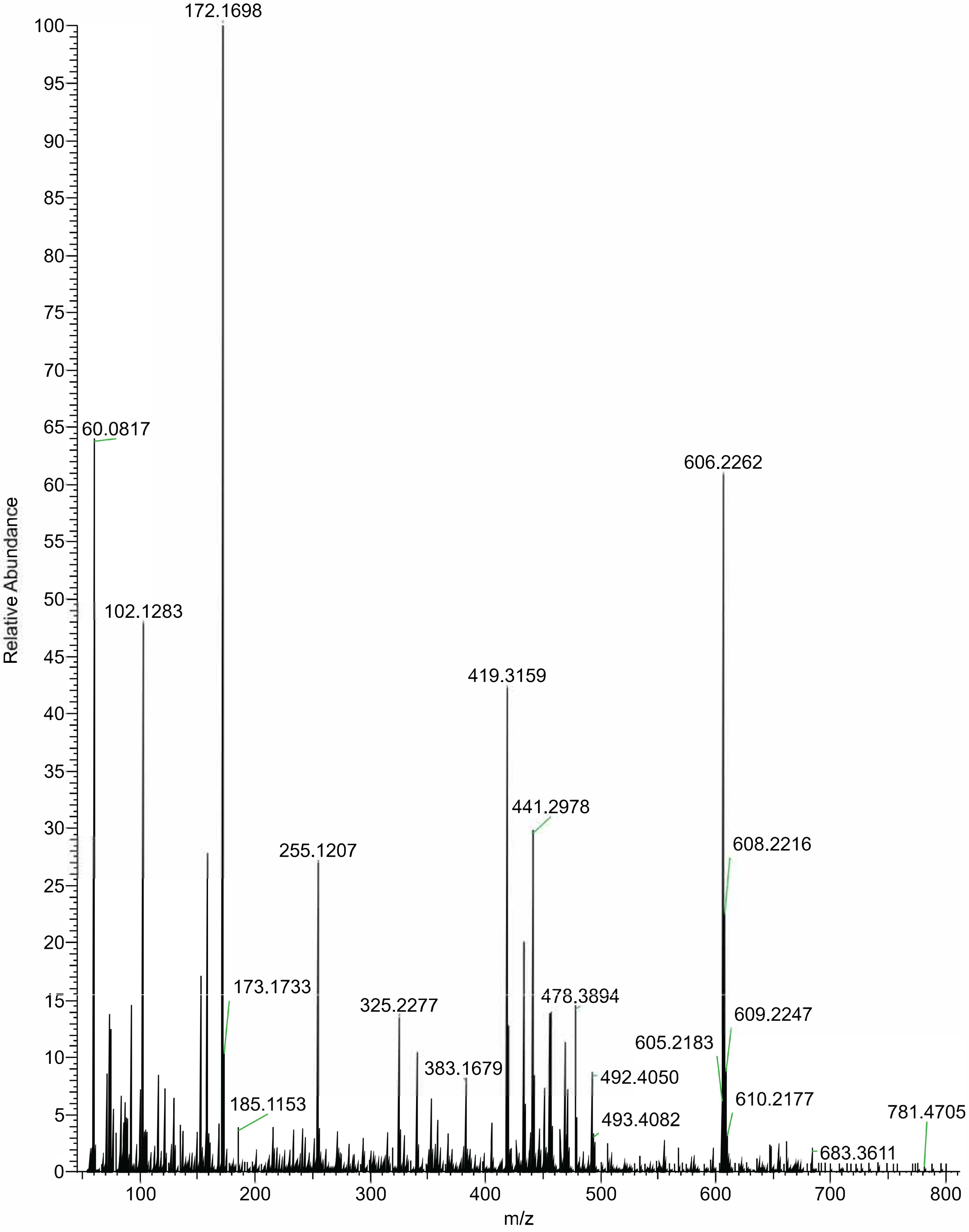

Figure S142. High resolution time-of-flight electrospray ionization mass spectrum of nickel complex **7cNi**.

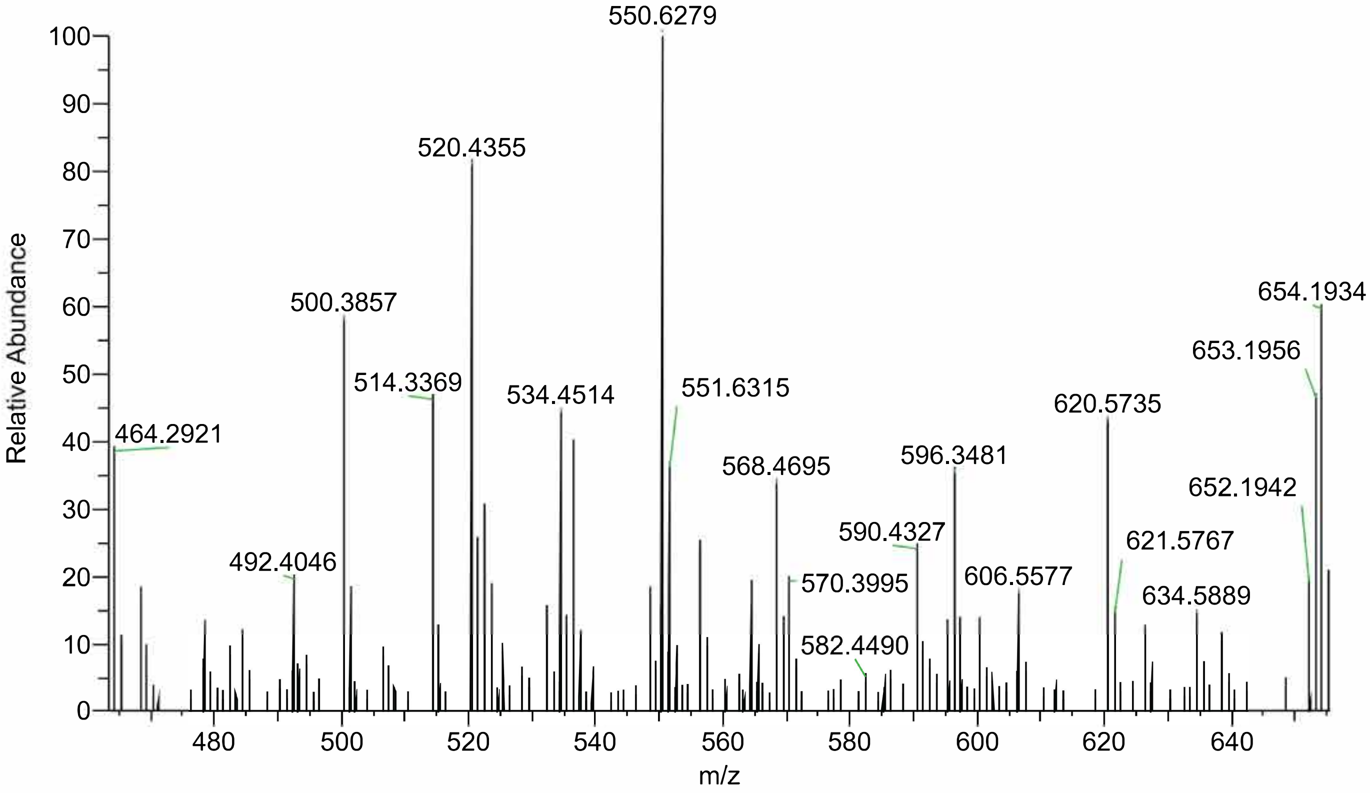

Figure S143. High resolution time-of-flight electrospray ionization mass spectrum of palladium complex **7cPd**.

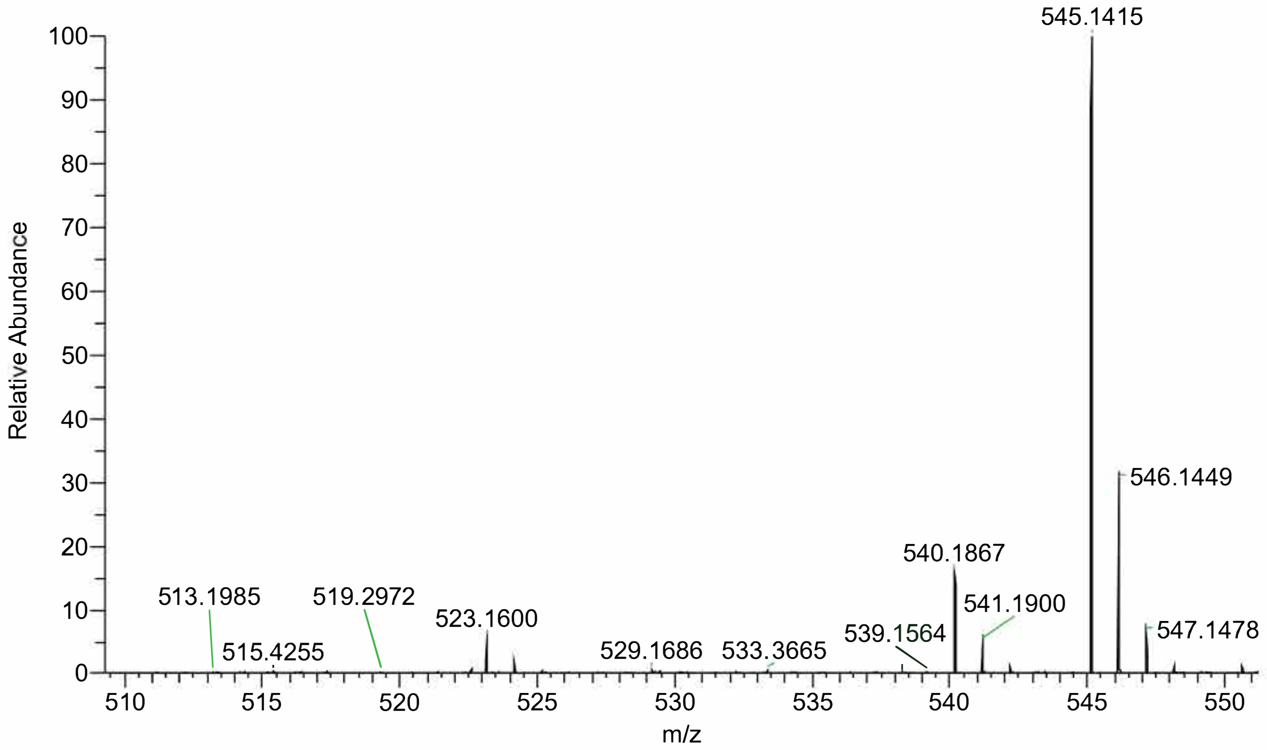

Figure S144. High resolution time-of-flight electrospray ionization mass spectrum of **16a**.

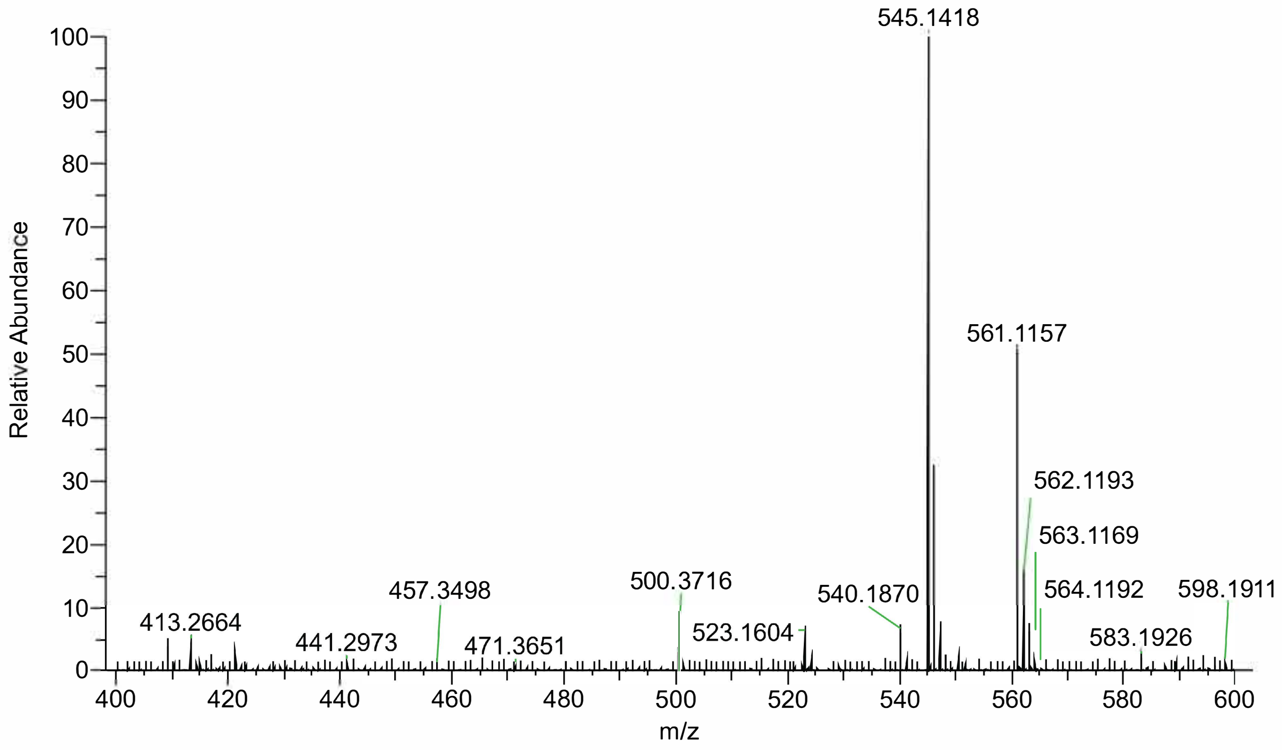

Figure S145. High resolution time-of-flight electrospray ionization mass spectrum of **16b**.

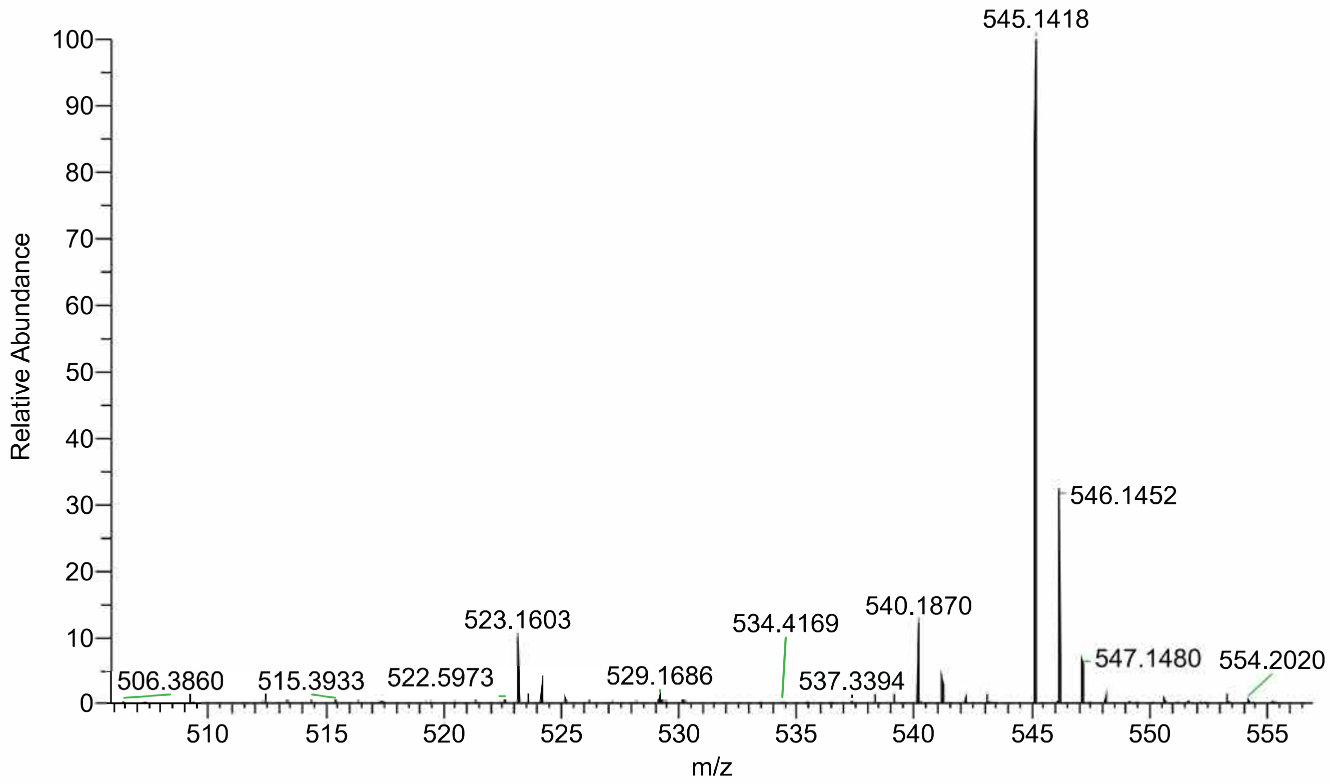

Figure S146. High resolution time-of-flight electrospray ionization mass spectrum of **16c**.

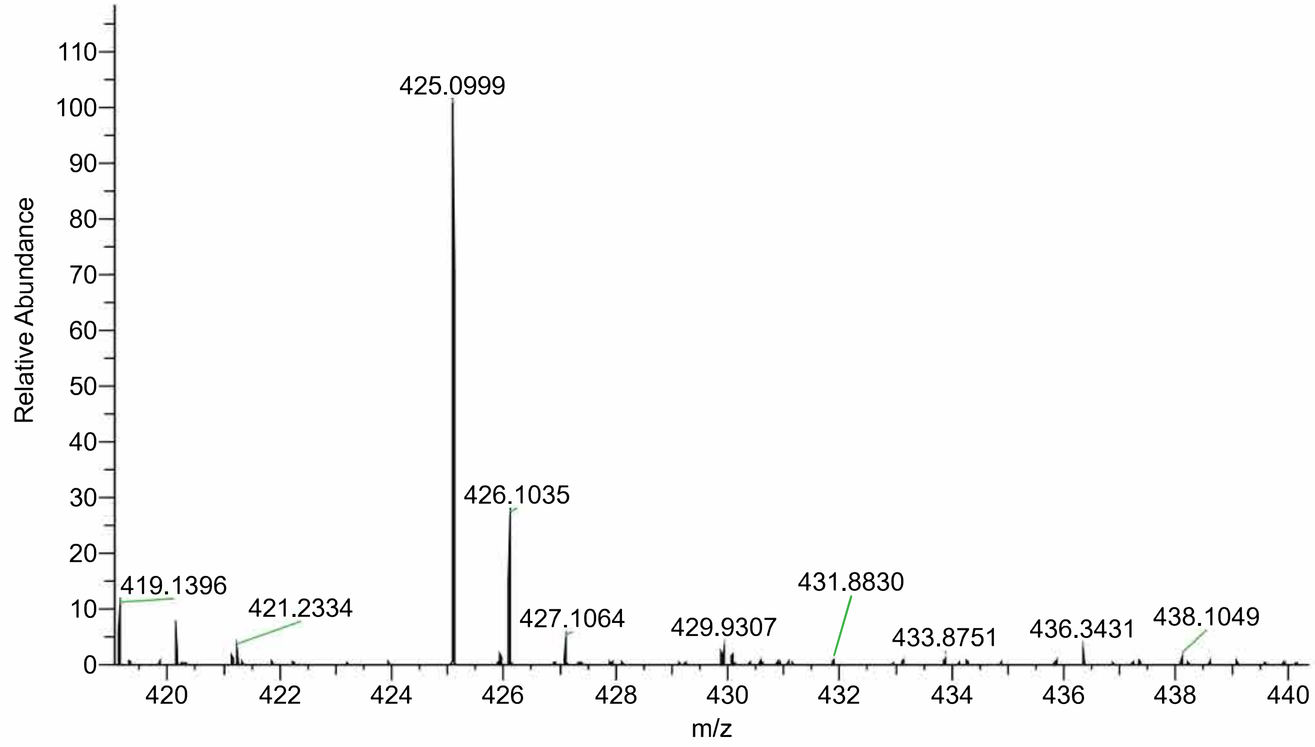

Figure S147. High resolution time-of-flight electrospray ionization mass spectrum of **18a**.

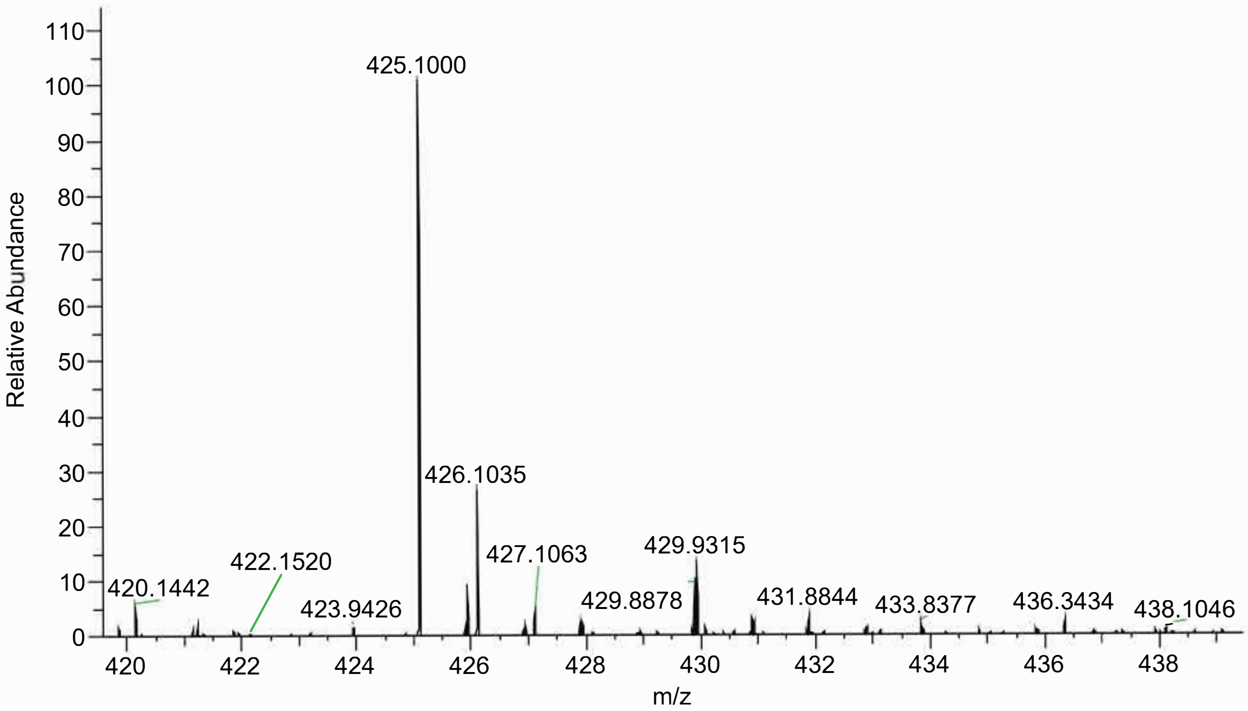

Figure S148. High resolution time-of-flight electrospray ionization mass spectrum of **18b**.

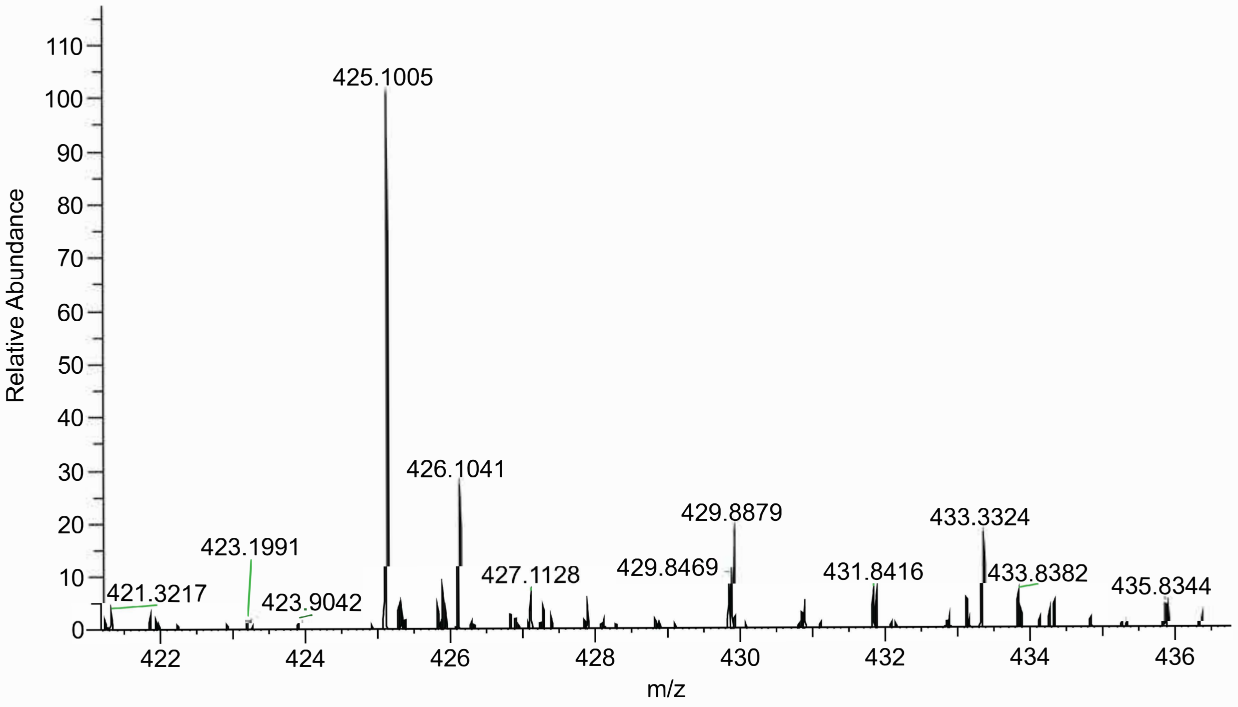

Figure S149. High resolution time-of-flight electrospray ionization mass spectrum of **18c**.
